# A study on the current status and influencing factors of psychological capital among medical undergraduates

**DOI:** 10.3389/fpsyg.2026.1830730

**Published:** 2026-10-30

**Authors:** Hongmei Li, Jingwen Xu, Ziheng Shen, Lizhong Liu, Qiyuan Huang

**Affiliations:** 1 The Second Clinical Medical School, Xuzhou Medical University, Xuzhou, Jiangsu, China; 2 School of Nursing, Wenzhou Medical University, Wenzhou, Zhejiang, China

**Keywords:** influencing factors, medical undergraduates, professional identity, professional satisfaction, psychological capital

## Abstract

**Objective:**

This study aimed to investigate the current status of psychological capital among medical undergraduates and its relationships with professional satisfaction and professional identity.

**Methods:**

A cross-sectional survey was conducted from August 2025 to December 2025 among medical undergraduates at a medical university in Jiangsu Province, China. Structured questionnaires were used to collect data on psychological capital, demographic characteristics, professional satisfaction, and professional identity. Data were analyzed using *t*-tests, analysis of variance (ANOVA) with Bonferroni-adjusted pairwise comparisons, and regression analysis.

**Results:**

A total of 837 valid questionnaires were collected. Results indicated that medical undergraduates exhibited a generally sound level of psychological capital, yet significant differences existed across academic years (*p* < 0.001), student leadership (*p* < 0.001), and place of origin (*p* = 0.002). Professional identity (*p <* 0.001) and academic year (*p =* 0.028) were significantly positively influenced on psychological capital among medical undergraduates, whereas non-student-leader status (*p =* 0.003) and rural origin (*p =* 0.032) were significantly negatively influenced on psychological capital. Professional satisfaction did not reach statistical significance (*p* = 0.383).

**Conclusion:**

This study revealed that the degree of professional identity served as a significant positive influence on psychological capital among medical undergraduates, whereas professional satisfaction had no significant influence. It was recommended to strengthen professional identity cultivation in medical education and optimize the selection and training mechanisms for student leaders to enhance medical undergraduates’ psychological capital and promote their comprehensive development.

## 1 Introduction

With the high-quality development of medical education (Li and Chen, 2025), the mental health and career development of medical undergraduates have attracted increasing attention (Eley and Slavin, 2024; Pfarrwaller et al., 2023). Medical students face significant challenges in their academic and professional journeys, including intense academic pressure, complex medical school environments, and ethical conflicts (Dyrbye et al., 2005; Yusefi et al., 2025). Depression, anxiety, burnout, and related distress remain prevalent in this population (Dyrbye et al., 2006; Lin et al., 2024; Eley and Slavin, 2024). Medical students may also be reluctant to seek professional psychological support because of stigma, concerns about confidentiality and professional consequences, and barriers to accessing services (Huang et al., 2019; Wang et al., 2023b), which may further affect their academic performance and career development. Therefore, enhancing medical students’ psychological quality and strengthening their ability to cope with stress have become critical issues in medical education (Nguyen et al., 2023; Santiago et al., 2024).

In recent years, psychological capital has gradually received increasing attention from scholars as a positive psychological resource. Psychological capital refers to a positive psychological state exhibited by individuals during their growth and development and comprises four core dimensions: self-efficacy, hope, resilience, and optimism (Luthans et al., 2007; Liu et al., 2024; Zhang, 2026). Psychological capital plays a crucial role in medical students’ academic and career development (Liu et al., 2023; Aldbyani et al., 2025). Earlier research indicated that occupational expectation pressure was the most prominent source of employment pressure among medical students and that resilience was associated with lower employment pressure (Wang and Ji, 2017). More recent evidence has further shown that employment pressure is closely related to the mental health of senior medical students and moderates the association between psychological capital and depressive symptoms (Huang et al., 2023). Psychological capital exhibits a significant positive predictive effect on employment expectations (Huang et al., 2025a, 2025b). Students possessing higher psychological capital often demonstrate greater confidence and motivation in career development and are more likely to secure desirable employment opportunities (Zhou et al., 2024; Shen et al., 2025). Simultaneously, enhancing psychological capital serves as an effective pathway to improve medical students’ core competencies (Liu et al., 2017; Wang et al., 2023a). A positive psychological state facilitates better mastery of professional knowledge and skills during learning, while also strengthening problem-solving abilities. Psychological capital exhibits a negative correlation with employment anxiety (Belle et al., 2022). Medical students with sufficient psychological capital maintain a more balanced mindset when facing employment pressures, reducing the negative impact of anxiety on job-seeking. Furthermore, a positive campus atmosphere significantly lowers academic burnout among medical students, and this effect is amplified when psychological capital levels are high (Yu et al., 2023), encouraging medical students to engage more actively in their studies. Overall, existing research has preliminarily demonstrated that psychological capital positively influences employment stress reduction, enhanced employment expectations, and improved academic performance among medical students, while also highlighting the interaction between campus environments and psychological capital (Aldbyani et al., 2025; Huang et al., 2023; Liu et al., 2023; Shen et al., 2025; Yu et al., 2023; Zhou et al., 2024). Collectively, these findings indicate that psychological capital is closely linked to medical students’ career development (Liu et al., 2023). Given that professional identity and professional satisfaction play guiding roles in medical students’ growth, clarifying their relationships with psychological capital is important for understanding medical undergraduates’ career development (Cruess et al., 2015; Liu et al., 2021; Zhang S. E. et al., 2022). However, the generalizability of previous findings on the association between psychological capital and professional identity remains controversial. Although studies among general practitioners and nursing interns reported positive correlations (Deng et al., 2023; Gong et al., 2025), variations in study populations, measurement instruments, and analytical strategies cast doubt on whether these conclusions could be extended to medical students. Moreover, empirical research specifically targeting medical undergraduates during this critical developmental stage is still scarce (Sarraf-Yazdi et al., 2024).

Psychological capital, as a key indicator of individuals’ positive psychological resources, has received increasing scholarly attention in diverse populations, including university students, nurses, teachers, and others (Bi and Jin, 2021; Sun et al., 2022; Li et al., 2023; Yao et al., 2023; Teng et al., 2024; Wu, 2024). However, the current developmental status of psychological capital among medical students, its specific influencing factors, and its relationships with professional satisfaction and professional identity have not been fully explored. Moreover, previous studies have been controversial regarding the impact of demographic characteristics (such as gender, place of origin, and grade) on psychological capital (Dong et al., 2025; Shi, 2018; Liu et al., 2025). Addressing these issues holds significant importance for optimizing medical education models, enhancing medical undergraduates’ mental health, and strengthening their professional adaptability. Therefore, this study aimed to investigate the relationships of medical students’ psychological capital with professional identity and professional satisfaction. Based on the factors influencing psychological capital, the study also sought to propose targeted educational strategies to enrich educational content and approaches and promote students’ healthy development and career growth. We hypothesized that (1) psychological capital would differ across demographic characteristics; and (2) professional identity and professional satisfaction would positively influence psychological capital after adjustment for demographic characteristics.

## 2 Materials and methods

### 2.1 Study design

This was a cross-sectional study (Schaie, 1965) designed to examine medical undergraduates’ psychological capital and its influencing factors. Professional identity and professional satisfaction were specified as core variables (Huang et al., 2023). The Strengthening the Reporting of Observational Studies in Epidemiology (STROBE) checklist was used to guide the reporting of this study (von Elm et al., 2007; Mokresh et al., 2024).

### 2.2 Participants and setting

The study employed convenience sampling (Jager et al., 2017) to recruit medical undergraduates enrolled at a medical university in Jiangsu Province, eastern China, from August 2025 to December 2025 (Li et al., 2024). Inclusion criteria: (1) Full-time undergraduate students enrolled at the university; (2) Voluntary participation after explicit explanation of the study objectives. Exclusion criteria: (1) Non-medical majors; (2) Students repeating a year, on leave of absence (including for military service), or international students.

*A priori* power analysis (G*Power 3.1) (Faul et al., 2009) for a multiple linear regression model (6 predictors, *f*^2^ = 0.15, α = 0.05, power = 0.95) indicated a minimum sample size of 146 participants. G*Power has also been used for sample-size estimation in medical-student research (Chiencharoenthanakij et al., 2024). To account for the convenience sampling design and potential invalid responses, a larger sample was recruited, resulting in 837 valid questionnaires for analysis.

### 2.3 Measures

#### 2.3.1 Psychological capital

Psychological capital was assessed using the Positive Psychological Capital Questionnaire (PPQ) developed by Zhang et al. (2010) and Wang S. et al. (2023). This scale comprises 26 items across four dimensions: self-efficacy, resilience, hope, and optimism. Responses were rated on a 7-point Likert scale, with 1 indicating “strongly disagree” and 7 indicating “strongly agree.” Higher scores indicate greater psychological capital and more positive attitudes among participants. Internal consistency was assessed using Cronbach’s alpha, originally introduced by Cronbach (1951) and recently applied in psychometric research among Chinese medical students (Zhang et al., 2025). The results of this study indicated that Cronbach’s alpha for this questionnaire was 0.93; Cronbach’s alpha coefficients for all four subscales were above 0.70.

#### 2.3.2 Professional satisfaction

Professional satisfaction was assessed using a single-item measure informed by previous research that used a single item to evaluate satisfaction with education among undergraduate medical students (Wissing et al., 2022). The item was “Overall, how satisfied are you with your chosen major?” A 4-point Likert scale was employed, with the following response options: Dissatisfied = 1; Indifferent = 2; Somewhat satisfied = 3; Satisfied = 4. Higher scores indicate greater professional satisfaction.

#### 2.3.3 Professional identity

Professional identity was assessed using a single-item measure, consistent with previous studies of pre-medical and medical students (Lin et al., 2021; Luman et al., 2022). The item was “Overall, how much do you identify with your major?” A 4-point Likert scale was employed, with the following response options: Not identified = 1; Somewhat identified = 2; Fairly identified = 3; Highly identified = 4. Higher scores indicate greater professional identity.

#### 2.3.4 General demographic information

A self-designed structured questionnaire was used to collect participants’ demographic data, including gender, academic year, place of origin, and whether they held a student leadership position (Artino et al., 2014; Pan and Ni, 2024).

### 2.4 Data collection

The questionnaires were developed, distributed, and collected via the Questionnaire Star online platform (Changsha Ranxing Information Technology Co., Ltd., n.d.), an online survey platform recently used in research involving Chinese medical undergraduates (Wang et al., 2024). A convenience sampling method was used at a single medical university, with the questionnaire link posted in the primary online communities for each undergraduate year. Participants first reviewed an electronic informed consent form and could only proceed to the main questionnaire after confirming their consent. A total of 844 questionnaires were initially collected. Data were exported from the Questionnaire Star backend in Excel format and underwent double-entry verification. Seven questionnaires were excluded from the analysis: five were removed due to identical responses to consecutive items, and two were excluded due to incomplete responses. All reported analyses were therefore based on the resulting sample of 837 undergraduates. This final dataset was imported into SPSS 27.0 (IBM Corp, 2020) for subsequent analysis. SPSS 27.0 has also been used in recent medical-student research (Schwarz et al., 2024).

### 2.5 Data analysis

Data analysis was conducted using IBM SPSS 27.0 software, with statistical tests performed at a significance level of α = 0.05. Harman’s single-factor test was conducted to assess potential common method bias across all 28 measurement items, comprising the 26 psychological capital items and the single items for professional satisfaction and professional identity. Five components had eigenvalues greater than 1, and the first component explained 39.83% of the total variance, below the 40% screening criterion (Lin et al., 2025). Based on this criterion, the test did not indicate substantial common method bias. Descriptive statistics, including frequencies, percentages, and mean ± standard deviation (mean ± SD), were used to summarize participants’ demographic characteristics and scores for psychological capital and its dimensions. Participants’ demographic characteristics are summarized in Table 1. *t*-tests (Student, 1908; Eames et al., 2024) and analysis of variance (ANOVA) (Fisher, 1925; Ranabhat et al., 2024) were employed to compare psychological capital scores across different demographic groups (Table 2). For academic year, significant ANOVA results were followed by pairwise comparisons using Fisher’s least significant difference (LSD) method (Supplementary Table S1). Bonferroni correction was applied to adjust for multiple comparisons within each outcome and control Type I error inflation, with adjusted *p* values capped at 1.0. Multiple linear regression analysis was employed to examine the factors influencing psychological capital. The psychological capital score served as the dependent variable, while demographic variables, professional satisfaction, and professional identity were treated as independent variables. Coding of independent variables is shown in Table 3. All independent variables were entered into the model simultaneously without prior variable screening.

**Table 1:** Composition ratio of questionnaire sources.

Variable	Category	Cases(%)
Gender	Male	337 (40.263)
	Female	500 (59.737)
Student leadership	Yes	594 (70.968)
	No	243 (29.032)
Academic year	2021 grade	86 (10.275)
	2022 grade	217 (25.926)
	2023 grade	135 (16.129)
	2024 grade	399 (47.670)
Place of origin	Urban	504 (60.215)
	Rural	333 (39.785)

**Table 2:** Comparison of psychological capital and its dimensions by demographic characteristics among medical undergraduates.

Variable	Cases	Dimensions of Psychological Capital														
		Self-efficacy			Resilience			Hope			Optimism			Psychological Capital		
		M ± SD	*t/F*	*p*	M ± SD	*t/F*	*p*	M ± SD	*t/F*	*p*	M ± SD	*t/F*	*p*	M ± SD	*t/F*	*p*
Gender																
Male	337	4.627 ± 1.030	0.539	0.590	4.480 ± 0.932	1.742	0.082	4.992 ± 1.142	−2.518	**0.012**	4.848 ± 1.229	−1.424	0.155	4.722 ± 0.943	−0.461	0.645
Female	500	4.589 ± 0.965			4.362 ± 1.000			5.185 ± 1.000			4.965 ± 1.075			4.752 ± 0.871		
Student leadership																
Yes	594	4.707 ± 0.943	4.740	**<0.001**	4.455 ± 0.945	2.121	**0.034**	5.205 ± 1.024	4.215	**<0.001**	5.023 ± 1.109	4.207	**<0.001**	4.827 ± 0.857	4.405	**<0.001**
No	243	4.353 ± 1.061			4.298 ± 1.035			4.868 ± 1.119			4.661 ± 1.175			4.528 ± 0.967		
Academic year																
2021 grade	86	4.603 ± 0.988	4.846	**0.002**	4.385 ± 0.977	3.060	**0.028**	5.153 ± 1.087	8.204	**<0.001**	4.845 ± 1.162	8.815	**<0.001**	4.727 ± 0.919	7.629	**<0.001**
2022 grade	217	4.426 ± 1.014			4.289 ± 0.918			4.825 ± 1.064			4.611 ± 1.160			4.524 ± 0.909		
2023 grade	135	4.521 ± 0.921			4.314 ± 0.906			5.077 ± 1.083			4.932 ± 1.120			4.688 ± 0.842		
2024 grade	399	4.729 ± 0.988			4.512 ± 1.017			5.261 ± 1.021			5.095 ± 1.098			4.878 ± 0.889		
Place of origin																
Urban	504	4.707 ± 0.998	3.734	**<0.001**	4.487 ± 0.988	2.833	**0.005**	5.179 ± 1.065	2.404	**0.016**	4.972 ± 1.158	1.682	0.093	4.818 ± 0.915	3.079	**0.002**
Rural	333	4.448 ± 0.962			4.293 ± 0.943			4.999 ± 1.052			4.836 ± 1.109			4.623 ± 0.866		

Bold *p* values indicate statistical significance (*p* < 0.05).

**Table 3:** Coding of independent variables in the regression model.

Variable	Coding used in the model
Gender	Male = 1; Female = 2
Academic year	2021 grade = 1; 2022 grade = 2; 2023 grade = 3; 2024 grade = 4
Place of origin	Rural = 1; Urban = 0
Student leadership	Yes = 1; No = 2
Professional satisfaction	Dissatisfied = 1; Indifferent = 2; Somewhat satisfied = 3; Satisfied = 4
Professional identity	Not identified = 1; Somewhat identified = 2; Fairly identified = 3; Highly identified = 4

### 2.6 Ethical consideration

Ethical approval was obtained from the Biological and Medical Research Ethics Committee of the first author’s institution before the study commenced. All methods were performed in accordance with the Declaration of Helsinki. Before confirming the electronic informed consent form, the participants fully understood the purpose, process, and benefits of the study, as well as their right to withdraw from the study at any time.

## 3 Results

### 3.1 General characteristics of research participants

A total of 844 questionnaires were distributed, with 837 valid responses collected, yielding a response rate of 99.2%. Among these: 337 (40.26%) were male; 594 (70.97%) were student leaders; 399 (47.67%) were first-year undergraduates, 135 (16.13%) were second-year undergraduates, 217 (25.93%) were third-year undergraduates, and 86 (10.27%) were fourth-year undergraduates; 504 (60.22%) resided in urban areas (Table 1). The overall mean psychological capital score was 4.74 ± 0.90, with hope (5.11 ± 1.06) scoring the highest and resilience (4.41 ± 0.97) the lowest. Hope (5.11 ± 1.06) and Optimism (4.92 ± 1.14) demonstrated comparatively higher levels relative to Self-Efficacy (4.60 ± 0.99) and Resilience (4.41 ± 0.97).

### 3.2 Comparison of differences in psychological capital and its dimensions across different demographic variables among medical undergraduates

Independent-samples *t*-tests were used to compare total psychological capital scores and scores for each dimension by gender, student leadership, and place of origin, as shown in Table 2. Hope dimension scores exhibited significant gender differences (*p* = 0.012), with female students demonstrating higher levels of hope; Self-efficacy, hope, optimism, and total psychological capital scores exhibited extremely significant differences based on student leaders (*p* < 0.001). Results indicated that student leaders had higher levels of self-efficacy, resilience, hope, optimism, and total psychological capital compared to non-student leaders. This finding was consistent with prior research indicating that leadership experiences in medical school shape implicit leadership theories and traits, which in turn influence professional identity and psychological engagement (Amini et al., 2025).

Analysis of variance was used to compare total psychological capital scores and scores for each dimension across academic years. As shown in Table 2, regarding academic year, medical students exhibited significant differences in total psychological capital scores and Hope/Optimism dimension scores (*p* < 0.001), Self-Efficacy dimension scores (*p* = 0.002), and Resilience dimension scores (*p* = 0.028). Further pairwise comparisons revealed that compared with students in the 2022 grade, those in the 2024 grade had significantly higher scores for total psychological capital (Adjusted *p* < 0.001), self-efficacy (Adjusted *p* = 0.002), resilience (Adjusted *p* = 0.039), hope (Adjusted *p* < 0.001), and optimism (Adjusted *p* < 0.001). The *t*-tests showed that medical students from urban areas had significantly higher scores in self-efficacy (*p* < 0.001), resilience (*p* = 0.005), hope (*p* = 0.016), and total psychological capital (*p* = 0.002) compared to their rural counterparts.

### 3.3 Analysis of influencing factors on psychological capital among medical undergraduates: professional satisfaction, professional identity, and demographic variables

Using psychological capital as the dependent variable and professional satisfaction, professional identity, and demographic variables as independent variables respectively, multiple linear regression analysis was conducted to examine the predictive role of professional satisfaction, professional identity, and demographic variables on psychological capital. Prior to the regression analysis, diagnostic tests were performed. The variance inflation factor (VIF) for all independent variables ranged from 1.02 to 1.53, indicating no multicollinearity concerns. The Durbin-Watson value was 2.03, suggesting no autocorrelation among the residuals. The coding of independent variables is presented in Table 3; research findings were shown in Table 4. Table 4 indicated that academic year (*B* = 0.060, *p* = 0.028), place of origin (*B* = −0.127, *p* = 0.032), student leadership role (*B* = −0.196, *p* = 0.003), and degree of professional identification (*B* = 0.422, *p* < 0.001) were identified as factors influencing medical students’ psychological capital. Both academic year and professional identification were positively correlated with psychological capital, meaning that the higher the academic year and professional identification, the stronger the psychological capital. In addition, student leadership and place of origin were also significantly related to psychological capital. Students with experience in student leadership positions and those from urban areas scored higher in psychological capital.

**Table 4:** Multiple linear regression of psychological capital on professional satisfaction, professional identity, and demographic variables.

Variable	Unstandardized coefficient		Beta	*t*	*p*	95% CI		VIF
	B	SE				Lower	Upper	
(Constant)	3.332	0.226	–	14.741	**<0.001**	2.889	3.776	–
Academic year	0.060	0.027	0.072	2.201	**0.028**	0.007	0.114	1.054
Student leadership No = 2; Yes = 1	−0.196	0.065	−0.099	−3.023	**0.003**	−0.324	−0.069	1.057
Professional satisfaction	0.044	0.050	0.034	0.872	0.383	−0.055	0.143	1.511
Professional identity	0.422	0.051	0.325	8.260	**<0.001**	0.322	0.523	1.523
Gender Female = 2; Male = 1	0.010	0.060	0.005	0.168	0.867	−0.107	0.127	1.042
Place of origin Rural = 1; Urban = 0	−0.127	0.059	−0.069	−2.152	**0.032**	−0.243	−0.011	1.016

Bold *p* values indicate statistical significance (*p* < 0.05).

## 4 Discussion

This study explored the relationship between psychological capital and both professional identification and professional satisfaction through a cross-sectional survey. The primary finding of this study was that the degree of professional identification serves as a significant positive impact of psychological capital among undergraduate medical students, whereas professional satisfaction exerts no discernible influence. In addition, we also conducted a categorized exploration of the psychological capital levels among different subgroups of gender, student leadership, academic year and place of origin. Therefore, based on this understanding, we can develop more targeted educational strategies, focusing on enhancing psychological capital and professional identification, in order to promote their healthy development and career progress. This suggested that in medical education, interventions should focus on professional identity rather than professional satisfaction in order to enhance psychological capital.

In this study, medical students’ psychological capital was generally favorable compared with existing research findings on university students’ psychological capital (Fu and Zhang, 2010; Tang and He, 2022). The current state of medical students’ psychological capital indicated that development remains uneven across its various dimensions. Among these dimensions, self-efficacy and resilience levels were comparatively lower, whereas hope and optimism levels were comparatively higher. This study shows that medical students possess a relatively favorable level of psychological capital. Previous research indicates that they have stronger positive psychological resources, which enables them to cope with negative emotions more skilfully, revealing that their subjective well-being has currently surpassed the average level (Tang et al., 2012; Yu et al., 2026). However, the relative shortfall in self-efficacy and resilience indicated room for improvement in areas essential for career success, including planning, self-actualization capabilities, a sense of achievement, and the capacity to counteract setbacks and overcome emotional fragility. This underscored the importance of cultivating self-efficacy and resilience dimensions in medical students for long-term career planning and resilience in adversity (Chang, 2014; Wang et al., 2025).

Meanwhile, this study also conducted analyses for each subgroup. It pointed out that female students have higher levels of hope compared to male students. Medical students from urban areas exhibited significantly higher scores in self-efficacy, resilience, hope, and overall psychological capital compared to students from rural areas. Additionally, student leaders demonstrate higher levels of self-efficacy, resilience, hope, optimism, and overall psychological capital compared to non-student leaders.

Research findings indicated that female students exhibit higher levels of hope than their male counterparts, a phenomenon potentially attributable to statistical effects stemming from the distinctive nature of medical studies. Overall, junior medical students demonstrated superior psychological capital and its constituent dimensions compared to senior cohorts, except for the grade 2021. The underlying relationship warrants further investigation. Relatively affluent family backgrounds and upbringings provided greater opportunities and scope for developing psychological capital and its various dimensions. Medical students from urban areas scored significantly higher than their rural counterparts in self-efficacy, resilience, hope, and overall psychological capital. This is consistent with existing research (Shi, 2018; Chen and Zhang, 2026). However, we also notice that in the same year, literature from almost the same region presents completely opposite conclusions, asserting that medical students from rural areas scored significantly higher than those from urban areas (Yu et al., 2026). We note that the samples in four studies, including this study, all come from a single medical university. This suggests that in subsequent research, we need to be mindful of avoiding selection bias and be cautious of its potential negative impact on the credibility of the research results (Shi, 2018; Chen and Zhang, 2026; Yu et al., 2026; Baumgartner et al., 2026; Lyu et al., 2026; Wang et al., 2026). Students from urban backgrounds received comparatively greater familial and societal support, possessing both the financial means and robust backing to pursue higher aspirations. Their clearer future planning facilitated the comprehensive development of psychological capital.

Findings from the aforementioned study indicated that student leaders exhibit higher levels of self-efficacy, resilience, hopefulness, optimism, and overall psychological capital compared to non-student leaders. This aligned with prior research suggesting that student leadership experiences within an educational context effectively enhance psychological capital and employability (Shi, 2018; Sun et al., 2025). Regarding employability, students with student cadre experience scored higher on interpersonal psychological capital. This may relate to their increased opportunities to interact with teachers, peers, and members of society, thereby better developing interpersonal skills and coping strategies (Li and Cao, 2011; Chen and Zhang, 2026).

Research findings indicated that professional identity served as an influencing factor for medical students’ psychological capital, whereas professional satisfaction was not significantly associated with psychological capital after adjustment for the other variables. Enhancing professional satisfaction offered certain benefits for medical students’ future development. Students demonstrating higher satisfaction with their chosen discipline typically cultivated robust learning abilities and a solid professional foundation during their academic years. Consequently, they possessed clearer career objectives and stronger competitiveness, thereby reducing the incidence of career decision-making difficulties among medical interns (Ma et al., 2024).

Results found in this research also indicated that medical students from urban areas, those holding student leadership roles, and those with higher professional identity exhibited greater psychological capital. Also, we found that medical students in 2024 grade exhibited greater psychological capital than those in 2022 grade. This correlated with influences from the pandemic era and post-pandemic context. Perceptions of the COVID-19 pandemic positively predicted medical students’ professional employment intentions: heightened awareness of the pandemic correlated with stronger commitment to pursuing careers within their discipline. Concurrently, social support and psychological capital exerted mediating and chain-mediating effects within this influence (Li J. L. et al., 2022). Against this backdrop, research has begun exploring and envisioning medical student training, advocating for novel approaches and the prioritization of funding and policies toward medical education (Zhang and Tong, 2020; Gu et al., 2025).

Conducting career planning guidance for university students, enabling them to undertake systematic career planning during their undergraduate years, represented an effective approach to strengthening ideological and political education among higher education students and enhancing graduate employment guidance under new circumstances (Lou and Zhao, 2002; Zheng, 2025). Previous research indicated that gender, academic year, student leadership role, basis for professional choice (or professional satisfaction), perception of career development direction, sources of career knowledge, and career awareness were direct factors influencing university students (including medical students) in forming clear career plans, while knowledge understanding, and learning ability are the most important employability for college graduates (Meng, 2020; Jiang et al., 2023). Psychological capital exerted a significant mediating effect on university student volunteers’ motivation and work flourishing (Li J. et al., 2022). Similarly, psychological capital played a role in fostering gratitude and professional ethos among medical students (Sun et al., 2018; Zhang H. J. et al., 2022). Consequently, it can be considered that the cultivation and enhancement of psychological capital played a certain role in the career planning and development of medical students. Currently, many universities have launched career planning guidance courses for college students, aiming to advance career planning work and cultivate outstanding medical students through intervention and guidance.

Medical students scored relatively low on self-efficacy and resilience dimensions, representing critical areas for enhancement within psychological capital. Educational interventions could include foundational mental health courses, confidence-building exercises, and the integration of simulated adversity scenarios within high-pressure settings like career planning, alongside refined feedback mechanisms to bolster self-efficacy and resilience (Xie et al., 2025).

Medical students’ mental health warranted proactive attention, with psychological capital enhancement serving as a pathway to develop core competencies. This approach fostered medical students better aligned with the bio-psycho-social model (Liu et al., 2017; Chen et al., 2025). Existing research indicated that medical students with higher levels of positive psychological capital demonstrated greater gratitude and stronger professional ethos, manifesting in career recognition and work engagement (Sun et al., 2018; Xu et al., 2023). Currently, some universities have implemented an undergraduate mentor system. By assigning professional mentors to each medical student, academic guidance and career planning advice are provided, thereby enhancing students’ sense of belonging and identification with their discipline.

Based on this study’s findings, tailored psychological capital development programs could be designed according to gender-specific characteristics. For male students, emphasis should be placed on enhancing hope and optimism dimensions, while female students could focus on improving self-efficacy and resilience. Similarly, phased psychological capital development plans should be formulated according to the characteristics of medical students at different academic years. For junior students, the emphasis should be on cultivating professional identity and foundational psychological qualities; for senior students, the focus should shift to enhancing career planning capabilities and stress resilience.

Research indicated that psychological capital levels among urban graduates exceeded those of rural graduates, correlating with family and societal support. Higher family satisfaction correlated with greater psychological capital scores among medical students. From a family support perspective, guiding parents to create favorable family environments, strengthening communication with student families, and securing familial support and understanding can bolster psychological capital, enabling effective early intervention before psychological issues arise (Zhou et al., 2017; Gao et al., 2026). The moderation analysis in previous research showed that family support played a moderating role in the conditional influence of psychological capital. Also, psychological capital negatively moderated the positive relationship between interpersonal sensitivity and depression (Xu et al., 2022). Optimizing environments conducive to psychological capital development and providing greater support and resources were crucial for enhancing medical students’ psychological capital. Existing research indicated that university students’ perceptions of an imbalance between effort and reward were significantly negatively correlated with positive psychological capital. This suggested that the greater the perceived imbalance between effort and reward, the lower the level of positive psychological capital among university students (Zhu and Tao, 2022). Regarding social support, providing scholarships, grants, and other forms of assistance to students facing financial hardship alleviates their economic burdens, enabling them to focus more intently on their studies and the enhancement of psychological capital.

The dimensions of hope and optimism represented the strengths within medical students’ psychological capital. Cultivating a positive mindset and sense of hope among medical students helped them better cope with academic and life pressures. For instance, offering courses in positive psychology can teach students how to develop a positive outlook, maintain optimistic emotions, and enhance their sense of hope.

## 5 Contributions and limitations

### 5.1 Contributions

The significant positive impact of professional identity on the psychological capital of medical students revealed in this study—consistent with recent findings on the intricate relationships among professional identity, micro-learning environments, and psychological capital (Gong et al., 2025), as well as the mediating effect of psychological capital in the relationship between interpersonal relationships and mental health among medical students (Dong et al., 2025)—can provide strong theoretical support for medical schools to deepen the cultivation of professional identity and optimize psychological intervention strategies in education and teaching. At the same time, the findings of this study regarding the influence of demographic characteristics such as student leadership experience, place of origin, and grade level on psychological capital can provide practical support for educational administrators to develop differentiated and precise mental health and career development guidance programs for different groups.

### 5.2 Limitations

This study has several limitations: (i) Given the cross-sectional nature of this study, causal relationships between variables remain inconclusive (Savitz and Wellenius, 2023). And this study used a single-center survey, which limits the generalization of the conclusions. Future research may employ multi-center longitudinal investigations to further elucidate the underlying determinants of psychological capital (Dudasova et al., 2024). (ii) The measurement of students’ professional satisfaction and professional identity in this study relied on a single multiple-choice question each. This may introduce a degree of information bias (Mund et al., 2022). Similarly, the data collected by this research institute are derived from outcome measures reported subjectively by the study subjects, which may introduce a degree of selective reporting bias (Wang et al., 2026). Therefore, it is recommended that future research employ more objective and authoritative scales for data collection. And we suggest that future research include objective measures like physiological and behavioral indicators to fully explore the factors affecting psychological capital (Wespi et al., 2023). (iii) The results collected in this study exhibit a certain degree of demographic imbalance. There were relatively fewer non-student leaders, and the number of individuals included in the statistics varies across different grades. To avoid selection bias, future research should improve data collection methods and could consider using stratified sampling to make sure everyone has an equal chance of being included in the sample (Arrogante, 2022).

## 6 Conclusion

This study investigated the current state of psychological capital among undergraduate medical students and its relationship with professional satisfaction and professional identity, analyzing the correlations between these factors. The study hypothesized that professional identity and professional satisfaction would positively influence psychological capital after adjustment for demographic characteristics, and that psychological capital would differ by these demographic characteristics. Findings indicated that professional identity serves as a significant positive predictor of medical undergraduates’ psychological capital, whereas professional satisfaction exhibits no significant influence. Also, psychological capital differed by academic year, place of origin, and student-leadership status.

## Data Availability

The original contributions presented in the study are included in the article/Supplementary material, further inquiries can be directed to the corresponding authors.

## Supplementary material

Supplementary Table S1 Bonferroni-adjusted pairwise comparisons of psychological capital and its dimensions by academic year.
