# Supplementary material for: A study on the current status and influencing factors of psychological capital among medical undergraduates
Source: Front Psychol. 2026 Oct 30;17:1830730. doi: 10.3389/fpsyg.2026.1830730 (PMC13759799; doi:10.3389/fpsyg.2026.1830730)
Supplement: Supplementary file 1 [file Data_Sheet_1.ZIP › References PDF/A Study on the Impact of the ‘Three-Dimensional Full-Chain’ Collaborative Intervention Model on the Mental Health of University Students Undertaking Clinical Placements in Hospitals.pdf]

“三维全链条”协同干预模式对医院实习大学生心理健康影响研究

高利娟[1]，许飞飞[2]，刘 楠[2]，张 转[2]

（1.邢台医学院，河北 邢台 054000；2.邢台医学院第二附属医院，河北 邢台 054000）

摘 要：探讨“高校-医院-社会”三维全链条协同干预模式对医院实习大学生心理健康的主要作用。第一阶段对实习生进行横断面调查，评估其自我效能感、心理弹性、焦虑及抑郁情况现状。第二阶段对实习生进行研究，对干预组实施为期 6 个月的三维全链条协同干预。三维全链条协同干预后，干预组的自我效能感、心理弹性得分显著提升，焦虑、抑郁得分显著下降。运用“三维全链条”协同干预模式能有效提升实习生的积极心理资本，显著降低其焦虑与抑郁水平，减轻学生心理压力，提高大学生的心理健康水平。

关键词：医院实习生；医学生；高校-医院-社会；协同干预

中图分类号：G640 **文献标识码**：A **文章编号**：2097—6232（2026）01—0072—05

引言

医院实习是医学教育的关键转型阶段，医学实习生面临着角色冲突、高强度工作负荷、复杂医患关系、职业暴露风险、学业与就业等多重压力，易导致焦虑、抑郁、职业倦怠等心理问题，研究显示，约40%—60%的实习医学生存在不同程度的焦虑、抑郁或职业倦怠[1]，这不仅损害其个人身心健康与专业认同，也可能影响临床实践质量与患者安全。实习期心理问题源于个体、教育与环境的多重交互作用。国外研究强调制度性支持的重要性，如合理工时、心理督导、同伴支持计划等[2]。国内研究则更多关注带教关系、职业认同与社会期望的影响[3]，但对跨系统协作机制的探讨仍显不足。现有干预措施往往呈现“片段化”特点：高校干预在实习期间“鞭长莫及”，医院干预侧重职业压力而忽略学业与个人发展衔接，社会支持系统普遍缺位或未被有效整合。实习生的心理健康支持在高校与医院之间存在“管理真空”，在实习期结束后缺乏有效的“追踪与转化”，未能形成一个贯穿实习全过程的闭环支持系统。这就导致实习生在关键过渡阶段缺乏连续、系统的心理支持[4]。因此，构建贯穿实习全周期、多主体协同的“三维全链条”模式，具有重要的理论与实践意义。研究提出“三维全链条”协同干预模式：“三维”指高校、医院、社会（含家庭、行业协会、社区资源）三方责任主体协同发力；“全链条”指干预措施贯穿实习准备期（前）、实习适应与发展期（中）、实习总结与生涯转换期（后）三个阶段，形成预防、干预、追踪一体化的连续服务链。研究旨在通过随机对照试验，验证该模式对改善医院实习大学生心理健康、提升社会支持、缓解实习压力的有效性，以期为构建系统化、制度化的医学生实习期心理健康支持体系提供实证依据。

一、对象与方法

（一）研究对象

1.横断面调查阶段

因基层医疗机构实习生人数较少且专业不全，故选取邢台市实习大学生人数最多专业最全的三所三

收稿日期：2025—10—04

基金项目：2025 年度邢台市社会科学发展研究课题——“增进医院实习大学生心理健康策略研究”，项目编号：XTSKFZ2025010

作者简介：高利娟（1981—），女，河北平乡人，邢台医学院，副教授。

中国知网 https://www.cnki.net

级医院2025年2月—2025年5月期间的医学相关专业实习大学生为研究样本，涵盖临床医学、护理学、中医、针推、康复、药学、口腔医学、影像学8个专业，实习生分布在内科、外科、妇产科、儿科、中医及各医技科室，包含医院全部科室的实习生。纳入标准：全日制医学相关专业大学生，正在医院进行临床实习，入医院实习时长≥1个月，自愿参与研究，具备基本的沟通和理解能力；排除标准：短期见习生、有严重精神疾病史或正在接受精神科治疗的学生、因身体或其他原因无法完成问卷填写和访谈的学生。

采用整群抽样法，从各专业、各科室实习生中选取571名实习大学生进行问卷调查。其中实习早期（实习时长1—3个月）285名，实习晚期（实习时长8—10个月）286名；男生123名（21.5%），女生448名（78.5%）；独生子女140人（24.5%），非独生子女431人（75.5%）；来自单亲家庭59人（10.3%），来自非单亲家庭512人（89.7%）；处于恋爱中130人（22.7%），处于非恋爱中441人（77.3%）；自觉家庭经济状况为较差21人（3.7%）、中等497人（87%）、较好53人（9.3%）。

3. 研究阶段

从拟来我院实习的医学大学生中，在各个专业招募280名作为研究对象。采用随机数字表法将研究对象分为干预组与对照组，每组140人。两组在年龄、性别、专业、家庭所在地等基线资料上差异无统计学意义（$P>0.05$）。

（二）研究工具

一般情况调查表：自行设计，包括性别、年龄、专业、生源地、家庭经济状况、是否为独生子女、是否处于恋爱中、实习科室、实习时长等。

一般自我效能感量表（General Self Efficacy Scale，GSES）[5]：共10个条目，采用1—4级评分，评估实习生的自我认知与应对信心；GSES评分：涉及个体遇到困难或挫折时的自信心，以1—4级评分法评价，1分为完全不正确，2分为有点正确，3分为多数正确，4分为完全正确，总分是40分，0—10分是自我效能感最弱，10—20分是自我效能感弱，20—30分是自我效能感强，30—40分是自我效能感最强。分值越高，自我效能感越强。

Connor-Davidson心理弹性量表简化版[6]：采用0分（很少）—4分（几乎总是）5级评分，总分范围为0—40分，得分越高说明个体的心理弹性水平越高。评估实习生的抗压与心理恢复能力。

患者健康问卷（Patient Health Questionnaire-9，PHQ-9）[7]：对大学生的抑郁症状进行评估。该量表包含9个条目，如“做事时提不起劲或没有兴趣”“入睡困难、睡不安或睡得过多”“对事物专注有困难，例如看电视或做作业时”等，每个条目均采用Likert 4级评分（0=完全不会、1=有几天、2=超过一周、3=几乎每天）。总分为0—27，总分越高表示抑郁症状越严重，其中≥10分表示存在有临床意义的抑郁症状[7]。目前该量表已广泛运用至中国大学生人群的研究[8]。

广泛性焦虑障碍量表（Generalized Anxiety Disorder-7，GAD-7）[9]：对大学生的焦虑症状进行评估。该量表包含7个条目，如“感到不安、担心及烦躁”“对各种各样的事情担忧过多”“感到似乎将有可怕的事情发生而害怕”等，每个条目均采用Likert4级评分（0=完全不会、1=有几天、2=超过一周、3=几乎每天）。总分为0—21，总分越高表示焦虑症状越严重，其中≥10分表示存在有临床意义的焦虑症状[9]。目前该量表已广泛运用至中国大学生人群的研究[10]。

中文版知觉压力量表（CPSS）[11]：由14个反应压力的失控感和紧张感条目组成，总分为0—56分，0—28分为正常范围，29—42分为压力较大，43—56分为压力过大，分值越高提示知觉压力越大。

实习满意度问卷：自编问卷，包括带教支持、工作安排、心理环境等。采用SPSS26.0对实习满意度问卷的Cronbach's α系数进行分析，Cronbach's α系数0.78，内部一致性较高，符合研究要求。

（三）方法

1.横断面调查

采用统一的线上问卷平台，由经过培训的调查员组织集体施测，共发放571份，回收有效问卷542份，有效回收率 95.0%。

中国知网 https://www.cnki.net

2.“三维全链条”协同干预模式（仅对干预组实施，为期 6 个月）

维度一：高校心理支持（前端赋能与全程跟踪）。①实习前强化训练：开设“实习心理适应”必修模块（8 学时），内容涵盖压力管理、医患沟通、职业价值观塑造。②实习期跟踪辅导：实施“心理辅导员-实习组长”双轨跟踪制度，每月进行一次团体心理辅导或线上督导。

维度二：医院带教管理（过程优化与专业支持）。①支持性带教方案：对带教老师进行专项培训，推行“三明治反馈法”（肯定-建议-鼓励），每周至少一次结构化反馈。②环境与资源支持：设立“实习心理驿站”，提供个别咨询、放松训练；优化排班制度，保障连续夜班不超过 3 次。③同伴支持小组：每月组织以科室为单位的同伴支持活动，促进经验分享与情感联结。

维度三：社会资源对接（环境营造与正向激励）。①职业认同激励：定期报道优秀实习生事迹，颁发“月度实践之星”荣誉。②资源链接：与邢台市心理学会合作，提供免费心理热线与专题讲座，并为有需要的学生对接职业规划咨询服务。

对照组接受常规实习安排与带教，不参与上述系统性干预活动。

3.观察指标

于干预前（T1）、干预 6 个月结束时（T2）对两组研究对象心理健康指标（GAD-7、PHQ-9、CPSS）、心理韧性指标（CD-RISC-10）、自我效能感（GSES）进行测评。

4.统计方法

采用 SPSS 26.0 软件进行数据分析。计量资料以$\bar{x}\pm s$表示，组间比较采用独立样本 t 检验，组内前后比较采用配对样本 t 检验；计数资料采用 χ^2检验。以 $P<0.05$ 为差异有统计学意义。

二、结果

（一）横断面调查结果（n=542）

实习生的自我效能感得分为（25.6±5.8）分，心理弹性得分为（26.4±7.2）分。Pearson 相关分析显示，GSES、CD-RISC-10 得分均与 PHQ-9 标准分（r=-0.412，-0.498，P<0.01）和 GAD-7 标准分（r=-0.385，-0.467，P<0.01）呈显著负相关。此结果显示提升积极心理资本的紧迫性，并为干预方案的构建提供了直接依据。

（二）心理健康指标变化

干预前，对照组和干预组的学生的 GAD-7、PHQ-9、CPSS 评分，经比较差异无统计学意义（P>0.05）。

干预后，时间与组别在焦虑、抑郁、压力得分上存在显著交互作用（P＜0.01）。干预组在 T2 时点的得分显著低于对照组（表 1）。

表 1 两组心理健康得分比较（均值±标准差）

指标	组别	T1	T2
GAD-7	干预组	8.51±4.30	5.22±3.15**
	对照组	8.49±4.33	7.88±4.02
PHQ-9	干预组	9.20±4.90	6.05±3.42**
	对照组	9.15±4.86	8.76±4.55
CPSS	干预组	25.40±6.70	18.35±5.22**
	对照组	25.45±6.74	24.20±6.30

注：**P<0.01

（三）心理韧性、自我效能感与满意度比较

干预前，对照组和干预组的学生的 GSES、CD-RISC-10、满意度评分，经比较差异无统计学意义（P>0.05）。

干预 6 个月后（T2），干预组各项指标较干预前及同期对照组均发生显著积极变化（表 2）。积极心理资本提升：干预组的 GSES 得分从 25.4 分显著提升至 29.8 分，CD-RISC-10 得分从 26.2 分显著提升至 31.6 分，实习生整体满意度从 90.42%上升到 95.28%，组内及组间比较差异均具有高度统计学意义（P<0.01）。

中国知网 https://www.cnki.net

表 2 两组实习生干预后（T2）心理韧性、自我效能感与满意度得分比较及组内变化（x̄±s）

组别	时间点	GSES	CD-RISC-10	满意度
干预组	T1	25.4±5.7	26.2±6.9	90.42±0.69
	T2	29.8±4.9	31.6±6.1	95.28±0.72
	t 值(T2vsT1)	7.85**	8.12**	5.18**
对照组	T1	25.8±6.0	26.6±7.3	90.51±0.70
	T2	25.5±5.8	25.9±7.5	90.29±0.66
	t 值(T2vsT1)	0.47	0.92	-1.21
组间比较 t 值 (T2 时刻)		5.67**	6.01**	-7.25**

注：**P<0.01

三、讨论

（一）邢台地区医学实习生心理健康水平不高

本次横断面调查揭示了当前邢台地区医院实习大学生积极心理资本（自我效能感和心理弹性）储备不足，而负性情绪（焦虑和抑郁）问题较为普遍且自我效能感和心理弹性与焦虑、抑郁情绪存在显著的负向关联，这与已往研究一致[12-14]，自我效能感高的实习生，对自身处理临床任务、应对挑战更有信心，能更积极地看待困难，从而减少无助感和焦虑感。而心理弹性强的实习生，则如同拥有了“心理免疫系统”，能够更好地从挫折、批评和高强度压力中恢复过来，避免负面情绪的长期累积，表现出更强的适应能力。因此，提升这两项积极心理品质，是干预实习生心理健康问题的核心靶点。

（二）“三维全链条”协同干预模式的有效性分析

本研究结果表明，与常规管理相比，实施“三维全链条”协同干预模式能显著改善实习大学生的心理健康状况，表现为焦虑、抑郁水平显著降低，社会支持感显著提升，主观压力感得到更好控制。重复测量方差分析的显著交互效应证实了干预的效果并非自然随时间变化，而是模式本身的作用。这一结果符合压力-评价-应对理论，即通过系统性地优化和联结实习生所处的微、中、外系统，能够创造更有利的发展环境[15]。

协同干预模式的有效性可能源于以下几个机制。第一，打破了支持壁垒，高校与医院的正向协同机制确保了支持责任的连续传递，填补了管理真空。第二，实现了资源整合与精准供给，社会维度（家庭、行业）的引入，拓展了支持来源，使情感支持、榜样示范、职业导航等多元化需求得到更充分满足。第三，全链条设计有效预防了实习生的过度心理应激，实习前预防性教育提升了心理资本与应对资源，实习中持续干预帮助及时处理压力事件，实习后总结与转化促进了创伤后成长与身份整合，形成了完整的压力管理闭环。

（三）对抑郁、焦虑因子改善的启示

干预组在抑郁、焦虑因子上的显著改善提示三维全链条干预可能通过以下路径发挥作用：针对抑郁与焦虑，医院内的支持性氛围建设和高校的团体督导，直接提供了安全的人际练习场，降低了人际威胁感，全链条的支持网络通过提供问题解决资源（如带教老师指导）、减少孤独感（朋辈与家庭互动）、提升对未来可控感（生涯规划指导），直接针对了抑郁焦虑的核心认知（无望感、无助感）。

（四）实践意义与推广建议

研究验证的模式为院校和医院提供了可操作的蓝图。制度层面，高校与教学医院应签订包含心理健康支持条款的实习协议，明确双方权责；资源层面，设立专项经费，培训医院内部的“心理关怀导师”，开发标准化培训模块；技术层面，可开发专属线上平台，集成资源推送、同伴支持、预约咨询、危机预警等功能；文化层面，在医院和高校共同倡导关注医学生心理健康的行业文化，减少“坚强沉默”的刻板印象压力。

（五）研究局限与展望

研究优势在于其系统性、主动性与前瞻性，它改变了以往被动应对心理危机的模式，转而从教育前端、实践过程和支持环境进行全链条布局，将心理健康促进融入医学人才培养的整体框架。但也存在一定局限：

中国知网 https://www.cnki.net

第一，样本来自同一所医学院及来自邢台市区的三家三级医院，未来需在不同地区、不同院校、不同级别的实习生中进行多中心验证；第二，干预周期为 6 个月，对更长期的效果及结束后的持续影响需进一步追踪；第三，评价主要依赖自评量表，未来可结合他评（如带教老师评估）、生理指标（如皮质醇）进行多方法评估；第四，模式中各具三个维度干预措施的相对贡献度有待通过析因设计进一步厘清。

四、结论

研究构建并实证了“高校-医院-社会”三维全链条协同干预模式能有效整合分散的支持资源，形成了连续、系统的支持网络，显著提升了医院实习大学生的社会支持水平，缓解其焦虑、抑郁情绪，促进心理健康。研究结果支持从生态系统视角出发，通过制度化、结构化的跨系统合作来应对医学生实习期的心理挑战，为医学教育改革和健康人才培养提供了重要的实践路径。

参考文献：

[1]杨烨，张颖颖，周栩，等.医学生心理素质调研与评估[J].心理月刊，2022，17（10）：56-59+188.
[2]CHEN C,CHEN Y,SONG Y.Reciprocal relationship between interpersonal communication and depressive symptoms and the mediating role of resilience across two years:Three-wave cross-lagged panel model[J].Journal of affective disorders,2023(334):358-369.
[3]王彦华，姚春霞.医患关系现状及基于 EAP 视角的改进对策研究[J].河北工程大学学报（社会科学版），2020，37（03）：30-35.
[4]李敏，汤雅婷，吴志雄，等.2015—2018 年某医学高专新生心理健康状况调查[J].中国校医，2021，35（01）：1-4.
[5]胡象岭，田春凤，孙方尽.中文版一般自我效能量表的信度和效度检验[J].心理学探新，2014，34（01）：53-56.
[6]WANG L, SHI Z, ZHANG Y, et al. Psychometric properties of the 10-item Connor-Davidson Resilience Scale in Chinese earthquake victims[J]. Psychiatry and Clinical Neurosciences, 2010, 64(05): 499-504.
[7]KROENKE K, SPITZER R L, WILLIAMS J B. The PHQ-9: validity of a brief depression severity measure[J]. Journal of General Internal Medicine, 2001, 16(09): 606-613.
[8]皮陆杨，张桂莲，何坤镁，等.自我同情对大学生情绪问题的纵向影响：基本心理需要满足的双重作用模型[J].中国健康心理学杂志，2024，32（01）：149-155.
[9]SPITZER R L, KROENKE K, WILLIAMS J B, et al. A brief measure for assessing generalized anxiety disorder: the GAD-7[J]. Archives of Internal Medicine, 2006, 166(10): 1092-1097.
[10]黄郁，张洛奕，李娜，等.社交媒体成瘾与大学生焦虑的关系:睡眠拖延的中介作用和压力知觉的调节作用[J].现代预防医学，2024，51（20）：3756-3761.
[11]徐霞，王亚铃，徐铭，等.军医大学护士长心理压力与情感衰竭、生活满意度及情绪调节方式的关系[J].第三军医大学学报，2020，42（24）：2433-2438.
[12]周燕莉，卢建兰，徐瑶，等.护理实习生压力源、自我效能及应对方式的现状调查研究[J].中国当代医药，2025，32（03）：30-34.
[13]李平东，盛青青，黄瑶，等.“00 后”护生临床实习环境与临床实习适应不良的相关性研究[J]. 广州医科大学学报，2022，50（04）：95-98.
[14]张越，刘海迎，郭雪琪，等.临床实习适应在护理本科生角色压力与心理健康间的中介效应分析[J].中华护理教育，2025，22（06）：699-704.
[15]LAZARUS R S, DELONGIS A, FOLKMAN S, et al. Stress and adaptational outcomes: the problem of confounded measures[J]. American Psychologist, 1985, 40(07): 770-779.

中国知网 https://www.cnki.net
