# Supplementary material for: A study on the current status and influencing factors of psychological capital among medical undergraduates
Source: Front Psychol. 2026 Oct 30;17:1830730. doi: 10.3389/fpsyg.2026.1830730 (PMC13759799; doi:10.3389/fpsyg.2026.1830730)
Supplement: Supplementary file 1 [file Data_Sheet_1.ZIP › References PDF/A Study on the Influencing Factors of College Students' Career Planning-Based on the Survey Data of 9 Universities in the Yangtze River Delta.pdf]

中国青年社会科学
2020 年第 4 期

青少年与社会发展
第 39 卷(总第 209 期)

大学生职业生涯规划影响因素研究
——基于长三角 9 所高校的调研数据

■ 孟祥敏
(浙江万里学院 商学院,浙江 宁波 315100)

【摘要】职业生涯规划与大学生就业和职业生涯发展有着密切的关系。根据长江三角洲 9 所高校 1 242 名大学生职业生涯规划状况的调查数据,采用交叉表和 logit 模型分析发现,性别、年级、是否学生干部、职业发展认知、职业规划认知对大学生形成明确的职业生涯规划有着重要影响。为了提高大学生职业生涯规划的可行性和有效性,高校应加强对大学生的职业生涯规划意识教育,建立健全职业生涯规划管理和服务体系,最大限度地提升大学生的就业竞争力。

【关键词】大学生 职业生涯规划 影响因素 职业教育
DOI:10.16034/j.cnki.10-1318/c.2020.04.015

一、问题的提出

自 1999 年中国高校扩招以来,高校毕业生数量持续逐年攀升。据教育部公布的数据,2002 年高校毕业生 145 万,2016 年毕业生人数增长到 765 万,2017 年达 795 万,2018 达 820 万,2019 达 834 万①,2020 年达 874 万②,18 年增长了 5 倍。毕业生数量持续激增必然导致待业大学生人数不断增加。据统计,本科毕业生待业人数 2004 年为 69 万人③,2020 年预计达到两百多万。今年受疫情影响,就业形势不佳,大学生就业问题更加突出,引起各级政府和高校高度重视。2020 年 2 月 25 日,国务院常务会议就大学生就业问题出台了专升本、研究生扩招、鼓励入伍参军、鼓励自主就业等一系列有针对性的措施,教育部还推出了“24365 全天候网上校园招聘”等服务。

择业和就业过程是劳动市场上需求和供给的匹配过程,劳动供给和需求的匹配成功率的高低在很大程度上决定了就业率或失业率的高低。劳动供求匹配成功率决定于多种因素,其中的一个重要因素就是劳动供给者的技能、素质和就业愿望与劳动需求的吻合程度。在校大学生作为未来的或潜在的劳动力供给者,技能和素质固然很重要,但是在校期间做好职业生涯规划也同样重要。大学生是高学历高素质的劳动者,他们择业就业不仅仅是要找一份工作,更是为了

收稿日期:2020-06-01
作者简介:孟祥敏,浙江万里学院商学院讲师,南京航空航天大学经济与管理学院博士研究生,主要研究大学生职业生涯教育。

① 参见《中国历年高校毕业生人数一览表》,https://www.mrcjcn.com/n/z94584.html.
② 参见李 澈 等《将小我融入大我,以青春贡献祖国》,载《中国教育报》,2020 年 7 月 10 日。
③ 参见《本科毕业生待业率 30% “新失业现象”来势凶猛》,https//yjbgs.com/jinyezhidao/zhiyeguihual/47955.html.2017-08-13.

(C)1994-2022 China Academic Journal Electronic Publishing House. All rights reserved. http://www.cnki.net

实现自己的人生价值。大学生通过职业生涯规划明确自己的发展方向和人生理想，可以有选择有重点地学习相应的专业知识和技能，从而提高自身竞争力，为实现自己的人生理想和人生价值最大化开好局、起好步。正是由于这个缘故，各类高校和在校大学生近些年来都非常重视并积极推进大学生职业生涯规划，高校纷纷开设《职业生涯规划》《就业指导》等课程，举办职业生涯规划大赛等活动。但是我们也发现，在校大学生形成明确职业生涯规划的比例仍然不高。

大学生职业生涯规划不是简单地人为制定出来的，它受到各种因素的影响和制约。因此，要使大学生能够结合自身特点、潜质和理想做好职业生涯规划，理清大学生职业生涯规划的影响因素就显得必要和重要。

本文以长三角地区的高校为样本，在实证调查的基础上，对大学生职业生涯规划的影响因素进行分析，重点探讨大学生职业生涯规划的意愿、行为及影响因素，揭示大学生职业生涯规划的特点、问题根源，为大学生职业生涯教育提供依据，有针对性地帮助大学生做好职业生涯规划，提高大学生的职业生涯规划意识和能力，为他们毕业时提高就业竞争力和就业率创造条件。

二、文献综述与研究设计

（一）文献综述

职业生涯规划的好坏，在很大程度上影响一个人的未来发展和事业成功与否，因此国内外不少学者都对在校大学生的职业规划状况及其影响因素作过许多研究并形成了职业生涯规划理论。职业生涯规划理论认为，职业生涯规划与个人就业和职业生涯发展有着密切的关系[1]。Crites[2]，Achebe[3]和 Lokan[4]研究指出，不同年级的学生在生涯感受、生涯探索、生涯计划与生涯行动上有显著差异，且随年级的增加而增加。Orpen 研究发现，系统的职业生涯规划有助于获得成功的职业生涯[5]。Flum & Blustein 认为，大学生有无明确的职业生涯规划将影响其就业和职业发展[6]。影响大学生开展职业生涯规划的因素更受到国内外学者的广泛关注。Bakar & McCracken 研究发现，相同专业不同年级的青少年生涯发展无显著性差异，说明年龄不是影响大学生职业生涯规划的主要因素[7]。甘寿国、蔡涛研究发现，与女生相比，男生对待职业生涯的态度更自信、更积极，说明性别在大学生开展职业生涯规划方面存在显著性差异[8]；陈芬、毕佳萍研究发现，学习的动机与学习生活适应性、职业决策能力、职业规划策略的应用对大学生职业生涯规划有显著正相关影响[9]。季学军、吴朝琴研究指出，90 后大学生认识到职业生涯规划重要，但缺乏对职业生涯规划的了解，因而也就缺乏具体的职业生涯规划行动[10]。张玉等认为个人因素对职业生涯规划的影响较大，其中随年纪的增长而不同[11]。姜明伦等研究认为，在校大学生认可职业生涯规划重要但是缺乏相关的职业生涯规划知识，近一半的学生没有明确的职业生涯规划，影响大学生有无明显职业生涯规划的主要因素有年级、身份、学科、专业选择依据、专业满意度、职业规划认知等[12]。

（二）研究设计

如上所述，国内外学者在大学生职业生涯规划研究方面取得了一些成果，但是在“哪些因素影响了大学生职业生涯规划”的问题上还存在诸多争议。国外的研究成果大多基于发达国家的情形，这些研究成果并不适合国内现状；国内学界在这方面的研究成果大多是基于理论分析或案例分析，通过问卷调查获取数据再进行计量分析的实证研究不多。本文是基于中国经济和教育最发达的长三角地区的代表性高校进行大学生群体的调查，再根据调查获取的一手资料和数据进行实证研究，这种“接地气”的研究有助于了解大学生群体职业生涯规划的现状、特点和影响因素，为提出切实可行的、针对性较强的对策建议奠定基础。

(C)1994-2022 China Academic Journal Electronic Publishing House. All rights reserved. http://www.cnki.net

借鉴已有的研究成果和基于作者多年从事在校大学生学生工作积累的经验以及对大学生职业生涯规划现状的思考，本文对大学生职业生涯规划的影响因素，对问卷调查的变量指标(假设)作出了如下设计。

性别。一般而言，对待职业生涯的态度，男生比女生更自信、积极，女性比男性更畏惧风险，渴求稳定，故提出研究假设 H1：男生相比女生更倾向于制定职业生涯规划。

年级。随着年级升高、专业课程的深入学习和实践活动的锻炼，学生的能力得到有效培养和提升，故提出研究假设 H2：年级越高越有可能制定职业生涯规划。

是否学生干部。由于学生干部有更多机会参与、组织校园的集体活动，与社会打交道，能够锻炼各种能力，获得更多信息，故提出研究假设 H3：学生干部比普通学生更可能制定职业生涯规划。

学科。一贯接受系统与计划思维训练的理工类大学生制定计划的能力明显高于文科类大学生，故提出研究假设 H4：理工科大学生比文科生更可能制定职业生涯规划。

选择专业依据。在高考填报志愿时，结合自身情况自主选择专业的学生，往往比根据外部意见填报志愿的学生更加积极主动地关注自身职业能力的提升，更会主动思考和规划职业生涯，故提出假设 H5："自我选择专业"的大学生比"受外部意见选择专业"的大学生更可能制定职业生涯规划。

对专业的满意度。一般而言，对所学专业满意的大学生相比于对专业不满意的大学生，往往更加关注专业发展前景和未来职业生涯发展，故提出假设 H6：对专业满意度高的大学生比对专业不满意的大学生更可能制定职业生涯规划。

职业发展认知。对职业发展方向了解的大学生，往往会根据职业发展目标制定相应的计划，故提出假设 H7：对职业发展方向了解的大学生往往更倾向于制定职业生涯规划。

职业知识来源。一般而言，高校开设的职业生涯规划课程和职业测评等课程比较系统和专业，而通过网络、书籍报刊等获得职业生涯规划知识往往缺乏系统性和专业性，故提出假设 H8：职业生涯规划知识来源于学校的比来源于其他途径的大学生更可能制定职业生涯规划。

根据上述设计，本课题组于 2019 年 11－12 月对浙江大学、浙江工商大学、浙江树人大学、江南大学、南京工程学院、南京信息工程大学滨江学院、上海交通大学、东华大学、上海工会管理职业学院 9 所高校进行了问卷调查和访谈。调查抽样采用多阶段分层抽样法。本次共发放调查问卷 1 500 份，实际回收 1 426 份，有效问卷为 1 294 份，有效率为 90.7%。

本文被调查样本高校具有较强的代表性：从学校性质上看，分为公办高校(7 所)和民办高校(2 所)；从学科构成上看，分为综合性大学(5 所)、工科院校(2 所)和财经类院校(2 所)；从学历教育层次上看，分为本科院校(8 所)和专科院校(1 所)。此外，在调查样本高校中，985 高校 2 所，211 高校 4 所，其他高校 3 所。被调查大学生也具有代表性：从学历结构上看，以本科为主，占 68.1%；从性别上看，男生占比 58.5%，女生占比 41.5%；从年级构成上看，以大一、大二为主，二者合计达 61.4%；从学科结构上看，以理工类为主，占 55.0%，其次是文史类，占 38.5%，艺术类占 2.2%，其他学科专业占 4.3%，基本上反映了长三角地区高校大学生就读专业分布情况。

三、研究发现

(一) 变量描述性统计

本文选择的模型变量、变量定义以及样本数据按常规数理统计进行定义，例如年级：大一＝

(C)1994-2022 China Academic Journal Electronic Publishing House. All rights reserved.   http://www.cnki.net

1，大二 =2，大三 =3，大四 =4 ，极小值为 1，极大值为 4，均值为 2.18，标准差为 1.085; 描述性统计结果见下页表 1。

表 1　模型变量说明及描述性统计

变量	变量定义	极小值	极大值	均值	标准差
	解释变量				
性别	男生 =1，女生 =0	0	1	0.41	0.493
年级	大一 =1，大二 =2，大三 =3，大四 =4	1	4	2.18	1.085
干部	学生干部 =1，非学生干部 =0	0	1	0.37	0.483
学科	理工科 =1，文科及其他 =0	0	1	0.55	0.497
专业选择根据	适合自己 =1，外部意见 =0	0	1	0.39	0.488
专业满意度	对专业满意 =1，不满意 =0	0	1	0.51	0.501
职业发展认知	了解职业发展方向 =1，不了解 =0	0	1	0.51	0.501
职业知识来源	职业指导知识来源学校 =1，其他 =0	0	1	0.29	0.456
职业规划认知	了解职业规划知识 =1，不了解 =0	0	1	0.32	0.466
	被解释变量				
职业规划制定	进行了职业生涯规划 =1，未进行 =0	0	1	0.67	0.469

注: 大一、大二、大三样本含专一、专二、专三，专科三年级和本科四年级都视同毕业年级。下同。

（二）大学生职业生涯规划影响因素的交叉分析

为了筛选可能对大学生制定职业生涯规划产生影响的因素，本文对变量进行交叉分析，输出结果如下页表 2 所示。具体分析如下:

性别: 性别不同的大学生职业生涯规划不同，女生中具有职业生涯规划的比例（71.6%）高于男生（64.5%），且差异显著（ $P<0.01$ ）。说明，性别是影响大学生职业生涯规划的重要因素，研究假设 H1 不成立。

年级: 其交叉表的卡方值在 1% 的水平上显著，这说明，年级是影响大学生职业生涯规划的重要因素。其中职业生涯规划比例最高的为大三学生，高达 73.7%，然后依次是大二和大四年级，大一学生具有生涯规划的比例最低，为 59.2%，可见研究假设 H2 成立。

学生干部: 其交叉表的卡方值在 1% 的水平上显著，这说明，是否学生干部也是影响大学生职业生涯规划的重要因素。其中学生干部具有生涯规划的比例为 85.8%，高于非学生干部的比例，研究假设 H3 成立。

学科: 其交叉表的卡方值在 10% 的水平上显著。从数据来看，理工科生（69.6%）比文科生（64.9%）更倾向于制定职业生涯规划，研究假设 H4 成立。

专业选择依据: 其交叉表的卡方值在 1% 的水平上显著。根据“适合自己”的原则选择专业的大学生，其进行职业生涯规划的比例为 80.0%，远高于“听从外部意见”的大学生。这说明，根据兴趣爱好选择专业的大学生相比于听取外部意见选择专业的大学生而言更倾向于制定职业生涯规划，研究假设 H5 成立。

专业满意度: 其交叉表的卡方值在 1% 的水平上显著。对专业满意的大学生中有 76.1% 制定了职业生涯规划，远高于对专业不满意的大学生。这说明，对专业满意的大学生相比于对专业不满意的大学生更倾向于制定职业生涯规划，研究假设 H6 成立。

职业知识来源: 其交叉表的卡方值在 1% 的水平上显著。这说明，从学校获得职业生涯规

(C)1994-2022 China Academic Journal Electronic Publishing House. All rights reserved.　http://www.cnki.net

划知识的大学生比从其他途径获得职业生涯规划知识的大学生更倾向于制定职业生涯规划，研究假设 H7 成立。

表 2　大学生个体特征及职业生涯认知与大学生职业生涯规划的交叉分析

变量名称	变量分类	有无职业生涯规划(%)		合计	x2
		无	有		
性别	女生	28.4	71.6	100.0	7.242***
	男生	35.5	64.5	100.0	
	合计	32.6	67.4	100.0	
年级	大一	40.8	59.2	100.0	20.538***
	大二	29.2	70.8	100.0	
	大三	26.3	73.7	100.0	
	大四	29.9	70.1	100.0	
	合计	32.8	67.2	100.0	
干部	非学生干部	43.5	56.5	100.0	118.462***
	学生干部	14.2	85.8	100.0	
	合计	32.6	67.4	100.0	
学科	文科	35.4	64.6	100.0	3.672*
	理工科	30.4	69.6	100.0	
	合计	32.6	67.4	100.0	
专业选择依据	外部意见	37.0	63.0	100.0	36.497***
	适合自己	20.0	80.0	100.0	
	合计	31.1	68.9	100.0	
专业满意度	对专业不满意	41.6	58.4	100.0	46.010***
	对专业满意	23.9	76.1	100.0	
	合计	32.6	67.4	100.0	
职业发展认知	不了解职业发展方向	40.2	59.8	100.0	74.071***
	了解职业发展方向	16.1	83.9	100.0	
	合计	32.5	67.5	100.0	
职业知识来源	非学校	35.0	65.0	100.0	8.375***
	学校	26.7	73.3	100.0	
	合计	32.6	67.4	100.0	
职业规划认知	不了解职业规划知识	48.7	51.3	100.0	149.852
	了解职业规划知识	16.8	83.2	100.0	
	合计	32.6	67.4	100.0	

注: ***和* 分别表示在 1% 和 10% 的水平下显著。

职业发展认知: 其交叉表的卡方值在 1% 的水平上显著。结果表明: 了解职业生涯规划知

(C)1994-2022 China Academic Journal Electronic Publishing House. All rights reserved. http://www.cnki.net

识的大学生中有 83.2% 制定了职业生涯规划，高于不了解职业生涯规划知识的大学生。这说明，了解职业生涯规划知识的大学生比不了解职业生涯规划知识的大学生更倾向于制定职业生涯规划，研究假设 H8 成立。

（三）大学生职业生涯规划影响因素 Logit 模型分析

本文模型的被解释变量为"是否进行了职业生涯规划"，两分类变量：1 为是，0 为否。本文采用 Logit 模型进一步分析通过显著性检验的各个变量内部各类别对大学生职业生涯规划的影响。解释变量是影响大学生制定职业生涯规划的个体特点、职业生涯规划认知等因素，具体包括性别、年级、干部、学科、专业选择依据、专业满意度、职业发展方向认知，职业知识来源、职业规划认知，均为虚拟变量。设大学生 i 进行职业生涯规划的概率为 P_i，则 Logit 模型的具体形式为：

$$\mathrm{P_i} = F(\alpha + \sum_{j=1}^{m}\beta_j X_{ij} + u) = 1/\{1 + exp[-(\alpha + \sum_{j=1}^{m}\beta_j X_{ij} + u)]\}$$

其中，P_i 是第 i 个大学生制定职业生涯规划的概率，i 为大学生编号；β_j 表示第 j 种影响因素的回归系数；m 表示影响因素的个数；X_{ij} 是解释变量，表示第 i 个大学生的第 j 种影响因素；α 为截距；u 为误差项。

根据调研数据，本文运用统计软件 SPSS18.0，采用 Logit 模型进行回归分析，结果见下页表 3。其中，模型Ⅰ分析性别对大学生形成明确职业生涯规划的影响，模型Ⅱ分析年级对大学生形成明确职业生涯规划的影响，模型Ⅲ分析学生干部对大学生形成明确职业生涯规划的影响，模型Ⅳ分析学科对大学生形成明确职业生涯规划的影响，模型Ⅴ分析专业选择依据对大学生形成明确职业生涯规划的影响，模型Ⅵ分析专业满意度对大学生形成明确职业生涯规划的影响，模型Ⅶ分析职业发展方向认知对大学生形成明确职业生涯规划的影响，模型Ⅷ分析职业知识来源对大学生形成明确职业生涯规划的影响，模型Ⅸ分析职业规划认知对大学生形成明确职业生涯规划的影响，模型Ⅹ分析上述 9 个因素中通过显著性检验的各个变量内部类别对大学生形成明确职业生涯规划的影响。

模型估计结果显示，各模型总体显著性水平不高，但是，系数的显著性水平足以说明大学生个体特点及职业认知等因素对其职业生涯规划形成的影响。具体分析如下。

从性别变量的影响来看，其系数在模型Ⅰ中通过 1% 的显著性水平检验，在模型Ⅹ中通过 1% 的显著性水平检验。这说明，性别是影响大学生形成明确的职业生涯规划的重要因素。

从年级变量的影响来看，大二及以上年级大学生更加具有明确的职业生涯规划，且年级越高系数越大。这说明，年级是影响大学生形成明确的职业生涯规划的重要因素。但是，大四在模型Ⅹ中没有通过显著性检验，这可能是因为加入其他变量后产生了交互影响所致。

从是否学生干部变量的影响来看，其系数在模型Ⅲ和模型Ⅹ中均通过 1% 的显著性水平检验。这说明，是否学生干部对大学生形成明确的职业生涯规划影响大。

从学科变量的影响来看，其系数在模型Ⅳ中通过 10% 的显著性水平检验，在模型Ⅹ中未通过显著性水平检验。这说明，学科不是影响大学生形成明确职业生涯规划的重要因素。

从专业选择依据变量的影响来看，其系数在模型Ⅴ和模型Ⅹ中均通过 1% 的显著性水平检验。这说明，专业选择依据变量是影响大学生形成明确的职业生涯规划的重要因素。

从专业满意度变量的影响来看，其系数在模型Ⅵ模型中通过 1% 的显著性水平检验，但在模型Ⅹ中未通过显著性水平检验。这主要是因为，专业选择依据和专业满意度具有交互影响，专业选择依据会直接影响专业满意度，根据自身兴趣、爱好选择专业的，其专业满意度也高，在单样本 T 检验中也证明了这一点。因此，在模型中纳入其中一个变量就可以。

(C)1994-2022 China Academic Journal Electronic Publishing House. All rights reserved. http://www.cnki.net

表 3　大学生职业生涯规划影响因素计量结果表

解释变量	模型Ⅰ	模型Ⅱ	模型Ⅲ	模型Ⅳ	模型Ⅴ	模型Ⅵ	模型Ⅶ	模型Ⅷ	模型Ⅸ	模型Ⅹ
性别(以女生为参照组)										0.485***
男生	0.322***									
年级(以大一为对照组)										
大二	0.514***									0.753***
大三	0.657***									0.888***
大四	0.479***									0.167
干部(以非干部为对照组)										
学生干部		1.542***								1.442***
学科(以文科对照组)										
理工科			0.228*							0.182
专业选择(以外部意见为对照组)										
适合自己				0.856***						0.587***
专业满意度(以不满意为对照组)										
专业满意					0.820***					0.205
职业发展认知(以不了解为对照组)										
了解职业发展方向							1.548***			1.228***
职业知识来源(以非学校为参照)										
学校职业指导中心								0.391***		0.616***
职业规划认知(以不了解为参照组)										
了解职业规划知识									1.255***	0.755***
截距	0.596	0.371	0.260	0.602	0.533	0.339	0.050	0.618	0.396	-1.558
-2Log likelihood	1626.236	1514.099	150.117	1625.780	1447.769	1583.057	1476.596	1614.830	1541.226	1099.754
Cox-Snell R2	0.005	0.017	0.094	0.003	0.031	0.035	0.113	0.007	0.060	0.229
Pseudo R2	0.008	0.023	0.132	0.004	0.044	0.049	0.157	0.009	0.084	0.321

注:* 表示在 10% 的水平下显著,**表示在 5% 的水平下显著,***表示在 1% 的水平下显著。

从职业发展认知变量的影响来看,其系数在模型Ⅷ和模型Ⅹ中均通过 1% 的显著性水平检验。这说明,职业发展认知是影响大学生形成明确的职业生涯规划的重要因素。

从职业知识来源变量的影响来看,在模型Ⅷ和模型Ⅹ中均未通过 1% 的显著性水平检验。这说明,职业生涯知识来源是影响大学生形成明确职业生涯规划的重要因素。

从职业生涯认知变量的影响来看,在模型Ⅷ和模型Ⅹ中均未通过 1% 的显著性水平检验。这说明,职业生涯认知变量是影响大学生形成明确职业生涯规划的重要因素。

四、研究结论及启示

对长三角 9 所高校 1 242 名大学生的调查数据表明,大学生形成明确职业生涯规划的比例比较高,希望通过此类方式更好地规划未来、实现梦想。其中,性别、年级、是否学生干部、专业选择依据(或对专业满意度)、职业发展方向认知、职业知识来源、职业生涯认知对大学生形成

(C)1994-2022 China Academic Journal Electronic Publishing House. All rights reserved. http://www.cnki.net

明确的职业生涯规划有直接影响。

从性别因素来看，女生比男生具有更加明确的职业生涯规划。性别差异加上受传统思想影响，男性在就业市场上的优势明显。从就业市场竞争来看，女性一般重视获得安全感和稳定性，所以就业选择范围客观上小于男性。而为了在激烈竞争的劳动力市场上获得与男性同等的发展机会，女性大学生在大学阶段就会重视和着手自身的职业生涯规划。

从年级因素来看，年级高的大学生比年级低的大学生具有更明确的职业生涯规划。经过不断的学习和积累，大学生的心理成熟度随之增加，对自己潜力和发展意向的认识更加清晰，随着年级的提升，就业选择的临近，加上学校职业教育的影响，他们更加重视职业生涯规划。

从是否学生干部因素来看，学生干部比普通学生更可能制定职业生涯规划。由于在学生群体中，学生干部在思想上更为积极进取，行动上也更为主动，并且有更多的机会参与组织和策划各类学生活动，他们的执行力和综合能力有更多的机会得以提升，这些机会和品质会促使他们为将来的职业生涯做好充分准备，因而表现出较强的职业生涯规划能力和行动力。

从专业是否自主选择因素来看，依据自身情况自主选择专业的大学生相比于外界帮助选择专业的大学生具有更明确的职业生涯规划。这说明具有较强独立性和自主意识的大学生，更为积极主动地开展职业生涯规划。

从职业发展方向认知因素来看，清楚职业发展方向的大学生比不了解职业发展方向的大学生具有更明确的职业生涯规划。这表明有明确的人生理想和目标的大学生更重视自己的职业生涯规划。

从职业知识来源因素来看，职业生涯知识来源于学校的比通过其他途径获得规划知识的具有更明确的职业生涯规划。这可能是由于学校的职业教育更系统更专业更有针对性，而其他途径获得的职业规划知识可能是碎片式的、针对性不高的。

从职业生涯规划认知因素来看，职业生涯规划知识丰富的大学生比这方面知识匮乏的大学生具有更明确的职业生涯规划。这提示我们给在校大学生传授职业生涯规划知识是必要的。

上述研究结果对各类高校加强在校大学生职业生涯规划提供了许多有益的启示。

一是重视并加强职业生涯规划意识教育，提高大学生对职业生涯规划重要性的认识。高校应积极营造良好的职业生涯规划氛围，将多样化的职业生涯教育形式融入到高校就业指导中，让大学生实实在在地接受职业生涯规划的熏陶，感受职业生涯规划对其就业选择和自身发展的重要意义。

二是构建完善的大学生职业生涯教育辅导体系。高校应努力构建全程式的大学生职业生涯规划辅导体系，不仅要有负责干部主抓，而且必须有约束性的规划与计划。笔者认为，应构建一个逐级上升的大学生职业生涯辅导阶梯，分阶段、有针对性地对大学生进行自我认知教育、专业的职业前景分析和阶段性职业生涯目标辅导(见下页图)。对于新入学的大一学生，他们内心充满憧憬，对未来职业发展抱着一种理想的态度，对此应着重培养学生的兴趣和爱好，为以后做好职业生涯规划打下良好的基础。针对大二学生，在职业生涯规划辅导内容选择上，应帮助学生做好自我职业测评和职业定位选择。大三是一个关键时期，应为大学生做好职业实践指导，辅助学生做好职业发展路径设定，根据职业发展目标适时地帮助学生调整方向。大四是职业需求和实现的阶段，面对理想和现实的落差，学校应关注对大学生的就业心理疏导、求职面试技巧传授，帮助学生及时地调整心态，增强其克服困难的信心和勇气。

三是加强专业认知教育。专业认知教育是培养学生专业兴趣的起点。学生对专业的正确认知是激发专业兴趣，形成正确的学业认知、职业意识、职业生涯规划以及自我认知的必要条件。因此，在大学生职业生涯规划教育过程中，应加强对大学生的专业认知教育，拓宽专业认知

(C)1994-2022 China Academic Journal Electronic Publishing House. All rights reserved.   http://www.cnki.net

			确定职业生涯选择	职业生涯教育内容
		职业实践指导	就业心理疏导教育	
	职业定位和选择	职业发展路径设定	面试技巧	
大学学习生活规划	职业测评	职业环境分析	求职指导	
职业生涯规划基础知识	职业生涯规划基础知识	职业生涯规划基础知识	职业生涯规划基础知识	
大一	大二	大三	大四	

大学生职业生涯规划辅导的上升阶梯图

途径。设立专业认知教育课程，进一步完善大学生的专业认知教育体系。将专业认知教育融入到大学生的职业生涯规划中来，开启动态专业认知新模式，满足大学生成才成功的强烈愿望。

四是建立健全大学生职业生涯规划管理和服务体系。正确理解实施职业生涯规划的复杂性和长期性，加强大学生职业生涯规划管理制度建设，构建一套完善的职业生涯管理和服务体系，其中最为重要是加强大学生职业指导师资队伍建设，努力提高就业指导队伍的专业化和职业化水平，尤其是辅导员的业务素质和职业指导水平；要能够根据社会需要和学生自身特点，指导大学生做好职业生涯规划，为每位学生建立一份个人特点、兴趣、职业能力倾向及家庭背景的个人资料档案，并以此进行动态的辅导管理，提高大学生的职业素养和职业发展能力。

[参 考 文 献]

[1] Gould, S. Characteristics of career planners in upwardly mobile occupations. Academy of Management Journal, 1979, (3).

[2] John. crites. Measurement of vocational maturity in adolescence: Attitude test of the vocational development inventory. Psychological Monographs, 1965, (2).

[3] Achebe, C. Assessing the vocational maturity of students in the east central state of Nigeria. Journal of Vocational Behavior, 1982, (2).

[4] Janice j. lokan, Marvin w. Boss, et al. A study of vocational matuity during adolescence and locus of control. Journal of Vocational Behavior, 1982, (3).

[5] Christopher Orpen. The effects of organizational and individual career management on career success. International Journal of Manpower, 1994, (1).

[6] Hanoch Flum, David L. Blustein. Reinvigorating the Study of Voeational Exploration: A Framework for Research, Journal of Vocational Behaviour 2000, (3).

[7] Bakar, Ab. Rahim, et al. Youth organization and supervised Agricultural experience participation as predictors of the career maturity of agricultural education students. 2006 - 4 - 3. https: //eric. ed. gov/? id = ED369917

[8] 甘寿国 蔡 涛《大学生职业生涯规划状况及其影响因素探究——来自广东金融学院的调查报告》，载《高教探索》，2007年第4期。

[9] 陈 芬 毕佳萍《师范类研究生职业生涯规划状况及影响因素探究——来自华东师范大学的调查报告》，载《中国大学生就业》，2008年第13期。

[10] 季学军 吴朝琴《"90"后大学生职业生涯规划意识现状调查与分析》，载《文教资料》，2011年第18期。

[11] 张 玉 班兰美 等《大学生职业生涯规划现状及其影响因素》，载《中国健康心理学杂志》，2013年第3期。

[12] 姜明伦 李战国 等《大学生职业生涯规划行为选择及影响——基于宁波市6所高校的调查分析》，载《高教探索》，2016年第2期。

(责任编辑：汤杏林)

(C)1994-2022 China Academic Journal Electronic Publishing House. All rights reserved. http://www.cnki.net
