# Supplementary material for: A study on the current status and influencing factors of psychological capital among medical undergraduates
Source: Front Psychol. 2026 Oct 30;17:1830730. doi: 10.3389/fpsyg.2026.1830730 (PMC13759799; doi:10.3389/fpsyg.2026.1830730)
Supplement: Supplementary file 1 [file Data_Sheet_1.ZIP › References PDF/A Study on the Mediating Role of a Sense of Meaning in Life between Medical Students’ Belief in a Just World and Their Positive Psychological Capital.pdf]

生命意义感在医学生公正世界信念与积极心理资本间中介作用的研究

郑亚楠[1，2]

【摘要】目的 了解医学生公正世界信念现况及公正世界信念与积极心理资本和生命意义感的关系，为医学院校教学改革提供参考。方法 于2023年5—6月采用公正世界信念量表、积极心理资本问卷和生命意义感量表对753名赣南医科大学医学生进行问卷调查。结果 女生公正世界信念量表评分为（53.32±9.53）分，高于男生的（50.70±10.55）分，差异有统计学意义（$P < 0.001$）；大一医学生公正世界信念量表评分为（53.59±9.43）分，高于大二医学生的（52.42±10.43）分、大三医学生的（51.30±9.98）分，差异有统计学意义（$P < 0.05$）。医学生公正世界信念与积极心理资本和生命意义感呈正相关（$P < 0.05$）。结论 生命意义感在医学生公正世界信念和积极心理资本之间起部分中介效应，积极心理资本可以直接对医学生公正世界信念产生正向预测作用，也可以通过生命意义感间接影响公正世界信念。医学院校应有针对性地开展相应的宣传教育工作。

【关键词】医学生；公正世界信念；积极心理资本；生命意义感；中介效应；人文精神
【中图分类号】G642 【文献标志码】A
【文章编号】1004-6763（2025）02-0090-04
doi: 10.3969/j.issn.1004-6763.2025.02.023

公正世界信念是个体对公正的信念、认可程度及对公正的渴望与心理需求，即个体相信自己的努力能够获得回报，可以通过努力得到自己应得的[1]。因大学生受就业压力、高校扩招等因素影响，这一群体的心理健康教育与提升策略研究已受到社会各界的关注[2-3]。而医学生在大学生群体中较为特殊，因学业压力更大、就业岗位对学历要求更高以及医患关系复杂等多方面因素的影响，医学生心理健康程度偏低[4]。而医学生是我国医疗卫生事业的后备人才，提升医学生公正世界信念对提升医学生职业素养有重要意义。积极心理资本是个体内心拥有的积极心理资源，与个体心理健康水平[5]关系密切。生命意义感是给予个体价值感和方向感的追求目标，即能够使个体感到自己的生活是有意义、有价值的，具有明确的目标规划，个体生命意义感水平偏低可以诱发心理矛盾与冲突，影响积极心理资本进而导致各类心理问题[6]，也可能影响个体的职业认同等思想信念[7]。为探讨医学生公正世界信念现状及其与生命意义感和积极心理资本的关系，本研究开展以下调查。

基金项目：江西省教育科学“十四五”规划2023年度课题（23YB228）
作者单位：1 赣南医科大学精神医学教研室，江西 赣州 341000；2 赣南医科大学苏区卫生与健康中国建设研究中心，江西 赣州 341000

1 资料与方法

1.1 一般资料

采用分层整群抽样，于2023年5—6月在赣南医科大学大一至大三选取16个临床医学班级共764名学生作为调查对象。纳入标准：1）已完成临床医学导论、心理健康教育等入学教育，对心理健康特点、我国医疗环境、医疗特点、医患关系及医学专业发展、就业、相关政策有一定了解的临床医学专业学生；2）自愿参与。排除标准：1）法医学等基础医学专业学生；2）已进入临床实践学习的医学生。共发放问卷764份，排除漏填、乱填等问卷，回收有效问卷753份，问卷有效率为98.56%。调查对象年龄17 ~ 25岁，平均（20.22±1.26）岁；男生286名，女生467名；大一学生219名，大二学生243名，大三学生291名；城镇生源医学生248名，农村生源医学生505名。

1.2 方法

采用公正世界信念量表、生命意义感量表和积极心理资本问卷对753名赣南医科大学医学生进行问卷调查。

1.3 观察指标

1.3.1 公正世界信念、生命意义感、积极心理资本调查

1）公正世界信念量表：本量表最初由Dalbert编制，由苏志强等[8]进行本土化修订，最终量表共13个项目。量表采用1 ~ 6点计分，量表总分范围为13 ~ 78分，总评分越高表示调查对象公正世界信念越强、越相信世界是公正的。本研究中，该量表Cronbach α 为0.917。

2）生命意义感量表：本量表最初由Steger等编制，共10个项目，采用1 ~ 7点计分。量表总评分越高，表明调查对象生命意义感水平越高。但刘思斯、甘怡群[9]在我国大学生群体中对该量表进行信、效度检验时，发现量表第10题存在双重负载故未予以保留。本研究采用国内9题的修订版。本研究中该量表Cronbach α 为0.797。

3）积极心理资本问卷：本问卷由张阔等[10]于2010年编制，包括希望、坚韧性、乐观、自我效能4个维度，共26个题目。问卷采用1 ~ 7点计分，量表总分范围为26 ~ 182分，问卷总评分越高，表明调查对象的积极心理资本水平越高，抗挫折能力越强，应对问题时内心资源越

中国知网 https://www.cnki.net

丰富。本研究中该量表 Cronbach α 为 0.901。

1.3.2 公正世界信念、生命意义感、积极心理资本相关分析

以医学生公正世界信念量表、生命意义感量表及积极心理资本问卷总评分进行相关分析，探讨三者间的关系。

1.3.3 生命意义感在医学生积极心理资本与公正世界信念间的中介效应分析

为进一步探讨三者间的关系，按中介效应研究过程[11]，分别以公正世界信念为因变量、积极心理资本为自变量，以生命意义感为因变量、以积极心理资本为自变量，以公正世界信念为因变量、以生命意义感和积极心理资本共同作为自变量，进行 3 次回归分析，以进一步分析生命意义感在医学生积极心理资本和公正世界信念间的中介效应。

1.4 统计学处理

本研究采用 SPSS 21.0 统计学软件对调查取得的数据进行统计分析。计数资料以 n（%）表示，计量资料以（$\bar{x} \pm s$）表示，2 组间比较采用两独立样本均数 t 检验，多组间比较采用单因素方差分析，以 Pearson 相关分析和回归分析考查医学生公正世界信念、积极心理资本和生命意义感等变量间的关系。检验水准 $\alpha = 0.05$。

2 结果

2.1 医学生公正世界信念、生命意义感、积极心理资本的一般情况

753 名医学生公正世界信念量表评分为（52.32 ± 10.00）分，生命意义感量表评分为（43.70 ± 7.00）分，积极心理资本问卷评分为（116.98 ± 18.08）分。其中，女生公正世界信念量表评分高于男生，大一医学生公正世界信念量表评分高于大二、大三医学生，差异有统计学意义（$P < 0.05$）。不同生源地医学生公正世界信念量表评分对比差异无统计学意义（$P > 0.05$）。见（表 1）。

2.2 医学生公正世界信念、生命意义感与积极心理资本的相关分析

医学生公正世界信念量表总评分、生命意义感量表总评分、积极心理资本问卷总评分分别呈正相关（$P < 0.05$）。见（表 2）。

2.3 生命意义感在医学生积极心理资本与公正世界信念间的中介效应检验

路径 a 统计结果显示，积极心理资本对医学生公正世界信念有显著预测作用（$P < 0.001$）。路径 b 统计结果显示，积极心理资本对医学生生命意义感的回归效应显著（$P < 0.001$）。路径 c、路径 c’ 统计结果显示，生命意义感和积极心理资本二者均对公正世界信念具有显著预测作用（$P < 0.001$），且积极心理资本对医学生公正世界信念的影响系数由 0.403 下降为 0.305。见（表 3）。

3 讨论

3.1 医学生公正世界信念受性别、年级的影响

本研究结果显示，女生公正世界信念量表评分高于男生，差异有统计学意义（$P < 0.001$），与其他大学生公正世界信念研究结论相似[12]。原因可能在于社会对不同性别赋予的角色差异：相对于女性，社会对男性施加更多的压力，并要求其承担更多的社会责任，而医学专业的特点决定从

表 1 753 名不同性别、生源地、年级医学生公正世界信念量表评分比较（分，$\bar{x} \pm s$）

因素	类别	公正世界信念	t / F 值	P 值
性别	女（n=467）	53.32 ± 9.53	3.525	< 0.001
	男（n=286）	50.70 ± 10.55		
生源地	城镇（n=248）	53.03 ± 9.87	1.360	0.174
	农村（n=505）	51.98 ± 10.06		
年级	大一（n=219）	53.59 ± 9.43	3.309	0.037
	大二（n=243）	52.42 ± 10.43		
	大三（n=291）	51.30 ± 9.98		

表 2 医学生危机公正世界信念、生命意义感和积极心理资本的相关分析（r）

项目	公正世界信念	生命意义感	积极心理资本
公正世界信念	–		
生命意义感	0.345①	–	
积极心理资本	0.403①	0.572①	–

注：①表示 $P < 0.05$。

表 3 医学生生命意义感在积极心理资本和公正世界信念间的中介作用检验

步骤	因变量	自变量	标准化回归系数	t 值	P 值	R^2 值
路径 a	公正世界信念	积极心理资本	0.403	12.059	< 0.001	0.161
路径 b	生命意义感	积极心理资本	0.572	19.088	< 0.001	0.326
路径 c	公正世界信念	生命意义感	0.171	4.245	< 0.001	0.180
路径 c’		积极心理资本	0.305	7.580	< 0.001	

注：路径 a 代表的是积极心理资本对医学生公正世界信念影响的回归方程；路径 b 代表的是积极心理资本对医学生生命意义感影响的回归方程；路径 c 代表的是生命意义感和积极心理资本对医学生公正世界信念影响的回归方程中生命意义感对公正世界信念的回归系数，路径 c’ 代表的是生命意义感和积极心理资本对医学生公正世界信念影响的回归方程中积极心理资本对公正世界信念的回归系数。

中国知网 https://www.cnki.net

业者需要接受长期的专业技能训练，从业年龄较晚，导致男医学生在毕业后较女生面临更大的就业、家庭、经济压力。因此，在校期间，男医学生更容易对“付出能否有回报”“就业是否公平公正”产生怀疑。提示不能因为医学专业的特点而忽视不同性别对社会的认识，未来有必要在思想政治教育或学生管理过程中针对不同性别开展宣传和教育。而高年级医学生较低年级医学生公正世界信念更低，其原因可能在于高年级医学生面临更大的升学、就业、实习压力，缺乏大一新生入学阶段的“新鲜感”，相对而言更容易怀疑自身专业的选择合理性，导致公正世界信念下降。但本研究发现不同生源地医学生公正世界信念对比差异无统计学意义（$P > 0.05$），与预想“因城乡二元化的差异，农村生源医学生公正世界信念更低”不同，其原因可能在于，相较城乡教育、经济水平、医疗卫生资源等差异，医学专业自身特点可能对医学生公正世界信念的影响更为显著。

3.2 医学生公正世界信念与生命意义感和积极心理资本关系密切

本研究中，医学生公正世界信念、生命意义感、积极心理资本间的相关分析结果表明，医学生人生目标越明确、规划越清晰、越具有明确的奋斗目标、内心心理资源越丰富、抗挫折能力越强、面对困难越乐观，其公正世界信念越强。一般情况下，医学生目标越明确，越相信能够通过自己的努力实现目标，公正世界信念自然越强；而积极心理资本的自我效能（个体具有足够的自信心面对困难并相信能够获得成功）、乐观性（对个体成功积极的归因）、希望性（个体对目标进行合理规划和调整以获得成功的能力）、坚韧性（遇到困难能够坚持、遇到打击能够自我调整与恢复以获得成功的能力）4个维度均赋予个体面对困难时的心理资源和从打击中恢复的能力，医学生积极心理资本水平越高，越具有面对挫折和困难的信心，自然更倾向于相信凭借努力获得成功，进而认为世界是公正的。上述研究提示，培养医学生的公正世界信念除进一步加强医学生职业素养教育、思想政治教育及医德医风教育外，心理健康教育也是重要途径，通过提升医学生的心理资源并赋予其明确的职业目标，可以更有效地增强其对世界公正的信念和认识。

3.3 生命意义感能够在医学生积极心理资本和公正世界信念间发挥部分中介效应

在相关分析的基础上，本研究进一步进行中介效应检验，结果显示，在医学生群体中，积极心理资本既能够直接影响公正世界信念水平，也能够通过生命意义感的部分中介效应对公正世界信念产生间接影响。从本研究相关分析结果可以发现，医学生积极心理资本水平与生命意义感呈正相关（$P < 0.05$），同时对生命意义感具有预测作用。而积极心理资本水平更高的医学生，其自我效能感更高，在缺乏明确目标、面对学业困境、就业压力时更容易保持乐观心态，进而更容易寻求学业过程中的意义和价值，提升其生命意义感。因此，在通过心理健康教育等途径提升医学生积极心理资本的同时，在学业过程中赋予医学生明确的目标和生命价值感也能够达到在一定程度上促进这一群体提升对公正的追求、相信自己能够得到公正的对待等公正世界信念水平的目的。其原因可能在于，当医学生具有更为明确的学习目标及价值，他们的心理资源也更为丰富，进而能够更为明确地探寻专业价值和医学的意义，更加坚定“天生我才必有用”的信念，对所处的医疗环境和医患关系更易接受，进而提升其公正世界信念。本研究进一步验证了目前医学院校在医学生职业素养教育中存在的问题：仅通过开设医学人文类课程往往不能显著提升其公正世界信念，只有从心理层面上予以改善才能达到加强医学生职业素养的目的。而积极心理资本是医学生抗挫折、内心心理资源等积极心态的体现，而在校期间这种积极心态能够通过赋予明确的学习意义和价值予以改善。提示全面提升医学生的积极心理资本较为复杂，而通过赋予他们明确的学习意义和价值，给予明确的奋斗目标，提升其生命意义感水平，同时通过临床实践、职业精神教育等方式达到提升其公正世界信念水平的方案更为切实可行。

3.4 建议与对策

本研究得到的启示在于，医学生公正世界信念虽然与医患关系、医学生就业压力、学业压力、住院医师规范化培训制度等因素有关，但同样也与自身心理特点有关。绝不能因为医学专业就业方向较为单一、医学专业自身特点需要具有人文精神等因素便认为他们具有更高水平的公正世界信念，而应通过强化生命意义感教育等方式予以改善。

首先，加强医学生使命教育，提升公正世界信念。部分医学生因多方面原因导致缺乏使命感，缺乏明确的奋斗目标，进而影响对社会的正确认识和公正的态度。未来医学院校可以考虑通过提升医学生使命感、责任感，培养医学生服务社会的理念，使其正确认识自己的人生价值，进而提升公正世界信念。如在学生管理过程中和思想政治教育、大学生心理健康教育、就业教育、创新创业教育等课程中开设职业生涯规划，帮助医学生规划自身的职业发展；

中国知网 https://www.cnki.net

组织医学生假期入乡镇卫生院等基层医疗机构了解我国医疗资源不均衡的现状，激发其基层就业的意识；组织医学生参观敬老院、儿童康复中心等机构进一步激发其责任意识。通过以上一系列手段，能够达到强化医学生生命意义感，提升责任意识，进而提升公正世界信念的目的。

其次，加强医学生乐观与抗挫折教育，提升公正世界信念。相对于生命意义感，积极心理资本影响因素较为复杂。在医学生培养过程中，医学院校也应考虑通过提升医学生的乐观特质、抗挫折能力等达到提升积极心理资本的目的，如可以考虑在校园心理健康知识宣传的过程中，通过组织积极心理学相关的演讲、征文、校园情景剧大赛、影片展等方式提升医学生对乐观的认识；通过适当增加课程难度、实验与讨论的教学过程中重视小组合作提升医学生对自身专业困难的认识，达到强化坚韧性的目的；考虑在医学生 5 年在校期间均开设心理健康教育，针对不同阶段开展相应的专题，如新生入学阶段开设专业认同及新生适应教育，在大二年级开设考试压力、人际关系心理健康教育，在高年级结合临床医疗特点开设常见心理障碍及调适、职业生涯规划等，帮助医学生有效处理各阶段的心理问题，使其正确认识自身面临的压力，有效解决问题，提升抗挫折能力，进而强化他们乐观与坚韧性等积极心理资本，使其通过自身努力克服困难，最终达到提升公正世界信念的目的。

最后，加强医学生人文精神的培养，有效提升公正世界信念。医学专业精神本身就包含公正世界信念，而有效的社会公正教育和个人道德修养的课堂教育是提升医学生公正世界信念的有效途径。但目前国内思想政治教育类课程教学过程中存在“重理论”“与实践结合不足”的缺陷[13]。因此，在开展医学生公正世界信念的教学过程中，一定要重视与专业教育结合、与职业教育结合。除在传统的思想政治理论课教学过程中需要进一步加强公正世界信念的教育外，在医学人文类课程中可以进一步考虑结合公正世界信念。如在医学伦理学课程教学中，通过“两难问题”启发医学生对公正、有利等医学原则的认识；在医患沟通课程教学中，医学生通过模拟临床实践中的医患沟通，进一步体验公正世界信念对临床医疗的意义；在医学心理学课程教学中，进一步重视针对患者心理干预等教学内容中积极关注的意义和价值。此外，在临床实践学习中，带教医生也需要在临床教学中重视实习生职业素养教育，进一步帮助他们将理论知识应用于医疗实践。通过参与式教学等多种途径，能够帮助医学生在专业学习过程中进一步内化公正世界信念。

综上所述，提升医学生公正世界信念是一个系统工程，不能仅通过单纯的理论知识教育，或寄希望于医学生对职业的“顿悟”，还需要从心理层面进一步提升他们对公正世界信念的认识。只有赋予医学生明确的奋斗目标和足够的心理资源，才能够有效提升他们对公正世界信念的理解，进而达到培养优秀医学人才的目的。

参考文献

[1] LERNER M J. Evaluation of performance as a function of performers reward and Attractiveness[J]. J Pers Soc Psychol，1965（4）：355−360.

[2] 杨莎，褚成静 . 人格特质、自尊和正念对高校大学生心理健康状况的影响 [J]. 中国健康教育，2023，39（11）：1046−1050.

[3] 王雷霞，尹小兰，费丽萍，等 . 医学院校大学生宿舍人际关系对抑郁情绪的影响分析 [J]. 现代预防医学，2022，49（13）：2409−2412.

[4] 王婧，陈勇，魏恒顺 . 基于健全人格取向的医学生心理健康状况分析及提升策略 [J]. 中国医学伦理学，2023，36（8）：941−946.

[5] 邹长华，信中贵，李泳汉，等 . 大学生情绪调节自我效能感和积极心理资本在心理弹性与心理健康关联中的作用 [J]. 中国学校卫生，2023，44（1）：94−98.

[6] 哈丽娜，常庆宁，陈旭 . 大学生心理扭力对自杀意念的影响：生命意义感与心理痛苦的链式中介作用 [J]. 中国健康心理学杂志，2022，30（8）：1228−1233.

[7] 龙锦花，王幼芳，邝焕明，等 . 生命意义感在 ICU 护士职业性悲伤和职业认同间的中介效应 [J]. 职业与健康，2023，39（22）：3086−3091.

[8] 苏志强，张大均，王鑫强 . 公正世界信念量表的修订及在大学生应用的信效度研究 [J]. 中华行为医学与脑科学杂志，2012，21（6）：561−563.

[9] 刘思斯，甘怡群 . 生命意义感量表中文版在大学生群体中的信效度 [J]. 中国心理卫生杂志，2010，24（6）：478−481.

[10] 张阔，张赛，董颖红 . 积极心理资本：测量及其与心理健康的关系 [J]. 心理与行为研究，2010，8（1）：58−64.

[11] 温忠麟，张雷，侯杰泰，等 . 中介效应检验程序及其应用 [J]. 心理学报，2004，36（5）：614−620.

[12] 陈心怡 . 大学生公正世界信念与网络利他行为的关系：共情与社会责任感的链式中介作用 [D]. 福州：福建师范大学，2021：45−46.

[13] 闻纯，徐志平，郭玲玲 . 医教协同背景下高职护理专业思政教育融入临床实践的研究 [J]. 卫生职业教育，2023，41（2）：106−109.

[收稿日期：2024−10−23]

中国知网 https://www.cnki.net
