# Supplementary material for: A study on the current status and influencing factors of psychological capital among medical undergraduates
Source: Front Psychol. 2026 Oct 30;17:1830730. doi: 10.3389/fpsyg.2026.1830730 (PMC13759799; doi:10.3389/fpsyg.2026.1830730)
Supplement: Supplementary file 1 [file Data_Sheet_1.ZIP › References PDF/A Study on the Relationship between Medical Students’ Sense of Efficacy in Learning and Positive Psychological Capital The Mediating Role of Professional Identity.pdf]

医学生学习效能感与积极心理资本的关系研究——专业认同的中介作用

徐胜男 常蕾 胡雪霏*

杭州医学院，浙江 杭州 311300

[摘要]目的 探究医学生专业认同在积极心理资本和学习效能间的中介作用，为提高医学生学习效能提供依据。方法 2022年6月~7月，在杭州某医学院校抽取656名在校大学生，采用积极心理量表、专业认同量表、学习效能量表进行调查。采用Pearson相关分析积极心理资本、专业认同、学习效能感之间的关系，使用SPSS插件Process 4.0进行中介效应检验，采用Bootstrap对医学生专业认同在积极心理资本和学习效能感的中介作用进行检验。结果 SPSS分析表明积极心理资本与专业认同呈显著正相关（$r=0.739$，$P<0.01$）与学习效能感呈显著正相关（$r=0.670$，$P<0.01$），专业认同和学习效能感呈显著正相关（$r=0.680$，$P<0.01$）。专业认同在医学生积极心理资本和学习效能感之间中介作用显著，中介效应值0.213，中介效应占总效应43.80%。结论 专业认同在积极心理资本和学习效能之间发挥部分中介的作用。

[关键词]医学生；学习效能感；积极心理资本

[中图分类号]B84 [文献标识码]A doi:10.19738/j.cnki.psy.2023.07.015

A study on the relationship between learning efficacy and positive psychological capital of medical students: the mediating role of professional identity

XU Shengnan, CHANG Lei, HU Xuefei*

Hangzhou Medical College, *Hangzhou* 311300, *China*

[Abstract] Objective To explore the mediating role of medical students' professional identity between positive psychological capital and learning efficiency, and to provide a basis for improving college students' learning efficiency. Methods June to July 2022, a total of 656 college students were selected from a medical college in Hangzhou and investigated with the positive psychological scale, professional identity scale and learning efficacy scale. Pearson correlation analysis was used to analyze the relationship between positive psychological capital, professional identity and learning efficacy. SPSS plug-in Process 4.0 was used to test the mediating effect. Bootstrap was used to test the mediating effect of medical students' professional identity on positive psychological capital and learning efficacy. Results SPSS analysis showed that positive psychological capital was significantly positively correlated with professional identity ($r=0.739$, $P<0.01$) and learning efficacy ($r=0.670$, $P<0.01$). Professional identity was significantly positively correlated with learning efficacy ($r=0.680$, $P<0.01$). Professional identity played a significant mediating role between positive psychological capital and learning self-efficacy of medical students. The mediating effect value was 0.213, and the mediating effect accounted for 43.80% of the total effect. Conclusion Professional identity plays a partial mediating role between positive psychological capital and learning efficacy.

[Key words] medical students; learning efficacy; positive psychological capital

学习效能感是指在学习场景中，学生展现出积极的学习行为能够主动使用学习策略并进行自我检查和自我调整，同时过程中收获并体验到积极的情感体验[1]。学习效能可以正向预测学业成就，即，学习效能感越高学业表现就越好[2]。积极心理资本是一种内在的积极心理力量，可以帮助个体应对压力与挑战，从而提高学业成绩和生活满意度[3]。心理资本是一种积极心理资源，它可以通过干预提高并且在一段时间内保持稳定[4]，它可以帮助个体应对成长中的诸多挑战[5]。医学生由于专业的特殊性更容易产生负面情绪和消极心理，从而产生学习倦怠降低学习效能[6]，已有研究证明积极心理资本可以提高个体的主观幸福感从而缓解压力和负面情绪[7]，心理资本有利于提高个体的学业成绩和绩效[8]。由此，提出假设1：积极心理资本对学习效能有直接影响。

专业认同是指学习者对所学的专业的接受和认可，从而在情感和行为上对专业产生归属和投入[9]。积极心理资本高的学生内在价值感更高，具有良好的自我动机从而提高

专业认同[10]，研究表明[11-12]，大学生心理资本与就业能力之间有显著的相关性积极心理资本高的学生更能对自己的专业前景进行预测，而对自己专业认同度高的大学生，更喜欢自己的专业，对专业的了解更深入，花费在专业学习上的时间更多，更容易以良好的心态、高效的学习方法投入到学习中，更不容易出现学习倦怠。由此，提出假设2：专业认同在积极心理资本与学习效能中具有中介作用。

综上，本研究以积极心理资本为自变量，学习效能感为因变量，专业认同为中介变量探究医学生积极心理资本和学习效能感之间的关系，为提升医学生的学习效能感提供方向和理论依据。

1 对象与方法

1.1 研究对象

本研究在2022年6月~7月，以杭州某医学院校在校大学生为主要调研对象，通过钉钉发送问卷星电子问卷，采用统一的指导语说明调查的目的、填写方法及注意事项，遵循自愿、匿名的填写方式。删除作答不完整或者答案完全一样的虚假问卷，最终获得有效问卷656份。纳入标准：医学专业1~4年级在校生，对本次调查知情同意，愿意配合完成调查；排除标准：不愿意参与调查，调查期间网络不畅无法完成调查，因个人原因休学和已经参加实习工作的同学。

1.2 调查工具

1.2.1 一般资料调查问卷：自行设计一般资料问卷，内容包括性别、年级、年龄、是否为独生子女、生源地、学历层析、是否为学生干部。

1.2.2 积极心理资本量表：积极心理资本量表（positive psychological capital scale，PPCS）[13]由张阔等在PCQ-24的

基金项目：2022年国家级大学生创新创业训练计划项目（202213023021）

作者简介：徐胜男（2001.09-），女，本科在读，研究方向：护理学
通讯作者：胡雪霏（1990.12-），男，硕士，助教，研究方向：学生思政和心理健康教育

(C)1994-2023 China Academic Journal Electronic Publishing House. All rights reserved. http://www.cnki.net

基础上编制，共26道题目，分为自我效能、韧性、希望、乐观4个维度。该量表采用Likert 7点评分，1～7分别表示从“完全不符合”～“完全符合”，无方向计分题，得分越高表示心理资本水平越高。该问卷具有良好的信度，量表的Cronbach's α系数为0.850。

1.2.3 学习效能感表：学习效能感量表（learning efficacy scale，LES）[14]是由梁宇颂于2000年在学业自我效能感量表基础上修订的，该量表共有22各题目，包括2个维度，分别为学习能力自我效能感、学习行为自我效能感。该量表采用5点计分方法，从1～5分别代表完全不符合、比较不符合、不确定、比较符合、完全符合，无反向几分题，量表总分越高代表个体学习效能感越高。该量表信效度良好，量表的Cronbach's α系数为0.820。

1.2.4 专业认同量表：专业认同量表（professional identity scale，PIS）[15]由秦攀博2009年编制的量表，共23个条目，分包括4个维度，分别为认知性、情感性、行为性、适切性，该量表采用5点计分方法，从1～5分别代表完全不符合、比较不符合、不确定、比较符合、完全符合。无反向几分题，量表总分越高代表个体专业认同越高。该量表信效度良好，量表的Cronbach's α系数为0.917。

1.3 统计学方法

使用SPSS 26.0进行数据录入，统计一般人口学资料，正态计量资料采用 $\bar{x}\pm s$ 表示，用Harman单因子检验共同方法偏差，采用Pearson相关分析相关性；使用SPSS插件Process 4.0进行中介效应检验，采用Bootstrap（5 000次抽样）对中介模型进行检验。

2 结果

2.1 一般人口学资料

研究对象的一般人口学资料如表1所示。

2.2 共同方法偏差检验

由于研究数据会受数据的来源、问卷的设计，和测量环境的影响，可能会出现共同方法偏差所以本研究运用Harman提出的单因素检验对所有量表题目进行单因素检验，结果显示，23个因子特征值大于1，抽取的第一公因子的方差解释百分比为27.89%（＜30%）[16]，因此本研究不存在共同方法偏差。

2.3 各变量之间相关性分析

相关性分析结果显示，医学生积极心理资本与专业认同之间呈显著正相关（$r=0.739$，$P<0.01$），与学习效能感呈显著正相关（$r=0.670$，$P<0.01$）；专业认同和学习效能感呈显著正相关（$r=0.680$，$P<0.01$)。各变量之间的相关关系为探讨领悟专业认同在积极心理资本与学习效能感的中介关系奠定了基础。详见表2。

表1 一般人口学资料（n，%）

项目	类别	例数	占比
性别	男	171	26.10
	女	485	73.90
年级	大一	284	43.30
	大二	176	26.80
	大三	113	17.20
	大四	83	12.70
生源地	农村	405	61.70
	城镇	251	38.30
独生子女	是	258	39.30
	否	398	60.70
学生干部	是	388	59.10
	否	268	40.90
学历	本科	398	60.70
	专升本	42	6.40
	专科	177	27.00
	高职	39	5.90

表2 各变量之间的相关性分析（n=656）

变量	$M\pm SD$	积极心理资本	专业认同	学习效能感
积极心理资本	94.48±15.97	1		
专业认同	87.68±15.64	0.739**	1	
学习效能感	73.15±11.58	0.670**	0.680**	1

注：**$P<0.01$

表3 中介效应检验

回归方程		拟合指标			系数显著性		
结果变量	预测变量	R	R^2	F	β	SE	t
专业认同	积极心理资本	0.742	0.551	113.562	0.726	0.026	27.702***
	年级				-0.001	0.394	0.004
	生源地				-0.270	0.903	-0.299
	是否是独生				0.490	0.898	0.546
	是否是学生干部				0.109	0.864	0.127
	性别				-1.401	0.972	-1.441
	学历				0.366	0.422	0.867
学习效能	积极心理资本	0.729	0.531	91.517	0.275	0.029	9.362***
	专业认同				0.294	0.030	9.888***
	年级				0.169	0.307	0.551
	生源地				-0.406	0.684	-0.593
	是否是独生				0.405	0.680	0.596
	是否是学生干部				0.262	0.654	0.400
	性别				-1.584	0.738	-1.869*
	学历				0.162	0.307	0.551*
学习效能	积极心理资本	0.676	0.456	90.815	0.488	0.021	22.942***
	年级				0.194	0.329	-0.589
	生源地				-0.485	0.733	-0.661
	是否是独生				0.550	0.729	0.754
	是否是学生干部				0.294	0.701	0.419
	性别				-1.996	0.790	-2.528*
	学历				0.710	0.343	2.070*

注：*$P<0.05$；***$P<0.001$

(C)1994-2023 China Academic Journal Electronic Publishing House. All rights reserved. http://www.cnki.net

2.4 积极心理资本的中介效应检验

基于专业认同、积极心理资本、学习效能感之间存在显著的相关性，采用Hayea编制的SPSS宏程序Process 4.0进行中介效应检验。首先对专业认同、积极心理资本、学习效能感3个主要变量进行标准化处理，应用模型4，以积极心理资本为预测变量，专业认同为中介变量，学习效能感为结果变量，年级、生源地、是否为独生子女、是否是学生干部、性别学历为控制变量，检验专业认同在积极心理资本与学习效能感的中介效应。结果如表3所示，积极心理资本正向预测医学生的学习效能（$\beta=0.488$，$P<0.001$），积极心理资本越高医学生学习效能越高，假设1成立；积极心理资本正向预测医学生的专业认同（$\beta=0.726$，$P<0.001$），当积极心理资本和专业认同同时预测医学生学习效能感时，积极心理资本和专业认同均显著正向预测医学生学习效能感（$\beta=0.275$，$P<0.001$；$\beta=0.294$，$P<0.001$）。由此，积极心理资本会通过专业认同影响医学生的学习效能感，即专业认同是调节积极心理资本与学习效能感之间的中介变量。

研究进一步采用Bootstrap法检验中介效应的显著性，若各效应量的95%置信区间不包括0，则表示中介效应显著。结果如表4所示，积极心理资本对学习效能感的总效应量是0.488，其95%的置信区间[0.447，0.530]不包含0，表示总效应显著；积极心理资本对学习效能感的直接效应是0.275，其95%置信区间[0.217，0.332]，表示直接效应显著；当介入专业认同时，中介效应量是0.213，其95%的置信区间[0.160，0.267]不包含0，表示中介效应显著。综上，专业认同在积极心理资本对学习效能感的影响中起显著的中介作用，假设2成立，中介效应占总效应的43.80%，中介模型及路径系数如图1所示。

表4 中介效应分析

效应	效应值	Bootstrap *SE*	*t*	95%的置信区间	相对效应(%)
总效应	0.488	0.021	22.942***	[0.447, 0.530]	100.00
直接效应	0.275	0.029	9.362***	[0.217, 0.332]	56.20
间接效应	0.213	0.027		[0.160, 0.267]	43.80

注：***$P<0.001$

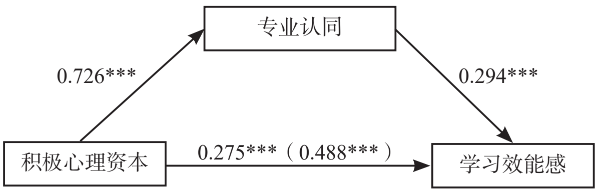

图1 中介模型及路径系数

注：***$P<0.001$

3 讨论

本研究基于积极心理学理论，考察了医学生心理资本与学习效能感之间的关系，探究了专业认同在积极心理资本与学习效能感之间的中介作用，建立了医学生积极心理资本对学习效能感的影响机制，有助于我们更好地提升医学生的学习效能感。

3.1 积极心理资本能正向预测学习效能感

研究数据显示，医学生的积极心理资本与学习效能感呈显著正相关，即积极心理资本越高的医学生学习效能越高，而在加入专业认同这一中介变量后，医学生的积极心理资本和学习效能感仍呈正向关系，这与前人的研究结果一致[17]。医学专业不仅学制长而且难度大，不仅需要学习专业知识还需要学习专业技能知识，这对医学生理论学习能力和实践动手能力提出了更高的要求。导致医学生学习难度大，学生容易产生挫败感，产生消极心理，从而影响学习效能感。有研究发现，接受了积极心理训练的同学自控能力、自我效能、觉察力、自我调节能力显著提高，从而提高学习效能和缓解、减少消极心理[18]。积极心理资本高的同学更容易觉察到当下的学习状态，并自我更新，进而以开放、探索的积极态度面对应激事件[19]。因此，提高医学生的学习效能可以从训练和增加学生的积极心理资本入手。这对教师提出了更高的要求，医学高校教师作为教学与专业双领域的专业人才不仅需要帮助学生解决学业学习上问题，还需要了解学生的心理，关心学生帮助学生形成积极心理资本，帮助其增加对于学习的自信感和效能感，引导学生树立学习兴趣，主动解决学习和生活中遇到的各种问题[20]。

3.2 专业认同在积极心理资本与学习效能间起部分中介作用

研究进一步发现，积极心理资本与专业认同呈正相关，专业认同和学习效能感呈正相关，专业认同在医学生积极心理资本和学习效能感之间发挥部分中介的作用，中介占比43.80%。说明积极心理资本既能够直接正向作用于学习效能感，又可以通过专业认同间接影响学习效能感，这与前人的研究结果相一致[21]。研究中发现积极心理资本高的医学生具有高的专业认同，这可能与高积极心理资本的同学对于专业学习的觉知力更高，更容易体验到积极情绪，也更愿意探索新的学习经验，改变自我认识偏差，这有利于形成高专业认同。高的专业认同，可以感受在专业中的自我价值，更愿意接受职业培训，从而可以影响个体的思维和行为，促进其对自身专业知识和技能的肯定，进而促进专业学习效能。因此，提高医学生的专业认同是提高学习效能的重要方法。在学生学习的过程中应给予学生充分的支持，实施就业和专业教育，让学生深入了解所学专业，在专业课程的学习中，教师可引入临床案例，给予学生正向的专业情感，提高对专业的认同度使医学生愿意并主动投入更多的时间进行专业课的学习，从而提高学习效能感。

4 策略建议

4.1 增强教师的支持作用

教师是高校医学生重要的可控的外部环境，积极的教师支持，如：学习支持、情感关怀、能力认可等，可以促进学生心理资本的积累，减少学生由于学习产生负面情绪，提高学习积极性和持久性。教师应主动与学生进行沟通，使学生感受到更多的教师支持。教师应密切关注学生的身心发展状况，尤其是对处于心理危机的学生，营造良好的支持系统。教师作为学生学习和认识自身专业的领头人，应发挥自身的榜样作用，通过自己的专业技能素养给予学生正向的专业引导。

4.2 培养学生的心理资本

学校应根据自身情况广泛开展与积极心理有关的心理健康课程，个体或者团体心理辅导，帮助学生调动积极的情绪，不仅可以使学生在学习过程保持积极的兴趣体验还可以帮助学生体验到教师的支持和帮助。组织和让学生参与校园活动和竞赛，帮助发展积极的心态，以便他们能够面对困难和挑战，鼓励学生展示他们的能力和潜力，帮助他们建立自信，以便他们能够相信自己可以克服困难帮助他们发现自己的成功，并为他们实现更高的目标提供机会。通过向学生提

下转第（129）页

(C)1994-2023 China Academic Journal Electronic Publishing House. All rights reserved. http://www.cnki.net

其是在工作和学业方面的功能缺损，但未能明显提升生活质量满意度。考虑与CCBT程序中认知行为疗法干预密切相关，帮助患者纠正错误认知，促进心理康复。另外，以计算机为媒介，通过音频、视频等形式的放松练习和认知功能训练，也可使患者掌握能够更好适应社会的认知和行为调整方法，调动自身的主动性和积极性，改善应对技能，恢复社会功能，由于本研究治疗时间及研究周期较短，生活满意度的改善需在长期随访中继续评估，因此，在今后研究中需适当增加治疗干预时间及随访次数，以得出更全面客观的结论。

（收稿日期：2023-01-11；修回日期：2023-02-23；编辑：张唯婷）

参考文献

[1] Huang Y, Wang Y, Wang H, et al. Prevalence of mental disorders in China:a cross-sectional epidemiological study[J]. Lancet Psychiatry, 2019,6(3):211-224.

[2] 刘晓秋,白志军.中国抑郁症患者自杀危险因素的元分析[J].中国临床心理学杂志,2014,22(2):291-294.

[3] Wittchen H U, Jacobi F. Size and burden of mental disorders in Europe--a critical review and appraisal of 27 studies[J]. Eur Neuropsychopharmacol, 2005,15(4):357-376.

[4] Ustun T B, Ayuso-Mateos J L, Chatterji S, et al. Global burden of depressive disorders in the year 2000[J]. Br J Psychiatry,2004,184:386-392.

[5] Chisholm D. Investing in mental health[J]. East Mediterr Health J,2015,21(7):531-534.

[6] Friedrich M J. Depression Is the Leading Cause of Disability Around the World[J]. JAMA,2017,317(15):1517.

[7] 季建林.中国抑郁障碍防治指南修订与抑郁障碍的规范治疗[J].中华行为医学与脑科学杂志,2015,24(4):292-293.

[8] Titov N, Andrews G, Sachdev P. Computer-delivered cognitive behavioural therapy:effective and getting ready for dissemination[J]. F1000 Med Rep,2010,2:49.

[9] 朱军红,钟宝亮. Sheehan残疾量表在美沙酮维持治疗门诊患者中应用的信效度研究[J].中国药物依赖性杂志,2010,19(4):281-284.

[10] 唐茂芹,邱惠敏,简佳.简明幸福与生活质量满意度问卷在中国精神障碍患者中的信效度[J].中国心理卫生杂志,2010,24(9):680-684.

[11] 张甜怡.基于认知行为疗法的自助干预对抑郁症残留症状的效果研究[D].湖州:湖州师范学院,2020.

[12] 范燕飞,孙红立,刘美玲,等.米氮平联合CCBT治疗轻中度抑郁症的效果及对生活质量的影响[J].四川精神卫生,2019,32(5):413-417.

[13] Geramita E M, Herbeck B B, Abebe K Z, et al. The Association Between Increased Levels of Patient Engagement With an Internet Support Group and Improved Mental Health Outcomes at 6-Month Follow-Up:Post-Hoc Analyses From a Randomized Controlled Trial[J]. J Med Internet Res,2018,20(7):e10402.

[14] Kim D R, Hantsoo L, Thase M E, et al. Computer-assisted cognitive behavioral therapy for pregnant women with major depressive disorder[J]. J Womens Health(Larchmt),2014,23(10):842-848.

上接第（56）页

供资源和机会，帮助他们找到希望，以便他们能够相信自己能够克服困难，并对未来充满乐观。

4.3 建立学生的专业认同

专业认同的形成是一个复杂的过程，会受多方因素的影响，因此需要多方进行协作。教师通过向学生介绍专业知识，让他们对该专业有更深入的了解，并为他们提供更多的了解机会。学校因组织实习和见习等直接与医院接触的活动，给学生提供发展机会，让学生参加实习，让他们亲身体验这个专业的实践和应用，从而了解该专业的实际情况让他们在实践中展现自己的能力和技能。

5 研究的不足

研究主要在杭州某所医学院校进行，限制结果的推广性，未来的研究可扩大样本范围提高结论的推广性。其次本研究属于横断面研究调研，阐释研究变量因果关系受限，未来可开展纵向研究进一步验证。

（收稿日期：2023-02-04；修回日期：2023-03-14；编辑：齐亚梦）

参考文献

[1] 王红瑞,付凡伦.宝鸡市农村地区初中生学习效能感、学习投入与心理健康的关系研究[J].职业与健康,2022,38(8):1116-1120.

[2] 石常秀.初中生学习适应性、学习自我效能感与学业成绩的关系[D].南京:南京师范大学,2006.

[3] 臧运洪,伍麟.人际信任和心理复原力在贫困大学生积极心理资本与挫折心理间的中介作用[J].中国学校卫生,2016,37(3):387-390.

[4] 王文龙,鞠鑫,杨翀.大学生的心理资本、社会支持与心理症状之间的关系研究[J].高教学刊,2022,8(18):58-61,65.

[5] 喻小艳,喻鹏,张晓霞,等.本科护生心理资本、职业认同与择业效能感的相关性分析[J].卫生职业教育,2022,40(11):77-79.

[6] 陈姝含,娄欣然,尹洁,等.医学生情绪调节自我效能感对学业倦怠的影响[J].职业与健康,2022,38(16):2255-2259.

[7] 余琼,王彦娜,柳林.优秀贫困大学生积极心理资本现状及影响因素[J].中国学校卫生,2022,43(4):562-565,569.

[8] 鹿丰玲.大学生心理资本对学习成绩和人际关系的影响[J].开封教育学院学报,2017,37(7):161-163.

[9] 孙潇,王文文,王泽炫.大学生专业认同度与学业成绩的关系:心理资本的中介作用[J].保定学院学报,2020,33(1):111-114.

[10] 李洪,刘蕾,钟丽丽.基于自我决定理论的护理本科生学习动机模型的构建与验证[J].护理学杂志,2020,35(22):77-80.

[11] 陈保睿.大学生心理资本、专业认同与学业倦怠间的关系研究[D].长春:东北师范大学,2016.

[12] 孙潇,王文文,王泽炫.大学生专业认同度与学业成绩的关系:心理资本的中介作用[J].保定学院学报,2020,33(1):111-114.

[13] 张阔,张赛,董颖红.积极心理资本:测量及其与心理健康的关系[J].心理与行为研究,2010,8(1):58-64.

[14] 梁宇颂.大学生成就目标、归因方式与学业自我效能感的研究[D].武汉:华中师范大学,2000.

[15] 秦攀博.大学生专业认同的特点及其相关研究[D].重庆:西南大学,2009.

[16] 周浩,龙立荣.共同方法偏差的统计检验与控制方法[J].心理科学进展,2004,12(6):942-950.

[17] 周国蔚.初中生积极心理资本与学习投入的关系[J].中小学心理健康教育,2022,20(11):4-8.

[18] 乐鑫.正念训练对大学生注意觉知和情绪调节自我效能感的干预实验[D].桂林:广西师范大学,2017.

[19] 李静,杨威.大学生学业自我效能感、内控性人格和正念对自主学习能力的影响研究[J].广西教育,2020,67(47):140-143.

[20] 李博文.中职生感知教师支持对学习投入的影响:心理资本与专业认同的链式中介作用[J].教育科学论坛,2022,36(36):21-26.

[21] 常旭芳.大学生学习投入与积极心理资本的关系研究——专业认同的中介作用[J].现代交际,2021,35(24):133-135.

(C)1994-2023 China Academic Journal Electronic Publishing House. All rights reserved. http://www.cnki.net
