# Supplementary material for: A study on the current status and influencing factors of psychological capital among medical undergraduates
Source: Front Psychol. 2026 Oct 30;17:1830730. doi: 10.3389/fpsyg.2026.1830730 (PMC13759799; doi:10.3389/fpsyg.2026.1830730)
Supplement: Supplementary file 1 [file Data_Sheet_1.ZIP › References PDF/A Survey on the Current Status of Psychological Capital Among Medical Students in the Context of Core Socialist Values A Case Study of Z University.pdf]

社会主义核心价值观下医学生心理资本现状的调查研究——以Z大学为例

陈　彤　张英楠

摘要：社会主义核心价值观对医学生的医德培育、人文关怀、社会责任感塑造具有积极引领作用。本研究以Z大学医学生为研究对象，采用问卷法调查法探究其心理资本现状，并据此提出培养路径。研究发现，医学生的心理资本在年级上呈不平衡态势；女生心理资本总体水平及乐观维度显著高于男生；城市生源医学生心理资本各维度得分均显著高于农村生源；担任学生干部及学业成绩居中上的医学生心理资本发展较好。基于此，在社会主义核心价值观引领下，应深化其与医学教育的融合，强化实践育人环节，并构建系统化、多层次的心理支持体系，以有效提升医学生心理资本。

关键词：社会主义核心价值观；医学生；心理资本；现状调查

一、研究价值与意义

心理资本的概念最早应用于经济学与社会学领域，指个体在成长过程中表现出来的一种积极心理状态。医学生的培养目标是以临床医生岗位胜任能力为导向，培养具有医学科学精神和医学人文精神，较强实践能力和终身学习能力的临床医学专门人才[1]。医学生的培养年限长，课程内容多，学习任务繁重，因此这些培养目标的达成离不开个体拥有健康的身心。心理资本是一种内心的积极信念，它会帮助医学生继续努力学习，好好生活，在面对未来生活时，无论是怎样的处境，内心依然坚定。对医学生心理资本的研究有助于培养他们拥有乐观品质，提升他们的自信水平、抗挫折能力，进而促进医学生具有良好的心理素质，其综合素质也会得到全面提升[2]。社会主义核心价值观对医学生的医德培育、人文关怀、社会责任感等方面有积极影响。社会主义核心价值观下医学生心理资本的研究，既是医学教育改革的必然要求，也是应对社会健康挑战的重要举措。其价值在于通过心理资源开发与价值观引领的协同作用，培养出兼具专业能力与人文精神的医学人才，最终推动医疗行业的高质量发展与社会的全面进步。

二、研究对象与方法

Z大学是一所医科大学，本研究以该校医学生为研究对象，共发放问卷730份，回收有效问卷722份，有效率为98.90%。本研究使用SPSS23.0软件进行数据分析。本研究使用学者路桑斯编制的心理资本问卷，该问卷是学术界较为成熟的心理资本量表，已被学者广泛使用。该问卷共24题，主要分为四个维度：1~6题为自信水平；7~12题为希望水平；13~18题为韧性水平；19~24题为乐观水平[3]。该问卷采用利克特（Liken）5点记分法记分，问卷总分为120分。总分108分及以上，代表个体的心理资本水平很高，工作、生活中的挑战并不会给其带来太多影响，能积极面对并创造新的机会。总分96~107分，代表个体的心理资本水平相对较高，能够主动应对工作、生活中的挑战，经过自我调整，可以保持积极的心理状态。总分72~95分，代表个体的心理资本水平良好，会有情绪波动，但愿意尝试面对并解决工作、生活中的挑战。总分71分及以下，代表个体的心理资本水平相对较低，经常会有挫败、悲观的状态，日后，需要加强和关注自己的心理资本水平，可以适度寻求外部支持[4]。在研究中，该量表的内部一致性系数为0.954。

三、社会主义核心价值观下医学生心理资本的现状及特点

（一）医学生心理资本发展呈现显著的年级不平衡特征

数据显示，自信水平在大五年级最高，希望、乐

中国知网　https://www.cnki.net

观及总分在大三年级最高，韧性水平在大二年级最高。单因素方差分析表明，在乐观维度上，大二、大三学生的得分显著高于大五年级（$p < 0.05$）。

从总体趋势看，大三年级医学生的心理资本整体状况较好。大一年级医学生刚刚经过高考进入校园，对大学校园生活的一切都是陌生的。这一阶段也是医学生从基础教育阶段进入高等教育阶段的过渡期。随着其对学校的了解和专业课程学习的深入，加上从各种学生会以及社团活动中得到的锻炼[5]，从大二年级开始医学生心理资本呈上升趋势，到大三年级达到最好的状态。

（二）女性医学生心理资本总体水平及乐观维度显著高于男生

本研究收集的722份问卷，其中回收男生问卷305份，回收女生问卷417份。对Z大学医学生的心理资本总体水平及各个维度做性别上的差异检验。经检验，在乐观水平上，男生与女生存在显著差异（$t = -2.032$，$p < 0.05$），女生得分显著高于男生。这说明女生在乐观水平上发展好于男生，与男生相比，女生更具有积极的归因方式，并对现在和未来持积极态度。在心理资本的总体上，男生与女生也存在显著差异（$t = -0.686$，$p < 0.05$），女生得分显著高于男生。

由于男女在生理上、身体素质上均存在差异，从原始社会开始，男性女性的社会职能就有所不同。两性差异心理学认为，青春期之后男性和女性的心理发展在认识结构、神经机能活动特征以及感觉器官适应刺激的速度上均存在差异。个体的兴趣爱好、观念、态度、抱负水平、情绪等方面，两性差异也比较明显。

（三）城市生源医学生心理资本各维度发展均优于农村生源

本研究收集的722份问卷，其中回收城市生源问卷503份，回收农村生源问卷219份。对Z大学医学生的心理资本总体水平及各个维度做生源地上的差异检验。经检验，医学生心理资本的四个维度以及总体得分均在生源地上存在显著差异（$t = 4.289$，$p < 0.01$；$t = 3.662$，$p < 0.01$；$t = 3.290$，$p < 0.01$；$t = 3.219$，$p < 0.01$；$t = 4.070$，$p < 0.01$）。城市生源医学生的四个维度以及总体得分均显著高于农村生源的医学生。

城市生源的医学生与农村生源的医学生在大学生活适应性上存在一定的差异。城市生源的医学生的交往能力强，其交际能力与交际范围要广于农村生源的医学生。由于经济基础不同，农村生源医学生的消费以满足基本需求为主，城市生源医学生的消费除满足基本需求外，还会增加娱乐休闲方面的消费。城市生源的医学生绝大多数家庭里有从事医疗相关行业的父母或亲属，而农村生源的医学生较为缺乏社会支持，这就导致在未来就业问题上农村生源的医学生会产生一定的担忧。这些差异都会给农村生源的医学生造成一定的心理压力，导致其心理资本发展低于城市生源的医学生。

（四）担任学生干部的医学生心理资本发展水平较高

本研究收集的722份问卷，其中担任学生干部的问卷291份，不担任学生干部的问卷431份。对Z大学医学生的心理资本总体水平及各个维度做学生干部任职情况上的差异检验。经检验，医学生心理资本的四个维度以及总体得分均在是否担任学生干部上存在显著差异（$t = 3.519$，$p < 0.01$；$t = 2.662$，$p < 0.01$；$t = 2.518$，$p < 0.05$；$t = 2.947$，$p < 0.01$；$t = 3.243$，$p < 0.01$）。担任学生干部的医学生的四个维度以及总体得分均显著高于没有担任学生干部的医学生。

学生干部是维持学校管理稳定有序运用的重要群体。担任学生干部也是对学生的一种锻炼，他们具有较高的政治觉悟和思想品质，具有良好的沟通能力、分析能力、决策能力、组织能力、应变能力。由此可见，担任学生干部可以明显改善学生的心理资本状况。

（五）学业成绩处于中上等的医学生心理资本发展较好

根据医学生上一学年的学业成绩排名，将其分为学业成绩前20%、20%~40%、40%~60%、60%~80%、80%以后五个等级。研究发现，学业成绩在20%~40%的医学生其心理资本总体水平及各维度得分较高。对医学生的心理资本数据做学业成绩上的单因素方差分析发现，学业成绩在20%的医学生其自信、希望、韧性、乐观得分均显著高于在60%~80%以及80%以后的医学生（$p < 0.05$）。学业成绩在20%~40%的医学生其自信、希望、韧性、乐观得分均显著高于在60%~80%以及80%以后的医学生（$p < 0.05$）。学业成绩在40%~60%的医学生其自信、希望、韧性、乐观得分均显著高于在60%~80%以及80%以后的医学生（$p < 0.05$）。

研究发现，并不是学业成绩最好的医学生的心理资本及各个维度得分最高，反而是学业成绩处于20%~40%的医学生的心理资本及各个维度得分最高。学业成绩处于40%~60%、60%~80%、80%以后的医学生的心理资本及各个维度得分依次降低。学业成绩最好的医学生往往其他方面也较优秀，性格上更为要强，他们如果想保持住当下的状态就需要付出更多的努力（如获得推免资格等），因此他们的心理压力会比学业成绩处于20%~40%的大。学业成绩处于20%~40%的医学生，他们在学习学生或生活中不会过于要求自己事事争得第一，因为不会给自己较大的压力，反而在身心上表现得更轻松。学业成绩处于40%~60%、60%~80%、80%以后的医学生，他们既要面对学业压力，还要面对社会分流以及就业的压力，因此他们的心理压力要大于学业成绩排在前面的医学生。

中国知网 https://www.cnki.net

四、社会主义核心价值观下医学生心理资本的培养路径

心理资本是可以更新的，且经证实，通过短期和高聚焦的微干预措施可以得到显著提高[6]。在社会主义核心价值观的引领下，对医学生心理资本的培养不仅契合新时代医学教育的发展需求，也为提升医疗服务质量、构建和谐医患关系提供了新的视角。

（一）深化社会主义核心价值观与医学教育的融合渗透

社会主义核心价值观强调“敬业、友善、诚信”等个人层面的价值准则，而心理资本作为积极心理资源，能够为医学生的职业道德培育提供内在动力。因此促进社会主义核心价值观与医学教育的融合有助于培养医学生的心理资本。第一，重视思政课程的价值引领。敬业精神与心理资本中的“希望”和“自我效能”密切相关，通过思政课目标设定和能力提升，可强化医学生的职业使命感。这种融合为医学教育理论注入了心理学视角，丰富了社会主义核心价值观的实践内涵。第二，将社会主义核心价值观融入“医学伦理学”“医学心理学”等课程，通过案例教学（如医患沟通中的诚信实践）强化“敬业”“友善”等价值准则。第三，开设“积极心理资本开发”等选修课，系统教授自信、希望、乐观、韧性四大维度，结合医学生实习压力场景设计心理韧性训练。

（二）构建以实践体验为核心的心理资本培育机制

社会主义核心价值观与中医“大医精诚”理念高度契合，在实践中弘扬中华优秀传统文化中的医德精神，引导医学生将“仁爱”“奉献”等传统美德内化为职业信念，进而培养其心理资本。第一，为学生多提供临床情境模拟训练。如高仿真模拟人实训，使用具备生理反馈功能的标准化病人（SP）和模拟人系统，创设急诊抢救、医患冲突等高压场景。第二，在实习阶段设置“临终关怀”“医患冲突处理”等情境模拟，通过角色扮演强化“救死扶伤”的职业认同，培养在压力下保持情绪稳定的能力。第三，以志愿服务实践为载体，积极组织“三下乡”义诊、医院导诊志愿者、社区健康服务等社会实践，使学生在利他行为中体验“友善”“奉献”的价值感。通过以上路径可以在实践体验中提升医学生的职业素养与抗压能力，改善医患关系、增进社会信任。

（三）完善高校系统化、多层级心理支持体系

在新时代医学教育的大背景下，医学生面临着学业、临床实践以及未来职业发展等多方面的巨大压力，培养他们的心理资本显得尤为重要。高校作为培养医学生的重要阵地，提供心理支持并构建系统性、多层次的支持体系刻不容缓。首先，提供精准化心理咨询服务是关键一环。高校应建立分层干预机制，通过季度心理普测全面了解学生的心理状态。例如，在每学期初开展大规模的心理健康问卷调查，运用专业的心理测评量表，如症状自评量表（SCL-90）等，精准识别学生的心理问题倾向。依据测评结果，建立“轻中重”三级预警档案。对于轻度心理问题的学生，安排心理中心教师定期进行一对一的心理辅导，通过谈心谈话帮助他们缓解压力；对于中度心理问题的学生，除了教师辅导外，还可以组织小组心理治疗活动，让学生在相互交流中获得支持；而对于重度心理问题的学生，则立即启动危机干预程序。同时，为满足学生随时可能产生的心理求助需求，设置24小时心理热线，安排经过专业培训的心理咨询师值班，确保学生在遇到困扰的第一时间就能得到及时、有效的心理支持。其次，建立医教协同咨询模式能极大提升心理危机干预的效率。高校应积极组建“精神科医生＋心理中心教师＋辅导员”团队。精神科医生凭借其专业的医学知识，能够对学生的心理问题进行准确的医学诊断；心理中心教师擅长运用心理学专业方法进行心理辅导和治疗；辅导员则熟悉学生的日常学习和生活情况，能更好地提供人文关怀和后续跟进。当发现心理危机学生时，为其开通绿色转介通道。比如，当辅导员发现学生出现严重的情绪低落、行为异常等情况，可立即通过绿色转介通道将学生转介给精神科医生进行紧急评估和诊断，随后心理中心教师介入，制定个性化的心理治疗方案，辅导员全程协助，共同帮助学生走出心理困境。

参考文献

[1]FRED LUTHANS,CAROLYN M.Youssef. Human, Social,and Now Positive Psychological Capital Management:Investing in People for Competitive Advantage[J]. Organizational Dynamics,2004(02):143-160.

[2] 路桑斯 . 心理资本——打造人的竞争优势 [M]. 李超平 , 译 . 北京 : 中国轻工业出版社 ,2007:221-222.

[3] 微奢糖 . 你要去相信 [M]. 北京 : 中国水利水电出版社 ,2021:249-252.

[4] 刘畅 , 张雁儒 . 河南省高职院校医学生心理资本的现状 [J]. 郑州大学学报 (医学版),2013(05):691-692.

[5] 王昭 , 孙丽岩 , 王福东 . 网络信息化变革与社会变迁对医学生的影响及教育启发 [J]. 法制与社会 ,2015(07):231-232.

[6] 苏伟 , 曹伟 . 医学生社会主义核心价值观认知现状调查 [J]. 济宁医学院学报 ,2014,37(05):350-352.

本文得到以下项目支持：中共沈阳市委教科工作委员会关于2026年沈阳市大中小学思政课一体化研究课题“后大学生自尊权变性的探索与研究”（SDSZ-2026-082）。

（作者：陈彤，中国医科大学第一临床学院助教；张英楠，中国医科大学第一临床学院讲师）

中国知网 https://www.cnki.net
