# Supplementary material for: A study on the current status and influencing factors of psychological capital among medical undergraduates
Source: Front Psychol. 2026 Oct 30;17:1830730. doi: 10.3389/fpsyg.2026.1830730 (PMC13759799; doi:10.3389/fpsyg.2026.1830730)
Supplement: Supplementary file 1 [file Data_Sheet_1.ZIP › References PDF/An Analysis of the Current State of Medical Students’ Professional Values in the Context of a Public Health Emergency.pdf]

公共卫生事件背景下医学生职业价值观现状分析

谷晶晶 黄云飞 李永欢 黄伊玟 马乐乐 张文轩 杨仕佳 朱颖▲

【摘要】目的 探讨公共卫生事件背景下医学生职业价值观现状并分析其影响因素。方法 2022年9—12月，采用分层等比例随机抽取华北理工大学护理、临床和预防专业190名医学生进行问卷调查。了解医学生职业价值观现状及其影响因素。结果 公共卫生事件背景下医学生的职业价值观中位得分为2.620，处于中等偏下水平；专业认同感和积极心理资本中位得分分别为3.317和4.601，处于中等偏上水平。多元逐步线性回归结果显示，父母从事相关职业、家庭人均月收入、专业类型、专业认同、积极心理资本是医学本科生职业价值观主要影响因素。结论 公共卫生事件背景下医学生的职业价值观有待改善，受多种因素影响，应采取措施提高医学生职业价值观，引导其积极就业。

【关键词】职业价值观；医学本科生；公共卫生事件；影响因素;专业认同感；积极心理资本
【中图分类号】G647 【文献标志码】A
【文章编号】1004-6763（2025）05-0192-05
doi: 10.3969/j.issn.1004-6763.2025.05.042

近年来突发公共卫生事件呈现出全球性蔓延、难以预料及掌控、后果不堪想象等特点，对世界各地的危机管理体系及医疗救护能力不断提出考验[1]。各类突发公共卫生事件不仅使人民群众的生命健康遭受重大威胁，而且，使与患者密切接触的临床一线医护人员面临极大的感染风险。巨大的工作负荷和心理压力容易导致医护人员产生负面情绪和应激反应，影响身心健康。在校医学生是医疗卫生事业的后备军，他们对突发公共卫生事件的应激状态以及认知、态度等行为表现，能够直接反映其医学职业素养和责任担当，对职业价值观产生影响，进而影响医学生的职业认同、职业抉择和高质量就业。因此本研究随机抽取华北理工大学在校医学生进行调查，分析其职业价值观现状及影响因素，旨在寻找公共卫生事件背景下影响医学生职业价值观取向变化的因素，为消除负面取向，促进医学生树立正确的职业价值观提供依据，现报道如下。这项研究具有重要意义，不仅可以帮助学校和医学院更好地关注和引导学生的职业发展，也为整个医疗卫生事业的未来培养了更有责任感和使命感的医护人才做出贡献。通过努力消除负面因素，促进医学生对于医学事业的热爱和投入。

作者单位：华北理工大学护理与康复学院，河北 唐山 063210
▲通信作者：朱颖，E-mail：781808250@qq.com

1 资料与方法

1.1 一般资料

2022年9—12月，华北理工大学在读全日制1~4年级护理学、1~5年级临床医学和预防医学本科生，采用分层等比例随机抽样方法，随机抽取190名医学本科生作为研究对象。

纳入标准：（1）华北理工大学在读本科生；（2）符合以上三个专业中任一专业；（3）同意参加本研究并签署知情同意书。

排除标准：（1）停学、休学者；（2）参军等特殊原因无法及时联系者；（3）有心理疾病问题者；（4）受其他原因影响，中途退出此次调查研究者。

1.2 方法

1.2.1 研究工具

自制调查问卷，收集研究人群一般情况，包括性别、年级、专业、生源地、家庭人均月收入、是否为独生子女、父母是否从事相关职业、学习成绩、是否担任过班干部、学医原因、重新选择是否会选择医学专业。

1.2.2 资料收集方法

采用问卷星线上电子问卷调查的形式，线上填写完毕后立即提交。线上问卷链接开放时间为2022年9月—12月，问卷题项均设置为必答题项，以保证问卷填写的完整性。本次调查发放电子问卷190份，回收190份，回收率100%；剔除无效问卷后，获得有效问卷180份，问卷有效率94.73%。

1.3 观察指标

大学生职业价值观量表[2]分为内在价值观（利他

中国知网 https://www.cnki.net

主义、独立性、美感、变异性或追求新意、智力刺激)、外在价值观(成就感、安全感、社会地位、经济报酬)、外在报酬(管理、人际关系、社会交际、舒适)。汉化版量表的 Cronbach's α 系数为0.760,重测信度为 0.639,信效度良好。

大学生专业认同量表[3]包括职业认知、职业行为、职业情感三个维度。共 15 个条目,该量表的 Cronbach's α 系数为 0.816,重测信度为 0.767,信效度良好。

大学生积极心理资本量表[4]包括乐观维度、韧性维度、希望维度、自我效能维度。该量表的 Cronbach's α 系数为 0.869,重测信度为 0.797,信效度良好。

1.4 统计学处理

采用 SPSS 23.0 统计学软件分析。正态分布的计量资料用 $\bar{x}\pm s$ 表示;计数资料用例数(百分比)[n(%)]表示。医学生职业价值观、专业认同、积极心理资本得分和已知总体的比较采用单样本 t 检验;采用多元逐步线性回归对医学生职业价值观进行影响因素分析,以 $P<0.05$ 为差异有统计学意义。

2 结果

2.1 研究人群的一般情况

对 180 名医学生的一般情况进行统计。女生占比 68.889%;护理、临床、公卫专业人数各占 33.333%;大一至大四人数均占 21.667%,大五占 13.333%。生源地以农村为主,占 68.333%。多数学生(82.222%)父母未从事相关职业,学习成绩中等的占 49.444%,家庭人均月收入一般(<3000 元/月)的占 59.444%。非独生子女占 80.000%,多数学生(66.667%)未担任班干部。学医原因中因自己喜欢报考的占62.222%,且 59.444% 的学生表示重新选择仍会选本专业,见表1。

2.2 医学本科生职业价值观现状

基于调查问卷采用5点评分法进行评分,取3为中间参照点。医学本科生职业价值观中位得分 2.620,职业价值观中位得分与中间参照点3进行秩和检验,医学本科生职业价值观处于中等偏下水平,差异有统计学意义($P<0.001$)。从构成职业价值观的维度来看,外在价值观中位得分最高,其次是外在报酬,最低的是内在价值观,即外在价值观>外在报酬>内在价值观。但三个维度均没有达到一般水平,见表2。

2.3 医学本科生专业认同现状

基于调查问卷采用5点评分法进行评分,取3为中间参照点。医学本科生专业认同中位得分 3.317,专业认同中位得分与参照点3进行秩和检验得出,医学本科生专业认同现状处于中等偏上水平,差异有统计学意义($P<0.001$)。从构成专业认同的维度来看,职业行为得分最高,其次是职业认同,最低的是职业情感,即职业行为>职业认同>职业情感。三个维度均高于参考值,见表3。

表1 180名医学生的一般情况

项目	例数	百分比(%)
性别		
女	124	68.889
男	56	31.111
专业		
护理	60	33.333
临床	60	33.333
公卫	60	33.333
年级		
大一	39	21.667
大二	39	21.667
大三	39	21.667
大四	39	21.667
大五	24	13.333
生源地		
农村	123	68.333
城市	57	31.667
父母是否从事相关职业		
否	148	82.222
是	32	17.778
学习成绩		
中等(专业30%~60%)	89	49.444
一般(专业59%~100%)	55	30.556
较好(专业前29%)	36	20.000
家庭人均月收入		
一般(<3000元/月)	107	59.444
中等(3000~5000元/月)	56	31.111
较好(>5000元/月)	17	9.444
是否为独生子		
否	144	80.000
是	36	20.000
大学是否担任 班干部		
否	120	66.667
是	60	33.333
学医原因		
自己喜欢报考	112	62.222
重新选择是否会选择本专业		
家长推荐报考	68	37.778
是	107	59.444
否	73	40.556

中国知网 https://www.cnki.net

表2 职业价值观现状[分,$M(P_{25},P_{75})$]

项目	项目得分	Z值	P值
外在价值观	2.824(2.500,3.135)	−6.057	<0.001
内在价值观	2.652(1.807,3.122)	−7.017	<0.001
外在报酬	2.669(2.176,3.090)	−7.797	<0.001
职业价值观	2.620(2.307,2.961)	−9.855	<0.001

表3 专业认同现状[分,$M(P_{25},P_{75})$]

项目	项目得分	Z值	P值
职业情感	3.163(2.538,3.875)	−2.540	0.011
职业行为	3.462(2.981,3.918)	−8.314	<0.001
职业认同	3.423(2.885,3.942)	−7.229	<0.001
专业认同	3.317(3.098,3.747)	−9.746	<0.001

2.4 医学本科生积极心理资本现状

基于问卷采用7点评分法，取4为中间参照点。医学本科生积极心理资本中位得分4.601，积极心理资本中位得分与参照点4进行秩和检验得出，医学本科生积极心理资本现状处于中等偏上水平，差异有统计学意义（$P<0.001$）。从构成积极心理资本的各维度看，自我效能维度得分最高，其次是乐观、希望，最低是韧性，四个维度均超过一般水平，见表4。

表4 积极心理现状[分,$M(P_{25},P_{75})$]

项目	得分	Z值	P值
乐观	4.442(4.000,5.000)	−8.561	<0.001
希望	4.769(4.192,5.038)	−13.141	<0.001
韧性	4.808(4.231,5.115)	−13.183	<0.001
自我效能	4.885(4.192,5.231)	−12.625	<0.001

2.5 医学生职业价值观的多元逐步线性回归分析

以职业价值观为因变量，积极心理资本现状、专业认同现状、研究人群的一般情况为自变量，进行多元逐步线性回归分析。结果表明，父母是否从事相关职业、专业类型、家庭人均月收入、专业认同、积极心理资本进入多元逐步线性回归模型，是职业价值观的主要影响因素，回归方程为：医学生职业价值观=0.800+0.314×父母从事相关职业+0.401×专业+0.347×家庭人均月收入+0.395×专业认同+0.166×心理资本。见表5。

3 讨论

职业价值观作为价值观在职业生涯中的具体表现，影响职业生涯的导向[5]。当前大学生普遍存在着职业价值观认识模糊、职业生涯规划不足问题，而且在职业价值观上还呈现出“物质主义”和群体性差异[6]。这意味着许多大学生更关注物质利益和社会地位，而缺乏对于职业发展、自我实现的认识和追求。医学生职业价值观是医药卫生类学生职业生涯准入的精神标尺，对学生的专业学习、职业选择、职业行为、职业发展都有着重要影响[7]。公共卫生事件的发生，医护人员首当其冲，参与临床救治工作，会激发医学生的社会责任感，但也会引发他们的心理负担，产生消极影响。因此，本研究着眼于医学生的职业价值观，旨在研究公共卫生事件背景下医学生的职业价值观现状及其影响因素。通过深入了解医学生在面对公共卫生事件时的职业价值观变化和态度，我们可以更好地指导他们树立正确的职业理念、提高社会责任感，并建立积极的心理健康状态，从而帮助他们更好地应对职业生涯中的挑战，为未来的医疗卫生事业做出积极贡献。

本研究结果显示，在公共卫生事件背景下，我校医学本科生的职业价值观整体处于中等偏下水平。这表明，尽管受到公共卫生事件的影响，医学本科生对自己未来要从事的医疗职业有一定了解，并表现出一定的倾向行为，但整体上还没有完全接受并内化这一职业的核心价值观。心理发展规律显示，人们对负性

表5 职业价值观的多元逐步线性回归分析

项目	B	SE	t值	OR(95%CI)	P值
父母是否从事相关职业	0.314	0.099	3.155	0.401(0.196～0.606)	0.002
专业类型	0.401	0.104	3.857	0.314(0.117～0.51)	<0.001
家庭人均月收入	0.347	0.103	3.361	0.347(0.143～0.551)	0.001
专业认同	0.395	0.063	6.305	0.395(0.271～0.518)	<0.001
积极心理资本	0.166	0.053	3.121	0.166(0.061～0.271)	0.002
常量	0.800	0.210	3.900	0.801(0.396～1.206)	<0.001

注：R=0.721，调整后R^2=0.507，F=39.909，df=5.000，$P<0.001$。赋值[父母是否从事相关职业：是(1)、否(0)；专业类型：护士(1,0,0)、临床(0,1,0)、公卫(0,0,1)；家庭人均月收入：一般(1)、中等(2)、较好(3)；专业认同和积极心理资本原值带入]

中国知网 https://www.cnki.net

事件更敏感，更容易受负性事件的影响。突发公共卫生事件期间，部分医学生参与了救治外围工作，高年级医学生更可能在临床实习过程中接触到一线救治带来的超负荷工作等负性事件，对医学生心理造成一定的压力影响。在这样的背景下，研究建议高校可以通过职业教育课程、讲座、社会实践等方式，促进医学生职业价值观的全面提升。通过系统的职业教育课程，学生可以更好地了解医疗行业的现状和未来发展趋势，培养对医学职业的认同感和责任感。丰富多样的讲座和社会实践活动可以让学生更加深入地了解医疗领域的挑战和机遇，促使他们建立积极的职业态度和价值观。这些措施将有助于提升医学生的职业素养，增强他们的职业发展能力，为未来的医疗卫生事业培养更加优秀和有责任感的专业人才。

心理资本是个体行为的积极心理状态，积极心理资本研究的兴起为提高自我效能和改善职业认同提供了新的研究方向[8]。本研究结果显示，医学本科生的专业认同和积极心理资本均处于中等偏上水平。突发公共卫生事件应对过程中，无数青年医护工作者舍小家顾大家，闻令而动，积极响应国家号召，彰显了医务人员的专业素养与职业精神。这些正面行为极大的激励和鼓舞了医学生的家国情怀，也有助于提高他们的积极心理资本和专业认同。也有研究报道，紧张的医患关系和重大公共卫生事件是导致医学生产生未来职业焦虑的主要原因[9]。建议高校用好、用活先进典型事迹作为职业价值观教育的鲜活素材，邀请校内外专家、知名人士、杰出校友等进校园、进课堂、进生活，使职业价值观教育、工匠精神等走进学生的认知、感化学生行动，起到教育内化于心、外化于行的积极引导作用[10]。另外，引导学生理性认识和分析当前的医疗环境和医患关系，避免被夸大或不实宣传的影响，有助于学生保持对未来职业的信心和积极态度。通过全面了解行业情况和正确看待挑战，学生可以更好地应对职业生涯中的困难和压力，确保自己的职业发展走向成功和健康。

医学生职业价值观是医学生未来从事临床工作的内在心理基础，直接影响着从事临床工作的倾向和工作中的职业行为。多元线性回归结果表明，父母从事相关职业、专业类型、家庭人均月收入、专业认同、积极心理资本是医学生职业价值观的主要影响因素。这些因素不仅影响医学生对医学专业的认同程度，也塑造了他们的职业态度和行为。王志芳等[11]研究报道，医学生职业价值观的形成与父母的支持程度密不可分，与本研究结论一致。父母从事医学相关工作，能够潜移默化的对子女产生影响，子女能够亲身感受到医护人员无私奉献的救死扶伤精神，从而使其对医学专业价值有更高的认同感。对于护理、临床和预防三类医学专业，由于突发公共卫生事件的影响，预防专业人员需求量激增，专业重视程度得到迅速提升，对职业价值观的认同起到积极的正面影响作用。经济情况较好的家庭，子女的教育不受经济条件制约，具有更加积极的职业价值观。心理资本对应激事件的发生结果起着“缓冲”的作用，是一种积极的心理资源，心理资本水平越高，越会在学习和开发潜能上花费更多的时间和精力，越能更好地促进学生职业认同水平的发展[12]，与本研究结论一致。医学生对专业越有信心，职业认同感越高。遭遇挫折时越乐观，对职业定位的认知也就越充分，并愿意付出更多精力去克服逆境。因此，应采取措施来提高医学生的心理资本和专业认同感，通过积极传播正能量、弘扬正面形象等方式，激励医学生保持信心、面对职业挑战，并为他们的职业发展提供良好的表率和模范作用。这样能够帮助医学生更好地应对未来的临床工作，促进其个人和职业发展的稳健和健康[13]。但本研究仅为华北理工大学选取的医学生样本，代表性存在一定的局限，未来需要进一步采用多中心大样本调查研究。

综上所述，公共卫生事件背景下医学生职业价值观有待进一步提高，父母从医、家庭人均月收入良好、就读社会需求量大的专业、专业认同感高和积极心理资本是主要影响因素，应对公共卫生事件时应关注医学生群体，通过多种措施提升其职业价值观。重视医学生职业价值观对个人与医疗卫生事业发展意义重大。

参考文献

[1] 王琤.公立医院参与突发公共卫生事件应对的问题与策略研究[D].济南：山东大学，2022，1−9.

[2] 薛晶，刘红云.“职业价值观问卷（WVI）”的信

中国知网 https://www.cnki.net

效度检验、验证性因素分析及北京市大学生职业价值观现状的研究[C].中国心理学会.第九届全国心理学学术会议文摘选集，2001：167-168.
[3] 安蓉，胡道圣.高职学生职业认同现状及影响因素分析—基于对天津市的调查[J].职业技术教育，2015，36（19）：63-67.
[4] 彭丹蕾，陈诗建，何宇雁，等.大学生积极心理资本与就业能力关系及其干预研究[J].广东职业技术教育，2021（4）：120-123.
[5] 宋瑞璇.大学生职业价值观建构实证研究[J].杨凌职业技术学院学报，2021，20（3）：28-32.
[6] 张文龙，叶一舵.新时代中国青年的职业价值观：基于CGSS数据的分析[J].福建师范大学学报（哲学社会科学版），2019（5）：69-77，169.
[7] 张欣.后疫情时代医药卫生类高职学生职业价值观现状调查与对策建议[J].卫生职业教育，2023，41（6）：53-56.
[8] KIM M. The factors associated with person-centered care competence among nursing students [J]. International Journal of Environmental Research and Public Health，2022，19（5）：2787.
[9] 刘萍，刘婧，潘长玲.职业选择焦虑在高职护理专业学生的状态-特质焦虑水平对职业选择影响中的中介作用[J].护理实践与研究，2021，18（14）：2064-2067.
[10] 李兵，杨婷.职业价值观视域下高职生就业能力提升问题研究[J].职业技术教育，2021，42（20）：68-71.
[11] 王治芳，牛青云.基于家校协同的家长教养能力三维结构理论模型建构[J].中国成人教育，2021（8）：7-16.
[12] 王紫媚，李月娥.本科实习护生心理资本在职业认同与自我效能中的中介作用[J].全科护理，2022，20（27）：3759-3762.
[13] TREMBLAY MS，BARNES JD，GONZ?LEZ SA，et al. Global matrix 2.0：Report card grades on the physical activity of children and youth Comparing 38 countries [J]. Journal of Physical Activity & Health，2016，13（11 Suppl 2）：343-366.

[收稿日期：2025-02-01]

欢迎订阅《中国公共卫生管理》杂志

《中国公共卫生管理》杂志创刊于1985年，是由国家卫生健康委员会主管，中华预防医学会、黑龙江省卫生健康发展研究中心主办的医药卫生事业管理类学术期刊。本刊贯彻新时期卫生与健康工作方针，坚持理论与实践相结合，普及与提高相结合，报道我国公共卫生管理学术研究最新进展，反映我国公共卫生管理改革实践最新成果，为完善我国公共卫生体系建设和提升我国公共卫生管理水平服务。本刊主要读者对象为：全国专业公共卫生机构卫生人员，基层医疗卫生机构卫生人员，卫生健康行政管理人员，全国各级各类医疗机构公共卫生职能科室管理人员，承担管理职能的公共卫生技术人员，高等院校、科研机构的相关教学、研究人员。本刊重点栏目：公共卫生治理、公共卫生资源、公众健康、公共卫生服务、全球健康、公共卫生管理与实践等。

本刊欢迎有关公共卫生管理科学及相关选题的原始研究论著，涉及医药卫生体制改革、公共卫生体系与制度、个体与公众健康、公共卫生服务与公共卫生组织运营、公共卫生组织管理与外部治理、健康与疾病等方面的理论与实证研究、述评、综述，以及公共卫生管理理论、技术、方法在实践中应用的新鲜经验，国外公共卫生管理学术研究趋势和动态。

本刊为双月刊，邮发代号：14-319，定价：每册20元，年定价：120元。国内外公开发行。

作者通过《中国公共卫生管理》杂志网站（http:// ggws.cbpt.cnki.net）在线投稿。编辑部地址：哈尔滨市香坊区中山路112号，电话：0451-87253010。

中国知网 https://www.cnki.net
