# Supplementary material for: A study on the current status and influencing factors of psychological capital among medical undergraduates
Source: Front Psychol. 2026 Oct 30;17:1830730. doi: 10.3389/fpsyg.2026.1830730 (PMC13759799; doi:10.3389/fpsyg.2026.1830730)
Supplement: Supplementary file 1 [file Data_Sheet_1.ZIP › References PDF/Characteristics and Multidimensional Development of Psychological Capital in College Students.pdf]

·教育理论与实践·

大学生心理资本的特征与多维开发

常硕峰

(吉林大学 哲学社会学院,长春 132000)

[摘 要] 心理资本概念由卢森斯等人提出,指个体的积极心理发展状态,并由自我效能、乐观、希望和韧性四要素构成。它通过主效应、缓冲效应、调节效应和动态效应等四种模型机制发挥作用。其中,动态效应是我国学者根据心理资本和结果变量之间存在交互作用的现实,对前三种模型整合而来,并成为心理资本反应机制研究的新趋势。根据心理资本构成"四种作用机制"以及当前大学生心理资本呈现的不平衡性、可测量性和双重性特征,其心理资本开发途径为:激发动机、调动认知资源,使个体体验到成功的过程及成就感;面对挫折引导他们养成正确的归因方式;创造条件,逐渐增强他们的希望体验;构建社会支持体系,塑造他们坚韧自强的心理品质。

[关键词] 大学生;心理资本;卢森斯;动态效应

[中图分类号] G640;B84 **[文献标志码]** A **[文章编号]** 1000-8284(2014)03-0206-04

当今,心理学的主流乃是"以人为本"的人本主义,注重挖掘人的潜力,注重自我价值的实现。在这样的时代背景下,积极心理学在 20 世纪 90 年代应运而生。心理资本这个概念既是在积极心理学和积极组织行为学的框架下提出来的,注重人心里的积极方面,通过培养和开发员工的心理资本以提高工作绩效,并且最大化地创造经济效益,这也是人们逐渐把心理资本引入人力资源管理的体系中来的重要原因。除了以往对社会资本和人力资本的关注,心理资本也越来越多地成为企业选拔人才的指标之一。心理资本不仅在企业中受到重视,高校学生的心理资本水平也尤其需要受到关注。近几年频发的高校大学生自杀以及杀害室友案件提出警示,我们必须重视高校学生的心理健康问题。本文从心理资本的视角切入,探讨大学生的心理资本的水平以及为心理资本的开发提出对策性建议。

一、心理资本的缘起及构成要素

1997 年,经济学家高德史密斯(Goldsmith)和戴瑞特(Veum Darity)认为,心理资本是指能够影响个体的生产率的一些个性特征,这些特征反映了一个人的自我观点或自尊感,支配着一个人的动机和对工作的一般态度[1]。这时的心理资本概念着重应用在经济领域,主要是为了提高员工的工作绩效,创造更多的剩余价值。经过这一时期的发展,心理资本被纳入到人力资源管理体系中来,除了注重员工的人力资本(个体的知识技能)和社会资本(个体的社会关系网络),又逐渐成为人力资源管理的新趋势。2002 年,积极心理学之父塞利格曼(Seligman)基于人力资本理论,将"心理资本"这个概念引入到心理学的范畴。2004 年,卢森斯(Luthans)及其同事以积极心理学(positive psychology)和积极组织行为学(positive organizational behavior)[2][3]的观点为思考框架,在分析经济资本、人力资本和社会资本的特点和区别后,提出了以强调人的积极心理力量为核心的"积极心理资本(positive psychological capital)"概念[4]。2005

[收稿日期] 2013-01-06

[作者简介] 常硕峰(1990-),女,黑龙江哈尔滨人,硕士研究生,从事心理学、社会学研究。

(C)1994-2023 China Academic Journal Electronic Publishing House. All rights reserved. http://www.cnki.net

年，卢森斯等人明确将心理资本定义为“个体一般积极性的核心心理要素，具体表现为符合积极组织行为标准的心理状态，它超出了人力资本和社会资本之上，并能够通过有针对性的投入和开发而使个体获得竞争优势”[5]。2007 年，卢森斯(Luthans) 、尤瑟夫(Youssef) 和阿瓦里奥(Avolio) 又对心理资本的定义进行了修订，认为心理资本是指“个体的积极心理发展状态”，其特点是: (1) 拥有表现和付出必要努力、成功完成具有挑战性的任务的自信(自我效能感) ; (2) 对当前和将来的成功做积极归因(乐观) ; (3) 坚持目标，为了取得成功，在必要时能够重新选择实现目标的路线(希望) ; (4) 当遇到问题和困境时，能够坚持、很快恢复和采取迂回途径来取得成功”(韧性)[6]。大多数的学者也是比较认可将心理资本分为自我效能、乐观、希望和韧性四大要素。

二、心理资本的作用机制

1. 主效应模型。主效应模型可以理解为心理资本对个体、团体、组织、公司的某些结果变量有着直接的改善作用，并且心理资本不受其他因素的影响，不与其他变量产生交互作用。心理资本可以为企业带来巨额的利润。有研究证明，心理资本水平每提升 2%，经济效益会增加 1000 多万美金。不仅如此，心理资本与员工的工作满意度、组织承诺、工作态度、工作绩效、工作疏离感、离职倾向等显著相关[7]; 心理资本对企业领导的行为有效性也呈明显正相关[8]。心理资本的主效应机制不仅在员工身上有所体现，而且能够有效地预测高校大学生的心理健康水平[9]。这些研究均表明，心理资本在对个体或组织产生影响时均发挥主体作用。而且，其自我效能、希望、乐观、韧性四个要素结合起来对个体的影响要大于各个要素之和。

2. 缓冲效应模型。缓冲效应模型认为，心理资本通过中介效应对结果变量起作用，即心理资本间接地影响个体的工作绩效、心理健康等水平。我国学者对这一模型做出了实践检验证明: 惠青山认为，组织公平感和对主管的评价等变量，使心理资本对员工态度、行为变量的影响中起中介变量的作用[10]; 魏红权认为，组织支持感、工作满意感在心理资本对关系绩效的影响中起中介变量的作用[11]; 田喜洲的实验表明，接待业员工的心理资本在对其工作行为与工作态度的影响关系中，满意度起重要的中介作用[12]; 滕少霞的实验表明，员工的心理资本对其工作满意度、组织承诺和工作投入等工作态度的影响是通过组织气候这个中介变量而产生作用的[13]。同样，以大学生为被试同样验证了这一点: 潘清泉、周宗奎发现大学生应对方式在心理资本与心理健康状况之间起中介变量的作用; 章倩的实验表明，心理资本在学校支持感对其职业决策困难的影响中起积极的中介作用; 张阔、付立菲等人的研究表明，学生的学习策略在大学生心理资本与其学业成绩之间的关系中起中介变量的作用[14]。但是，中介作用的程度如何，完全中介还是部分中介，需要进一步地在实验中进行回归分析等相关研究。

3. 调节效应模型。调节效应模型，简言之，即心理资本在影响个体或组织的结果变量时起到的调节作用。例如，2006 年，科尔(Cole) 以失业员工为研究对象发现，在失业后的主观满意感与再就业的关系中，心理资本起着调节作用，心理资本水平越高，主观满意感对再就业的促进作用就越明显[15]。还有卢森斯、阿瓦里奥、艾薇(Avey) 、屈艳、井维华等国内外学者均做出类似的实验验证了调节效应模型，在此不再赘述。

4. 动态效应模型。以上三种效应模型均是对心理资本作用机制的单一看法，但是，在现实工作、学习中，心理资本的多种作用机制可能同时发生，即心理资本和结果变量之间存在着交互作用的关系，这其中，可能是线性关系也可能是非线性关系，鉴于此，我国学者王雁飞等对上述几种作用机制进行了整合，提出了一种新的作用机制——动态效应模型。该模型认为，心理资本与结果变量间的关系是交互的、动态的，可能随着时间空间的变化而变化，几种作用机制可能同时发生或间或发生。动态效应模型在目前阶段尚处于理论假设阶段，还需要更多的实验数据进行支撑，虽不够完善，但可以肯定的是，动态效应模型将是心理资本反应机制研究的新趋势。

三、大学生心理资本的特征

大学生作为社会的中坚力量，肩负着贡献社会、服务社会的伟大使命。对当前大学生的心理资本的发展水平以及特征的研究就显得尤为重要。根据以往学者的研究，本文将大学生心理资本总结概括为:

1. 不平衡性。高校学生心理资本发展的不平衡主要指两方面: 一是心理资本的各要素之间发展不平衡，二是学生与学生之间的心理资本发展水平不平衡。由于大学生心理资本状态的复杂性，且心理资本尚处于理论初期，除了包括自我效能感、乐观、希望、韧性四个要素，很多学者在编制问卷施测时，又都

(C)1994-2023 China Academic Journal Electronic Publishing House. All rights reserved. http://www.cnki.net

加入了符合积极组织行为学标准的其他积极心理结构，例如主观幸福感、智力等很多因素。蒋苏芹在编制大学生问卷时就加入了创新意识、包容宽恕、情绪智力、责任意识、成就动机五个方面。蒋苏芹的实验表明，大学生心理资本水平总体处于较高状态，但是各维度之间差异显著，其中责任意识的心理资本水平最高，希望的心理资本水平最低[16]。并且，心理资本各维度在性别、年级等人口学变量上差异也相当显著。心理资本状态的形成原因有很多种，无论是心理的、生理的，亦或是学校、家庭的教育、教养环境，种种的细微差异均可能造成心理资本水平之间的不平衡。

2. 可测量性。随着心理资本理论的不断发展，其测量问卷也应运而生。只有数量化的心理状态，才更易于对心理资本水平进行准确、精确、数量化、易操作的分析。研究表明，心理资本的测量问卷的确很多，但是国外的问卷主要还是围绕乐观、希望、韧性、自我效能四个因素，且国外的问卷在我国的信度、效度也并不是很高的，因此，我国学者不断地进行量表的本土化研究，除了提高了信度与效度，还扩充了维度，使得心理资本的结构要素更加全面。并且，心理资本问卷的施测对象也愈加准确了，从企业员工为被试主体到全体个体，然后又精确到大学生等特定群体。这种被试主体由特定群体扩展到普通大众又抽象成特定群体的过程，也恰恰证明了心理资本理论正不断地走向成熟。

3. 双重性。大学生心理资本具有双重性，即在特定的时间和空间条件下，心理资本具有相对的稳定性; 但是，在经过一定的训练和开发后，心理资本水平也是可以改变和提升的，这也证明了心理资本的可塑性。这种相对的稳定性也被称为特质性，认为心理资本就像个体身上的特质，如智力、态度、人格等特质，是随着个体、环境、遗传的不断对其加工改造，一旦形成后就很难改变，个体在一系列行为反应中均反映出这样的特质。2003 年，美国学者豪森(Hosen) 等人指出，心理资本是个体通过学习等途径进行投资后所获得的一种具有耐久性和稳定性的积极内在心理构念，这一心理构念主要包括一个人的个性品质、认知能力和自我监控力等[17]。而这种可塑性也被人称为状态性，即认为心理资本是可以重塑和开发的，通过对心理资本的培养，可以使得个体的工作绩效、工作态度等一系列行为反应得到改变。这种观点首先被塞利格曼所提出，认为心理资本是一种积极的心理状态。卢森斯、阿瓦里奥等人均支持这种观点，认为心理资本是指能够导致个体积极组织行为的积极心理状态，它能够通过有针对性的投入和开发而使个体获得竞争优势。随着心理资本理论的不断发展，特质论和状态论也逐渐走向融合，大学生的心理资本所具有的双重性，也被称为综合论。

四、对大学生心理资本的多维开发

1. 自我效能感的开发。自我效能感是由学者班杜拉提出的，是指个体对自己在特定的情境里能够激发动机，调动认知资源，以及采取必要的行动来完成某一项特定工作的信念或信心[18]。班杜拉也曾提出过提升自我效能感的四种方法，即直接经验、榜样作用、社会说服以及情绪唤醒。针对大学生这个特殊群体，自我效能感的开发首先需要其个体体验到成功的过程及成就感。榜样作用实质上就是一种替代性经验，其效果强度可能要弱于直接经验，但是当学生想要树立一个中长期目标，又不知道目标是否过高、能否实现，这时，榜样的作用就会凸显出来。榜样让学生看见了曙光，榜样的作用更像是一场激励。榜样的诚悦说服，无不是晓之以理、动之以情。当代高校学生是张扬个性的一代，对于很多事情有自己独特的看法。这种诚悦说服一定要针对不同的事件、不同的环境、不同的对象采取不同的沟通策略。反之，则会适得其反。但可以肯定的是，说服的主体一定要在社会中具有一定地位，这样才能使学生信服。最佳的途径是经常组织学者或者其他社会知名人士来学校进行讲座，分享他们成功的经验和过程，让学生可以自由提问，这种交流试的说服更易于接受。

2. 乐观情绪的开发。大学生的乐观情绪一般来源于对事件、对生活的体验和感知，这种认知状态会决定一个人的心态是否积极。所谓认知过程，即大学生对生活中所遇到的事件的一种解释，一种归因过程。根据维纳的归因理论，归因有三个维度: 可控性，即事件的原因是个人能力控制之内还是之外; 稳定性，即事件的原因是暂时的还是稳定的; 内外性，即事件的原因是内在的还是外在的。当大学生倾向于把失败、挫折的原因归因于内在的、稳定的、不可控时，则会给他们造成挫败感，在学习中甚至会产生习得性无助，使之没办法保持一种积极乐观的心态。针对这种情况，须调整其认知状态，引导他们面对困难进行积极的解释和归因。例如，有的学生有社交胆怯的倾向，认为自己胆小无能，这时应该鼓励他们勇敢地去向他人伸出求援之手，帮助自己提高社交经验和技巧，只要多多锻炼，这种技能是不难学会的。而所有这些都取决于大学生树立积极乐观的心态，养成正确的归因方式。

3. 希望心理的开发。根据卢森斯等人提出的心理资本干预模型，要想获得希望，需要制定一系列

(C)1994-2023 China Academic Journal Electronic Publishing House. All rights reserved. http://www.cnki.net

计划及其目标，克服各种各样障碍，并努力去实施这些计划。其整个过程的实现会让实施者获得希望的感觉，日积月累，这种希望的心理则会成为一种稳定的心理资本，在任何情境中均会获得希望的体验。针对大学生群体，希望的获得主要来自学校。因此，在学校文化环境下，老师、同学以及与他们的关系均直接影响着主体希望心理的萌生。在教学中，老师应充分发挥引路人的作用，把学生作为教学互动的主体，使之制定一系列学习计划，打破某种习得性无助。让每个学生都准备自己的职业生涯规划，然后进行小组讨论，在讨论中阐述自己所制定的计划及理由，为既定的目标而努力，从而逐渐增强希望的体验。这种自己逐渐掌控希望的过程会随着次数与频率的增多而沉淀，使希望这种品质成为一种心理特质。

4．*顽强韧性的开发*。顽强韧性的开发在某种意义上可以认为是一种抗挫折的心理品质，从大学生群体中可以看出，现代大学生的坚韧自强这种心理资本较低。有资料显示，近几年大学生的自杀率在逐渐升高，有的是因为情感问题，有的是因为学业问题。无论是什么原因，究其根本还是心理素质低，情感脆弱。就目前状况看来，积极塑造大学生的坚韧自强的心理品质势在必行。挫折是不可能避免的，每个人的人生中都会遇到或多或少的挫折，其强度也不尽相同，如何应对这些挫折并顺利地度过是建立顽强坚韧心理品质的重点。每个人都不是孤立的个体，尤其是在情感上，一但个体受到任何挫折、委屈，都想会找朋友倾诉或者是寻求帮助。因此，针对大学生，应该建立良好的社会支持体系，这种支持系统可以来自家庭、学校甚至是社会。学校的辅导员老师应该时刻关注着学生的动态，发现他们在思想上有任何波动都应该及时给予帮助。周围的同学关系也是良好的支持系统之一。学校开设的心理咨询室，应该成为学生心灵倾诉的港湾、答疑解惑的智慧库。

总之，针对大学生群体心理资本的培养还需要更多的理论支撑，并且大学生心理资本的因素维度也需要不断地拓展补充，本文仅对最经典的四因素提出若干建议，旨在为心理教育实践提供借鉴。但可以肯定的是，大学生的心理资本的开发已经迫在眉睫。

参 考 文 献

[1] 仲理峰. 心理资本研究评述与展望［J］. 心理科学进展，2007，(3)：482－487.

[2] Luthans F. The need for and meaning of positive organizational behavior［J］. Journal of Organizational Behavior，2002，23(6)：695－706.

[3] Luthans F. Positive organizational behavior：Developing and managing psychological strengths［J］. Academy of Management Executive，2002，16(1)：57－72.

[4] Luthans F，Luthans K W，Luthans B C. Positive psychological capital：Beyond human and social Business Horizons，2004，(47)：45－50.

[5] Luthans F，Avolio B J，Walumbwa F O，and Li W. The psychological capital of Chinese workers：Exploring the relationship with performance［J］. Management and Organization Review，2005，(1)：247－269.

[6] Luthans F，Youssef C M，Avolio B J. Psychological capital：Developing the human competitive edge［M］. Oxford，UK：Oxford University Press，2007：5.

[7] Larson M D，Luthans F. Potential added value of psychological capital in predicting work attitudes［J］. Journal of Leadership & Organizational Studies，2006，13(2)：75－92.

[8] Luthans F，Youssef C M，Avolio B J. Psychological capital：Developing the human capital edge［M］. Oxford，UK：Oxford University Press，2007.

[9] 张阔，张赛，董颖红. 积极心理资本：测量及其与心理健康的关系［J］. 心理与行为研究，2010，8(1)：58－64.

[10] 惠青山，凌文辁. 中国职工心理资本内容结构及其与态度行为变量关系实证研究［A］. 张文新. 第十二届全国心理学学术大会论文摘要集［C］. 济南，2009：329.

[11] 魏红权，冯江平. 企业员工心理资本、工作满意感、组织支持感与关系绩效关系的实证研究［A］. 张文新. 第十二届全国心理学学术大会论文摘要集［C］. 济南，2009：330.

[12] 田喜洲，谢晋宇. 心理资本对接待业员工工作态度与行为的影响效应与机理［J］. 软科学，2010，24(5).

[13] 滕少霞. 心理资本、组织气候与员工工作态度关系的实证研究［J］. 统计与决策，2010，(4)：88－90.

[14] 方必基. 青少年学生心理资本结构、特点、相关因素及团体干预研究［D］. 福州：福建师范大学，2012.

[15] 王雁飞，朱瑜. 心理资本理论与相关研究进展［J］. 外国经济与管理，2007，29(5).

[16] 蒋苏芹. 大学生心理资本的内涵与结构研究［D］. 南昌：南昌大学，2010.

[17] Hosen R，Solovey－Hosen D，Stern L. Education and capital development：Capital as durable personal，social，economic and political influences on the happiness of individuals［J］. Education，2003，123(3)：496－513.

[18] Fred Luthans，Carolyn M Youssef，Bruce J Avolio. 心理资本［M］. 李超平，译. 北京：中国轻工业出版社，2008，(1).

〔责任编辑：崔家善〕

(C)1994-2023 China Academic Journal Electronic Publishing House. All rights reserved. http://www.cnki.net
