# Supplementary material for: A study on the current status and influencing factors of psychological capital among medical undergraduates
Source: Front Psychol. 2026 Oct 30;17:1830730. doi: 10.3389/fpsyg.2026.1830730 (PMC13759799; doi:10.3389/fpsyg.2026.1830730)
Supplement: Supplementary file 1 [file Data_Sheet_1.ZIP › References PDF/Cultivation of Psychological Capital of College Students in Post-pandemic Era.pdf]

后疫情时代大学生心理资本培养

张文芳　同雪莉

(西北大学，陕西　西安　710127)

【摘　要】后疫情时代，是对疫情持续应对、反思和调整的时期。心理资本是大学生疫情下心理应激反应的有效中介变量，对高校心理健康教育工作来说，有助于降低重大疫情对大学生心理健康的影响、促进大学生自我成长、推动高校心理育人模式科学化。重大疫情对我国大学生心理资本的冲击有利有弊，政府的有效防控、民众的齐心努力和居家亲情陪伴等正性的环境和支持资源，有利于心理资本的乐观和希望因子生长；同时，重大疫情对于应激经验少、人格基础弱、心理压力大、社会支持少的大学生，对其自我效能和心理韧性都呈现出消极影响；因此，高校可以通过推动“三全”育人、加强教育和推动实践3个途径，从营造乐观希望的环境、提升效能感和增强韧性3个方面，提升大学生心理资本。

【关键词】后疫情时代；大学生；心理资本

【中图分类号】G444　　【文献标志码】A　　【文章编号】1003-0166(2020)11-0088-05

doi:10.3969/j.issn.1003-0166.2020.11.017

心理资本指个体在成长和发展过程中表现出来的一种正向积极心理状态[1]，包括：自我效能感，即能够接受挑战性的任务并努力取得成功；乐观，即对现在或未来的成功进行积极导向的归因；希望，即能够坚持目标，在必要时候也能调整或重新选择目标；韧性，即能够持之以恒，遇到问题或困境时能够“复原”并能采取迂回的办法取得成功[2]。心理资本被重视始于经济学领域，学者们关注到，自我知觉、人生信念等一些能够反映人的自尊感的、稳定的个体特质，能够影响个体的动机和态度，进而影响劳动生产率[3]，之后，心理资本逐渐成为热点议题，被广泛应用到企业管理、人才培养等领域。Luthans 等[4]认为，个体拥有的人力资源分为：人力资本、社会资本和心理资本，人力资本关注个体的知识技能，社会资本关注个体的外部关系网络，而心理资本则关注个体是否具备积极的心理特质和状态，并认为心理资本是可以被测量和开发的。

随着我国疫情趋于稳定，各行业有序复工、高校分批复学，中国正式进入了“后疫情时代”。具体什么是“后疫情时代”，没有统一的说法。综合专家观点，笔者认为“后疫情时代”有3个特点：一是疫情爆发之后渐趋平稳的时期；二是疫情并未完全消失，有可能小规模或季节性的爆发，也有可能迁延延长很长一段时间；三是疫情对各方面产生的影响是深远的，我们无法将一切都恢复如前，但也因此产生革新和嬗变。之所以给“后疫情”冠以“时代”的

收稿日期：2020-06-22

基金项目：陕西省社科界重大理论与现实问题研究“新时代高校学生心理困境与抗逆力提升研究”(2019Z135)；西北大学2019年校级学生思想政治教育工作研究专项重点课题“大学生心理资本提升路径研究——以西北大学为例”(xg2020002)

作者简介：张文芳　西北大学心理健康教育中心副主任，讲师，研究方向：心理健康教育

同雪莉　西北大学马克思主义学院教授，研究方向：社会心理学

(C)1994-2022 China Academic Journal Electronic Publishing House. All rights reserved.　http://www.cnki.net

词汇，是因为在疫情得到基本控制的这个节点之后，人们需要很长的一段时间，去持续应对和不断地反思疫情带来的影响和挑战。这种应对和反思，除了疫情防控常态化，更重要的是思维方式、生活习惯、心理应对能力等各方面的调整或增强。

在“后疫情”时期，各个行业都在积极探讨如何“适应”或“转型”，高校心理健康教育工作也不例外。新冠肺炎疫情给我国民众带来了多方面的影响和冲击，也给高校心理工作带来了诸多挑战。研究发现，大学生常常在一些大型的突发事件面前缺乏应对能力，进而在个人和群体层面产生巨大心理压力，给高校的学生工作乃至教学工作带来严峻的障碍[5]；因此，对于高校心理健康教育工作来说，研究如何加强预前教育和心理资本培养，增强大学生面对重大疫情等突发事件的应对能力，成为了一项具有重要价值的议题。

1 后疫情时代大学生心理资本培养的意义

作为继财力、人力和社会资本之后的“第四大资本”[6]，心理资本对于大学生的重要性毋庸置疑，对于当今高校而言，大学生面临的学业、职业、心理、人际等压力和随之产生的问题愈来愈凸显并且会持续很长一段时间仍然存在，使得积极心理学教育和前置性心理干预本身就是一个有意义的研究课题。大学生作为高级人才，被赋予“时代新人”的历史责任；因此，培养和提升大学生心理资本，帮助大学生在重大疫情等突发事件下，成功应对逆境、充分发挥潜力，实现正向成长、发挥有力担当，更是高校在后疫情时代的一项重要任务。笔者从以下 3 个方面论述其重要性。

1.1 降低重大疫情等突发事件对于大学生心理健康的不良影响

1 月 30 日，世界卫生组织宣布新型冠状病毒疫情为“国际关注的突发公共卫生事件”。突发公共卫生事件(emergent events of public health)指突然发生，造成或者可能造成社会公众健康严重损害的重大传染病疫情、群体性不明原因疾病、重大食物和职业中毒以及其他严重影响公众健康的事件[7]。突发事件发生时，因疫情、疾病、生活、工作以及社会和人际关系等因素而致的情绪和心理问题非常突出[8]。就高校大学生而言，由于生理心理不成熟、大学生活的特殊性、缺乏应对突发事件的能力等主观性原因[9]，以及重大疫情下的一些居家隔离、全面封闭或者无法与外界联系的客观出现的特殊措施，大学生在面对突发公共卫生事件时，易出现紧张、焦虑、恐惧、悲观、烦躁、抑郁等不良心理反应[10]，处于较高的应激水平[11]。研究发现，个体在高应激状态下，如果缺乏良好的应对方式，则心理问题发生率可达 43.3%，是普通人群的 2 倍[12]。

美国认知心理学家托尔曼(E.C.Tolman)在 1932 年提出了“S–O–R”应激反应模型，认为不同个体面对应激源S，产生的身心反应 R 不尽相同，这是由于个体的心理中介变量 O 不同影响的。这里的心理中介变量就是指个体的认知、应对方式、人格特征等与心理品质相关的因素。

个人认知、应对方式与人格特征都属于心理资本的范畴，是可以通过改变环境和针对性培养而改变的。刘立伟等认为疫情背景下帮助并引导大学生在心理层面实现正向成长非常重要[13]，如果能在高校心理健康教育体系中预先加入心理资本培养，促使大学生形成一种正向积极的心理状态，产生积极的自我认知、应对方式，拥有乐观、坚持等心理韧性品质，那么，面临重大疫情等突发性事件时，大学生的心理应激反应和心理健康状态更可能趋于正向和积极。

1.2 促进大学生全面发展，发挥大学生自我成长和服务社会的正能量

大学生是高校为国家和社会培养的综合性人才，不仅作为新技术、新思想的代表，还是在道德素养、专业文化知识、身心素质方面综合发展并具有代表性的优秀群体，是推动社会进步的主力军；因此，仅仅把大学生心理健康教育的目的定位于心理援助和心理干预是不科学的，优化大学生心理素质，充分发挥大学生在特殊时期自我调适、主动成长，爱己及人、服务社会的正能量，才是高校心理健康教育的主方向。

党的十八大以来，习近平总书记多次在给大学生和高校的回信与讲话中，勉励大学生为国家和社会贡献自己的智慧和力量。2017 年 12 月，中共教育部党组发布《高校思想政治工作质量提升工程实施纲要》，将“心理育人”纳入高校“十大”育人体系。高校大学生要承担起完善自我、服务社会的伟大重任，具有良好的心理素养和心理资本是必要条件。

不可忽视的是，当前时期，在各种现实压力、成长环境和家庭支持系统等影响下，随着越来越多的大学生因心理问题厌学、弃学，甚至自杀、凶杀等事件不时见诸媒体，大学生心理健康问题已逐渐成为社会关注的焦点。就笔者所在高校的咨询情况来看，心理咨询学生总数逐年递增，其中发展性的问题居多，占 87%，表现为：情绪控制、自我认知、人格发展等不完善造成的环境适应、学习调适、人际交往、恋爱等方面出现心理与行为的偏差。这给我们传递的信息有两点，一是亟需提升当前大学生的心理健康水平；二是大学生心理问题集中于发展性成长性需求，他们对于增强自我认知、人格发展、应对能力等相关能力的需求较高，这与心理资本包含的内容是一致

(C)1994-2022 China Academic Journal Electronic Publishing House. All rights reserved. http://www.cnki.net

的。可以说,大学生心理需求大部分是对于心理资本提升的迫切需求。

1.3 推动高校心理育人模式科学化转变

随着国家对高校心理育人工作的不断重视,大部分高校形成了“实践活动、课程教学、咨询服务、危机干预”四位一体的工作格局,但仍然存在“重干预轻预防、重帮扶轻自助”的问题。心理健康教育应当是一个从积极心理学角度出发的前置性教育,以提升学生心理资本,培养完善人格,提高个体心理素质和生活质量,最终实现自我价值为目的[14],而这项工作在更早的年龄段就应该开始。按照我国的现状,大学之前的中小学心理健康教育仅局限于一些零散的讲座宣传、团辅活动等,有些中小学甚至没有。随着一些带着心理问题和危机隐患的学生考入大学,使得高校的心理工作重点不得不放到危机识别干预和心理帮扶等问题应对型心理服务工作中来,没有太多精力在前置性积极心理学教育方面多做努力。在心理健康教育、心理资本培养方面的工作多诉诸于活动、讲座等形式,缺乏体系。

作为“十大育人体系”之一,心理育人是新时代高校心理健康教育的新任务、新使命[15]。“心理育人”的概念里,“心理”是路径、方式,“育人”是目的,与传统意义上的“心理健康教育”不同的是,心理育人是通过心理健康教育来实现育人的目的,凸显和强调了“育人”这个大目标。那么,“育”什么样的“人”? 党的十九大报告指出要“培养担当民族复兴大任的时代新人”。这就要求,高校心理健康教育要改变原有的、停留于问题干预和心理健康层面的工作思路,而应该着眼于“育人”这个大目标,把工作目标从“育心”转向“育人”,为国家和社会培养具有良好的心理健康素养,自尊自信、理性平和的社会心态的社会主义建设者和接班人[16]。要达到这个目标,就要不断提升大学生的心理资本,从而充分发挥心理潜能、积极应对挫折困境、全面实现个人价值。

可以说,加强心理资本这个生长性因子的培养,是发挥大学生作为“时代新人”在重大疫情等突发公共卫生事件中的积极应对、敢于担当、有所作为的作用,实现高校心理育人目标、科学化心理育人模式的重要途径。

2 重大疫情对大学生心理资本的冲击

所有的事件对个体产生的影响都可以一分两面来看,重大疫情等突发事件对于大学生心理资本带来的冲击有“危”有“机”。下面,以此次新冠肺炎为例,来探讨其对于大学生心理资本的积极和消极影响。

1)对于乐观、希望两因素的积极影响。

组织支持感是心理资本的前因变量[17]24−38,组织通过满足成员的情感和现实求、重视成员的贡献和关心他们的幸福的程度,能够提高成员的积极情绪,影响到成员的心理资本 。Eisenberger 等认为,成员感受到组织给予的支持越多,其情绪也越乐观[18] 。Scheier 等则认为,在人们面临问题需要解决的时候,而也有潜在解决可能性的时候 ,表现得更加乐观[19]。新冠肺炎爆发以来,我国政府的有效防控和民众的齐心努力,使得我国在疫情防控中成效突出,也进一步增强了社会成员的民族认同感、自豪感和自信心,增强民族凝聚力[20],为大学生形成心理资本等积极心理品质提供了环境和组织支持感。

对于大学生心理资本的前因变量来说,虽然客观性的环境变得危险因素增多、社交范围变窄等,但是主观性、人文性的环境变得有营养、有动力,无论从家庭成员之间、社会支持之间、国家民族自豪感都有很多正能量、激励人心的积极性因子。从国家层面展现出的全力抗疫、关注民生的情怀和强大高效的公共卫生体系,从社会层面呈现出的齐心抗疫、大爱无私的氛围和温暖感人的社会互助支持,让很多人,尤其是个性突出、爱憎分明的大学生,更多地感受到了被支持、被激励的力量和强烈的家国情怀。并且,在居家抗疫期间,大部分学生能够更多地享受亲情陪伴、放下压力、感受生活,体味人生的多面性和意义。这些正性的环境和支持资源在这个出现信仰危机[21]的时代,为大学生注入了新的力量、期待和希望,使得大学生有时间、有契机去自我觉察、思考成长,形成积极向上的自我认知、世界观和价值观,增强与心理资本相关的乐观、希望等因素。

2)对于效能感、心理韧性的消极影响。

自我效能感是班杜拉于 20 世纪 70 年代提出的,他认为自我效能感是个体对于自己能否有能力完成某一行为所进行的推测与判断,也就是个体对自身能力的一种自信程度。自我效能感受到个体的成败经验、他人的替代经验、言语劝说、情绪唤醒、情境条件等影响,笔者把它们归纳为 4 点,一是是否有成功经历或他人经验;二是是否有良好支持系统;三是个体心理特质和情绪反应特点;四是所面临事件的严重程度。应用到疫情中来,我们可以认为,个体的抗疫和抗逆经历、家庭支持、社会援助、个体的情绪认知等特质、疫情严重程度等因素,都能够影响到一个人的自我效能感的高低。此次新冠肺炎疫情以其突发、紧急、传染性强、涉及面广、危害程度高、对人们生活工作影响巨大且深远为特点;大学生社会阅历少、生活经验不足、情绪易波动等群体特点,使得大学生缺乏应对突发疫情的认知基础和经验能力;一些本来就有心理困扰或隐患的学生,在家庭关怀和人际支持等方面也本有缺失等这些因素都会对大学生整体自我效能感有所冲击,导致大学生在疫情期间的焦虑和抑郁情绪总体上高于其他群

(C)1994-2022 China Academic Journal Electronic Publishing House. All rights reserved. http://www.cnki.net

体[22]。同时,焦虑和抑郁情绪都会使个体出现负性认知、情绪低落、意志活动减退等反应,这些反应又反过来降低个体的自我效能感。

自我强化也是心理资本的前因变量[17]24-38,影响到心理资本的生成。自我强化主要指的是个体积极导向的不断激励、暗示自己的动机和行为。Greenwald 研究认为,如果个体能够不断地自我强化,就会表现出更高的弹性[23],这种弹性是指个体从逆境、困境中复原、回复的能力,也叫心理韧性。新冠肺炎疫情于很多人是一个很大的负性应激事件,尤其是对于原本就有心理健康问题或创伤经历的学生[24],假期延长、生活单调、计划搁置、娱乐受限、人际单一、学就业压力等客观原因,以及疫情信息过载、人格基础脆弱、应对能力不足、家庭支持薄弱等主观原因,都有可能致使大学生自我调节能力变差、自我强化功能变弱,出现抑郁失落、紧张无助等负性应激反应,影响大学生从重大疫情冲击的逆境中快速回复的心理韧性。

因此我们可以发现,新冠肺炎疫情对于原本应激经验少、人格基础弱、心理压力大、社会支持少的大学生,呈现出了负性冲击和消极影响,使其自我认知怀疑、自我效能感弱、心理韧性降低,也意味着,这部分大学生的心理资本是随之受损的。

3 后疫情时代大学生心理资本提升路径

3.1 “三全”育人,营造支持性环境

研究发现,组织氛围、领导风格等作为企业员工心理资本的前因变量,能够促使企业员工产生高水平的心理资本[25]。由此,我们可以推论,针对于大学生心理资本而言,我们优化学校氛围(如人文环境、校风、班风、舍风等)、教学和管理风格(如思政教育模式、课程教学机制、老师教育方式、管理服务模式等)、学习任务的内容和挑战性(难度、形式、成就感等)等,将有助于帮助大学生形成积极向上的学习生活状态和价值观,提高大学生应对学习、就业、人际压力以及突发事件冲击等逆境的能力,发展个体潜力,提升心理资本。

后疫情时代,缓解疫情应激反应、提供心理援助服务的工作需求逐渐减少,疫情带给教育的挑战和机遇、思考与变革才刚刚开始。高校更应聚焦于从积极心理学的视野,为大学生营造出全员、全程、全方位的心理育人氛围,形成促进心理资本生成和提升的支持性环境。“全员”是指高校心理育人主体应该包括与教育和育人相关的所有人员,即心理教师、学工干部、教师、导师、班主任、管理人员、教辅人员、后勤服务人员、安全保卫人员等等,都能够秉持积极的、正向的、支持性的育人理念,培养大学生乐观、希望等正性情绪;“全程”指从新生到毕业生,根据不同阶段的心理需求特点和面临的学习发展任务不同,针对性地进行教育、引导、支持,增强大学生的知识和能力,提高自我认知和自我效能感;“全方位”是指积极的心理育人理念应该贯穿、渗透到学生的课堂教学、实践活动、科学研究、文化生活、贫困资助、就业考研等环节中去,使学生在各方面接受和感触到一以贯之的积极支持和正向引导,通过文化育人、教书育人、管理育人,促使掌握知识、锻炼技能、磨练意志、养成乐观积极品质,提高自我效能感。

3.2 加强教育,提高心理素质

心理素质是在先天遗传基础上,经过后天环境与教育的影响,经过主体实践训练所形成的性格品质与心理能力的综合体现。这里的心理能力是指认知能力、心理适应能力和内在动力。对于应对重大疫情来说,心理素质就是个体关于此类重大应激事件相关的心理知识和应对能力。不同心理素质的人对于相同的突发事件,会产生不同的应激反应。有研究发现,此次新冠肺炎疫情中,临床专业学生焦虑、抑郁得分较低,非临床专业学生无助及焦虑抑郁情绪较重[26],这与大学生的专业思维方式、相关知识储备等诸多影响因素有关[27-28],由于非医学专业学生病毒知识缺乏,对“未知风险”与“忧虑性风险”关注较多[26],易产生非理性恐慌、形成焦虑抑郁反应;而医学专业的学生的专业教育、职业培育等都与健康教育、心理卫生、生命等相关,有研究证实系统的健康教育可以促进大学生形成健康的心理素质,较好地应对和适应生活应激[29]。

高校在日常教育体系中,加入心理卫生、健康教育、疾控常识、病毒知识、积极应对方法等教育普及,就能够矫正认识误区、避免恐慌情绪,帮助大学生形成科学理性的心理认知,从而采取正确有效的应对方式,培养大学生能够进行积极归因的乐观因子、可以坚持并灵活调整目标的乐观因子和敢于应对挑战性任务的自我效能感,全面提升心理资本。

3.3 推动实践,增强心理韧性

实践活动有助于提高大学生的人际沟通、团队协作、抗挫耐压、组织管理、开拓创新等方面的能力,有助于增强大学生的自信心、人际支持、自我效能感等积极体验和实践经验,使得大学生在面临到困境和突发状况时,能够乐观面对、有效应对、不断探索和寻求支持,在面对逆境时,呈现出积极乐观、不怕困难、平和理性的心理韧性。

高校可以从 3 个方面丰富完善教育活动体系,一是专业支持。发挥心理健康教育教师、心理咨询师的主导作用,开展心理成长小组、团体心理辅导、大学生心理骨干成长营等活动,将专业心理知识与学生成长需求紧密融合,让有自我成长需求、有爱己助人意愿的大学生,能够在专业心理知识和老师的带领下,注入心理能量、促进学

(C)1994-2022 China Academic Journal Electronic Publishing House. All rights reserved. http://www.cnki.net

生成长，在增强自身心理健康水平和心理资本的同时，影响和辐射到身边人群。二是氛围引导。发挥辅导员、班主任、导师等群体的引领作用，开展心理主题班会、院系特色心理活动、心理知识大赛、心理作品大赛、心理情景剧展演等活动，将心理成长与专业特点和学生需求结合起来，营造心理氛围，用文化育人引领学生心理成长。三是朋辈互助。发挥学生主体作用，充分调动学生主动性和积极性，开展心理读书沙龙、心理志愿服务、朋辈心理互助等活动，促进学生自助、互助，在实践中反思、在关系中成长。□

参考文献

[1]Fred Luthans,James Bavay,Brucee J Avolio,et al.Positive psychological capital:Measurement and relationship with performance and satisfaction [J].Personnel Psychology, 2007,60(3):541-572.

[2]Luthans F,Youssef CM,Bruce JA.心理资本[M].李超平，译.北京：中国轻工业出版社，2008.

[3]Goldsmith A H, Veum J R,Darity W. The impact of psychological and human capital on wages[J].Ecnomic Inquiry,1997(35):815-829.

[4]Luthans F,Luthans K W,Luthans B C. Positive psychological capital:Beyond human and social capital[J].Business horizons,2004,47(1):45-50.

[5]梅松丽，于佳鑫，何博武，佟倩.高校突发公共卫生事件的心理应对分析[J].医学与社会，2011,24(05):87-89.

[6]曾晓娟，阎晓军.国内外心理资本研究热点与发展趋势比较分析:2004-2017 [J]. 齐鲁师范学院学报，2019,34(03):82-90.

[7]中华人民共和国国务院.突发公共卫生事件应急条例(国令第376号)[Z].2003-05-09.

[8]王一牛，罗跃嘉.突发公共卫生事件下心境障碍的特点与应对[J].心理科学进展，2003(04):387-392.

[9]梅松丽，于佳鑫，何博武，佟倩.高校突发公共卫生事件的心理应对分析[J].医学与社会，2011,24(05):87-89.

[10]孟惠平，栾天宇，杨延哲.突发事件对大学生心理和生理影响的调查[J].吉林师范大学学报：自然科学版，2005(04):42-44.

[11]文庠，杜文东，王挺，任君红，沈永健.非典期间大学生心态及行为的调查报告[J].南京中医药大学学报：社会科学版，2003(02):121-124.

[12]曾荣侠.大学新生应付方式和抑郁情绪的相关研究[J].健康心理学杂志，2003,11(3):233-235.

[13]刘立伟，张瑾.新冠肺炎疫情下高校大学生的正向成长研究[J].华北理工大学学报：社会科学版，2020,20(03):23-27.

[14]同雪莉，徐茜.学校心理教育发展之走向:嵌入·融合·共 生[J].未来与发展，2017(6):86-90.

[15]马建青，杨肖.心理育人的内涵、功能与实施[J].思想理论教育，2018(09):87-90.

[16]丁闽江.辅导员在心理育人中的角色定位及工作方略：基于406封大学生求助信的思考[J].高校辅导员，2020(02):73-76.

[17]许萍.心理资本:概念、测量及其研究进展[J].经济问题，2010(02).

[18]Eisenberger R, Cummings J, Armeli S .Perceived Organizational Suppoa and Discretionary Treatment, and Job Satisfaction [J]. Journal of Applied Psychology,1997(82).

[19]Scheier M E, Weintraub J K, Carver C S .Coping With Stress:Divergent Strategies of Optimists and Pessimists [J]. Journal of Personality and Social Psychology, 1986(51).

[20]付洪，刘宇洋.在应对突发事件中增强民族凝聚力[J].未来与发展，2020(05):39-43+57.

[21]高晓娟，张军."90"后大学生马克思主义信仰危机与对策：以山西省四所高校为例[J].忻州师范学院学报，2019,35(04):71-74.

[22]边慧冕，潘婷，赵孟.新型冠状病毒肺炎疫情早期大学生情绪分析[J].中国学校卫生，2020,41(05):668-670.

[23]Greenwald A G.The Totalitarian Ego:Fabrication and Revision of Personal History [J]. AmeriCan Psychologist, 1980(35).

[24]刘湘玲.高职大学生疫情期间与入学时的心理健康状况比较[J/OL].中国健康心理学. http://kns.cnki.net/kcms/detail/11.5257.R.20200516.1451.002.html.

[25]蒋陆军.心理资本理论研究述评与展望[J].现代管理科学，2019(03):109-111.

[26]王轲，王琪，刘碧波，贾娇，任徽，陈明伟.新型冠状病毒肺炎疫情对某高校不同专业大学生心理影响[J/OL].西安交通大学学报(医学版).http://kns.cnki.net/kcms/detail/61.1399.R.20200526.1036.002.html.

[27]雷寰宇. 思维方式在医学院校文理科大学生中的差异[J].山西高等学校社会科学学报，2018,30(12):61-64.

[28]路翠萍.大学生思维方式与应对方式的关系研究[J]. 心理发展与教育，2008(4):56-60.

[29]Wang l m, wang q, wang y, etal. Study on the effect of health education on college students´ mental health [J]. China journal of health psychology,2005(2):107-110.

（下转第87页）

(C)1994-2022 China Academic Journal Electronic Publishing House. All rights reserved. http://www.cnki.net

Research on the Construction of Master´s Degree Training System in Medical Education

Zhang Ting , Wang Xiaofei

(Center of Medical Education ,Jinzhou Medical University ,Jinzhou Liaoning 121000, China)

Abstract: In view of the actual needs of medical development for medical education talents and the importance of training medical professionals in the new era, we are based on the characteristics of medical education, through the construction of theoretical curriculum system, education and teaching practice curriculum development, academic exchange activities design and graduate students. We will train and develop a series of measures, and gradually carry out scientific and systematic construction of the medical education master´s postgraduate training system, which provides institutional guarantee for the cultivation of high -level talents in medical education, and at the same time clarifies the future development direction.

Key words: medical education , master´s degree training , system construction

CLC number: R-012 Document code: A Article ID:1003-0166(2020)11-0083-05

doi:10.3969/j.issn.1003-0166.2020.11.016

(上接第 92 页)

Cultivation of Psychological Capital of College Students in Post-pandemic Era

Zhang Wenfang , Tong Xueli

(Northwest University, Xi´an Shaanxi 710127, China)

Abstract: Inpost-pandemic era, it will be a long time of continued to combat 、reflect and adapt. Psychological capital is an effective mediating variable of psychological stress response for college students.For mental health education in universities, psychological capital can help students to reduce the impact of epidemic disease on mental health and promote their self -development; And also, enhancing students´ psychological capital could improve the mode of psychological education in universities. For the psychological capital of college students in China, the epidemic situation not only has a negative impact, but also has a positive influence. On the one hand, college students´ self-efficacy and psychological resilience will be weakened who has less stress experience, weak personality base, high psychological pressure and less social support. On the other hand, positive environment and supporting resources are beneficial to´optimism and hope´ factors increase in psychological capital, such as the government´s effective prevention and control, people's concerted efforts and family companionship. Therefore, to enhance the psychological capital of college students could work on three aspects: creating an optimistic and hopeful environment, enhancing efficacy and enhancing resilience; and also in three ways: educate people include 'full staff, whole process, all directions´, strengthen education and promote practice.

Key words:post-pandemic era, college student, psychological capital

CLC number: G444 Document code: A Article ID:1003-0166(2020)11-0088-05

doi:10.3969/j.issn.1003-0166.2020.11.017

(C)1994-2022 China Academic Journal Electronic Publishing House. All rights reserved. http://www.cnki.net
