# Supplementary material for: A study on the current status and influencing factors of psychological capital among medical undergraduates
Source: Front Psychol. 2026 Oct 30;17:1830730. doi: 10.3389/fpsyg.2026.1830730 (PMC13759799; doi:10.3389/fpsyg.2026.1830730)
Supplement: Supplementary file 1 [file Data_Sheet_1.ZIP › References PDF/Current employment pressure among medical students and its relationship with psychological capital.pdf]

●院校管理

医学生就业压力现状及其与心理资本的关系

王 平[1,2],吉 峰[2*]

(1. 济宁医学院精神卫生学院; 2. 山东省高校行为与健康人文社科基地,山东 济宁 272067)

[摘要] 目的: 了解医学院校大学生就业压力现状,探讨就业压力与心理资本的关系。方法: 采用大学生就业压力问卷和积极心理资本量表,对 248 名医学专业本科生进行测量,结果: 就业压力项目平均分为 3,不同年级就业压力差异具有统计学意义($F = 3.475, P < 0.05$),心理资本与就业压力存在显著负相关($r = -0.195, P < 0.01$)。结论: 医学生就业压力处于中等水平,各年级学生均存在不同程度的就业压力,关注学生的就业压力要大力开发其心理资本。

[关键词] 医学生; 心理资本; 就业压力 DOI: 10.3969/j.issn.1002-1701.2017.03.027

[中图分类号] G444,G647 [文献标识码] A [文章编号] 1002-1701(2017)03-0057-02

近年来,高校毕业生数连年攀升,大学生面临的就业压力与日俱增。大学生就业问题已成为政府、高校及其他相关部门着力研究和解决的重点。就业压力指个人在面临择业时所产生的压力,是在就业过程中个人主观因素与就业情景交互作用所产生的一种心理紧张现象[1]。以往文献对大学生就业压力的研究多集中于现状调研、原因分析,缓解对策等质性研究,实证研究多选择社会支持、应对方式及心理健康等因素,未见大学生就业压力与心理资本的相关关系及其预测性研究。

本研究以医学院校大学生就业择业背景下的就业压力为研究主题,采取实证研究的方法,通过医学生就业压力问卷和积极心理资本量表,多维度地了解当前医学生就业压力状况,探讨医学生就业压力与心理资本之间的深层次关系,以期使医学生及其教育者认识到培养和开发积极心理资本对降低和缓解就业压力的作用,为相关部门对医学生的就业辅导提供新的思路和科学依据。

一、对象与方法

(一) 研究对象。

随机抽取济宁医学院在校大一至大四本科生,共发放问卷 300 份,回收有效问卷 248 份,有效率为 83%。年龄范围为 17-25 岁,平均 22.4 岁。其中男生 101 人,女生 147 人。城镇生源 98 人,农村生源 150 人。

(二) 研究方法。

1. 工具。大学生就业压力问卷: 田圣会等人编制[2],利克特 5 点计分方法,得分越高表明压力越大。该问卷由七个因素组成,分别为能力个性压力、环境挑战压力、专业前景压力、就业资源压力、社会支持压力、职业期望压力、学业学历压力。问卷的 α 系数为 0.812,各个因素的 α 系数为 0.72-0.745,建构效度为 0.829。积极心理资本量表(Positive Psychological Capital Questionnaire,PPQ): 张阔等人在 2008 年编制[3],通过总分以及各因子分来反映心理资本的状况,得分越高表明积极心理资本越丰富。该问卷有 26 个项目,4 个因子分别为自我效能、希望、乐观、韧性。问卷具有良好的结构效度,平均因子负荷达到 0.64,总体 α 系数 0.90。

2. 施测程序。选择晚上自习时间,在自习室内团体施测。参加测试的学生都可以得到一份小礼物,以此激励学生积极参与并表示感谢。要求参与者认真完成两份问卷。由心理学专业教师担任主试,统一指导语,在规定时间内作答,当场回收问卷。

3. 统计方法。调查结束后,对有效问卷进行统一编码,建立数据库,使用 SPSS 18.0 统计软件对数据进行描述、F 检验、相关、回归等统计分析。

二、结 果

(一) 大学生就业压力的现状。

计算出医学生就业压力问卷项目的平均分为 3 分,采用理论中位数 3 来划分压力的高低。总体项目平均分是 3,可以看出医学生就业压力处于中等水平。对就业压力的各因子的平均分进行排序,由高到低依次是职业期望压力、环境挑战压力、学业学历压力、就业资源压力、职业期望压力、专业前景压力、社会支持压力、能力个性压力(见表 1)。

表 1 大学生就业压力的描述性统计结果

	项目数	总体平均分	项目平均分	总体标准差
就业压力	38	113.85	3.00	19.00
能力个性压力	7	19.67	2.84	4.43
环境挑战压力	7	22.89	3.27	3.97
专业前景压力	5	14.98	3.00	3.42
就业资源压力	5	15.10	3.02	3.31
社会支持压力	4	11.87	2.97	2.55
职业期望压力	3	35.46	3.41	5.24

从不同人口学变量角度检验医学生就业压力的差异,结果显示不同性别和不同生源地医学生就业压力差异无统

* 通讯作者

中国知网 https://www.cnki.net

计学意义。大一至大四各年级学生就业压力差异性检验，发现年级间存在显著性差异（$F=3.475$，$P<0.05$）。进一步的多重比较显示，大一（107.75±16.00）与大二（115.75±20.35）、大一与大三（115.86±20.70）、大一与大四（125.42±15.24）、大二与大四、大三与大四年级就业压力差异存在统计学意义（$P<0.05$）。

（二）就业压力与心理资本的关系。

将大学生心理资本与就业压力进行相关分析（见表2）。大学生心理资本与就业压力存在显著的负相关（$r=-0.195$，$P<0.01$）。其中，心理资本的韧性因子与就业压力及其各因子存在显著的负相关（$P<0.01$）。

表2 心理资本与就业压力的相关分析

	就业压力	能力个性	环境挑战	专业前景	就业资源	社会支持	职业期望	学业学历
心理资本	-0.195**	-0.263**	0.043	-0.135*	-0.139*	-0.085	0.773**	-0.036
自我效能	-0.043	-0.087	0.042	-0.060	-0.042	0.003	0.548**	0.029
韧性	-0.343**	-0.394**	-0.174**	-0.190**	-0.185**	-0.214**	0.287**	-0.147*
希望	-0.144*	-0.208**	0.121	-0.105	-0.115	-0.058	0.684**	-0.047
乐观	-0.080	-0.136*	0.163**	-0.070	-0.099	0.007	0.952**	0.056

注：$**P<0.01$，$*P<0.05$

为了进一步探讨就业压力和心理资本的内在关系，以就业压力为因变量，心理资本及各因子为自变量进行逐步回归分析。结果显示，韧性进入了回归方程（$\beta=-0.336$，$P<0.01$），它对就业压力有显著的预测作用，能解释就业压力11.8%的变异。

三、讨 论

（一）医学生就业压力的特征。

本次调查表明，医学生的就业压力处于中等水平，在就业压力的七个维度中，职业期望压力的分数明显高于平均分，说明大学生对将来就业的期望值偏高。很多大学生反映“找工作不难，找个好工作很难。”这在一定程度上反映了医学生“高不成低不就”的状况，再加上家人、朋友对医学生的职位期望比较高，导致了医学生职业期望的压力较大。

大四学生的就业压力与其他年级有显著性差异。大四学生面临着毕业后立即就业，当前大学生的就业情形不容乐观，这种现实情况必然给大四的学生造成一定的压力。而大一到大三的学生暂时不会面临就业的问题。但是无论是大一还是大四都会面临就业压力，只是程度不同而已，而且随着毕业的临近就业压力会逐渐增大。而大二、大三学生可以有考研、考公务员、考事业编制等多种选择，所以就业压力方面差异不显著，与陈俊的研究结果一致[4]。

（二）医学生就业压力与心理资本的关系。

心理资本是美国著名学者Luthans[3]在2004年提出的一个概念，是指个体在成长和发展过程中表现出来的一种积极心理状态，能有效地测量和管理，投资与开发，是促进个体成长和提升绩效的核心心理资源。主要包括自信或自我效能感（Confidence or Self-efficacy）、希望（Hope）、乐观（Optimism）和坚韧性（Resilience）四个方面。本研究发现，大学生的心理资本与就业压力成显著的负相关关系，其中，韧性与就业压力总分及多个维度存在着显著的负相关。刘万军[5]的研究表明当前大学生的心理资本总体水平不高，在面对就业时，自我效能表现不高，自信心不足；就业受挫后情绪消极低落甚至放弃努力，缺乏韧性；不会主动推销自己，存在“等、靠、要”心理，希望有待提高[5]。

提升大学生就业水平，缓解就业压力应在学好专业知识的同时，积极开发和培育心理资本。因为心理资本不仅对学生的就业压力，乃至求职和就职后的工作绩效都有关键影响[6]。人力资本可以通过知识讲授的方式形成，而心理资本需要通过个体的体验式教育来积累。建议高等医学院校重视校园文化建设，创设实践导向的校园氛围，鼓励医学生勇于实践，善于实践，在各种校园活动和社会实践中，增加经历和见识，掌握有效的人际交往技巧，表现自我，增强自信。积极开展户外拓展训练，布置一些具有挑战性的任务，让学生面对困难，体验挫折，磨练其意志。大学生也要有意识地树立正确的就业观，锻炼良好的综合素质，培养乐观和希望的内在积极心理资本。

[参考文献]

[1] 刘春雷，马晓亮. 大学生就业压力研究的现状与展望[J]. 心理研究，2008，1(2)：12-15.
[2] 田圣会. 大学生就业压力实证研究[D]. 武汉：武汉大学，2010.
[3] 张 阔，张 赛，董颖红. 积极心理资本：测量及其心理健康的关系[J]. 心理与行为研究，2010，8(1)：58-64.
[4] 陈 俊. 大学生就业压力、应对方式与自我概念的关系研究[D]. 长春：东北师范大学，2008.
[5] 刘万军. 基于就业压力提高大学生心理资本的思考[J]. 中国成人教育，2011(16)：111-113.
[6] 柯江林，孙健敏，韩 瑛. 大学生心理资本对求职绩效的影响：积极求职行为的中介效应[J]. 心理学探新，2014，34(5)：468-473.

[收稿日期] 2016-02
[作者简介] 王 平，女，博士，讲师，研究方向：心理与行为测评，人力资源管理。
[基金项目] 山东省教育科学规划“教育招生考试科学研究专设课题”（编号：ZK1108282B001）。

中国知网 https://www.cnki.net
