# Supplementary material for: A study on the current status and influencing factors of psychological capital among medical undergraduates
Source: Front Psychol. 2026 Oct 30;17:1830730. doi: 10.3389/fpsyg.2026.1830730 (PMC13759799; doi:10.3389/fpsyg.2026.1830730)
Supplement: Supplementary file 1 [file Data_Sheet_1.ZIP › References PDF/Effect of COVID-19 Cognition on Professional Employment Intention in Medical Students.pdf]

·医学教育研究·

医学生的新冠肺炎疫情认知对专业就业意愿的影响

李俊龙[1]， 陈余梅[1]， 李心月[2]， 杜永红[1]， 陈俊国[1]， 李业[1]， 陈莎莎[1]

【摘要】 目的 探讨新冠肺炎疫情认知对医学生专业就业意愿的影响机制。**方法** 采用新冠肺炎疫情认知问卷、社会支持量表、心理资本量表和专业就业意愿问卷对 2 151 名医学生进行调查，采用 SPSS 19.0 与 AMOS 25.0 进行统计分析。**结果** 医学生新冠肺炎疫情认知与社会支持(r=0.187，P<0.01)、心理资本(r=0.417，P<0.01)、专业就业意愿(r=0.378，P<0.01)显著相关；新冠肺炎疫情认知可以正向预测医学生就业意愿(OR=3.931，95%CI=3.239～4.771)；社会支持(效应值=0.052，95%CI=0.027～0.088)、心理资本(效应值=0.438，95%CI=0.351～0.539)在新冠肺炎疫情认知与专业就业意愿间具有中介作用，且存在新冠肺炎疫情认知→社会支持→心理资本→专业就业意愿的链式中介效应(效应值=0.111，95%CI=0.070～0.157)。**结论** 新冠肺炎疫情认知不仅能直接影响医学生的专业就业意愿，还可通过社会支持与心理资本间接影响专业就业意愿。

【关键词】 新冠肺炎； 疫情认知； 就业意愿； 社会支持； 心理资本； 医学生

【中图分类号】 R183.3；G646 **【文献标志码】** A **DOI：10.3969/j.issn.1673-5625.2022.05.012**

Effect of COVID-19 Cognition on Professional Employment Intention in Medical Students

LI Junlong*, CHEN Yumei, LI Xinyue, DU Yonghong, CHEN Junguo, LI Ye, CHEN Shasha. **Sichuan Vocational College of Health and Rehabilitation, Zigong, Sichuan, 643000, China*

【Abstract】 Objective To explore how cognition of COVID-19 affected professional employment intention in medical students. **Methods** 2 151 medical students were investigated by COVID-19 cognition scale, social support scale, psychological capital scale and professional employment intention scale. SPSS 19.0 and AMOS 25.0 were used for statistical analysis. **Results** COVID-19 cognition was significantly correlated with social support(r=0.187, P<0.01), psychological capital(r=0.417, P<0.01) and professional employment intention(r=0.378, P<0.01). COVID-19 cognition could positively predict the medical students' professional employment intention(OR=3.931, 95%CI=3.239-4.771). Social support(effect value=0.052, 95%CI=0.027-0.088) and psychological capital(effect value=0.438, 95%CI=0.351-0.539) played an intermediary role in COVID-19 cognition and the professional employment intention, and there was a chain mediating effect COVID-19 cognition→social support→psychological capital→professional employment intention(effect value=0.111, 95%CI=0.070-0.157). **Conclusion** COVID-19 cognition not only directly affects the professional employment intention of medical students, but also indirectly affects the professional employment intention through social support and psychological capital.

【Key words】 COVID-19； Epidemic cognition； Employment intention； Social support； Psychological capital； Medical students

新冠肺炎重大疫情的暴发，对公众生命安全造成严重威胁，我国各地医务人员克服重重困难迅速投入疫情防控第一线，肩负起救治生命、守卫健康的重任。广大医务人员用生命和汗水守护人民群众生命安全，用实际行动生动诠释了“敬佑生命、救死扶伤、甘于奉献、大爱无疆”的崇高精神，受到全社会前所未有的关注、关怀与赞赏，同时也承担了常人无法想象的风险、压力与挑战，更有医务人员不幸为此付出生命。医学生作为未来的医务人员，在未成为正式医务人员前亲历与目睹了这场医学前辈与病毒的生死战役，可能会刺激他们弃医从事其他事业，也可能会进一步坚定其选择从事医学事业。这事关医疗卫生人才队伍健康可持续建设的大局，因此研究新冠肺炎疫情对医学生从事本专业就业意愿的影响效果及作用机制，探索建立坚定医学生专业承诺与从事本专业的就业意愿的举措，对提升医学生的专业素养、职业决策能力具有重要意义。基于此，本研究通过对四川省 4 所医学院校学生的抽样调查，探析医学生的新冠肺炎疫情认知对其从事医学专业就业意愿的影响机制，以期为医学院校的学生教育管理实践提供参考。

【基金项目】 四川省教育厅四川高校心理健康教育培训基地高校心理健康教育研究课题(2020SXJP024)；自贡市哲学社会科学一般规划课题(自社联〔2020〕10号)

【作者单位】 [1]四川卫生康复职业学院，四川 自贡，643000
[2]自贡市第四人民医院财务科，四川 自贡，643000
【通信作者】 杜永红，E-mail：593786101@qq.com

(C)1994-2022 China Academic Journal Electronic Publishing House. All rights reserved. http://www.cnki.net

1 对象与方法

1.1 研究对象

2020 年 3 月,采用方便抽样法,通过“问卷星”网络调研平台对四川省 4 所医学院校学生进行线上调研,回收数据 2 217 份,有效数据 2 151 份,有效率 97.02%,其中男生 287 人(13.34%);大一 1 079 人(50.16%),大二 514 人(23.90%),大三 395 人(18.36%),大四 163 人(7.58%);平均年龄(19.79±1.23)岁;独生子女 566 人(26.31%);学生会(社团)干部 608 人(28.27%);参与新冠肺炎疫情防控志愿工作 551 人(25.62%)。

1.2 研究工具

1.2.1 新冠肺炎疫情认知量表 该量表由 Lin 和 Lagoe[1]研发的疫情风险认知量表和 Ludolph 等[2]编制的疫情行为认知量表两个部分构成,包括个人风险认知、社会风险认知和疫情行为认知 3 个维度,共 14 个条目,采用李克特五级计分法,分值越高则表明对疫情的认知水平越高。本次研究中,量表 Cronbach's α 系数为 0.851,量表验证性因素分析的模型拟合指数为:CFI=0.951,TLI=0.935,GFI=0.948,AGFI=0.921,NFI=0.947,RMSEA=0.072,表明量表具有良好的信度与结构效度。

1.2.2 心理资本量表 采用张阔等[3]编制的积极心理资本量表,包含自我效能、韧性、希望与乐观 4 个维度,共 26 个条目,采用李克特七级计分法,反向条目计分相反,分值越高表明积极心理资本水平越好。本次研究中,量表 Cronbach's α 系数为 0.920,量表验证性因素分析的模型拟合指数为:CFI=0.920,TLI=0.907,GFI=0.875,AGFI=0.845,NFI=0.913,RMSEA=0.074,表明量表具有良好的信度和结构效度。

1.2.3 社会支持量表 采用肖水源[4]研制的社会支持量表,包含客观支持、主观支持以及对支持的利用度 3 个维度构成,共 10 个条目。根据研究对象为医学生的具体情况,本研究对量表作了适当调整。社会支持总分为各条目得分之和,分值越高则社会支持越高。本次研究中,量表 Cronbach's α 系数为 0.708,验证性因素分析的模型拟合指数为:CFI=0.930,TLI=0.890,GFI=0.977,AGFI=0.955,NFI=0.923,RMSEA=0.064,说明量表具有良好的信度和结构效度。

1.2.4 专业就业意愿问卷 采用自行编制的单维度专业就业意愿问卷,共 6 个条目,采用李克特五级计分法,从“非常不符合”到“非常符合”,分别赋值 1~5 分,均为正向计分,分值越高表明本专业就业意愿越强。本次研究中,量表的 Cronbach's α 系数为 0.917,验证性因素分析的模型拟合指数为:CFI=0.991,TLI=0.980,GFI=0.986,AGFI=0.957,NFI=0.990,RMSEA=0.075,说明问卷具有良好的信度和结构效度。

1.3 统计分析

本研究采用 SPSS 19.0 和 AMOS 25.0 进行数据处理分析,通过 Pearson 相关分析检验研究变量的相关性,采用 Logistic 回归分析新冠肺炎疫情认知对专业就业意愿的预测作用,通过 Bootstrap 分析检验社会支持与积极心理资本在其中的中介效应。以 $P<0.05$ 为差异具有统计学意义。

2 结果

2.1 共同方法偏差检验

采用 Harman 单因素法检验同源偏差情况,将问卷全部条目纳入未经旋转的因子分析模型,析出的第一个公共因素方差贡献率为 29.881%,低于 50%,表明本研究无明显的同源偏差。

2.2 新冠肺炎疫情认知、社会支持、心理资本与专业就业意愿的相关分析

对研究变量进行描述性统计和相关分析,结果显示,医学生的新冠肺炎疫情认知与心理资本、社会支持与专业就业意愿均成显著正相关($P<0.01$)。见表 1。

表 1 2 151 例医学生新冠肺炎疫情认知、社会支持、积极心理资本与专业就业意愿的相关分析

项目	均值	标准差	新冠肺炎疫情认知	心理资本	社会支持	专业就业意愿
新冠肺炎疫情认知	12.523	1.614	1.000			
心理资本	71.840	11.166	0.417**	1.000		
社会支持	35.850	5.796	0.187**	0.398**	1.000	
专业就业意愿	24.150	3.653	0.378**	0.609**	0.334**	1.000

注:** 表示 $P<0.01$。

2.3 新冠肺炎疫情认知与专业就业意愿的 Logistic 回归分析

将调查对象的专业就业意愿得分以中位数为截断值,分为高低两个组别作为因变量;将性别、年级、独生子女类别、学生干部情况、疫情防控志愿参与情况等因素作为控制变量,其中年级为多分类变量,对其进行哑变量转换;分别以新冠肺炎疫情认知 3 个维度、新冠肺炎疫情认知高低组为自变量纳入 Logistic 回归模型,进行回归分析。见表 2。

(C)1994-2022 China Academic Journal Electronic Publishing House. All rights reserved. http://www.cnki.net

表 2 2 151 例医学生新冠肺炎疫情认知与专业就业意愿的 Logistic 回归分析

自变量	Model 1					Model 2				
	β	P 值	OR	95%CI		β	P 值	OR	95%CI	
				下限	上限				下限	上限
个人风险认知	0.567	0.000	1.763	1.433	2.170					
社会风险认知	0.209	0.007	1.232	1.058	1.436					
疫情行为认知	0.809	<0.001	2.247	1.869	2.700					
疫情认知分组[a]						1.369	<0.001	3.931	3.239	4.771
性别[b]	-0.167	0.248	0.847	0.638	1.123	-0.134	0.344	0.874	0.662	1.155
年级[c]		<0.001					0.001			
大学二年级	0.084	0.649	1.088	0.757	1.565	0.020	0.914	1.020	0.710	1.466
大学三年级	-0.119	0.548	0.888	0.603	1.308	-0.184	0.351	0.832	0.565	1.225
大学四年级	-0.488	0.019	0.614	0.409	0.921	-0.531	0.011	0.588	0.392	0.883
独生子女类别[d]	0.149	0.178	1.160	0.935	1.441	0.146	0.184	1.157	0.933	1.436
是否为学生干部[e]	-0.115	0.280	0.891	0.723	1.099	-0.094	0.375	0.910	0.739	1.121
是否为疫情志愿者[f]	-0.128	0.248	0.880	0.709	1.093	-0.164	0.136	0.849	0.685	1.053
~2 对数似然值			2 542.873					2 593.912		
Cox & Snell R^2			0.108					0.086		
Nagelkerke R^2			0.148					0.119		

注:[a] 以疫情认知低分组为参照群体;[b] 以男性为参照群体;[c] 以大学一年级为参照群体;[d] 以独生子女为参照群体;[e] 以学生(社团)干部为参照群体;[f] 以参与新冠肺炎疫情防控志愿工作者为参照群体。

Model 1 为排除了控制变量影响后,新冠肺炎疫情认知 3 个维度对专业就业意愿的回归效应,结果显示,个体每增加一个单位的个人风险认知、社会风险认知、疫情行为认知,专业就业意愿就会分别提高 1.763 倍(95%CI=1.433~2.170)、1.232 倍(95%CI=1.058~1.436)、2.247 倍(95%CI=1.869~2.700)。Model 2 为新冠肺炎疫情认知高低分组与专业就业意愿的回归模型,结果显示,排除了控制变量影响后,新冠肺炎疫情高认知组的专业就业意愿是低认知组的 3.931 倍(95%CI=3.239~4.771)。Model 1、Model 2 结果均显示,在控制了社会人口学因素的情况下,医学生对新冠肺炎疫情的认知水平越高,其从事所学专业工作的就业意愿就越大,即新冠肺炎疫情认知对医学生专业就业意愿有正向预测作用。

2.4 社会支持与积极心理资本的链式中介效应检验

本研究在考察新冠肺炎疫情认知对专业就业意愿预测作用的基础上,结合与专业就业意愿有显著相关的社会支持与积极心理资本两个重要变量,构建结构方程模型路径图(图 1),分析新冠肺炎疫情认知通过社会支持与积极心理资本对于专业就业意愿的作用机制。所构建模型的拟合指数为:GFI=0.941,AGFI=0.917,NFI=0.943,RFI=0.929,IFI=0.948,TLI=0.935,CFI=0.948,RMSEA=0.067,模型拟合较好[5]。

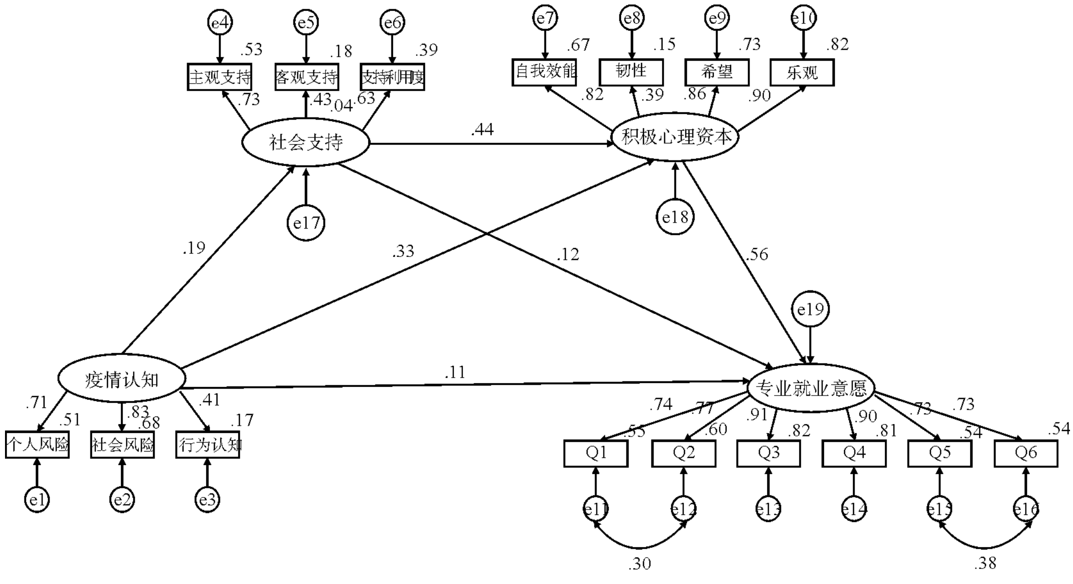

图 1 医学生新冠肺炎疫情认知的社会支持与积极心理资本的链式中介模型

采用偏差校正百分位 Bootstrap 法(重复抽样 5 000次)进行中介效应检验,结果见表 3。由表 3 可知,社会支持在新冠肺炎疫情认知与专业就业意愿间的中介效应 95%置信区间 Bias-corrected 检验结

(C)1994-2022 China Academic Journal Electronic Publishing House. All rights reserved. http://www.cnki.net

果为0.027～0.088,区间大于0,中介效应显著,效应值为0.052。心理资本在新冠肺炎疫情认知与专业就业意置信愿间的中介效应95%区间 Bias-corrected 检验结果为0.351～0.539,区间不包含0,中介效应显著,效应值为0.438。社会支持与心理资本在新冠肺炎疫情认知与专业就业意愿间的中介效应95%置信区间 Bias-corrected 检验结果为0.070～0.157,区间不包含0,中介效应显著,中介效应值为0.111。因此,社会支持与心理资本在新冠肺炎疫情认知与专业就业意愿间起多重链式中介作用,即新冠肺炎疫情认知即可以通过社会支持或心理资本间接影响医学生专业就业意愿,还可以通过社会支持进一步作用心理资本,间接影响医学生专业就业意愿。见表3。

表3 Bootstrap 中介效应检验

路径	效应值	标准误	Bias-corrected 95%*CI* 下限	上限	*P* 值
A	0.052	0.015	0.027	0.088	<0.001
B	0.438	0.048	0.351	0.539	<0.001
C	0.111	0.022	0.070	0.157	<0.001

注:路径A为疫情认知-社会支持-专业就业意愿;路径B为疫情认知-积极心理资本-专业就业意愿;路径C为疫情认知-社会支持-积极心理资本-专业就业意愿。

3 讨论

3.1 新冠肺炎疫情认知与医学生专业就业意愿的关系

本研究结果显示,新冠肺炎疫情认知可以正向预测医学生专业就业意愿,即随着对新冠肺炎疫情认知水平的提高,医学生对继续从事本专业的就业意愿越强,这与丁雪丽等[6]的研究结果相似。丁雪丽等[6]的研究显示,在新冠肺炎疫情下,83.7%的医学生职业选择的态度维持不变或者变得更加坚定。这表明医学生从事本专业的就业意愿并没有受到疫情期间产生的负性情绪的影响,或者说受到负性情绪的积极影响。一项SARS期间的研究显示,93.5%的医学生认为适度的焦虑与恐慌反而有助于其应对危险[7],李亚萍等[8]研究发现,面对疫情由焦虑、恐慌等负性情绪产生的积极影响远大于消极影响。医学生作为准医务人员,对各类突发公共卫生事件具有高度的敏感性,往往较同龄人承受更多的压力性事件,表现出更多的负性情绪,这些压力和情绪则会促使医学生增强对新冠肺炎的了解和认知,作为有深厚医学专业知识积淀的医学生在进一步认知到病毒的发病机制、治疗方案、感染风险、危险性以及预防措施等知识后,会增强其较其他专业学生的使命感与职业信心,使其觉得从事医疗工作是非常有意义的,进而会激发他们维护生命健康的职业觉悟。医学院校管理者应采取多样化手段加强对医学生新冠肺炎知识的培训,通过理论教学、现场培训与实践参与等方式,着重介绍与培训新冠肺炎的传染源、传播途径、易感人群、防护措施等知识以及有关新冠肺炎病毒的防控、治疗与研究的最新进展,帮助医学生更加深入地认识新冠肺炎,以缓解其面对新冠肺炎的紧张、畏惧等负面情绪,激发他们的职业使命感和坚定继续从事医学事业的信心。

3.2 社会支持的中介效应

本研究结果显示,社会支持在新冠肺炎疫情认知与专业就业意愿的关系间起中介作用,即表明新冠肺炎疫情认知在直接作用医学生就业意愿的同时还通过社会支持对专业就业意愿产生间接影响。相关研究显示,社会支持可以改变个体认知,促使个体产生积极情绪[9]。王轲等[10]的研究显示,新冠肺炎疫情期间较高的社会支持可以有效提升医学生的专业自豪感。根据社会支持理论,个体在高支持感下,压力所产生的负面效果会减小[11]。新冠肺炎疫情期间,广大医护人员抗疫的先进事迹受到社会的空前关注、赞赏与宣扬,人们对医务人员的支持力度也空前强烈,作为准医务人员的医学生也深受鼓舞,随着其感知的社会支持越高,其对新冠肺炎疫情的认知也表现的更为积极,其使命感、荣誉感与自豪感进一步激发,进而会削弱紧张、恐慌等压力所致的职业畏惧,医学生的专业认同感会得以恢复甚至提升,医学生继续从事本专业的就业意愿也随之增加。因此,医学院校管理者应注意关注医学生的心理健康状况,保障生活物质供应,在生活与学习上提供足够的支持,同时做好医务人员先进事迹宣传报道,争取更多的社会支持,从而帮助医学生保持良好的心理健康状态,进一步促进对医学事业的承诺与选择。

3.3 心理资本的中介效应

心理资本是个体贮藏于内心的一种积极向上的状态和能量[12],包含自我效能感、韧性、希望与乐观4个维度,具有能量补充与动机激发作用[13]。本次研究结果发现,心理资本同样在新冠肺炎疫情认知与专业就业意愿的关系间起中介作用。多项研究表明,大学生心理资本水平越高,其对所学专业的认同感越高,也更愿意从事与所学专业相关的职业[14-15]。疫情期间,随着医学生对疫情认知水平的提高,其自我效能感、希望等心理资本也随之增加,心理资本一方面通过"能量补充"促使个体倾向于积极归因,削弱由疫情产生的职业畏惧等负性情绪;同时,还通过"动机激发"促进医学生更加深刻全面地了解医学专业的价值和意义,激发专业认同度与使命感,对医学生继续从事所学专业的就业意愿起到积极作用。因此,医学院校管理者需注重提升医学生的心理资本,通过优化校园环境,营造良好氛围,改进教学手段,提供心理援助,开展实践活动等举措,培养医学生敢于应对挑战性任务的自我效能感,提高其应对突发事件冲击的能力,增强心

(C)1994-2022 China Academic Journal Electronic Publishing House. All rights reserved. http://www.cnki.net

理韧性,提升心理资本,促使医学生在逆境中更加坚定继续从医的意志。

3.4 社会支持、心理资本的链式中介效应

本研究结果显示,社会支持与心理资本在新冠肺炎疫情认知与专业就业意愿的关系间具有链式中介效应,即医学生的新冠疫情肺炎认知水平能够通过社会支持作用心理资本进而影响专业就业意愿。新冠肺炎疫情期间,随着医学生对于新冠肺炎疫情认知水平的提升,其也更加了解"内幕",更加渴望得到来自家庭、朋友、学校及社会的支持。根据社会人理论,人具有社会性,彼此之间不是相互孤立的,而是相互需要的,当个体感知到的支持与帮助越多,其内心的紧张与焦虑等负面情绪会受到削弱,会表现得愈加自信、从容、乐观与充满希望,对实现目标的动机愈加强烈[16]。也就是说,医学生在感知到高水平的社会支持时,其心理资本水平也就越高。而积极的心理能力能够促使大学生更加理性地进行职业规划与决策,使医学生愈加认可和肯定医学专业的价值[17],愈能激发其作为准医务人员救死扶伤的社会责任感,从而更加坚定信念,继续选择从事医学专业。因此,医学院校管理者需加强新冠肺炎相关知识的教育培训,采取各种措施加强对医学生的支持与引导,开展心理健康教育,开发积极心理资本,增强医学生的自我效能,激发医学生的职业使命感。

参考文献

[1] Lin C A, Lagoe C. Effects of news media and interpersonal interactions on H1N1 risk perception and vaccination intent[J]. Commun Res Rep, 2013, 30(2): 127-136.

[2] Ludolph R, Schulz P J, Chen L. Investigating the effects of mass media exposure on the uptake of preventive measures by Hong Kong residents during the 2015 MERS outbreak: the mediating role of interpersonal communication and the perception of concern[J]. J Health Commun, 2018, 23(1): 1-8.

[3] 张阔,张赛,董颖红. 积极心理资本:测量及其与心理健康的关系[J]. 心理与行为研究,2010,8(1):58-64.

[4] 肖水源.《社会支持评定量表》的理论基础与研究应用[J]. 临床精神医学杂志,1994,4(2):98-100.

[5] 温忠麟,侯杰泰. 马什赫伯特. 结构方程模型检验:拟合指数与卡方准则[J]. 心理学报,2004,36(2):186-194.

[6] 丁雪丽,刘思凡,荆雪,等. 医学生在新型冠状病毒肺炎疫情背景下职业选择的调查研究[J]. 中国高等医学教育,2020,35(6):22-23.

[7] 赵良玉,司晓芸. 重大突发公共卫生事件对医学研究生的心理影响及干预措施[J]. 医学教育研究与实践,2020(2):218-221.

[8] 李亚萍,朱建宏,王沐淇,等. 医学生新冠肺炎认知情况及疫情对医学生心理状态和职业认同感的影响[J]. 中国医学教育技术,2020,34(6):699-703;707.

[9] Zahodne L B, Sharifian N, Manly J J, et al. Life course biopsychosocial effects of retrospective childhood social support and later-life cognition[J]. Psychol Aging, 2019, 34(7): 867-883.

[10] 王轲,王琪,刘碧波,等. 新型冠状病毒肺炎疫情对某高校不同专业大学生心理的影响[J]. 西安交通大学学报(医学版),2021,42(1):146-151.

[11] Cohen S, Wills T A. Stress, social support, and the buffering hypothesis[J]. Psychol Bull, 1985, 98(2): 310-357.

[12] Luthans F, Youssef C, Avolio B. Psychological capital[M]. New York: Oxford University Press, 2007.

[13] Schaufeli W B, Salanova M, González-romá V, et al. The measurement of engagement and burnout: a two sample confirmatory factor analytic approach[J]. J Happin Stud, 2002, 3(1): 71-92.

[14] 陈俣睿. 大学生心理资本、专业认同与学业倦怠间的关系研究[D]. 长春:东北师范大学,2016.

[15] 周金莉. 高职护生心理资本与专业认同的相关性研究[J]. 护理管理杂志,2017,17(6):405-406;414.

[16] 周天梅,张权伟,王超颖. 社会支持对中学生抗挫折能力的影响:一个链式中介效应模型[J]. 西南师范大学学报(自然科学版),2020,45(6):88-96.

[17] 曹虹. 心理资本干预对高职护生专业承诺、职业决策自我效能的效果及作用机制研究[D]. 合肥:安徽医科大学,2018.

(收稿日期 2021-11-09)(本文编辑 杨婷婷)

(C)1994-2022 China Academic Journal Electronic Publishing House. All rights reserved. http://www.cnki.net
