# Supplementary material for: A study on the current status and influencing factors of psychological capital among medical undergraduates
Source: Front Psychol. 2026 Oct 30;17:1830730. doi: 10.3389/fpsyg.2026.1830730 (PMC13759799; doi:10.3389/fpsyg.2026.1830730)
Supplement: Supplementary file 1 [file Data_Sheet_1.ZIP › References PDF/Effects of Gratitude Intervention on Professionalism of Medical Students.pdf]

感恩干预对医学生医师职业精神的影响*

孙翠勇[①] 张瑞芹[②] 罗艳艳[②]

摘要:医学生职业精神教育是医学生教育的重要内容之一,以 119 名临床学生作为调查对象,分为对照组和试验组,通过感恩干预对医学生感恩、积极心理资本和职业精神的影响情况进行分析。采用六项目感恩问卷、职业精神量表、积极心理资本量表对干预效果进行评价,运用回归分析进行中介效应检验三者的关系。结果发现,感恩干预能显著提高医学生感恩、积极心理资本及职业精神水平,积极心理资本在感恩与职业精神的关系中起到部分中介的作用。

关键词:感恩干预, 积极心理资本, 医师职业精神, 医学生

中图分类号:R－05 **文献标识码:**A **文章编号:**1002－0772(2018)01－0049－03

DOI:10.12014/j.issn.1002－0772.2018.01a.13

Effects of Gratitude Intervention on Professionalism of Medical Students *SUN Cui-yong, ZHANG Rui-qin, LUO Yan-yan.*

School of Management, Xinxiang Medical University, Xinxiang 453003, China

Abstract: Professionalism education is very important for medical students. This study was conducted with 119 clinical students, who were divided into control group and experimental group, aiming to find out the effects of gratitude intervention on the gratitude, the positive psychological capital and the professionalism of medical students. The authors used via Gratitude Questionnaire-6, medical interns' professionalism evaluation form, and form of positive psychological capital to evaluate the effects of intervention. Moreover the regression analysis was used to investigate the relationship among gratitude, positive psychological capital and professionalism in medical students. The results the method of gratitude intervention could improve the level of gratitude, positive psychological capital and professionalism. Positive psychological capital played a partial mediating effect in the relationship between gratitude and professionalism.

Key Words: gratitude intervention, positive psychological capital, medical professionalism, medical student

医师职业精神是医学生所具备的一种能力和素养,医学生对学校职业精神培育方式的认可也会带动其自身对职业精神的认可与学习,对于提高医学生的职业道德大有裨益[1]。感恩与心理资本是积极心理学领域中重要的心理健康指标,如主观幸福感、自我概念、职业素养、专业承诺等,研究证实,积极的心理健康水平越高,医生的职业精神素养越高[2]。本文利用感恩引导反思干预方法,提高医学生感恩、积极心理资本和职业精神水平,并进一步验证三者之间的关系,为提高医学生职业精神提供干预措施,为改善医患关系提供新的途径。

1 对象与方法

1.1 研究对象

通过抽样法抽取新乡医学院 2013 级本科临床医学专业学生 120 名,按照随机的方法分为对照组和试验组,剔除对照组一份无效问卷,实收问卷分别为 59 份和 60 份。

1.2 研究方法

1.2.1 试验组感恩引导反思法

在 2013 级本科医学生第七学期临床见习时进行感恩干预,每周见习 3 天,上课 2 天。指导学生书写反思日记,共 10 周,每天见习结束或参加活动后需要发表自己的感悟与体会,并且按照 1 分～7 分递增的顺序,记录感恩的分数。每周抽查感恩日记 1 次,起到监督作用。集中进行感恩讲座和放映感恩视频各 4 周。

1.2.2 对照组干预方法

对照组进行常规的见习讲座、临床教学、专业思想教育活动等。

1.3 研究工具

1.3.1 六项目感恩问卷

该问卷由 McCullough 等编制、杨芳[3]汉化,共有 6 个条目,被试者需要根据最近的感受进行评价。评价方式为 1 分～7 分,从"完全不同意"到"完全同意",其中条目 3 和条目 6 为反向计分题,得分越高,表示个体的感恩水平越高。该量表的内部一致性为 0.651。

1.3.2 职业精神量表

由中南大学美国中华医学基金会资助研究项目组提供的"医学生职业精神评价调查表"[4],共 29 个条目,

* 基金项目:2017 年河南省医学教育项目(Wjlx2017075);2015 年河南省教育厅重点项目(15A320062);2017 年新乡医学院高峰高原项目;2016 年新乡医学院精神神经医学学科群支持计划

①新乡医学院管理学院 河南新乡 453003
②新乡医学院护理学院 河南新乡 453003
作者简介:孙翠勇(1979－),男,硕士,讲师,研究方向:教育管理。
通讯作者:张瑞芹(1981－),女,硕士,讲师,研究方向:积极心理学。E-mail:zrq19221@163.com

分为 7 个维度，评价方式为 5 分～0 分，评价结果以"优－良－中－差－很差－不清楚"为等级划分。该问卷总体 Cronbach's α 系数为 0.89。

1.3.3 积极心理资本量表

该量表由张阔等[5]编写，共有 26 个条目，分为 4 个维度。采用 Likert 7 级评分法，由"完全不同意"到"完全同意"以 1 分～7 分评价，总分值为 26 分～182 分，总分值越高，表示受试者心理资本积累情况越好。该问卷总体 Cronbach's α 系数为 0.881，其中各维度 Cronbach's α 系数为 0.713～0.841。

1.3.4 试测方法

试验组与对照组均进行了两个时间点的问卷测试。第一次测试时间是干预前一天(T1)，作为基线水平。第二次测试时间是干预结束后第一天(T2)，作为后测水平。

1.4 统计分析

采用 SPSS 22.0 对数据进行描述性统计分析、χ^2 检验，两组均数比较用独立样本 t 检验，用回归分析进行中介效应检验。

2 结果

2.1 对照组和试验组一般资料数据

对照组和试验组一般数据如下：年龄均值为(20.88±0.85)岁和(20.55±1.16)岁(t=1.803，P=0.074)，籍贯(城市为 26 人和 17 人，农村为 33 人和 43 人)(χ^2=2.936，P=0.087)，性别(女为 40 人和 36 人，男为 19 人和 24 人)(χ^2=0.906，P=0.447)，独生子女(是为 22 人和 26 人，否为 37 人和 34 人)(χ^2=0.310，P=0.711)。一般资料比较结果显示，无统计学差异(P>0.05)，具有可比性。

2.2 两组干预前后进行独立样本 t 检验的分数比较

干预前，感恩、职业精神、积极心理资本的总分均值差异不显著(P>0.05)；干预后，试验组感恩、职业精神、积极心理资本的总分均值显著高于对照组(P<0.05)，见表 1。试验组除了感恩的总分均值与干预前无差异(P>0.05)之外，职业精神和积极心理资本的总分均值均高于干预前(P<0.05)，对照组感恩、职业精神、积极心理资本的总分均值与干预前无差异(P>0.05)。

表 1 对照组与试验组感恩、职业精神、积极心理资本比较(分，$\bar{x}\pm s$)

项目	时间	对照组(n=59)	试验组(n=60)	t 值	P 值
感恩总分	基　线(T1)	38.27±2.504	38.93±4.325	－1.020	0.310
	干预后(T2)	34.63±4.303	38.92±4.589	－5.258	0.000
利他主义	基　线(T1)	15.64±2.362	14.85±2.306	1.856	0.066
	干预后(T2)	15.80±2.124	17.63±1.089	－5.952	0.000
荣誉感与正直	基　线(T1)	28.85±3.487	28.73±4.836	0.148	0.883
	干预后(T2)	29.14±3.219	30.88±2.681	－3.220	0.002
关怀人爱之心和善于交流	基　线(T1)	8.51±1.318	8.52±1.535	－0.031	0.975
	干预后(T2)	8.53±1.180	8.93±1.039	－2.002	0.048
尊重他人	基　线(T1)	17.81±2.452	17.83±2.644	－0.043	0.966
	干预后(T2)	17.47±2.087	18.37±1.766	－2.519	0.013
尽职尽责	基　线(T1)	20.02±3.665	19.70±4.224	0.437	0.663
	干预后(T2)	20.08±3.245	21.30±2.689	－2.226	0.028
追求卓越和精益求精	基　线(T1)	16.93±2.791	17.02±2.837	－0.163	0.871
	干预后(T2)	16.97±2.512	18.12±1.595	－2.988	0.003
领导能力	基　线(T1)	12.10±2.218	12.47±2.127	－0.916	0.361
	干预后(T2)	12.15±1.946	13.42±1.369	－4.104	0.000
乐观	基　线(T1)	30.12±5.113	29.28±5.530	0.855	0.394
	干预后(T2)	29.95±5.036	39.42±1.418	－14.010	0.000
希望	基　线(T1)	29.54±3.816	29.77±3.881	－0.318	0.751
	干预后(T2)	30.19±5.280	37.98±2.311	－10.465	0.000
韧性	基　线(T1)	36.66±3.767	36.63±3.742	0.04	0.968
	干预后(T2)	37.44±3.420	42.35±2.845	－8.518	0.000
自我效能	基　线(T1)	37.20±3.658	37.32±3.671	－0.168	0.867
	干预后(T2)	37.86±3.240	43.10±2.741	－9.522	0.000

2.3 感恩干预后试验组相关性分析

感恩与职业精神呈正相关(r=0.420，P<0.01)，感恩水平越高，其职业精神素养就越高；感恩与积极心理资本呈正相关(r=0.514，P<0.01)，感恩水平越高，其积极心理资本水平就越高；积极心理资本与职业精神呈正相关(r=0.729，P<0.01)，积极心理资本

水平越高，职业精神素养就越高。见表 2。

表 2　感恩、职业精神、积极心理资本相关性分析(r)

项目	感恩总分	职业精神总分	积极心理资本总分
感恩总分	1.000	0.420*	0.514*
职业精神总分		1.000	0.729*
积极心理资本总分			1.000

注：* 表示 $P<0.01$

2.4　干预后感恩、积极心理资本与职业精神的关系

通过回归分析考查干预后积极心理资本在职业精神和反思感恩之间是否存在中介作用。首先，以职业精神为因变量、以感恩为自变量进行回归分析，y=0.982x，$P<0.01$，可以进行下一步；其次，以积极心理资本为因变量、以感恩为自变量的回归分析，m=0.994x，$P<0.001$，可以进行下一步；再次，以感恩、积极心理资本为自变量，以职业精神为因变量进行回归分析，y=0.431x+0.565m，$P<0.01$，β均有统计学意义。最后，判断积极心理资本对感恩影响职业精神是完全中介效应还是部分中介效应，计算得出中介效应量为 0.994×0.565=0.561，中介效应占总效应的比例为0.994×(0.565/0.982)×100%=57.19%，经计算发现中介效应解释了因变量的方差变异为 57.19%，具有部分中介效应。见表 3。

表 3　积极心理资本对感恩和职业精神的中介效应的检验

结果变量	因变量	自变量	R^2_{adj}	β	t 值	P 值
第一步	职业精神	感恩	0.988	0.982	71.238	0.000
第二步	积极心理资本	感恩	0.985	0.994	36.842	0.000
第三步	职业精神	感恩	0.990	0.431	2.400	0.000
		积极心理资本		0.565	3.148	0.000

3　讨论

3.1　感恩干预提高了医学生的积极心理资本和职业精神

目前提高医学生职业精神的教学方法中，引导反思法是常用的一种[6]。本研究的感恩反思干预方法是借鉴这种教学方法，加以修改，与医学生专业密切相关。通过记录感恩反思日记，试验组的感恩水平高于没有接受感恩干预的对照组($P<0.01$)，这表明通过记录临床见习中的一些感动的事迹或某些细小却能体现温情的举动，真实感受到了临床教师和患者对自己的所做工作的肯定后，加深了对医学服务和医师职业精神的理解，增强了他们对专业的认同感和满意度，对医生这个职业更是充满了信心和坚定的信念，表现出了积极向上的学习和工作热情，在见习中医技水平有了一定的提高。通过感恩讲座和感恩活动的开展，医学生的积极心理资本有了明显的提升($P<0.001$)，与 Larson 和 Luthans 研究员工的心理资本对其工作满意度有重要的影响结论一致[7]。感恩建构起了重要的积极心理资本个体资源，增强了个体的心理韧性及适应性。感恩提升职业精神表现在医学生对职业的认可和工作积极性上，同时增强了医学生回报社会、服务社会的积极性与自愿性，从而增强了其对社会的回报程度。

3.2　积极心理资本对感恩和职业精神的中介效应分析

本研究发现，尽管感恩对职业精神具有直接影响，但中介检验结果表明，积极心理资本在感恩与职业精神的关系中起到部分中介的作用。说明感恩对职业精神具有直接和间接作用，部分作用是通过积极心理资本的中介作用间接进行的，中介作用占感恩对职业精神总效应的 56.5%。这说明感恩水平高的医学生一方面直接提高了职业精神，另一方面又体现了积极心理资本水平高的医学生更懂得感恩，具有更高的医师职业精神。

本研究通过与临床实习教学紧密结合的感恩干预措施，引导医学生记录感恩反思日记，提高了其积极心理资本和医师职业精神，也验证了积极心理资本在感恩与医师职业精神之间的中介效应，为进一步增强医学生的医师职业精神提供了参考依据。

参 考 文 献

[1]　钟凤宏. 医学生医师职业精神认知现状调查研究：以大连医科大学的调查为例[J]. 医学与哲学，2017，38(1A)：75－77.
[2]　ZHOU Ping，SHI Jianwei，ZHANG Yu. Relationships between physicians' psychological contract and job satisfaction，intention to leave medical practice，and professionalism：A cross-sectional survey in China[J]. Lancet，2016，388(1)：12－18.
[3]　杨　芳. 冠脉支架植入患者健康促进的感恩模型研究[D]. 上海：第二军医大学，2013.
[4]　梁　进，刘瑞梓，鲁英青，等. 复旦大学上海医学院医学生职业素养现状调查研究[J]. 中华医学教育杂志，2015，35(2)：174－177.
[5]　张　阔，张　赛，董颖红. 积极心理资本：测量及其与心理健康的关系[J]. 心理与行为研究，2010，8(1)：58－64.
[6]　韦旭楠，王子薇，刘露萍，等. 国内外医学生职业精神教学方法研究进展[J]. 医学与哲学，2016，37(6A)：41－43.
[7]　邓晨曦. 感恩对高职学生心理健康及职业素质的影响[J]. 辽宁高职学报，2014，16(5)：97－99.

收稿日期：2017－03－24
修回日期：2017－11－22　　　　**(责任编辑：邹明明)**
