# Supplementary material for: A study on the current status and influencing factors of psychological capital among medical undergraduates
Source: Front Psychol. 2026 Oct 30;17:1830730. doi: 10.3389/fpsyg.2026.1830730 (PMC13759799; doi:10.3389/fpsyg.2026.1830730)
Supplement: Supplementary file 1 [file Data_Sheet_1.ZIP › References PDF/Lou, R. G. and Q. Q. Zhao (2002). Exploration and Practice of Career Planning Guidance for College Students.pdf]

DOI:10.16298/j.cnki.1004-3667.2002.06.040

大学生职业生涯规划指导的探索与实践

楼仁功 赵启泉

随着高等教育管理体制的改革和毕业生就业方式的改变,开展大学生职业生涯规划指导,帮助大学生增强主体意识,开拓美好前程,是新形势下加强高校学生思想政治教育,做好毕业生就业指导工作的一种行之有效的方式和方法。

一、大学生职业生涯规划指导

1.大学生职业生涯规划指导提出的背景

第一,随着计划经济体制向社会主义市场经济体制的转轨,“国家统包统分”的毕业分配制度已被“在国家有关毕业生就业的方针和政策指导下,通过‘供需见面’、‘双向选择’,在一定范围内落实就业单位”的毕业就业制度所取代。大学毕业生择业的自主权越来越大,但由于大学生的职业观和高校的就业指导未能与时俱进,许多大学生的职业意识竞争意识薄弱。因此,迫切需要改进毕业就业指导方式,运用科学的方法,指导大学生进行职业生涯规划。

第二,随着高等教育改革的不断深入,学分制的逐步到位,学生选择专业、课程的自主权加大,以及知识量的快速增长,迫切需要在大学生中开展职业生涯规划指导,目的是使大学生明确今后的职业发展方向,确立大学期间的学习目标,学会有目的地构建知识、能力、素质结构,为今后的职业生涯打下坚实的基础。

第三,由于知识经济的来临,导致产业结构调整、技术更新加快,企业的竞争空前激烈,企业的兼并、裁员、破产加剧,致使一些职业岗位减少甚至消亡;同时新的职业不断的产生,新的岗位不断增加,以致过去学一种技术,用一辈子的情况大大减少。在这种就业新形势下,容易导致一些大学生迷失学习方向,失去学习动力。因此,大学生迫切需要得到相应的职业生涯的指导,明确自己今后的职业生涯目标和发展方向。

2.大学生职业生涯规划指导的特殊性

第一,由于大学生对社会、对职业的了解较少,择业观尚未定型,因此,大学生职业生涯规划中的不确定因素较多,要制定长期的具体的规划有较大的难度;另一方面,大学生虽然有一定的专业,但在大学期间可以选修其他一些课程,因此,职业的可塑性还很大;大学生职业生涯规划的目的不是现在就业,而是为今后整个职业生涯发展打基础,因此更注重素质的提高,而不仅是具体技术的培训。

第二,目前,我国对大学生职业生涯规划指导的研究较少,基本上没有专业人员,学校思政人员也缺少这方面的知识和专业培训;我国的国情及办学体制与国外有较大的差别,在高考志愿的选择上,父母、老师的指导、影响较大,常常先选学校、再选专业,许多考生也并不了解自己选择的专业和今后的职业方向的关系,上大学后,仍有相当一部分大学生没有明确的职业定位;我国对大学毕业生的教育更注重社会的需要,而国外大学的职业生涯指导则注重对大学生个性的测评,注重职业意识、职业技能的培养。因此,对大学生开展职业生涯规划指导,思政人员首先自己需要学习职业生涯规划的知识,提高职业生涯规划指导的能力;其次,要引导大学生提高主体性意识、自主选择职业的意识,学会如何正确规划自己今后的职业生涯。

3.开展大学生职业生涯规划指导的原则

第一,以学生为主体的原则。开展大学生职业生涯规划指导,必须坚持以学生为主体,引导大学生根据自身的素质特征,结合个人的价值取向和兴趣爱好,并考虑人才市场的现状和发展趋势,设计职业发展目标及其实施计划。

第二,以社会为导向原则。社会的需求在不断发展变化,因此在指导大学生设计职业生涯规划时,要教育、引导大学生注意职业需求变化对职业生涯的影响,规划要有弹性,要密切关注职业的发展变化,学会收集、分析社会需求信息,并根据社会的变化调整职业生涯规划。

第三,指导的个性化原则。每个人的职业生涯目标、规划应因人而异。因此,职业生涯规划的指导要注意充分发挥大学生个性特长,指导大学生按照“择己所爱,择己所长,择世所需,择己所利”的原则,进行正确的职业“定向”、“定点”、“定位”。

第四,指导的系统性原则。大学生职业生涯规划与毕业生或在职人员比较,更注重其素质的积累、提高,注重对职业需求发展变化的适应性。因此,大学生职业生涯规划的指导,不仅仅是组织一次活动,而是贯穿整个大学学习生活。

第五,指导的主动性原则。由于大学生自主意识的增强,主动接受帮助指导的意识淡化,因此,在开展大学生职业生涯规划活动中,要主动加强与学生的正式或非正式的沟通。

(C)1994-2022 China Academic Journal Electronic Publishing House. All rights reserved. http://www.cnki.net

二、大学生职业生涯规划活动的实施

1.大学生职业生涯规划的步骤、内容

第一,认识自我。从兴趣、特长、性格、价值观、品德、能力等方面深入认识自我。通过优势分析——曾经做什么,学会了什么,最成功的是什么;弱势分析——性格弱点,教训或经历中所欠缺的,等等,全面地认识自己的优势与不足。

第二,了解社会。通过与父母、亲戚、朋友、老师、同学的沟通,了解他们对自己的看法与期望,了解社会就业形势和社会对人才的素质要求,使大学生认识社会环境对成才和职业生涯发展的影响,增强自己的社会责任感。

第三,确立目标。在认识自我、了解社会的基础上,引导大学生从自身实际和社会的需要出发,确立职业发展的方向,明确达到职业目标需要具备的素质和实现目标的优势与困难。

第四,制定策略。引导大学生通过制定大学学习生活的总体目标和阶段性目标、步骤、措施与标志,增强学习自觉性和积极性。制定大学毕业后总体的目标、步骤、措施、标志,使长远目标与大学学习有机衔接,增强学习的目的性。

第五,生涯评估。引导大学生结合大学生综合素质测评和父母、同学、朋友、教师的评价对自己职业生涯规划进行分析、评估、修正。

2.大学生职业生涯规划指导的方式、方法

第一,根据不同学习阶段的特点,有针对性地开展职业生涯规划指导。大学生在学习的不同阶段,其学习的重点不同,心理特征有别,关注的重点有异,考虑问题的方式也不一样。因此,针对大学生不同学习阶段的特点,有针对性地开展职业生涯规划指导,才能取得较好的效果。

第二,指导大学生根据自身实际需要,自主地、有选择地接受指导。不同的学生,个性不同,职业生涯的道路也多种多样。因此,大学生职业生涯规划的指导,必须设计多种成才途径和提高素质的指导模块,让学生根据自身实际需要,自主地、有选择性地接受指导。

第三,建立职业生涯规划指导网站,开展网上职业生涯规划指导。网络内容丰富、交流平等,是满足学生个性化、多样化地接受指导;实现社会与学生、教师与学生、学生与学生平等交流的有效途径。因此,建立大学生职业生涯规划指导网站,可以引导学生根据自身职业生涯规划的需要,自主地选择、利用丰富的网络资源。

第四,加强思政人员的培训,提高职业生涯规划指导的能力。高校思政人员要主动学习研究大学生成才规律,学会指导大学生根据自己个性、爱好和社会需要,确立职业生涯目标和实施规划。高校要创造条件,加强思政人员的职业生涯规划指导的培训,提高思政人员职业生涯规划指导的能力。

第五,引导教师与社会的参与,提高职业生涯规划指导的效益。职业生涯规划的指导是一个系统工程。大学生择业观的形成,职业技能、素质的提高,决不仅仅是思政人员所能完成的,需要社会各界的广泛参与。因此,增强广大业务教师对大学生职业生涯规划的指导意识,加快推进导师制,并通过社会实践加强学生与社会的沟通,对提高大学生职业生涯规划的指导的效益有着一定的积极作用。

三、大学生职业生涯规划指导的成效

第一,通过开展大学生职业生涯规划指导,增强了大学生主体意识。由于受应试教育、工具教育的影响,有相当一部分大学生主体意识欠缺。学习是为了父母、老师,却唯独没有考虑是自己今后职业生涯发展的需要。开展大学生职业生涯规划指导后,大学生全面地认识自己的个性、长处与不足,认识社会对人才的需求,从而制定了自己的职业生涯目标,并自觉地将目标规划转化为行动,增强了学习的目的性,主体性意识明显增强。

第二,通过开展大学生职业生涯规划指导,帮助大学生开拓美好前程。虽然每个学生都有成才的愿望,但由于原来缺乏职业生涯规划教育,有的有目标不知从何做起,有的目标还不明确甚至还没有。职业生涯指导则促进了大学生树立正确的职业生涯观,学会了根据自身的素质特征,结合个人的价值取向和兴趣爱好,并考虑人才市场的现状和发展趋势,来确立自己职业生涯目标,设计符合个人实际的合理的职业发展规划。

第三,开展大学生职业生涯规划指导是新形势下高校学生思政工作的一种行之有效的新形式、新方法。随着社会主义市场经济的不断发展和现代化事业的不断推进,社会经济成分、组织型式、就业方式、利益关系和分配方式的多样化,使大学生思想的独立性、选择性、多变性、差异性明显增强。重社会、集体,忽视个体教育的传统思想政治工作面临重重困难。围绕高素质创新人才培养,开展大学生职业生涯规划指导,不仅使高校学生思政工作更贴近学生的需要,而且使国家、社会利益与学生个体的利益有效地统一起来,是提高学生思政工作针对性、有效性、系统性和规范性的有效途径之一。

(作者工作单位:浙江大学环境与资源学院,浙江杭州 310029)

(C)1994-2022 China Academic Journal Electronic Publishing House. All rights reserved. http://www.cnki.net
