# Supplementary material for: A study on the current status and influencing factors of psychological capital among medical undergraduates
Source: Front Psychol. 2026 Oct 30;17:1830730. doi: 10.3389/fpsyg.2026.1830730 (PMC13759799; doi:10.3389/fpsyg.2026.1830730)
Supplement: Supplementary file 1 [file Data_Sheet_1.ZIP › References PDF/Positive Psychological Capital Measurement and Relationship with Mental Health.pdf]

心理与行为研究 2010，8(1)：58~64
Studies of Psychology and Behavior

积极心理资本：测量及其与心理健康的关系*

张 阔[1] 张 赛[2] 董颖红[1]
（1 南开大学社会心理学系，天津 300071）（2 武警医学院附属医院，天津 300162）

摘 要 在文献分析和参考国内外相关测量工具的基础上，编制了包含自我效能、韧性、乐观、希望等 4 个因子的积极心理资本问卷（*PPQ*）。信度和效度验证的结果显示，该问卷具有良好的结构效度和内部一致性信度；验证性因子分析的结果也支持了心理资本这一高阶因子的合理性。研究还发现，积极心理资本及其子成分与自尊、内控性、情感平衡以及焦虑、抑郁、偏执、人际敏感等心理健康指标有显著的相关，回归分析的结果也显示心理资本对心理健康有积极的促进作用。

关键词 积极心理学，心理资本，测量，心理健康。
分类号 B849

1 引言

在 20 世纪末，以时任 APA 主席的 Seligman 为首的一批心理学家发起了积极心理学（*positive psychology*）运动[1]。积极心理学强调用科学的方法研究人的积极情感、优秀品质和积极心理状态，以促进个体认识和发掘自身发展潜力，提升个人幸福感，增强团队和组织的效能[2,3]。近年来，积极心理学已经在人格、健康、临床、咨询和治疗等心理学分支领域产生了较大影响[4,5]。受积极心理学运动的影响和启发，以美国著名管理学家 Luthans 为首的一批组织行为学者，将积极心理学的思想引入到组织行为学领域，倡导积极组织行为（*positive organizational behavior*）的研究，希望通过测量和开发积极导向的心理特性和能力，来获取持续的人力资源竞争优势[6,7]。为了和人本主义心理学、人类潜能运动（如 *NLP*）等理论相区别，Luthans 等为积极组织行为的研究对象设定了四条标准：①以科学的理论和研究为基础；②可以准确测量；③类状态（*state-like*），会发展变化，因而是可开发的；④积极导向，即对绩效和满意度有正面影响[7,8]。

2004 年，Luthans 和 Youssef 等人在积极心理学和积极组织行为的框架下，提出了积极心理资本（*positive psychological capita1*）的概念，简称心理资本（*PsyCap*）[9,10]。Luthans 等将心理资本定义为，“个体在成长和发展过程中表现出来的一种积极心理状态”，它包括了四个核心成分：自我效能（*self-efficacy*）、乐观（*optimism*）、韧性（*resiliency*）和希望（*hope*）[8]。其中，自我效能是指个体有胜任任务的自信，能面对挑战并力争成功；乐观是指个体具有积极的归因方式，并对现在和未来持积极态度；韧性是指个体能从逆境、挫折和失败中快速恢复过来，甚至积极转变和成长；希望则是通过各种途径努力实现预定目标的积极动机状态。尽管除此以外，还有一些心理品质也具有积极的倾向，如幸福感、幽默等，但 Luthans 等认为自我效能、乐观、韧性和希望这四种心理结构在心理学领域都有很好的研究基础和有效的测量工具，因而最符合积极组织行为和积极心理资本的标准。这四种结构不仅每一种都具有概念上的独立性和有实证基础的区分效度，还会相互促进，以协同方式发挥作用，从而形成心理资本这一更高层次的构念[8]。此外，为了强调心理资本各成分的可变性和可开发性，Luthans 认为，心理资本的 4 个子成分（自我效能、乐观、韧性和希望）都处于特质——状态的连续体上偏向状态的一端，即所谓的“类状态”[8,11]。

心理资本的概念一经提出，便在心理学和管理学领域受到重视，相关的理论和应用研究也不断涌

收稿日期：2009-3-27
* 本研究得到天津市科技计划 09ZCZDSF04600 项目和天津市社科规划 TJJX062014 项目的资助。
作者简介：张 阔，男，南开大学社会心理学系副教授，博士。Email: zhkuo@126.com。

(C)1994-2022 China Academic Journal Electronic Publishing House. All rights reserved. http://www.cnki.net

现。例如，Luthans 和 Avolio 等人的研究发现，员工的心理资本对其工作绩效有促进作用 [12]；Avey，Patera和 West 研究证明，心理资本及希望、乐观、韧性和自我效能都与员工的旷工呈负相关 [13]；Jensen 和 Luthans 的研究发现，企业家的心理资本与其领导力呈正相关，对下属的心理资本也有积极影响 [14]；Larson 和 Luthans 的研究发现，员工的心理资本对他们的工作态度有积极影响 [15]。

由于在心理学领域中，对心理资本的 4 个子成分的研究与测量早已存在，因此国内外学者在心理资本的相关研究中，大多借鉴已有的测量工具，即将测量四种心理结构的量表组合起来，用于测量心理资本 [12,13]。其中最常用的是 Parker 开发的自我效能量表，Scheier 和 Carver 开发的乐观量表，Wagnild 和 Young 开发的乐观量表，以及 Snyder 开发的状态希望和特质希望量表。但这样的组合测量缺乏充分的结构效度证据，而且项目也较多，不利于实践应用。后来，Luthans 等人以上述量表为基础，开发了具有 24 个项目的心理资本问卷（*PCQ*），其中译本也已由国内学者李超平完成，但 PCQ 主要适用于工作人员和管理者，其适用对象的范围相对有限，且目前尚缺乏充分的效度证据 [11,16]。此外，Luthans 等在理论建构的过程中，为突出心理资本可开发的特点，使得 PCQ 倾向于对心理状态的测量，这也导致了 PCQ 的重测信度不够理想 [11]。

为更好地解决上述问题，本研究拟开发更具一般性、适用范围更广的积极心理资本问卷（*PPQ*），并探索心理资本高阶因子结构的合理性。此外，以往对心理资本的研究多采用组织行为学的视角，考察其对工作绩效和周边绩效的影响，本研究拟采用心理健康水平为效标，来验证问卷的实证效度，同时也考察心理资本与心理健康水平的关系。

2 研究方法

2.1 初始问卷的形成

自心理资本概念提出以来，很多学者都对其内容进行了探讨。例如，Goldsmith 等认为，心理资本的核心是自尊；Page 和 Bonohue 认为，心理资本应该包括希望、乐观、自我效能、韧性和诚信等 5 个成分；Larsons 等提出，心理资本应该包括自我效能、乐观和韧性；Jensen 和 Luthans 曾使用包含了希望、乐观和韧性测量的心理资本问卷，国内学者仲理峰的研究中也采用了类似的测量 [17,18]。从已有研究来看，心理资本的 4 因子结构被更多的学者所接受，因此本研究中也沿用了 4 因子的问卷结构。

问卷初始项目的搜集和编写工作主要通过两种途径进行。通过文献回顾，检索国内外相关文献，收集与心理资本的 4 个因子相关的测量问卷，对其项目进行分析和整理。此外，根据心理资本的理论建构和自我效能、乐观、韧性和希望的概念，由研究小组自拟了一部分项目。将这两部分项目组合起来，形成项目池。再请 3 名南开大学社会心理系的研究生，独立地评定每个项目测量的有效性。最后，研究小组经过讨论，从测量每个因子的项目库中抽取 10 个有效性评定较高的项目，形成了包含 40 个项目的初始问卷，各项目均采用 7 点评定的形式。

2.2 样本和施测程序

初测样本：145 名大学生志愿完成了初始问卷的测试，获得有效问卷 137 份（其中男生 36 份，女生 101 份）。根据初测结果进行项目分析，删去区分度小于 0.45 及因子负荷不符合单维性要求的项目，调整了部分项目的表述，另外从原项目库中补充若干项目，组成再测问卷。

测试样本 2：193 名大学生志愿完成了再测问卷的测试，获得有效问卷 181 份（其中男生 32 份，女生 149 份）。根据再测结果进行项目分析和因子分析，删去区分度小于 0.5 及因子负荷不符合单维性要求的项目，形成了包含 4 个因子 26 个项目的正式问卷。

测试样本 3：223 名大学生志愿完成了使用正式问卷进行的重测，获得有效问卷 210 份（其中男生 108 份，女生 102 份）。根据测试结果进行验证性因子分析，检验 4 因子模型及其高阶因子模型的合理性。

采用 Spss13.0 和 Lisrel8.5 软件进行数据分析。

2.3 效标测量

样本 3 的 223 名大学生在测试正式的心理资本问卷的同时，还参加了效标问卷的测试。效标问卷包括：自尊量表（*SES*）、核心自我评价量表（*CSES*）、情感量表（*AS:PNA*）、症状自评量表（*SCL-90*）。其中 CSES 选取了一般效能和控制点两个分量表；SCL-90 选取了焦虑、抑郁、偏执、人际敏感等 4 个分量表。

3 结果分析

3.1 基于样本 2 的探索性因子分析

(C)1994-2022 China Academic Journal Electronic Publishing House. All rights reserved. http://www.cnki.net

正式的积极心理资本问卷（*PPQ*）由 26 个项目组成，其中自我效能和韧性因子各包括了 7 个项目，乐观和希望因子各包括了 6 个项目。对正式问卷的测试数据进行 Bartlett 球形检验，结果 χ^2=2384.953，p<0.000；KMO=0.868，显示数据适合做因子分析。采用主成分分析法进行因子分析，通过观察碎石图，发现第一个因子方差贡献率为 30.3%，而其后的各因子方差贡献率均在 10%以下且变化平缓，绝大部分项目在第一因子上的负荷也在 0.4 以上，显示单因子模型的解释有一定的合理性。但单因子模型方差解释率较低，且不利于更深入分析，故再采用 Promax 斜交旋转法，得到 4 因子模型，各因子特征值均大于 1，累积方差贡献率为 54%，比单因子模型有较大提高。各项目的因子负荷见表 1。

表 1 积极心理资本问卷各因子所属项目的因子负荷

因子 1	负荷	因子 2	负荷	因子 3	负荷	因子 4	负荷
T1	0.87	T8	0.82	T15	0.84	T21	0.72
T2	0.82	T9	0.82	T16	0.82	T22	0.72
T3	0.67	T10	0.69	T17	0.80	T23	0.66
T4	0.65	T11	0.67	T18	0.66	T24	0.57
T5	0.64	T12	0.64	T19	0.60	T25	0.45
T6	0.61	T13	0.59	T20	0.50	T26	0.44
T7	0.48	T14	0.51				

其中，第 1 因子（自我效能）的方差贡献率约为 17%，包括了“我的见解和能力超过一般人”，“我乐于承担困难和有挑战性的工作”等 7 个项目，项目共同度的均值为 0.54。第 2 因子（韧性）的方差贡献率约为 14%，包括“遇到挫折时，我能很快地恢复过来”，“糟糕的经历会让我郁闷很久”等 7 个项目，项目共同度的均值为 0.53。第 3 因子（希望）的方差贡献率约为 13%，包括了“我充满信心地追求自己的目标”，“我积极地学习和工作，以实现自己的理想”等 6 个项目，项目共同度的均值为 0.55。第 4 因子（乐观）的方差贡献率约为 10%，包括了“我总是看到事物好的一面”，“我觉得生活是美好的”等 6 个项目，项目共同度的均值为 0.52。

以项目分数和子问卷总分的相关为指标，计算各项目的区分度，结果见表 2。

表 2 项目的区分度

项目	区分度	项目	区分度	项目	区分度	项目	区分度
T1	0.74	T8	0.78	T15	0.80	T21	0.64
T2	0.70	T9	0.76	T16	0.77	T22	0.68
T3	0.80	T10	0.76	T17	0.64	T23	0.61
T4	0.74	T11	0.66	T18	0.73	T24	0.72
T5	0.69	T12	0.69	T19	0.74	T25	0.74
T6	0.71	T13	0.71	T20	0.61	T26	0.71
T7	0.65	T14	0.65				

从表 2 中可见，积极心理资本问卷（*PPQ*）26 个项目的区分度介于 0.61~0.80 之间，均比较理想。

3.2 基于样本 3 的验证性因子分析

为验证问卷的结构效度，基于样本 3 的数据进行验证型因子分析。共提出了 5 种理论模型。其中，模型 1 为标准的 4 因子模型；模型 2 为高阶因子模型，即在模型 1 的基础上，假定存在高阶的心理资本因子；模型 3 为纯粹的单因子模型，用于检验将所有项目归属于一个心理资本因子的合理性。此外，由于心理资本 4 个子成分间存在着较密切的关系，例如，乐观的个体通常具有更高的韧性，而高自我效能的个体通常更加乐观，反之亦然。根据理论分析和子问卷的相关情况，提出另外两个 3 因子的竞争模型，其中模型 4 是将自我效能和乐观合并为一个因子；模型 5 是将乐观与韧性合并为一个因子。各模型的拟合指数见表 3。

表 3 验证性因子分析中各模型的拟合指数

Model	χ^2	*df*	χ^2/df	*RMSEA*	*ECVI*	*NNFI*	*CFI*	*IFI*
1	475.39	317	1.50	0.049	2.86	0.93	0.94	0.94
2	481.28	319	1.51	0.049	2.87	0.93	0.94	0.94
3	822.08	323	2.55	0.086	4.46	0.85	0.86	0.86
4	532.87	320	1.67	0.056	3.10	0.91	0.92	0.92
5	643.05	320	2.01	0.070	3.63	0.90	0.90	0.91

从表 3 中可见，模型 1 和模型 2 的 χ^2/df、*RMSEA* 、和 *ECVI* 值最小，而 *NNFI*、*CFI*、*IFI* 值最大，且都达到了良好拟合的标准。这说明相对于其他模型而言，4 因子模型及其高阶因子模型具有更好的解释力。

3.3 问卷的内部一致性信度和区分效度

(C)1994-2022 China Academic Journal Electronic Publishing House. All rights reserved. http://www.cnki.net

表 4　子问卷得分的相关和信度

子问卷	*M*	*SD*	自我效能	韧性	希望	α系数
自我效能	5.04	0.88				0.86
韧性	4.38	1.09	0.37**/0.11*			0.83
希望	5.34	0.88	0.55**/0.41**	0.25**/0.01		0.80
乐观	4.76	0.86	0.56**/0.36**	0.50**/0.37**	0.43**/0.16*	0.76

注：/号后为偏相关系数，$^{**}p<0.01$，$^{*}p<0.05$，以下同。

采用 Cronbach α 系数作为问卷的内部一致性信度指标。4 个子问卷的 α 系数分别为 0.86，0.83，0.80 和 0.76；全问卷的 α 系数为 0.90。从表 4 中可见，4 个子问卷得分的简单相关系数均达到了显著性水平，且均为中度相关。在控制了其它变量的影响后，得到的偏相关系数均有一定程度的降低，基本上都在 0.4 以下，其中韧性和希望两个子问卷得分的偏相关系数接近于 0。上述结果显示，积极心理资本问卷（*PPQ*）具有较好的内部一致性信度，各子问卷也具有合理的区分效度。

表 4 中的第二列和第三列数据是大学生被试在 4 个子问卷上得分的均值和标准差。由于问卷采用的是 7 点评定 Likert 量表形式，可见大学生的心理资本总的来说是较为积极和正面的。

3.4　心理资本与心理健康的关系

以 Rosenberg 的自尊量表（*SES*）、Bradburn 的情感量表（*AS:PNA*）以及 Judge 的核心自我评价量表（*CSES*）和症状自评量表（*SCL-90*）的若干分量表（焦虑、抑郁、偏执、人际敏感）作为效标问卷，计算心理资本及其子成分同效标的相关，相关系数见表 5。空白部分表示相关不显著。

表 5　心理资本及其子成分与效标的相关系数

	自尊	自我效能	内控	外控	正性情感	负性情感	情感平衡	焦虑	抑郁	偏执	敏感
自我效能	0.51	0.53	0.36	−0.12	0.34	−0.27	0.39	−0.18	−0.24		−0.22
韧性	0.41	0.14		−0.22		−0.57	0.48	−0.45	−0.50	−0.41	−0.56
希望	0.56	0.36	0.39	−0.25	0.20	−0.31	0.36	−0.16	−0.29		−0.21
乐观	0.59	0.33	0.37		0.17	−0.42	0.40	−0.28	−0.35	−0.27	−0.36
心理资本	0.67	0.44	0.39	−0.22	0.27	−0.53	0.54	−0.37	−0.46	−0.25	−0.46

从表 5 中可见，心理资本及其子成分与自尊、自我效能、内控性、正性情感、情感平衡呈正相关，而与外控性、负性情感、焦虑、抑郁、偏执、敏感呈负相关。其中，心理资本与自尊、负性情感、情感平衡、抑郁、敏感的相关相对较高。就其子成分而言，自我效能、希望和自尊的相关度较高；韧性和负性情感、抑郁、敏感的相关度较高；乐观和自尊、负性情感、情感平衡的相关度较高。这些结果均符合预期。不过，PPQ 中的自我效能得分与 CSES 中一般自我效能得分的相关并不高(0.53)，这可能是由于 PPQ 和 CSES 中关于自我效能的项目都较少（在 *CSES* 中仅有 4 个），导致其自身测量信度不高的缘故。

将 SCL-90 中焦虑、抑郁、偏执、敏感四个分量表得分相加，作为心理症状的指标。再以自尊、内控性、情感平衡和心理症状作为心理健康的指标和因变量，分别以心理资本的 4 个子成分和心理资本总分为自变量，以性别为控制变量，采用逐步回归法进行多重回归分析。两组回归分析的标准化回归系数、R^2、F 值见表 6，空白部分表示回归系数不显著。

从表 6 中可见，在第一组回归中，心理资本的 4 个子成分对心理健康指标中的自尊、内控性和情感平衡有正向的预测作用，对心理症状有负向的预测作用，并联合解释了上述指标 47%、18%、28%和 31%的变异，回归方程和大多数回归系数均达

(C)1994-2022 China Academic Journal Electronic Publishing House. All rights reserved. http://www.cnki.net

表 6 心理资本及其子成分对心理健康指标的多重回归分析

		自尊	内控性	情感平衡	心理症状
第一组	性别			0.12*	
	自我效能	0.23**	0.18*		
	韧性	0.13*		0.40**	–0.55**
	希望	0.32**	0.29*	0.24**	–0.16*
	乐观	0.24**			
	R^2	0.47	0.18	0.28	0.31
	F	36.83	9.34	16.61	30.82
第二组	性别				
	心理资本	0.67**	0.36**	0.50**	–0.51**
	R^2	0.45	0.13	0.27	0.19
	F	85.01	15.22	37.34	23.82

到了显著性水平。在第二组回归中，心理资本总分对自尊、内控性和情感平衡也有显著的正向预测作用，对心理症状有显著的负向预测作用，并分别解释了上述指标 45%，13%，27%和 19%的变异。与以心理资本的 4 个子成分独立进行的单变量回归分析结果相比，心理资本总分对心理健康的解释力更强。这些结果显示了心理资本与心理健康水平的密切关系，同时也证明了积极心理资本问卷（*PPQ*）具有良好的实证效度。

4 讨论

心理资本这一概念最早出现在经济学和社会学的研究文献中，例如，经济学家 Goldsmith 曾用心理资本指代能够影响个体收入的一些个性特征，如自尊等 [19]。在积极心理学和积极组织行为的框架下，心理资本一词被赋予了新的含义，它特指由乐观、自我效能、韧性和希望组成的心理结构。Luthans 强调，资本（*capital*）一词除传统上用于经济和财政领域外，还用于代表人力资源的价值、资源（*asset*）和优势（*advantage*），如人力资本和社会资本 [11]。其中，人力资本是指个体通过接受教育或经验积累而逐步掌握的知识、技能及其他素质；社会资本则是指人的社会关系和社会声誉等，二者对个人和组织的绩效、发展和成功都具有重要影响。Luthans 认为，心理资本和人力资本、社会资本一脉相承，也是影响个体和组织的绩效、适应和成功的重要因素 [8]。

心理资本这一构念的提出，无疑具有理论上的合理性。首先，乐观、自我效能、韧性和希望都是具有积极倾向的概念；同时，它们之间又存在着密切的联系，例如，高自我效能的个体对未来的预期也相对乐观，在逆境中也更有韧性。基于这 4 个子成分间的相互影响和协同效应，形成了更高层次的构念——心理资本[8]。在本研究的探索性因子分析中发现，如果对所得的因子负荷矩阵不加旋转，将能够提取一个“积极性”因子，而且各个项目也都在该因子上有较高的负荷；在验证性因子分析部分，高阶因子模型的拟合效果很好；而且 4 个子成分的测量也都具有显著的相关性，这些结果都为心理资本这一构念提供了初步的实证效度。类似地，Luthans 等人最近的研究也支持了高阶因子的存在 [11]。

Luthans 等人在心理资本概念的建构和测量过程中，特别强调了其成分的状态性或类状态性，即是发展的，可变的和可开发的。从培训和应用的角度，这样定义无疑具有很大的吸引力。但某种心理结构，究竟是特质还是状态，主要取决于其稳定程度和发展变化的开放性。从已有研究来看，也有一些学者认为乐观、韧性等是个体相对稳定的心理特点 [20,21]。因此，本研究在对心理资本进行测量时，并没有像 Luthans 等人那样强调其类状态的特点，这也是开发积极心理资本问卷（*PPQ*）的原因之一。当然，这并不表明包括心理资本在内的积极心理品质是难以训练和开发的。例如，Seligman 和 Peterson 等人的研究就显示，积极性干预的效果有充分的实证效度证据 [22]。

本研究中的探索性因子分析结果显示，积极心理资本问卷（*PPQ*）具有良好的结构效度，几乎所有项目的因子负荷都在 0.5 以上（平均 0.64），项目区分度也都在 0.6 以上（平均 0.71）。验证性因子分析的结果也显示，4 因子模型和高阶因子模型具有良好的拟合效果。积极心理资本问卷（*PPQ*）4 个子问卷的的 α 系数分别为 0.86、0.83、0.80 和 0.76，全问卷的 α 系数为 0.90，显示它具有良好的内部一致性信度，且略高于 PCQ 的信度指标 [13]。子问卷得分的相关系数在 0.25~0.56 之间，显示对心理资本 4 个子成分的测量具有合理的区分效度。

对心理资本与心理健康关系的考察显示，心理资本与自尊、内控性、情感平衡、心理冲突等反映心理健康水平的指标具有显著的相关，心理资本的 4 个子成分对上述指标的方差解释率在 18%~47% 之间，表明心理资本对心理健康有显著的积极影响。同时，心理资本及其子成分与自尊、内控性、

(C)1994-2022 China Academic Journal Electronic Publishing House. All rights reserved. http://www.cnki.net

正性情感、负性情感、情感平衡、焦虑、抑郁、偏执、敏感等多种效标都有显著的相关，而且相关的强度和方向也合乎预期，证明积极心理资本问卷（*PPQ*）具有较好的实证效度。

本研究中另一个值得关注的发现是：个体对自己的积极评价和消极评价表现出一定的分离，即二者并不是高度相关的。例如，在初始问卷对乐观的测量中，选取了 Scheier 和 Carver 开发的乐观量表中的若干项目，但因子分析的结果显示，个体对未来的乐观和悲观预期总是被分为两个因子。子问卷和全问卷的因子分析结果都显示，“我总是预期会有最好的结果”，“我总是看到事物好的一面”等项目与“事情很少按照我所期盼的方式发展”，“我很少指望好事发生在我身上”等项目分属两个相对独立的因子。由此看来，本研究的结果更支持 Marshall 和 Fred 的二因素乐观模型[23]。在对自我效能的测量中，也有类似的情况出现，而且反映个体对未来悲观预期的项目和反映低自我效能的项目常常汇聚在一个因子下面，这可能是由于这些项目都测量了个体的负性自尊。以上分析表明，心理资本的每个子成分都是复杂的甚至是多维的心理结构。尽管因子分析的结果显示，心理资本的 4 因子模型和高阶因子模型都具有良好的拟合，但这并不意味着心理资本的 4 种子成分一定是平行的关系，它们之间是否存在更为复杂的因果关系，还需要在今后的研究中进一步探索。总的来看，作为积极心理学领域的一个较新构念，心理资本在理论与实践、测量与开发方面都需要更多、更深入的研究。

5 结论

本研究的结果表明：积极心理资本问卷（*PPQ*）具有良好的信度和效度指标；积极心理资本这一构念的合理性得到了探索性因子分析和验证性因子分析结果的支持；积极心理资本与个体的心理健康存在着密切的关系。

参 考 文 献

1 Seligman M. The president's address. American Psychologist, 1999, 54 (8) : 559–562

2 Seligman M. Authentic happiness. New York: Free Press, 2002

3 Seligman M, Csikszentmihalyi M. Positive psychology: An introduction. American Psychologist, 2000, 55 (1) : 5–14

4 Gable S, Haidt J. What (and why) is positive psychology? Review of General Psychology, 2005, 9 (2) : 103–110

5 Linley P A, Joseph S. Positive psychology in practice. New Jersey: John Wiley&Sons, 2004

6 Luthans F. The need for and meaning of positive organizational behavior. Journal of Organizational Behavior, 2002, 23 (6) : 695–706

7 Luthans F. Positive organizational behavior: Developing and managing psychological strengths. Academy of Management Exective. 2002, 16 (1) : 57–72

8 Luthans F, Youssef C, Avolio B. Psychological capital. New York: Oxford University Press, 2007

9 Luthans F, Youssef C. Human, social, and now positive psychological capital management: Investing in people for compitvie advantage. Organizational Dynamics, 2004, 33 (2) : 143–160

10 Luthans F, Luthans K W, Luthans B C. Positive psychological capital: Beyond human and social capita1. Business Horizons, 2004, 47: 45–50

11 Luthans F, Avolio B, Avey J, et al. Psychological capital: measurement and relationship with performance and satisfaction. Personnel Psychology, 2007, 60 (3) : 541–573

12 Luthans F, Avolio B, Walumbwa F, et al. The psychological capital of Chinese workers: Exploring the relationship with performance. Management and Organization Review, 2005, 1 (2) : 249–271

13 Avcy J, Patera J, West B. The implications of positive psychological capital on employee absenteeism. Journal of Leadership and Organizational Studies, 2006, 13 (2) : 42–60

14 Jensen S M, Luthans F. Relationship between entrepreneurs' psychological capital and their authentic leadership. Journal of Managerial Issues, 2006, 18 (2) : 254–273

15 Larson M, Luthans F. Potential added value of psychological capital in predicting work attitudes. Journal of Leadership and Organizational Studies, 2006, 13 (1) : 45–62

16 Iuthans F, Youssef C M, Avolio B J. 心理资本：打造人的竞争优势. 李超平译. 北京：中国轻工业出版社, 2008

17 王雁飞, 朱瑜. 心理资本理论与相关研究进展. 外国经济与管理, 2007, 29 (5) : 32–39

18 仲理峰. 心理资本对员工的工作绩效、组织承诺及组织公民行为的影响. 心理学报, 2007, 39 (2) : 328–334

19 Goldsmith A, Venm J, Darity W. The impact of psychological and human capital on wages. Economic Inquiry, l997, 35 (4) : 815–829

20 温娟娟, 郑雪, 张灵. 国外乐观研究述评. 心理科学进展, 2007,

(C)1994-2022 China Academic Journal Electronic Publishing House. All rights reserved. http://www.cnki.net

15 (1) : 129–133

21 于肖楠, 张建新. 韧性 (resilience) —在压力下复原和成长的心理机制. 心理科学进展, 2005, 13 (5) : 658–665

22 Seligman M, Steen T, Park N, et al. Positive psychology progress: Empirical validation of interventions. American Psychologist, 2005, 60 (5) : 410–421

23 Fred B B, Jamie A C. Distinguishing hope and optimism: two sides of a coin, or two separate coin. Journal of Social and Clinical Psychology, 2004, 23 (2) : 273–302

POSITIVE PSYCHOLOGICAL CAPITAL: MEASUREMENT AND RELATIONSHIP WITH MENTAL HEALTH

Zhang Kuo[1], Zhang Sai[2], Dong Yinghong[1]

(1 Department of Social Psychology, Nankai University, Tianjin 300071; 2 The Affiliated Hospital of Medical College of Chinese People's Armed Police Force, Tianjin 300162)

Abstract

Positive Psychological Capital (*Psycap*) is defined as an individual's positive psychological construct which consists of four dimensions: self–efficacy, resiliency, optimism and hope in Positive Psychology and Positive Organizational Behavior. The Positive Psycap Questionnaire (*PPQ*) has been developed in this research and about 530 undergraduates of 3 samples participated in the survey. The results of EFA and CFA indicate PPQ has favorable construct validity, and Psycap, the composite higher–order factor, is proved to be a rational psychological construct. All of the Cronbach α coefficients in PPQ are sufficient. The Psycap significantly correlate with its criterions such as self–esteem, locus of control, positive affect and so on. The regression analysis show Psycap has great effect on mental health. All above results suggest that The PPQ is a capable instrument.

Key words positive psychology, psychological capital, measurement, mental health.

(C)1994-2022 China Academic Journal Electronic Publishing House. All rights reserved. http://www.cnki.net
