# Supplementary material for: A study on the current status and influencing factors of psychological capital among medical undergraduates
Source: Front Psychol. 2026 Oct 30;17:1830730. doi: 10.3389/fpsyg.2026.1830730 (PMC13759799; doi:10.3389/fpsyg.2026.1830730)
Supplement: Supplementary file 1 [file Data_Sheet_1.ZIP › References PDF/Psychological capital and CanMEDS core competence of medical students.pdf]

医学生心理资本与医师核心能力相关性分析

刘嘉祯,王道珍,陆耀红,张 锋,邹 扬,李 萍*
(上海交通大学附属第六人民医院教学办公室,上海 200233)

[摘 要] 目的 通过调研医学生心理资本、核心能力的现状,挖掘积极心理资本对其能力培养的影响作用。方法 应用 Luthas 心理资本问卷和 CanMEDS 核心能力自评量表对 168 名医学生进行调查,所得数据采用 SPSS 17.0 软件进行分析。结果 医学生心理资本平均得分是 102.20 ± 15.31 分,本科医学生心理资本得分低于住院医师($P = 0.048$)。医学生 CanMEDS 核心能力得分是 4.41 ± 0.63 分,本科生得分低于住院医师($P < 0.001$)。医学生心理资本与 CanMEDS 核心能力呈正相关性($R = 0.752$,$P < 0.001$)。结论 医学生的心理健康需要得到积极关注;提高心理资本是提高核心能力的一种途径;关注多维度教育,培养符合时代要求的医师。

[关键词] 医学生;心理资本;核心能力;CanMEDS
DOI: 10.16770/J.cnki.1008-9985.2017.10.025
[中图分类号] R 197.32 [文献标识码] A [文章编号] 1008-9985(2017)10-0974-04

Psychological Capital and CanMEDS Core Competence of Medical Students

LIU Jiazhen, WANG Daozhen, LU Yaohong, ZHANG Feng, ZOU Yang, LI Ping* (Teaching Department, No.6 People's Hospital Affiliated to Shanghai Jiao Tong University, Shanghai 200233)

[Abstract] **Objective** To investigate the status quo of psychological capital and core competence of medical students so as to figure out their correlation. **Methods** Luthas psychological capital questionnaire and CanMEDS core competence self-evaluation questionnaire were used to investigate 168 medical students. The data were analyzed by SPSS 17 software. **Results** The average score of psychological capital of medical students was 102.20 ± 15.31, and the score of psychological capital of undergraduate medical students was lower than that of residents ($P = 0.048$). The score of CanMEDS core competence of medical students was 4.41 ±0.63, and the scores of undergraduates were lower than those of residents ($P < 0.001$). There was a positive correlation between psychological capital and CanMEDS core competence of medical students ($R = 0.752$, $P < 0.001$). **Conclusion** Mental health of medical students should be paid more attention to. Improvement of psychological capital is a way to improve the core competence. Multi-dimensional education is critical to train doctors who meet the requirements of the times.

[Key words] medical student; psychological capital; core competency; CanMEDS
Hosp Admin J Chin PLA, 2017, 24(10): 974-976, 988.

医学生作为一个相对特殊的社会群体[1],在从单纯的理论学习进入到临床高节奏的实习转化中,可能更容易产生心理健康问题[2]。如果不能适当处理紧张心理状态,容易导致医学生对学业的兴趣降低或对未来职业规划的不确定,最终导致医学生在择业时选择离开医疗行业等现象。在获得同等教学资源的前提下,提高心理健康可以提高职业核心能力[3]。本研究旨在通过调研医学生心理资本和核心能力的现状,挖掘积极心理资本对其能力培养的影响作用,为开展有针对性的心理健康培训,间接提高医学生的核心能力,提升医疗质量,保证患者安全提供理论依据。

[基金项目] 上海市第六人民医院 2016 年度院级管理类科研基金
[作者简介] 刘嘉祯,女,硕士,初级,科员;电话:13564274759
[通信作者] 李 萍,E-mail: lipingxu365@163.com

1 研究背景

1.1 心理资本 心理资本(psychological capital)2004 年诞生于积极组织行为学,指能够导致个体积极组织行为的心理状态,是除了财力、人力、社会三大资本以外的第四大资本。人的潜能是无限的,而其根源在于人的心理资本[4];其包含效能(成功的信心)、希望(意志和途径)、韧性(复原与超越)、乐观(现实而又灵活)4 个成分,且 4 个成分具有协同效应[5]。心理资本与组织支持感是员工取得高绩效的必要条件[6]。拥有过人的心理资本的个人,能承受挑战和变革,可以成为成功的医师,在团队中可以成为管理者,从逆境走向顺境,从顺境走向更大的成就。自信、乐观、坚韧的人,勇于创新,敢于创新,能够因地制宜地将知识和技能发挥到最大限度[7]。此外,心理资本可通过后天努力获得

中国知网 https://www.cnki.net

开发和提高[8]。

1.2 医学生核心能力 加拿大医师能力框架（Canada Medical Education Direction System, CanMEDS, 2015 版）是加拿大皇家内科和外科医师学会（Royal College of Physicians and Surgeons of Canada, RCPSC）提出的医师能力框架，指出医师应该扮演 7 种不同角色，即医疗专家（Medical Expert）、交流者（Communicator）、协作者（Collaborator）、领导者（Leader）、健康促进者（Health Advocate）、研究者（Scholar）和专业者（Professional）。

我国和国际上一些医学教育组织对医学生核心能力评价也提出自己的见解，比如国际医学教育组织制订的全球医学教育最基本标准，即全球医学教育最基本要求（Global Minimum Essential Requirements in Medical Education），将医学生核心能力分为 7 个领域 60 条标准[9]；美国的 ACGME 以"结果为基础"，确定医师必须具备的六大能力[10]。我国教育部在 1998 年颁布的《普通高校本科专业介绍》和中国医科大学等 17 所院校在 2000 年校协作项目制订的培养目标中也对医学生核心能力进行定义[11]。

由于加拿大的国情与中国相类似，CanMEDS 框架基本满足医学模式的转变和医疗实践的挑战对医师能力提出的新要求[12]，且于 2015 年进行重大更新，因此本课题以此框架为基础，构建适合中国医学生的核心能力评价体系。

2 对象与方法

2.1 研究对象 采用简单随机抽样方法，在自愿配合的原则下，抽取上海交通大学附属第六人民医院临床医学院临床医学专业本科生及在该院规培的住院医师作为研究对象。共发放问卷 180 份，最终回收有效问卷为 168 分，问卷回收率和有效率分别为 100% 和 93.33%。其中男生 78 人（46.43%），女生 90 人（53.57%）；本科生 91 人（54.17%），住院医师 77 人（45.83%）。

2.2 研究方法 本研究采用问卷调查法对研究对象的心理资本及 CanMEDS 核心能力进行评估。

2.3 研究工具

2.3.1 Luthas 心理资本问卷[13] 问卷包括 4 个维度：自我效能、希望、韧性和乐观。采用 Likert6 级评分分别赋值 1～6 分，从"非常不同意"到"非常同意"，总分为 20～144 分，分数越高表明研究对象的心理资本水平越高。经检验，该量表的 Cronbach's α 系数为 0.920[14]，因而采用此问卷进行调查的结果是可信的。

2.3.2 CanMEDS 核心能力自评量表 以 CanMEDS 框架为基础，在参考有关文献及专家意见的基础上，编制医学生 CanMEDS 核心能力自评量表。量表共有 34 个条目，7 个维度：医疗专家、交流者、协作者、领导者、健康促进者、学者和专业素养，采用 Likert6 级评分分别赋值 1～6 分，从"非常不同意"到"非常同意"。通过内在一致性检验，该量表 Cronbach's α 系数为 0.963，KMO 抽样适度检验和 Bartlett's 球形检验结果显示，该问卷的 KMO 值为 0.933，各维度的信度及内容效度也较高（表 1），因而采用此问卷进行调查的结果是可信的。

表 1 CanMEDS 核心能力自评量表信度和效度

维 度	信度	内容效度	结构效度
医疗专家	0.921	0.853**	
交流者	0.916	0.892**	
协作者	0.779	0.854**	
领导者	0.843	0.836**	
健康促进者	0.872	0.828**	
学者	0.743	0.823**	
专业素养	0.719	0.620**	
总体	0.963		0.933**

** $P<0.01$

2.4 统计学方法 采用 Epidata 3.1 录入数据，以及采用 SPSS 19.0 统计软件进行分析，用平均值和方差对学生的心理资本和 CanMEDS 量表得分进行统计描述，利用相关性分析对结果进行统计推断，$P<0.05$ 为差异具有统计学意义。

3 结 果

3.1 医学生心理资本得分 表 2 可见，医学生心理资本得分是 102.20±15.31 分，最高值为 138 分，最低值为 46 分。本科医学生的心理资本得分低于住院医师（$P=0.048$），特别在乐观子维度（$P=0.010$）中的得分较低。其中 10 名（5.95%）学生的得分低于 80 分，需要加强和训练心理资本，以应对挑战和危机[14]。

3.2 医学生 CanMEDS 核心能力得分 表 3 可见，医学生 CanMEDS 核心能力得分是 4.41±0.63 分，住院医师在医疗专家（$P<0.001$）、交流者（$P<0.001$）、协作者（$P<0.001$）、领导者（$P=0.006$）和学者（$P=0.004$）角色上的得分高于本科生。随着学习、工作年限的增长，医学生的核心能力也在不断提升（$P<0.001$）。

3.3 心理资本与 CanMEDS 核心能力的相关性 图 1 可见，医学生心理资本与 CanMEDS 核心能力呈正相关性。通过 Pearson 相关性分析，两者的相关系数为 0.752（$P<0.001$），相关性较强。

表 4 可见，心理资本和 CanMEDS 核心能力子维度均具有中度以上的相关性，其中韧性、自我效能子维度与 CanMEDS 核心能力子维度相关性最高。心理资本与 CanMEDS 核心能力中专业素养子维度相关性最弱。

中国知网 https://www.cnki.net

表 2 医学生心理资本得分

项 目	总 体	本科生	住院医师	t	P
心理资本	102.20 ± 15.31	100.05 ± 16.84	104.73 ± 12.94	−1.991*	0.048
自我效能	25.71 ± 3.92	25.20 ± 4.33	26.32 ± 3.30	−1.858	0.065
希望	25.60 ± 4.26	25.40 ± 4.63	25.84 ± 3.80	−1.858	0.507
韧性	25.27 ± 4.62	24.67 ± 4.78	25.99 ± 4.34	−0.664	0.065
乐观	25.27 ± 4.69	24.78 ± 5.20	26.58 ± 3.80	−1.855*	0.010

* $P < 0.05$, $n = 168$

表 3 医学生 CanMEDS 核心能力评分

项 目	总 体	本科生	住院医师	t	P
核心能力	4.41 ± 0.63	4.21 ± 0.68	4.64 ± 0.48	−4.803**	<0.001
医疗专家	4.35 ± 0.74	4.06 ± 0.77	4.71 ± 0.52	−6.457**	<0.001
交流者	4.26 ± 0.87	3.97 ± 0.98	4.60 ± 0.57	−5.215**	<0.001
协作者	4.51 ± 0.71	4.32 ± 0.75	4.72 ± 0.59	−3.877**	<0.001
领导者	4.31 ± 0.83	4.16 ± 0.92	4.50 ± 0.67	−2.764**	0.006
健康促进者	4.49 ± 0.71	4.41 ± 0.79	4.59 ± 0.60	−1.672	0.096
学者	4.70 ± 0.62	4.29 ± 0.81	4.61 ± 0.61	−2.942**	0.004
专业素养	4.41 ± 0.63	4.62 ± 0.63	4.78 ± 0.60	−1.667	0.097

* $P < 0.05$, ** $P < 0.01$, $n = 168$

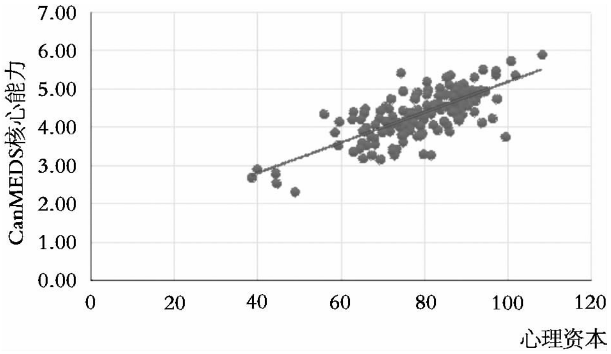

图 1 医学生心理资本 – 核心能力

表 4 心理资本和 CanMEDS 子维度相关系数**

类 别	自我效能	希望	韧性	乐观
医疗专家	0.500	0.488	0.575	0.570
交流者	0.523	0.563	0.620	0.589
协作者	0.612	0.574	0.613	0.540
领导者	0.605	0.519	0.596	0.432
健康促进者	0.636	0.595	0.619	0.499
学者	0.609	0.515	0.558	0.419
专业素养	0.418	0.422	0.437	0.393
核心能力	0.666	0.634	0.701	0.614

** $P < 0.01$

4 讨 论

4.1 医学生心理健康需要得到积极关注 大学生心理资本的均值为 5.17 分(量表评分中点值为 3.5 分)[15],医学生心理资本较低($\bar{x} = 4.25 \pm 0.38$)。作为大学生中一个特殊的群体,医学生的课业繁重,学制长,就业压力大,因此其心理健康需要得到更多的关注。由于本科生面临着单纯的理论学习进入到临床高节奏的实习转变,难以及时调整自己的状态,容易产生自我怀疑、消极的心理状态,因此其在乐观维度上的得分和住院医师相比较而言较低。部分媒体对医患矛盾的失实报道,容易造成本科生对行业的悲观情绪,这也会导致其乐观子维度的得分较低。

4.2 提高心理资本是提高核心能力的一种途径 研究表明,心理资本的各维度均与 CanMEDS 核心能力呈正相关性,心理资本的子维度自我效能、希望、韧性和乐观均对核心能力有较强的预测力。当学生相信通过自身的努力获得成功(自我效能)时,更能积极主动学习;当学生坚持自我去应对学习中的困难时(希望),便能够不断尝试克服瓶颈、找到正确的方法来应对困难;当学生在面对困难时,可以及时调整,且足够肯定自己的能力时(乐观),会更愿意尝试突破自己;当学生从逆境、冲突、失败、责任和压力中迅速自我恢复(韧性),便不惧挑战,更有勇气。因此,可以通过培养和提升学生的心理资本,从而提升医学生自我肯定的信念,杜绝消极畏惧情绪,培养积极心态,进而提升其核心能力。

4.3 关注多维度教育 培养符合时代要求的医师 从研究中可以看到,心理资本与 CanMEDS 核心能力中专业素养维度相关性不高($R = 0.393 - 0.437$),即仅仅依靠心理资本的提升,不能全面提高医学生的核心能力。高校、医院可以通过人文培养,以提高医学生的沟通能力,提高保护患者隐私、尊重患者文化的意识;通过开展团队合作的案例研究,提高学生相互合作,领导自我的能力;通过营造浓郁的教学氛围,培养学生终

(下转第 988 页)

中国知网 https://www.cnki.net

入需要综合考量的情境，设置开放性试题等方法对学生的医学思维进行考核或者对学生进行分模块考核，比如将考核分为理论考核、实践参与、创意比拼等模块，督促学生在学习过程中不断思考、体会与反思，弱化以往应试教育中考试成绩所占的比重，以激发学生对于医学本身而非考试的兴趣。

5 小 结

医学思维作为影响医学生素质与能力提升的一种软实力，对于医学生的成长与发展必不可少。然而目前医学教育上对于医学思维的培养意识有待加强，系统并不完善，应加强包括整体思维、创新思维、逆向思维、辩证思维和个体化思维在内的五种医学思维训练，需要在实际工作中进一步实践和探讨。

【参考文献】

[1] 孙景芬，邢辉，于淼. 医学思维方式的演变及创新思维刍议[J]. 理论界，2006(2)：136－137.

[2] 岳志斌，马冠峰. 试论如何推进大学医学教育[J]. 中国科教创新导刊，2013(22)：104.

[3] 黄利川. 探讨医学生临床思维中存在的问题与教育对策[J]. 中华全科医学，2008，6(10)：1056－1057.

[4] 赵丽微，辛程远，蔡建辉. 临床医学专业课程整合的探索与思考[J]. 重庆医学，2016，45(26)：3732－3733.

[5] 曹国强，余显礼. 专科医生应注重整体性和系统思维训练——由一例甲亢患者门诊长期误诊引发的哲学思考[J]. 医学与哲学，2009，30(18)：65－66.

[6] 郭斌，王莉，范钦华. 整合医学思维与眼底病诊治发展[J]. 医学争鸣，2016(3)：21－24.

[7] 吴元洁，王键，李净，等. 基于问题教学培养中医院校医学生高阶思维能力[J]. 安徽中医药大学学报，2016，35(2)：94－96.

[8] 刘虹. 整合：医学创新思维的重要形式[J]. 医学与哲学，2009，30(7)：13－15.

[9] 施源. 你身上的“新器官”：肠系膜[J]. 康复，2017(3)：10－11.

[10] 熊玮，戴晓天，杨和平. 逆向思维在医学领域的应用[J]. 现代医药卫生，2005，21(24)：3505－3506.

[11] 王东侠，戴力，张波. 浅谈女性冠心病认识中的辩证思维[J]. 医学与哲学，2016，37(14)：73－75.

[12] 陈贤楠. 个体化医疗与脓毒性休克指南：从个体化与规范化医疗谈起[J]. 中国小儿急救医学，2012，19(5)：449－451.

[13] 田建伟，赵连友. 高血压个体化治疗的哲学思考[J]. 医学与哲学，2002，23(12)：17－19.

(2017－07－05 收稿 2017－09－25 修回)

(本文编辑 胡友花)

(上接第 976 页)

身学习、参与教学的意识；通过举办公益性讲座、义诊等形式，让每位医学生担负起健康促进者的角色；加强思想道德教育，树立“敬畏生命”的职业自豪感。

5 结 语

医学生的自信、希望、韧性和乐观四种积极心理状态及合并而成的心理资本，都与其核心能力呈正相关，积极心理资本能够增加自信，有助于个人价值的实现。目前，医学已经转化成生物－心理－社会的模式[16]，医学生的教育，也相应地转化为知识理论－心理健康－社会支持相结合。

【参考文献】

[1] 黄蕾，马希权，荣海健，等. 医学生心理健康状况与相关影响因素研究[J]. 中华医学教育杂志，2014，34(2)：242－246.

[2] 姜绮霞，姚定康，梁春，等. 早期接触临床培养医学生医患沟通能力的探讨[J]. 西北医学教育，2011，19(2)：255－256，259.

[3] 张蔚，李哥. 团体心理训练在提升高职学生职业核心能力中的应用研究[J]. 科技资讯，2010(18)：219－220.

[4] 叶庆. 浅析心理资本在人力资源管理中的意义与应用[A]. 湖南省人力资源管理学会首届(2011)学术年会论文集[C]. 长沙：湖南省人力资源管理学会，2011：219－221.

[5] Luthans F，Luthans K W，Luthans B C. Positive psychological capital：beyond human and social capital[J]. Business Horizons，2004，47(1)：45－50

[6] 田喜洲，谢晋宇. 组织支持感对员工工作行为的影响：心理资本中介作用的实证研究[J]. 南开管理评论，2010，13(1)：23－29.

[7] 陈晨. 心理资本[M]. 北京：蓝天出版社，2008：20－55.

[8] Avey J B，Luthans F，Smith R M，at el. Impact of positive psychological capital on employee well－being over time[J]. Occup Health Psychol，2015，15(1)：17－28

[9] 国际医学教育组织. 全球医学教育最基本要求[S]. 1999－06－09.

[10] ACGME Outcome Project [EB/OL]. http：//www. acgme. org/Outcome，2011－02－15.

[11] 周继芳，朱德超，王绪伦，等. 新形势下医学生核心能力探析[J]. 中国医药导报，2012，9(5)：166－168.

[12] 冯攀. 加拿大医师能力框架对我国制定执业医师能力标准的启示[J]. 中国医院管理，2013，33(4)：61－63.

[13] Luigi Pedrabissi，J. P. Rollnad. Stress and Burnout Among Teachers in Italy and France[J]. The Journal of Psychology，127(5)：529－535.

[14] 王世英. 医患关系、心理资本对医生工作倦怠的影响[D]. 新乡：新乡医学院，2015：14－26.

[15] 王雁飞，李云健，黄悦新，等. 大学生心理资本、成就目标定向与学业成就关系研究[J]. 高教探索，2011(6)：128－136，148.

[16] 王衍晶，郭福玲. 顺应医学模式转变的全科医学理念与人才观[J]. 中华医学教育杂志，2012，32(6)：812－813，830.

(2017－06－20 收稿 2017－09－29 修回)

(本文编辑 胡友花)

中国知网 https://www.cnki.net
