# Supplementary material for: A study on the current status and influencing factors of psychological capital among medical undergraduates
Source: Front Psychol. 2026 Oct 30;17:1830730. doi: 10.3389/fpsyg.2026.1830730 (PMC13759799; doi:10.3389/fpsyg.2026.1830730)
Supplement: Supplementary file 1 [file Data_Sheet_1.ZIP › References PDF/Relationship between University Students' Positive Psychological Capital and Subjective Well-Being.pdf]

DOI:10.13342/j.cnki.cjhp.2012.07.035
中国健康心理学杂志 2012年第20卷第7期 China Journal of Health Psychology 2012, Vol 20, No. 7

大学生积极心理资本与主观幸福感的关系

唐家林① 李祚山△ 张小艳

【摘要】 **目的** 探讨大学生积极心理资本与主观幸福感的关系。**方法** 采用积极心理资本问卷和主观幸福感问卷,对重庆市3所大学的235名大学生进行调查。**结果** ①大学生积极心理资本状况总体较好,自我效能因子存在显著生源地差异($F=15.38, P<0.001$);自我效能因子存在显著专业类型差异($F=5.07, P<0.05$);希望因子存在显著专业类型差异($F=12.71, P<0.001$);希望因子存在显著年级差异($F=3.84, P<0.05$),进一步多重比较表明,四年级大学生的希望水平显著高于三年级大学生;②大学生主观幸福感状况总体不够理想,生活满意度因子存在显著生源地差异($F=11.22, P<0.01$);消极情感因子存在显著专业类型差异($F=8.27, P<0.01$);③大学生的积极心理资本和主观幸福感正相关,前者对后者各因子有显著的预测作用(预测效应介于16.2%~29%之间)。**结论** 大学生积极心理资本对主观幸福感有积极影响。

【关键词】 健康心理学;积极心理资本;主观幸福感;大学生

Relationship between University Students' Positive Psychological Capital and Subjective Well-Being. *Tang Jialin, Li Zuoshan, Zhang Xiaoyan. Applied Psychological Key Lab of Chongqing Normal University, Chongqing 401331, P.R.China*

【Abstract】 **Objective** To observe the relationship of positive psychological capital and subjective well-being of university students. **Methods** A total of 235 university students were tested using positive psychological capital questionnaire and subjective well-being questionnaire. **Results** ①Positive psychological capital of university students was generally well, there was significant difference of the self-efficacy level between students from rural and urban ($F=15.38, P<0.001$), there was significant difference of the self-efficacy level on subject categories($F=5.07, P<0.05$), there were significant differences in hope factor on subject categories($F=12.71, P<0.001$) and among grades ($F=3.84, P<0.05$), the hope level of senior students was higher than junior. ②Subjective well-being of university students was generally not ideal, there was significant difference of the life satisfaction level between students from rural and urban($F=11.22, P<0.01$), there was significant difference of the negative emotion level on subject categories ($F=8.27, P<0.01$). ③Positive psychological capital of university students was positively related to all of subjective well-being factors(16.2%~29%). **Conclusion** Positive psychological capital has positive effect on subjective well-being of university students.

【Key words】 Health psychology; Positive psychological capital; Subjective well-being; University students

主观幸福感,即Subjective well-being,简称SWB,是指个体根据自定的标准对其生活质量所作的整体性评价,是衡量个体生活质量的重要的综合性指标[1]。主观幸福感包括生活满意度和情感体验两个基本成分,前者是个体对总体生活质量的认知性评价;后者是个体生活中的情感性体验,包括积极情感(愉快、轻松等)和消极情感(压抑、愤怒、紧张等)[2]。因此,Diener认为主观幸福感由生活满意度、积极情感和消极情感3个部分构成[3]。随着近年来积极心理学的兴起,主观幸福感受到了研究者的极大关注。已有研究表明,外向性人格特征、个体自尊、社会支持和感恩等正性力量会对主观幸福感产生积极影响[4-7]。2004年Luthans等[8]在积极心理学和积极组织行为学的基础上提出了以个人积极心理力量为核心的积极心理资本的概念,并将其定义为个体在成长过程中表现出来的一种积极心理状态,它主要由自我效能、希望、乐观和韧性4个部分构成[9]。此概念一经提出,便受到心理学家和管理学家的高度重视。大量研究证实,积极心理资本对领导和员工的工作绩效、工作满意度、组织承诺、组织公民行为有促进作用[10-12];同时,积极心理资本对心理健康的各项指标也有显著的预测能力[13]。从理论上分析,作为积极心理力量的积极心理资本会对主观幸福感产生积极的影响。然而,对积极心理资本和主观幸福感关系的研究尚不多见。为此,本研究将积极心理资本从组织行为情景转移到教育情境,考察大学生积极心理资本和主观幸福感的基本状况,探索积极心理资本和主观幸福感的关系。为研究积极心理资本对主观幸福感的影响提供依据,同时对于当前如何有效地开展大学生的心理健康教育也具有重要的现实意义。

1 对象与方法

1.1 对象 从重庆市3所大学以班级为单位随机抽取二~四年级共250名学生,进行集体施测,有效问卷235份(94%)。有效被试中,二年级59人,三年级54人,四年级122人;男生47人,女生188人;理科103人,文科132人;城镇102人,农村133人。

1.2 方法 采用积极心理资本问卷和主观幸福感问卷进行测量。

1.2.1 积极心理资本问卷(PPQ) 该问卷由张阔等编制,4个维度(自我效能、韧性、乐观、希望),共26个题项,采用7点评分,得分越高说明积极心理资本状况越好,问卷信效度良好[13]。本样本中各分问卷的内部一致性系数在0.72~0.82

① 中国,重庆师范大学应用心理学重点实验室 401331 E-mail:tjlok12345@126.com △通讯作者

(C)1994-2022 China Academic Journal Electronic Publishing House. All rights reserved. http://www.cnki.net

之间，总问卷的内部一致性系数为 0.87。

1.2.2 生活满意度量表(SWLS)　该量表由 Diener 等编制，共 5个题项，采用 7等级评分，从"完全不符合"到"完全符合"分别评定为 1～7级，得分越高表明生活满意度越高，量表具有较好的信效度[14]。本研究中该量表的内部一致性系数为 0.75。

1.2.3 积极情感消极情感量表(PANAS－R)　该量表由邱林等[15]修订，包括积极情感和消极情感体验描述词各 9个，要求被试用 1～5评价在过去 1周里他们在多大程度上体验到了这些词汇所描述的情感，积极情感和消极情感得分分别为 9个项目上得分的均分。实测中积极情感量表的内部一致性系数为 0.90，消极情感量表的内部一致性系数为 0.71，总量表的内部一致性系数为 0.76。

1.3 数据处理　采用 SPSS 16.0统计软件对数据进行分析处理。

2 结　果

2.1 大学生积极心理资本和主观幸福感的现状　对被试大学生的积极心理资本和主观幸福感进行描述性统计分析，见表 1。自我效能、韧性、希望和乐观 4个因子的平均分都在量表中值 4以上，说明大学生积极心理资本状况较好；除了在消极情感因子上，大学生平均得分较好外，大学生生活满意度平均得分小于量表中值 4，积极情感平均得分小于量表中值 3，说明大学生主观幸福感状况不够理想。

2.2 大学生积极心理资本和主观幸福感的人口学变量差异分析　采用多元方差分析，考察性别、年级、生源地及专业类型对大学生积极心理资本和主观幸福感的影响，见表 2。自我效能因子存在显著的生源地差异(F = 15.38，P < 0.001)，城镇大学生的自我效能显著高于农村大学生的自我效能；自我效能因子存在显著的专业类型差异(F = 5.07，P < 0.05)，文科大学生的自我效能显著高于理科大学生的自我效能；希望因子存在显著的年级差异(F = 3.84，P < 0.05)，进一步多重比较表明，四年级大学生的希望水平显著高于三年级大学生的希望水平；希望因子存在显著的专业类型差异(F = 12.71，P < 0.001)，文科大学生的希望水平显著高于理科大学生的希望水平；生活满意度因子存在显著的生源地差异(F = 11.22，P < 0.01)，城镇大学生的生活满意度显著高于农村大学生的生活满意度；消极情感因子存在显著的专业类型差异(F = 8.27，P < 0.01)，理科大学生的消极情感显著多于文科大学生的消极情感；城镇大学生得分除了在自我效能因子和消极情感因子上，显著优于农村大学生外，在其他所有因子上，城镇大学生的得分均略高于农村大学生。

表 1　大学生积极心理资本和主观幸福感的现状

因　子	$\bar{x} \pm s$
自我效能	4.52±0.78
韧　性	4.27±0.99
希　望	5.47±0.91
乐　观	5.00±0.91
生活满意度	3.59±0.96
积极情感	2.79±0.79
消极情感	1.90±0.53

表 2　大学生积极心理资本和主观幸福感的人口学变量差异($\bar{x} \pm s$)

	性别		年级			生源地		专业类型	
	男	女	二	三	四	农村	城镇	文科	理科
自我效能	4.71±0.73	4.47±0.79	4.51±0.82	4.46±0.75	4.54±0.78	4.35±0.77	4.74±0.75	4.62±0.75	4.39±0.80
F	3.46			0.23		15.38***		5.07*	
韧　性	4.42±0.96	4.23±0.96	4.25±1.02	4.26±1.04	4.28±0.90	4.18±0.94	4.38±0.97	4.26±0.96	4.27±0.97
F	1.45			0.02		2.42		0.01	
希望	5.29±0.91	5.52±0.91	5.40±0.92	5.23±0.90	5.62±0.90	5.39±0.85	5.59±0.99	5.66±0.87	5.24±0.92
F	2.67			3.84*		2.89		12.71***	
乐观	4.90±1.00	5.02±0.89	4.97±0.82	5.00±0.85	5.02±0.99	4.94±0.91	5.07±0.92	5.09±0.95	4.88±0.85
F	0.71			0.06		1.04		3.23	
生活满意度	3.60±1.03	3.58±0.95	3.48±0.96	3.64±0.94	3.66±0.97	3.41±0.92	3.82±0.97	3.63±0.97	3.54±0.96
F	0.02			0.51		11.22**		0.50	
积极情感	2.67±0.83	2.82±0.78	2.91±0.75	2.59±0.82	2.82±0.78	2.71±0.78	2.90±0.79	2.86±0.79	2.70±0.78
F	1.50			2.49		3.58		2.41	
消极情感	1.94±0.65	1.90±0.50	1.93±0.55	1.90±0.58	1.90±0.50	1.92±0.53	1.88±0.52	1.81±0.50	2.02±0.55
F	0.28			0.07		0.28		8.27**	

注：* P < 0.05，** P < 0.01，*** P < 0.001，下同

2.3 大学生积极心理资本和主观幸福感的相关分析　为初步检验大学生积极心理资本和主观幸福感的关系，计算了二

(C)1994-2022 China Academic Journal Electronic Publishing House. All rights reserved.　http://www.cnki.net

者的相关，见表 3。大学生积极心理资本和主观幸福感之间有非常显著的相关关系，积极心理资本各因子和生活满意度正相关，相关系数介于 0.31～0.36之间；积极心理资本各因子和积极情感正相关，相关系数介于 0.36～0.42之间；积极心理资本各因子和消极情感负相关，相关系数介于 0.17～0.38之间。

表 3 大学生积极心理资本和主观幸福感的相关(*r*)

项 目	自我效能	坚 韧	希 望	乐 观
生活满意度	0.34**	0.33**	0.31**	0.36**
积极情感	0.42**	0.36**	0.39**	0.46**
消极情感	-0.17**	-0.38**	-0.25**	-0.19**

2.4 大学生主观幸福感对积极心理资本的回归分析 经过散点图检验线性关系，发现主观幸福感和积极心理资本呈线性相关，说明可以进行回归分析。为了进一步考察大学生积极心理资本对主观幸福感的影响，分别以主观幸福感的 3个因子为因变量，以积极心理资本 4个因子为自变量，采用逐步回归的方法建立回归模型，大学生积极心理资本与主观幸福感关系密切，前者对后者有一定的影响作用。乐观、自我效能和韧性能解释生活满意度因子 17.8%的变异；乐观、自我效能、韧性和希望能解释积极情感因子 29%的变异；韧性和希望能解释消极情感因子 16.2%的变异。

3 讨 论

3.1 大学生的积极心理资本状况 从总体上看，大学生的积极心理资本状况较好，可见他们自身有一定的积极心理力量，这和付立菲等[16]的研究结果一致。具体来说，在年级水平上，四年级大学生在希望因子上的得分最高，三年级大学生在希望因子上的得分最低，二者之间差异显著。这可能是因为四年级大学生即将离开学校，自我同一性延缓状态行将结束，他们会谨慎地确立自己的目标，并且坚定地去执行；三年级大学生由于学习任务相对较轻，他们开始思考自己的未来，在发现理想和现实的巨大反差时，可能会陷入迷茫。在生源地水平上，城镇大学生的积极心理资本状况优于农村大学生的积极心理资本状况，这种差异在自我效能上非常显著。这表明由于客观条件的限制，农村大学生的心理更脆弱，他们更容易体验到悲观失望的情绪，也更容易对未来丧失信心，潘清泉、周宗奎[17]对贫困大学生的研究在一定程度上支持了这一点。在专业类型水平上，文科大学生在希望因子上的得分显著高于理科大学生在希望因子上的得分。原因可能是，由于专业培养的不同，和理科生相比，文科生的个性更为温和、平静，做起事来更细致，一方面他们主动为自己设立目标，做好规划，另一方面他们持之以恒地追求并实现自己的目标。同时，文科大学生的自我效能强于理科大学生。可能的原因是，文科大学生大多以直觉形象思维为主，这种思维更具开放性、灵活性和发散性，导致他们内在情感体验更丰富、强烈，自我意识也更好。可见，年级、生源地和专业类型 3个变量对大学生积极心理资本都有一定的影响，且主要体现在自我效能和希望 2个因子上。

3.2 大学生的主观幸福感状况 从总体上看，大学生主观幸福感状况不容乐观，这一问题亟待解决。具体来说，在生源地差异上，城镇大学生的主观幸福感强于农村大学生的主观幸福感，这种差异在生活满意度因子上非常显著，这和严标宾等[18]的研究结果一致。原因可能是，城乡差距的客观存在导致城乡大学生对日常生活产生不同的体验，另一方面，农村大学生进城求学，他们必须改变过去的作息规律和生活方式，这样一个城市化的过程会给他们带来心理压力。在专业类型差异上，和姜永杰[19]的研究结果一样，文科大学生的主观幸福感强于理科大学生的主观幸福感，这种差异在负性情感因子上非常明显。这可能是因为理科注重抽象、逻辑思维能力，在学习中容易产生挫折感。

3.3 大学生积极心理资本与主观幸福感的关系 本研究发现，大学生的积极心理资本和主观幸福感有密切的正相关关系，符合研究假设。采用逐步回归分析表明，大学生积极心理资本对主观幸福感的预测效应达到非常显著的水平，它分别解释了生活满意度(17.8%)、积极情感(29%)、消极情感(16.2%)的变异。可见，大学生主观幸福感状况不容乐观，而积极心理资本状况相对较好，大学生积极心理资本对主观幸福感有显著的预测作用。鉴于目前对大学生积极心理资本和主观幸福感关系的研究不多，希望本文能够给广大心理健康教育工作者提供新的思路，以针对性地开发学生积极的心理力量为出发点，来提高他们的主观幸福感，最终达到心理和谐。

4 参考文献

[1]吴明霞. 30年来西方关于主观幸福感的理论发展[J]. 心理学动态，2000，8(4)：23-28

[2]Diener E. Subjective well－Being[J]. Psychology Bulletin，1984，95(3)：542-575

[3]Diener E. Assessing subjective well－being：Progress and opportunities[J]. Social Indicators Research，1994，31：103-157

[4]DeNeve K M. Happy as an extraverted claim? The role of personality for subjective well－being[J]. Current Direction in Psychological Science，1999，8(5)：141-144

[5]Diener E，Diener M. Cross cultural correlates of life satisfaction and self－esteem[J]. Journal of Personality and Social Psychology，1995，68：653-663

[6]严标宾，郑雪，邱林. 社会支持对大学生主观幸福感的影响[J]. 应用心理学，2003，9(4)：22-28

[7]Emmons R A，McCullough M E. Counting blessings versus burdens：An experimental investigation of gratitude and subjective well－being in daily life[J]. Journal of Personality and Social Psychology，2003，84(2)：377-389

[8]Luthans F，Youssef C. Human，social and now positive psychological capital management：Investing in people for competitive advantage[J]. Organizational Dynamics，2004，33(2)：143-160

[9]Luthans F，Luthans K W，Luthans B C. Positive psychological capital：Beyond human and social capital[J]. Business Horizons，2004，47：45-50

[10]Peterson S，Luthans F. The positive impact and development of hopeful leaders[J]. Leadership and Organizational Development Journal，2002，24：26-31

[11]Luthans K W，Jensen S M. The linkage between psychological capital and commitment to organizational mission：A study of nurses[J]. The Journal of Nursing Administration，2005，35：304-310

[12]仲理峰. 心理资本研究评述与展望[J]. 心理科学进展，2007，15(3)：482-487

[13]张阔，张赛，董颖红. 积极心理资本：测量及其与心理健康的关系

(C)1994-2022 China Academic Journal Electronic Publishing House. All rights reserved. http://www.cnki.net

医学院校大学生负性情绪与应对方式的相关研究

陈晓美[①] 罗红格 郭永芳 郭 鑫

【摘要】 目的 探讨医学院校大学生负性情绪与应对方式之间的关系。**方法** 采用简易应对方式问卷(SCSQ)、自评抑郁量表(SDS)以及焦虑自评量表(SAS)随机抽取某医学院校135名大学生进行测试。**结果** ①不同性别医学院校大学生在焦虑情绪($t=1.826$, $P<0.05$)、负性情绪($t=1.049$, $P<0.05$)存在显著差异,应对方式不存在显著差异;②医学院校心理学专业的学生与非心理学专业的学生在焦虑情绪($t=0.072$, $P<0.05$)、负性情绪($t=-0.698$, $P<0.05$)存在统计学差异,应对方式均不存在差异;③抑郁情绪、焦虑情绪以及负性情绪与积极应对存在显著负相关($P<0.01$),而与消极应对存在显著正相关($P<0.01$)。**结论** 医学院校男生焦虑情绪、负性情绪高于女生;心理学专业学生焦虑情绪高于非心理学专业学生,负性情绪低于非心理学专业学生。医学院校大学生应对方式影响其负性情绪。

【关键词】 健康心理学;医学院校大学生;负性情绪;应对方式

Relationship between Negative Emotions and Coping Style of Medical College Students. *Chen Xiaomei, Luo Hongge, Guo Yongfang, et al. Hebei United University, Tangshan 063000, P. R. China*

【Abstract】 Objective To explore the relationship between negative emotions and coping style of medical college students. **Methods** Using the simplified coping style questionnaire(SCSQ), self-rating depression scale(SDS) and the self-rating anxiety scale(SAS) to investigate 135 medical college students. **Results** ①The different gender medical students had significant differences in anxiety($t=1.826$, $P<0.05$) and negative emotions($t=1.049$, $P<0.05$), and not significant differences in coping style. ②The psychology and non-psychology students had significant differences in anxiety ($t=0.072$, $P<0.05$) and negative emotions($t=-0.698$, $P<0.05$), and not significant differences in coping style. ③Depression, anxiety and negative emotions were significantly negative correlation with actively coping ($P<0.01$), and significantly positive correlation with negative coping ($P<0.01$). **Conclusion** In medical university, the male college students are higher than the female college students in anxiety and negative emotions; The psychology students are higher than the non-psychology students in anxiety, while lower in negative emotions. The medical students' coping style has influence to their negative emotions.

【Key words】 Health psychology; Medical students; Negative emotions; Coping style

大学生是国家和民族的未来,医学院校大学生更是肩负着救死扶伤的重要使命,因此医学院校大学生在学习生活中面临着更多的压力和挑战,此时若不能合理应对则容易产生负性情绪。有研究显示,应对策略会在很大程度上影响负性情绪[1]。本文旨在探讨医学院校大学生负性情绪与应对方式之间的关系,以其为今后教育教学工作提供建议和参考。

1 对象与方法

1.1 对象 采用随机抽样的方法,在某医学院校选取大二、大三和大四3个年级的学生作为研究对象,包含应用心理学,精神卫生,临床医学,口腔医学,医学检验,药学等专业。共发放问卷150份,有效问卷率90%。被试主要特征如下:男生55人(40.7%),女生80人(59.3%);心理学专业47人(34.8%),非心理学88人(65.2%);大二39人(28.9%),大三43人(31.9%),大四53人(39.2%);农村88人(65.2%),城市47人(34.8%);独生子女38人(28.1%),非独生子女97人(71.9%);有恋爱经历的64人(47.4%);无恋爱经历的71人(52.6%)。

1.2 方法

1.2.1 简易应对方式问卷(SCSQ)[2] 简易应对方式问卷由积极应对和消极应对两个维度(分量表)组成,包括20个条目。问卷为自评量表,采用4级评分,此量表具有较高的信度和效度。

1.2.2 自评抑郁量表(SDS)[2]和焦虑自评量表(SAS)[2] 系William W K Zung分别于1965年、1971年编制的,均由20条目组成,按1~4级评分。

1.3 统计处理 问卷回收后,采用SSPS 11.5统计软件建立数据库,进行独立样本t检验和相关分析。以$P<0.05$为有统计学意义。

[J]. 心理与行为研究, 2010, 8(1): 58-64

[14] Pavot W, Diener E. Review of the satisfaction with life scale[J]. Psychological Assessment, 1993, 5: 164-172

[15] 邱林,郑雪,王雁飞. 积极情感消极情感量表(PANAS)的修订[J]. 应用心理学, 2008, 14(3): 249-254

[16] 付立菲,张阔. 大学生积极心理资本与学习倦怠状况的关系[J]. 中国健康心理学杂志, 2010, 18(11): 1356-1359

[17] 潘清泉,周宗奎. 贫困大学生心理资本、应对方式与心理健康的关系[J]. 中国健康心理学杂志, 2009, 17(7): 844-846

[18] 严标宾,郑雪,邱林. 家庭经济收入对大学生主观幸福感的影响[J]. 中国临床心理学杂志, 2002, 10(2): 118-119

[19] 姜永杰. 大学生主观幸福感问卷的编制及其与气质关系的研究[D]. 上海:华东师范大学, 2006

(收稿时间: 2012-01-15)

① 中国 河北联合大学心理学院(唐山) 063000

(C)1994-2022 China Academic Journal Electronic Publishing House. All rights reserved. http://www.cnki.net
