# Supplementary material for: A study on the current status and influencing factors of psychological capital among medical undergraduates
Source: Front Psychol. 2026 Oct 30;17:1830730. doi: 10.3389/fpsyg.2026.1830730 (PMC13759799; doi:10.3389/fpsyg.2026.1830730)
Supplement: Supplementary file 1 [file Data_Sheet_1.ZIP › References PDF/Relationship of Positive Psychological Capital and Learning Burnout in University Students. China Journal of Health Psychology.pdf]

DOI:10.13342/j.cnki.cjhp.2010.11.044

大学生积极心理资本与学习倦怠状况的关系*

付立菲①② 张 阔②

【摘要】 **目的** 探讨大学生积极心理资本与学习倦怠之间的关系。**方法** 采用积极心理资本问卷和学习倦怠问卷,对天津师范大学 389 名大学生进行调查。**结果** ①大学生积极心理资本状况:自我效能感因子在性别上具有显著性差异($t=3.99$,$P<0.05$);乐观因子在年级上具有显著性差异($F=3.96$,$P<0.05$),进一步多重比较表明,三年级大学生乐观水平显著高于二年级;乐观因子在学科类别上具有显著差异($t=5.49$,$P<0.05$);②大学生学习倦怠状况:情感耗竭因子在性别上具有显著性差异($t=10.99$,$P<0.01$);在学科类别上具有显著性差异($t=16.00$,$P<0.001$);在不同家庭住址上也具有显著性差异($t=3.50$,$P<0.05$);成就感低因子在年级上具有显著性差异($F=5.06$,$P<0.01$),进一步多重比较表明,一年级大学生成就感水平显著高于二、三年级大学生;③大学生的积极心理资本与学习倦怠负相关,前者对后者具有显著的预测效应(23.7%)。**结论** 大学生积极心理资本对学习倦怠有重要的影响,应当鼓励他们发挥自身积极心理资本优势,避免学习倦怠心理与行为。

【关键词】 积极心理资本;学习倦怠;大学生

Relationship of Positive Psychological Capital and Learning Burnout in University Students.*Fu Lifei,Zhang Kuo.Civil Aviation Management Institute of China,Langfang 065001,P.R.China*

【Abstract】 **Objective** To observe the relationship of positive psychological capital and learning burnout in University students. **Methods** A total of 389 university students were tested using positive psychological capital questionnaire and learning burnout questionnaire. **Results** ①There was significant difference of the self-efficacy level between boys and girls ($t=3.99$,$P<0.05$), there was significant difference of the optimism level among grades ($F=3.96$,$P<0.05$). After multiple comparisons,we found that the optimism level of junior students was higher than sophomore, there was significant difference in optimism factors on subject categories($t=5.49$,$P<0.05$). ②There was significant difference of the emotional exhaustion level between boys and girls($t=10.99$,$P<0.01$), there was significant difference of the emotional exhaustion level on subject category ($t=16.00$,$P<0.001$), there was significant difference of the emotional exhaustion level between students from towns and cities($t=3.50$,$P<0.05$), there was significant difference of the achievement level among grades($F=5.06$,$P<0.01$). After multiple comparisons,we found that achievement level of freshman was higher than sophomore and junior. ③Positive psychological capital of university students was negatively related to learning burnout(23.7%). **Conclusion** Positive psychological capital has important effect on learning burnout of the university students, they should be encouraged to play their positive psychological capital strengths, and avoid psychology and behavior of learning burnout.

【Key words】 Positive psychological capital;Learning burnout;University students

21 世纪是一个以知识竞争和科学技术突飞猛进为特点的信息化时代,培养人才是国家的重任。学生能否克服学习过程中消极因素的干扰,充分发挥自身积极心理因素的作用,提高学习效率成为教育者关心的问题。

在社会竞争日益激烈,社会压力不断增强的环境下,学生承受的压力也随之增大。不同阶段的学生群体中一些个体逐渐失去学习积极性和兴趣,学习动机不强烈,在学习过程中情绪低落,成就感低,旷课行为时常发生,即所谓的学习倦怠现象[1-2]。而大学期间专业知识和技能的掌握,对大学生走向社会,谋求生存尤为关键。如果出现学习倦怠,必然会严重地影响他们的学习质量和未来发展。个别研究已指出,大学生在一定程度上存在学习倦怠现象,尽管还没有达到严重化的程度,但是形势不容乐观[1]。不少学者也通过研究发现学习倦怠心理与行为、学习策略、专业承诺、乐观主义、专业适应性等因素之间存在一定的关系。近年来,积极心理学已经在人格、健康、临床、咨询和治疗等心理学分支领域产生了较大影响[3]。2004 年,Luthans 等[4]人在积极心理学和积极组织行为的框架下,提出了积极心理资本的概念,简称心理资本,并将其定义为,"个体在成长和发展过程中表现出来的一种积极心理状态",它包括四个核心成分:自我效能、乐观、韧性和希望[5]。此概念一经提出,便在心理学领域受到重视,相关的理论和应用研究也不断涌现。随着全球经济竞争日益激烈,已有研究表明,人力资本、心理资本在很大程度上影响着企业员工个体的工作绩效、价值观、组织承诺、组织公民行为,以及企业整体的发展和成功[6-9],从理论上分析,积极心理资本作为一种积极的心理取向,会对大学生的学习产生积极影响。然而,对积极心理资本及其各因子与学习倦怠关系的研究尚未见到。

为此,本研究拟将积极心理资本从组织环境引进教育环境,通过问卷调查,考察大学生积极心理资本和学习倦怠的基本情况,探讨积极心理资本与学习倦怠之间的关系,以便为大学阶段的教育实践提供一定的理论依据。

① 中国.民航管理干部学院教学区(廊坊) 065001 E-mail:fulifei85@tom.com ②南开大学社会心理学系
* 基金项目:天津市社科规划项目 TJJX062014
(C)1994-2022 China Academic Journal Electronic Publishing House. All rights reserved. http://www.cnki.net

1 对象与方法

1.1 对象 从天津师范大学以班级为单位抽取了一至三年级共 430 名学生，进行集体测试，回收有效问卷是 389 份，有效回收率为 90.47%，有效被试中，一年级 118 人，二年级 210 人，三年级 61 人；男生 157 人，女生 232 人；理工科 201 人，文科 188 人；来自城镇 166 人，城市 223 人。

1.2 研究方法 采用积极心理资本问卷和学习倦怠问卷进行测量。

1.2.1 积极心理资本问卷 该问卷由张阔编制，原问卷包含 26 个题目，4 个维度（乐观、韧性、自我效能感、希望），问卷的信、效度良好[10]。在本研究中，根据研究需要从问卷中抽取了 11 个题目，组成简版形式。

1.2.2 学习倦怠问卷 采用由朱林仙、马剑虹等[11]根据 MBI－GS 量表修订的学习倦怠问卷，该问卷信效度良好。对个别题目的文字表述略有修改，并删去了一个题目。测试问卷包括 15 个题目，3 个维度（去个性化、情感耗竭、成就感低），每个维度有 5 个题目。2 个问卷均采用利克特 5 点量表评分。

1.3 数据统计 本研究对所研究和收集的数据资料进行检查和校对后，采用 SPSS 13.0 软件进行分析和处理，使用了 t 检验方差分析以及逐步回归分析法。

2 结 果

2.1 大学生的积极心理资本状况 对被试大学生在积极心理资本及其各个维度值进行了统计，具体结果见表 1。从表 1 中可见，总的来说，大学生积极心理资本以及乐观、韧性、自我效能感、希望 4 个因子上的平均得分都在量表中值 3 以上，说明大学生积极心理资本状态较好。

表 1 大学生积极心理资本的均值和标准差（$\bar{x} \pm s$）

积极心理资本	乐 观	韧 性	自我效能感	希 望
3.49±0.58	3.65±1.01	3.29±0.76	3.15±0.73	3.79±0.70

对不同性别、年级、专业类型和生源地的大学生的积极心理资本进行比较，见表 2。结果发现：自我效能感因子上存在显著的性别差异（$t=3.99$，$P=0.046$，$P<0.05$），男生自我效能感的水平显著高于女生；乐观因子上存在显著的年级差异（$F=3.96$，$P=0.01$，$P<0.05$），三年级大学生乐观水平显著高于二年级大学生；乐观因子上存在显著的学科类别差异（$t=5.49$，$P=0.02$，$P<0.05$），理工科大学生乐观水平显著高于文科大学生；积极心理资本的各因子在生源地上不存在显著性差异，但是城市大学生得分均值略高于农村大学生。

表 2 性别、年级、专业类别和生源地积极心理资本值及其比较（$\bar{x} \pm s$）

	性别		年级			专业类别		生源地	
	男	女	1	2	3	文 科	理 工	乡 镇	城 市
乐 观	3.67±0.69	3.65±1.18	3.71±0.74	3.54±0.70	3.94±0.75	3.59±0.75	3.75±1.03	3.65±0.72	3.67±1.18
P		0.84		0.01*		0.02*		0.85	
韧 性	3.30±0.75	3.28±0.78	3.31±0.77	3.30±0.77	3.21±0.80	3.27±0.78	3.33±0.74	3.28±0.73	3.30±0.80
P		0.81		0.66		0.34		0.79	
效 能	3.24±0.78	3.08±0.70	3.24±0.76	3.07±0.72	3.23±0.73	3.16±0.74	3.28±1.20	3.10±0.74	3.18±0.74
P		0.046 ·		0.08		0.10		0.32	
希 望	3.74±0.72	3.81±0.69	3.81±0.65	3.79±0.71	3.72±0.78	3.86±0.68	3.82±0.69	3.74±0.64	3.82±0.75
P		0.33		0.71		0.39		0.31	

注：* $P<0.05$，* * $P<0.01$，* * * $P<0.001$，下同

2.2 大学生的学习倦怠状况 对被试大学生在学习倦怠及其各个维度上值进行了统计，具体结果详见表 3。从表 3 中可见，总的来说，大学生学习倦怠以及去个性化、情感耗竭、成就感低 3 个因子上的平均得分都在量表中值 3.0 以下，说明大学生学习倦怠情况良好，并没有达到严重化程度，与已有研究中的结果比较一致。

表 3 大学生学习倦怠的均值和标准差（$\bar{x} \pm s$）

学习倦怠	去个性化	情感耗竭	成就感低
2.49±0.64	2.49±1.03	2.43±0.84	2.53±0.62

对不同性别、年级、专业类型和生源地大学生的学习倦怠进行比较。结果发现：情感耗竭因子存在显著的性别差异（$t=10.99$，$P=0.00$，$P<0.01$），男生情感耗竭水平显著高于女生；成就感低因子存在显著的年级差异（$F=5.06$，$P=0.007$，$P<0.01$），二、三年级大学生的成就感水平显著低于一年级大学生；情感耗竭因子存在显著的学科类别差异（$t=16.00$，$P=0.000$，$P<0.001$），理工科情感耗竭水平显著高于文科；生源地不同在情感耗竭因子也存在显著差异（$t=3.50$，$p=0.046$，$P<0.05$），乡镇大学生情感耗竭水平显著高于城市大学生。

表 4 性别、年级、专业类别和生源地学习倦怠值及其比较（$\bar{x} \pm s$）

	性别		年级			专业类别		生源地	
	男	女	1	2	3	文 科	理 工	乡 镇	城 市
去个性化	2.55±1.24	2.44±0.85	2.50±1.26	2.42±0.90	2.70±0.93	2.40±0.60	2.52±0.69	2.54±0.63	2.44±0.65
P		0.32		0.16		0.15		0.11	
情感耗竭	2.60±0.95	2.31±0.74	2.45±0.95	2.40±0.82	2.51±0.71	2.29±0.94	2.53±1.17	2.57±0.96	2.43±1.07
P	0.001**		0.61		0.000***		0.043*		
成就感低	2.49±0.70	2.56±0.55	2.40±0.61	2.56±0.60	2.69±0.65	2.57±0.59	2.52±0.65	2.54±0.60	2.53±0.63
P		0.29		0.007**		0.28		0.96	

2.3 大学生积极心理资本与学习倦怠的相关分析 为初步检验大学生积极心理资本和学习倦怠之间的关系，计算了二

(C)1994-2022 China Academic Journal Electronic Publishing House. All rights reserved. http://www.cnki.net

者的相关，结果见表 5。

表 5　大学生学习倦怠和积极心理资本的相关(*r*)

	学习倦怠	去个性化	情感耗竭	成就感低
积极心理资本	-0.38**	-0.27**	-0.21**	-0.44**
乐　观	-0.16**	-0.11*	-0.09	-0.18**
韧　性	-0.27*	-0.20**	-0.24**	-0.19**
自我效能感	-0.18**	-0.11*	-0.07	-0.39**
希　望	-0.47**	-0.36**	-0.23**	-0.57**

从表 5 中可知，学习倦怠与积极心理资本之间有显著的负相关，相关系数介于 0.07～0.57 之间。除了情感耗竭因子与乐观、自我效能感 2 个因子间的相关不显著外，其余各个因子间的相关都比较显著。并且，积极心理资本中的希望和韧性 2 个维度与学习倦怠的去个性化、情感耗竭、成就感低 3 个维度之间相关相对比较高，达到极其显著的水平。

2.4　大学生学习倦怠对积极心理资本的回归分析　依据散点图检验线性关系，发现学习倦怠和积极心理资本呈线性相关，说明可进行回归分析。为了进一步考察大学生积极心理资本对学习倦怠的影响，分别以学习倦怠及其 3 个维度为因变量，以积极心理资本的 4 个维度为自变量，采用逐步回归的方法建立回归模型。回归分析的结果见表 6。

表 6　学习倦怠对积极心理资本的回归分析

					非标准化回归系数			
因变量	自变量	R	R^2	*F*	B	SE	标准化回归系数(β)	*t*
学习倦怠	希　望	0.49	0.24	61.37***	-0.39	0.04	-0.43	-9.13
	韧　性				-0.12	0.04	-0.14	-3.01
去个性化	希　望	0.37	0.14	30.22***	-0.47	0.07	-0.32	-6.49
	韧　性				-0.13	0.07	-0.10	-1.98
情感耗竭	韧　性	0.32	0.10	14.76***	-0.22	0.06	-0.20	-3.92
	希　望			-0.29	0.07	-0.24	-4.22	
	自我效能感				-0.18	0.06	-0.15	-2.28
成就感低	希　望	0.59	0.34	100.35***	-0.43	0.04	-0.49	-10.61
	自我效能感				-0.13	0.04	-0.16	-3.45

表 6 回归分析的结果表明，大学生的积极心理资本与学习倦怠之间有密切的关系，前者对后者有一定的影响作用。希望和韧性解释学习倦怠 23.7%的变异、去个性化因子 13.1%的变异，希望、韧性以及自我效能感因子共同解释情感耗竭 9.6%的变异，希望和自我效能解释成就感低因子 33.9%的变异。

3　讨　论

3.1　大学生学习倦怠的总体状况　本研究发现，从总体上看，大学生学习倦怠程度尽管并不十分严重，但是部分学生的得分略高些，显示出他们出现了这种倾向，这和连榕、朱林仙等[1,11]人的研究结果一致。

具体来说，男生情感耗竭水平高于女生。即男生在学习中因面临的一些过度要求，而表现出一种超负荷和耗竭的情感。原因可能在于男生认识到自己将来要靠专业技能立足社会，学习目标要求过高，当超出自身承受能力难以实现时就会出现身心疲惫的感觉。同时，理工科的大学生情感耗竭水平显著高于文科大学生。理工科专业比较枯燥，长期之下会影响他们的情绪甚至身心健康水平。乡镇大学生情感耗竭水平高于城市大学生。前者在学习上不甘示弱，甚至学习动机更强烈，但由于自身条件不优越，没有城市大学生多才多艺，当对自己要求过高不能顺利实现目标时，难免会出现情感的超负荷体验。

其次，一年级大学生成就感水平最高，他们显著高于二、三年级大学生的乐观水平，而二、三年级之间没有显著差异。原因可能与二、三年级大学生学习负担重，考试、就业压力比较大有关，他们常常会遭遇失败，挫折，导致自我成就感水平较低。

可见，性别、年级、学科类别以及生源地等因素都对大学生学习倦怠有一定的影响，并且主要体现在情感耗竭和成就感低 2 个因子上。

3.2　大学生积极心理资本的总体状况　本研究发现，从总体上看，大学生积极心理资本具有较好的水平，可见他们自身都具有一定的积极因素。具体来说，男生的自我效能感水平高于女生。这一结果和传统文化教育方式、教育理念相一致。无论家庭教育还是学校教育，男生都被看作是生活的强者，他们要勇敢、坚强、乐观、不能示弱、退缩，而女生则可柔弱、哭泣等。

其次，三年级大学生乐观水平最高，二年级乐观水平最低，两者之间差异显著。原因可能在于大学期间，一年级学习相对比较轻松，二年级压力最大，课程任务最繁重，三年级大学生经历了近三年的磨练，心理素质较好、心态平和，对未来充满信心。在自我效能感因子上一、二年级间差异显著，前者比后者自信。同时，理工科大学生乐观水平显著高于文科大学生，这个结果原因可能在于，在目前严重的就业形势下，理工科学生就业机会还是比较多的，相对文科专业的大学毕业生，他们对未来持一种乐观的态度。

而城市大学生与乡镇大学生在积极心理资本上没有显著差异，说明家庭地址不是大学生积极心理资本水平高低的重要影响因素。

可见，性别、年级、学科类别因素对大学生积极心理资本也有一定的影响，并且主要体现在乐观和自我效能感 2 个因子上。

3.3　大学生学习倦怠与积极心理资本的关系　本研究发现，大学生的积极心理资本水平和学习倦怠之间有密切的负相关关系，符合研究预想。为进一步考察积极心理资本对学习倦怠及其各因子的影响关系，采用逐步回归法分析结果表明，大学生积极心理资本对学习倦怠的预测效应达到非常显著的水平，它分别解释了学习倦怠、去个性化、情感耗竭、成就感低 23.7%、13.1%、9.6%、33.9%的变异。

可见，大学生积极心理资本与学习倦怠状况基本良好，并

(C)1994-2022 China Academic Journal Electronic Publishing House. All rights reserved.　http://www.cnki.net

中国军校大学生风险决策特点及军事氛围影响因素分析

黄志强[①]　刘旭峰[△]　周红燕　黄志平　张　杰　张　琰

【摘要】 目的　分析军校大学生不同信息框架下的决策行为特点,并探讨军事氛围的影响。**方法**　以整群抽样的方法抽取军校大学生高年级组(三、四年级生)101 名,低年级组(一年级生)99 名,并分别随机分成两组,对 Kahneman 的经典亚洲疾病问题决策选择情景进行二选一的选择。**结果**　中美大学生对于亚洲疾病问题的选择有差异,中国军校大学生在正面框架下选择风险规避和风险寻求的比例大致相等;高、低年级组比较,高年级组被试在正面框架和负面框架下选择风险寻求的比例分别是 60.4% 和 69.8%,差异没有显著性($\chi^2=0.982$,$P=0.322$);低年级组被试在正面框架和负面框架下选择风险寻求的比例分别是 43.1% 和 64.6%,差异有显著性($\chi^2=4.572$,$P=0.032$)。**结论**　无论正面框架或负面框架,与美国大学生相比,中国军校大学生更倾向于风险寻求;与低年级组相比,高年级组更倾向于风险寻求。

【关键词】　框架效应;决策;军校大学生;军校氛围

Influence of Military Environment on Risk Decision-making of Undergraduates. *Huang Zhiqiang, Liu Xufeng, Zhou Hongyan, et al. Department of Psychology, Faculty of Aerospace Medicine, Forth Military Medical University, Xi'an 710032, P.R.China*

【Abstract】 Objective　To examine the framing effect and risk decision-making on Chinese military undergraduate. **Methods**　By mean of cluster sampling, 200 military undergraduates were investigated with Kahneman's well-known Asian Disease framing problem, included 101 senior students, 99 junior students. Respectively, they were randomly divided into two groups. The first group received positive format, the second received negative format. **Results**　In senior students of the positive framing, 60.4% choose the risk-seeking and 69.8% choose the risk-seeking in the negative framing. A Chi-square test didn't reveal a significant difference($\chi^2=0.982$, $P=0.322$); in junior students of the positive framing, 43.1% choose the risk-seeking and 64.6% choose the risk-seeking in the negative framing. A Chi-square test revealed a significant difference($\chi^2=4.572$, $P=0.032$). **Conclusion**　Compared with American, Chinese military undergraduates are more risk-seeking, whether in positive or negative format; Compared with junior students, senior students are more risk-seeking, whether in positive or negative format.

【Key words】　Framing effect; Decision-making; Military undergraduates; Military environment

Kahneman 将心理学的研究结果引入经济学,从而获得了 2002 年诺贝尔经济学奖。他主要研究了个体在不确定条件下进行判断和决策的过程[1],提出著名的"前景理论"。框架效应是前景理论的核心概念之一,指由于语义描述的不同,决策

且本研究结果证实了我们的预想,二者之间成负相关关系,积极心理资本对学习倦怠有重要的影响。基于分析积极心理资本与学习倦怠之间关系的研究还比较缺乏,因此本研究结果对大学教育工作者有一定的理论指导作用,希望能够帮助教育者们针对不同性别、年级、专业类别以及生源地的大学生,采取不同措施,引导他们消除学习倦怠等不良心理与行为,同时鼓励他们充分发挥自身积极心理品质,学好专业知识和技能。

4　参考文献

[1]杨丽娴,连榕.当前大学生学习倦怠状况及其与专业承诺关系的研究[D].福州:福建师范大学,2004:1-38

[2]向晓蜜,张进辅.大学生学习倦怠的成因模型及其量表编制[D].重庆:西南大学,2008:1-54

[3]Gable S, Haidt J. What (and why) is positive psychology[J]? Review of General Psychology, 2005, 9(2):103-110

[4]Luthans F, Youssef C. Human, social, and now positive psychological capital management: Investing in people for compitvie advantage[J]. Organizational Dynamics, 2004, 33(2):143-160

[5]Luthans F, Luthans K W, Luthans B C. Positive psychological capital: Beyond human and social capita[J]. Business Horizons, 2004, 47:45-50

[6]仲理峰.心理资本对员工的工作绩效、组织承诺及组织公民行为的影响[J].心理学报,2007,39(2):328-334

[7]Luthans F B, Avolio B J, Walumbwa F O, et al. The psychological capital of Chinese workers: Exploring the relationship with performance[J]. Management and Organization Review, 2005, 1(2):249-271

[8]Luthans K W, AndJensen S M. The linkage between psychological capital and commitment to organizational mission: A study of nurses[J]. Journal of Nursing Administration, 2005, 35(6):304-310

[9]Larson M, Luthans F. Potential added value of psychological capital in predicting work attitudes[J]. Journal of Leadership and Organizational Studies, 2006, 13(1):45-62

[10]张阔.积极心理资本:测量及其与心理健康的关系[J].心理与行为研究,2010,8(1):58-64

[11]朱林仙,马剑虹.大学生心理压力-社会支持及其与学习倦怠的关系研究[D].杭州:浙江大学,2007:1-74

(收稿时间:2010-07-24)

① 中国,第四军医大学航空航天医学系心理学教研室(西安)　710032　△通讯作者　E-mail:lllfmmu@fmmu.edu.cn

(C)1994-2022 China Academic Journal Electronic Publishing House. All rights reserved.　http://www.cnki.net
