# Supplementary material for: A study on the current status and influencing factors of psychological capital among medical undergraduates
Source: Front Psychol. 2026 Oct 30;17:1830730. doi: 10.3389/fpsyg.2026.1830730 (PMC13759799; doi:10.3389/fpsyg.2026.1830730)
Supplement: Supplementary file 1 [file Data_Sheet_1.ZIP › References PDF/Research on the Relationship between Psychological Capital and Employment Ability among College Students.pdf]

DOI:10.16298/j.cnki.1004-3667.2011.03.021

（徐大伟，大连理工大学经济学院副教授，辽宁大连 116024；北京大学经济与人类发展研究中心研究员，北京 100080；段姗姗，大连理工大学经济学院硕士研究生，辽宁大连 116024；王佳宏，大连理工大学经济学院硕士研究生，辽宁大连 116024；范志刚，大连理工大学经济学院硕士研究生，辽宁大连 116024）

（致谢：本次问卷调查得到了大连理工大学、东北大学、辽宁大学、东北财经大学、大连海事大学、大连医科大学、大连舰艇学院、大连交通大学、沈阳农业大学、沈阳师范大学、沈阳大学、辽宁对外经贸学院部分师生的大力支持和积极合作，在此表示衷心地感谢！）

参考文献

[1] Guagnano G A, Stern P C, Dietz T. Influences on attitude-behavior relationships: A natural experiment with curbside recycling[J].Environment and Behavior, 1995(05):699-718.

[2] Poortinga W, Steg L, Vlek C. Values Environmental concern and environmental behavior: A Study into household energy use[J].Environment and Behavior,2004,36(01):70-93.

[3] Hungerford H R, Peyton R, Wilke R. Goals for curriculum development in environmental education[J]. Journal of Environmental Education, 1980,11(03):42-47.

[4] Sia A P, Hungerford H R, Tomera A N. Selected predictors of responsible environmental behavior: an analysis[J]. The Journal of Environmental Education, 1985/1986,17(02):31-40.

[5] Smith Sebasto, Ayre S, Daphne Goldman, Bela Yavetz,Sara Peer. Environrnental Literacy in Teacher Training in Israel: Environmental Behavior of new students[J].The Journal of Environmental Education, 2006,38(01):3-22.

[6] 任莉颖.环境保护中的公众参与——环境问题与环境意识[M].北京:华夏出版社,2001:89-113.

[7] Chan R Y K. Determinants of Chinese consumers' green purchase behavior[J]. Psychology & Marketing,2001,18(04):389-413.

[8] 郑时宜.影响环保团体成员三种环境行为意向之因素的比较[D].高雄:台湾中山大学,2004.

[9] 彭何芬.群体动力论在大学德育教育中的应用[J].湛江海洋大学学报,2002,22(05):82-87.

[10] 孟 雁.群体动力理论在高校案例教学中的启示与实践[J].现代教育,2006(19):66.

[11] 童建福,沈锐强.大学生群体行为的直观表象与内涵探究[J].福建农林大学学报,2008,11(02):83-86.

[12] 李昱星.学生的"从众心理"及其教学效应[J].中国职业技术教育,2003(35):25.

[13] 姚建银.对从众心理影响下大学生行为的探讨[J].甘肃联合大学学报,2009,25(03):81-83.

[14] 易丹辉.结构方程模型方法与应用[M].北京:中国人民大学出版社,2008:166.

大学生心理资本与就业能力关系研究*

励 骅 曹杏田

摘 要：采用心理资本量表和就业能力自评量表对 7 所本科院校 2500 名大学生进行调查，结果发现：大学生人际型心理资本和就业能力在性别和年级上差异显著；学生干部经历有利于提高大学生心理资本和就业能力；大学生心理资本与就业能力之间存在显著的正相关；心理资本对就业能力有很好的正向预测作用。

关键词：心理资本；就业能力；大学生

一、问题的提出

心理资本早期出现在管理学、经济学和社会学等相关文献里，国内比较认同的是 F. Luthans 的观点，他认为心理资本是个体在成长和发展过程中表现出来的一种积极心理发展状态，主要包括四个方面的内容：自信或自我效能感（confidence or self-efficacy）、希望（hope）、乐观（optimism）和坚韧性（resilience）[1]。柯江林的研究将心理资本划为事务型心理资本与人际型心理资本。事务型心理资本包括自信、乐观希望、奋发进取和坚韧顽强；人际型心理资包括谦虚诚稳、包容宽恕、尊敬礼让和感恩奉献[2]。根据赵冬的观点，就业能力是指获得和保持工作的能力，进一步讲就是个体能够满足组织需要，并且始终胜任工作的能力和特质[3]。就业能力受到个体环境、求职环境、社会经济政治环境等因素的影响。相关研究表明，心理资本是一种有助于工作绩效提高的积极心态，事务型、人际型心理资本与任务绩效、周边绩效正相关[4]。个体既需要事务型心理资本去设定任务目标，渡过困难和实现目标，也需要人际型心理资本去与他人沟通和协作。员工的希望、乐观和坚韧性三者合并而成的心理资本，对他们的工作绩效、组织承诺和组织公民行为有积极影响作用[5]。有关研究发现[4][5]，通过培训员工的心理资本，可以帮助员工缓解工作压力。

通过分析已有的研究，可以看出已有研究已经认识到心理资本的构成及其重要性。那么心理资本和就业能力是什么样的关系？心理资本对就业能力的哪些因素有影响？是否通过改变个体的心理资本构成可以改变个体的就业能力？这些问题值得研究探讨。

二、对象与方法

本研究以班为单位采用整群抽样的方式，抽取 7 所本科

* 本文系安徽省高校省级自然科学研究项目"心理资本与大学生就业能力实证研究"（项目编号：KJ2010A317）成果之一

(C)1994-2022 China Academic Journal Electronic Publishing House. All rights reserved. http://www.cnki.net

院校共 2500 人进行调查，其中男生 1230 人，女生 972 人；大一学生 568 人，大二学生 623 人，大三学生 520 人，大四学生 491 人；独生子女 968 人，非独生子女 1234 人；来自农村的大学生 1123 人，来自城市的大学生 1079 人；人文类学生 355 人，理工类学生 403 人，经济类学生 384 人，建筑类学生 393 人，农林类学生 237 人，师范类学生 308，其他类学生 122 人；共发放问卷 2500 份，回收 2230 份，剔除无效问卷，回收 2202 份，有效回收率 88.08%。

对大学生心理资本的测量，采用柯江林在《心理学报》上公布的心理资本短版量表，采用 Likert 5 点计分。题项采用正反向计分。转换过后分数越高表明心理资本状况越好，反之则相反。量表共 20 个项目，分为事务型和人际型心理资本两个维度。两个维度共 8 个因子。问卷有较好的信度，克隆巴赫系数为 0.76，分半信度系数为 0.84；效度方面，各因子与心理资本总分之间的相关系数均在 0.64-0.86（$p < 0.001$）之间。

就业能力问卷采用赵冬自编的就业能力自我评定量表，量表分为 5 个维度 31 个项目，采用与心理资本量表同样方式计分，分数越高则就业能力越强。该问卷已公布的内部一致性系数为 0.8，分半信度系数为 0.7，各维度信度系数均在 0.52-0.78 之间。本次实测结果显示，克隆巴赫系数为 0.89，分半信度系数为 0.84。结构效度方面，各维度与总分之间的相关系数均在 0.50-0.86（$p < 0.001$）之间。施测过程中主试均为心理学专业教师和研究生，整个测验过程符合标准化测验过程。运用 SPSS15.0 对所有数据进行统计分析。

三、结果与分析

（一）大学生心理资本和就业能力在相关变量上的比较

以性别、年级和学生干部为自变量，以心理资本和就业能力的总分及各维度为因变量，进行多元方差分析（见表 1）。

1. 大学生心理资本和就业能力的性别比较。在性别上，如表 1 所示，心理资本总分和人际型心理资本在性别上存在显著差异，而事务型心理资本在性别上不存在显著差异。就业能力总分在性别上存在显著的差异，就业能力的各个维度中，自我管理能力和沟通能力在性别上不存在显著差异，团结合作能力、问题解决和计划组织能力在性别上存在显著差异。

2.学生干部与非学生干部的心理资本和就业能力比较。根据调查结果，将学生分为是否担任过学生干部和非学生干部两类。通过比较他们在心理资本和就业能力及各维度的差异发现，在总分上这两类学生存在显著的差异；而在人际型心理资本维度上存在差异，在事务型心理资本维度上两者不存在显著差异；而在计划组织能力、自我管理能力和问题解决能力维度上，两类学生得分的差异不显著，而在团结合作和沟通能力维度上二者存在显著差异。

3. 大学生心理资本和就业能力的年级比较。通过方差分析，发现不同年级大学生心理资本总分和就业能力总分存在差异。如表 1 所示，不同年级学生心理资本与就业能力总分之间存在一定的差异。总体来讲，高年级学生的心理资本和就业能力均优于低年级的学生。进一步对不同年级学生的心理资本与就业能力进行方差分析（表 2），结果显示，大一年级学生的心理资本和就业能力与其他年级学生心理资本之间存在显著的差异，表明高年级学生在心理资本和就业能力方面均优于大一学生。而其他三个年级学生之间在均值上差异不显著。

表 2 各年级之间心理资本总分和就业能力多重比较检验结果（F 值）

年级	大一		大二		大三	
	心理资本	就业能力	心理资本	就业能力	心理资本	就业能力
大二	-22.32**	-14.79**				
大三	-21.32**	-15.23**	1.00	-0.44		
大四	-20.80**	-15.37**	1.52	-0.59	0.52	-0.14

** $p < 0.01$

（二）大学生心理资本与就业能力的关系分析

对大学生心理资本与就业能力进行相关分析，结果如表 3 所示。

表 3 大学生心理资本与就业能力的相关分析

	自我管理能力	团结合作能力	沟通能力	问题解决能力	计划组织能力	就业能力总分
事务型心理资本	0.590**	0.036	0.203	0.421**	0.498**	0.643**
人际型心理资本	0.676**	0.652**	0.568**	0.070	0.029	0.732**
心理资本总分	0.702**	0.661**	0.594**	0.493**	0.567**	0.762**

** $p < 0.01$

由表 3 可知，心理资本总分与就业能力各维度及就业能力总分存在显著的正相关，事务型心理资本与自我管理能力、问题解决能力和计划组织能力均存在显著的正相关，与团结合作能力和沟通能力不存在显著的相关。人际型心理资本与自我管理能力、团结合作和沟通能力存在显著的相关，与问题解决和计划组织能力不存在显著的相关。这表明心理资本的不同维度影响着就业能力的不同方面。

（三）心理资本对就业能力的多元回归分析

以就业能力总分作为因变量，以心理资本的两个维度作为自变量，检测心理资本的两个维度对就业能力的预测作用。采用逐步回归法进行多元回归分析，结果如表 4 所示。研究结果显示，事务型心理资本和人际型心理资本对就业能力均有正向预测作用。

表 1 不同类型大学生心理资本和就业能力的比较分析

	心理资本总分	事务型心理资本	人际型心理资本	就业能力总分	自我管理能力	团结合作能力	沟通能力	问题解决能力	计划组织能力
性别	4.11*	0.15	8.33**	9.89**	1.35	4.75*	3.36	18.73**	14.06**
年级	237.29**	147.76**	89.83**	310.09**	130.43**	111.56**	77.14**	49.50**	70.46**
学生干部	5.64*	3.57	5.60*	9.76**	3.46	15.51**	6.71**	2.28	3.47
性别×年级	1.28	0.25	1.26	0.09	0.40	0.92	0.52	0.32	1.58
年级×学生干部	1.62	0.80	2.46	2.12	1.15	0.88	3.07	2.79	0.27
性别×学生干部	0.00	0.02	0.10	0.46	0.41	0.11	0.15	0.12	1.78
性别×年级×学生干部	1.14	0.31	2.09	1.30	0.60	1.37	1.45	1.13	2.11

*$p<0.05$ **$p < 0.01$

四、思考与讨论

1. 大学生心理资本与就业能力的性别差异分析。心理资本的总体状况在性别上的统计结果存在一定的差异性，但可以看出心理资本总体状况的差异主要来源于人际型心理资本的差异，而事务型心

(C)1994-2022 China Academic Journal Electronic Publishing House. All rights reserved. http://www.cnki.net

表 4 心理资本与就业能力的多元回归分析

因变量	预测变量	R	R2	F	B	Beta	t
就业能力	方程模型	0.766	0.586	2536.219**			
	事务型心理资本				0.500	0.294	16.408**
	人际型心理资本				0.743	0.543	30.332**

** p <0.01

理资本在性别之间没有显著的差异，这与柯江林[4]的研究结果一致，人际心理资本与工作态度、绩效的相关性更大，表现的个体差异更大。同时这也表明心理资本状况的改变应在加强事务型心理资本培养同时，有区别地对人际型心理资本进行培养。就业能力在性别上的差异在各个维度上的表现不是一致的，总体来讲再就业能力在性别上的差异显著，主要表现在团结合作、问题解决和计划组织能力方面的差异，这与男女生不同的处事方式和个性差异相关，因此，有区别地对男女学生这方面能力的训练显得十分有必要。

2. 大一学生的心理资本和就业能力显著低于其他三个年级的学生，而其他三个年级的学生这两个因变量之间不存在显著的差异。这一研究结果表明，学生经过若干年的大学生活，心理资本得到明显的提高，体现出教育在人才培养中的重要作用。除大一学生以外其他三个年级的学生不存在显著差异，表明心理资本的相对稳定性，并非可以无限地提高。

3. 学生干部的经历能有效提高心理资本和就业能力。从结果中可看出，有学生干部经历的学生在人际型心理资本上的得分更高，这可能与他们有更多的机会接触教师、同学和社会人士相关，人际交往的技巧和应对策略无形中得到了更好的锻炼。而事务型心理资本相对来讲更加稳定，没有得到明显的提高。由于学生干部在校期间承担更多的事务，需要更多的团结合作和与人沟通，对自己的学习和工作有更高的要求，所以在就业能力的各个维度上，有学生干部经历的学生的表现要优于其他学生。这无疑可以看出，在校期间适当担任学生干部，无论是对心理资本，还是就业能力的提高都有很好的促进作用。

4. 心理资本和就业能力的关系。总体而言，心理资本和就业能力之间存在显著的相关。事务型心理资本包括自信、乐观希望、奋发进取和坚韧顽强这四个因子，在现实工作中体现在做事态度、意志力和自我效能等方面，而人际型心理资本涉及个体在人际交往和处理人际关系时所表现出来的心理状态。可以看出，人际心理资本与自我管理能力、团结合作和沟通能力之间的关系比较明显，这些因子主要涉及更多的是个体在处理人际关系时所表现出来积极心理状态。自我管理能力与心理资本两个维度之间均存在显著的关系，这也表明了自我管理能力在工作和生活中的重要性，同时也说明心理资本的改善将有利于自我管理能力的改善，自我管理能力的改变将意味着就业能力的改善，符合有关研究结果[6]，要提升自身的就业竞争能力，在学好专业的同时，还要借助社会和学校的各种实践平台，锻炼提升组织管理能力、心理承受能力、人际交往能力和应变能力等综合素质。

5. 通过改变心理资本促进就业能力的提高。通过研究数据可以看出，心理资本的改变对就业能力的改变有很好的正向预测作用。需要指出的是，事务型心理资本相对于人际型心理资本的培养更具有价值，大学生随着个体的成熟和在大学期间人际交往的广泛，人际型心理资本得到了很好的训练和发展，但是事务型心理资本具有更高的稳定性，要改变需要得到更多的训练和培养，因此，应该更加有意识地加强事务型心理资本的培养。因而，对于大学生和就业指导部门来说，关注就业能力比关注就业机会更具有发展和竞争的意义。

（励 骅，铜陵学院心理健康教育与咨询中心副教授，安徽铜陵 244061；曹杏田，安徽师范大学教育科学学院硕士研究生，安徽芜湖 241000）

参考文献

[1] Luthans F,Youssef C,Avolio B J.心理资本——打造人的竞争优势[M].李超平,译.北京:中国轻工业出版社,2007:1.
[2] 柯江林,孙健敏,李永瑞.心理资本:本土量表的开发及中西比较[J].心理学报.2009(09):875-888.
[3] 赵 冬.大学生就业能力自评量表的初步编制[D].成都:四川师范大学,2006.
[4] 柯江林,孙健敏,石金涛,顾琴轩.人力资本、社会资本与心理资本对工作绩效的影响——总效应、效应差异及调节因素[J].心理学报,2010(04):29-35.
[5] 仲理峰.心理资本对员工的工作绩效、组织承诺及组织公民行为的影响[J].心理学报,2007(02):328-334.
[6] 黄领仪,陈 婷,王树青.大学生就业困难原因分析与指导政策[J].职业,2010(07):28-29.

（上接第 34 页）

（姜尔林，华东理工大学社会与公共管理学院副教授，上海 200237；马桂敏，华东理工大学研究生院研究员，上海 200237；宋恭华，华东理工大学校长助理、教授，上海 200237）

参考文献

[1] [美]道格拉斯·C·诺斯.制度、制度变迁与经济绩效[M].上海:格致出版社,上海三联书店,上海人民出版社,2008.
[2] 张海英,等.关于我国工程硕士培养现状的调查报告[J].高等工程教育研究,2006(03).
[3] 夏天娟,等.工程硕士培养质量存在的问题与对策:基于问卷调查结果的分析[J].高等工程教育研究,2009(01).
[4] 李景元,等.浅析“双导师制”在工程硕士培养过程中的落实情况[J].学位与研究生教育,2008(12).
[5] [美]R·科斯,A·阿尔钦,D·诺斯,等.财产权利与制度变迁:产权学派与新制度学派译文集[M].上海:上海三联书店,上海人民出版社,1994.
[6] [德]柯武则,史漫飞.制度经济学:社会秩序与公共政策[M].北京:商务印书馆,2000.

(C)1994-2022 China Academic Journal Electronic Publishing House. All rights reserved. http://www.cnki.net
