# Supplementary material for: A study on the current status and influencing factors of psychological capital among medical undergraduates
Source: Front Psychol. 2026 Oct 30;17:1830730. doi: 10.3389/fpsyg.2026.1830730 (PMC13759799; doi:10.3389/fpsyg.2026.1830730)
Supplement: Supplementary file 1 [file Data_Sheet_1.ZIP › References PDF/Study on Medical Students' Psychological Capital.pdf]

·论 著·(社会心理)

医学生心理资本现状调查研究*

史艳琴

中国.山西医科大学汾阳学院(山西汾阳) 032200 E-mail:52965488@qq.com

【摘 要】 目的:了解医学生心理资本现状。方法:采用张阔等人修订编写的大学生心理资本调查问卷对 500 名大三医学生进行问卷调查。结果:医学生的心理资本得分与标准差是(4.697±0.572),说明医学生心理资本总体状况较正面积极。心理资本在性别、生源地、是否独生、是否单亲、是否获得奖学金上的得分没有显著差异;在家庭经济情况、父母教养方式、学习成绩、是否学生干部、人际关系状况上得分差异显著($F=3.272,P<0.05;F=4.745,P<0.01;F=9.955,P<0.001;t=2.124,P<0.05;F=37.166,P<0.001$)。结论:家庭经济富裕、父母教养方式为民主型、学习成绩中上等以上、担任学生干部及人际关系很好的医学生积极心理资本显著优于该变量所在分组的其他变量。

【关键词】 医学生;心理资本;自我效能;乐观;韧性;希望

中图分类号:R395.9 文献标识码:A 文章编号:2096-4811(2018)03-0425-05

doi:10.13342/j.cnki.cjhp.2018.03.030

Study on Medical Students' Psychological Capital

SHI Yanqin

Fenyang College of Shanxi Medical University,Fenyang 032200,*China*

【**Abstract**】 **Objective**:To study the status of medical students' psychological capital. **Methods**:A total of 500 medical students were investigated with positive psychological capital scale by Zhang Kuo. **Results**:The general situation of psychological capital of medical students was more positive. There was no significant difference in terms of sex,origin,whether only one-child,whether single-parent,whether got a scholarship. The significant differences existed in family economics,parental rearing pattern,achievements,whether student cadre and interpersonal relationship($F=3.272,4.745,9.955,2.124,37.166;P<0.05$). **Conclusion**:Family economic prosperity,democratic parental rearing patterns,medium and high grade in achievements,student cadres,and good interpersonal relationship play more important roles in psychological capital of medical students than the other variables.

【**Key words**】 Medical students;Psychological capital;Self-efficacy;Optimism;Resilience;Hope

心理资本是个体在成长和发展过程中表现出来的积极心理状态,是促进个人成长的心理资源。这一概念最早在 2004 年由 Luthans 提出,与人力资本、社会资本概念共同出现在经济学、管理学、投资学等学科领域且具有超过人力资本、社会资本的地位。

Luthans 等人经过后续研究,最终将心理资本的定义修订为个体的积极的心理状态[1],具有以下四大特点,一是拥有表现和付出必要努力成功完成具有挑战性任务的自信(自我效能感),二是对自身当前及未来获得的成功做出积极归因(乐观),三是坚守既定目标,在遭遇困难或挫折时,能重新选择路线并不断努力力求既定目标实现(希望),四是遭遇困难或挫折时能坚持、很快恢复和采取迂回途径来获得成功(韧性)。这种积极的心理资源是类似于状态(state-like)的积极心理力量,可以通过有针对性的投入和开发而使个体获得竞争优势。也恰恰是这一点,为本研究铺设了理论基础。

国内外对心理资本的研究主要集中在经济学、管理学、组织行为学及投资学领域。这些研究大多是以企业领导和员工为研究对象,研究内容涉及心理资本的构成要素,用实证研究的方法探讨心理资本与领导和员工的工作态度和工作行为之间的关系[2-7]。国内对心理资本的研究尤其心理学学科领域,主要集中在近 5 年。研究对象多数以大学生为主,内容多数围绕心理资本研究述评、现状调查、心

* 基金项目:山西省高等学校哲学社会科学研究一般项目(编号:20172219)

(C)1994-2023 China Academic Journal Electronic Publishing House. All rights reserved. http://www.cnki.net

理资本的要素构成研究等。也正是这些研究所取得的成就启发了笔者对医学生心理资本的关注与研究。

医学生是高校教育中的特殊专业群体,不仅有一般大学生群体存在的适应、学习、人际交往等问题,同时还有独属于医学生的职业认同及未来职业决策等问题。一般医学院校的课程门数较多,安排较紧张,对知识尤其是医学知识的掌握准确度较高,因此,学生的学习压力较大,业余时间较少,业余生活相对单调,极易出现心理问题。了解医学生的心理资本状况及其心理资本的影响因素,并努力寻求培育心理资本的有效途径对提高医学生的心理健康水平有重大意义。

1　对象与方法

1.1　对象

采用方便抽样的方法,选取山西医科大学汾阳学院五年制本科临床医学专业、医学影像专业和口腔医学专业大三学生为研究对象,发放问卷 550 份,回收有效问卷 500 份(93.4%)。

1.2　方法

实施方法:采用现场发放和问卷星网站收集问卷相结合的方式开展调查。现场回收的问卷采用 Epidata 软件录入数据。采用问卷星回收的问卷数据直接以 Excel 文件形式下载,问卷链接地址为:https://sojump.com/jq/12326101.aspx。

研究工具:采用张阔等人修订编写的大学生心理资本调查问卷。该问卷包括自我效能、韧性、希望和乐观 4 个维度,共 26 道题目。其中有 5 道题目为反向计分。各维度下项目得分均值作为各维度的分值,以各维度的均值作为总问卷分值,分值越高说明各维度或总体的心理资本水平越高。该问卷 Cronbachα 系数为 0.899,各维度 Cronbachα 系数分别为 0.836、0.780、0.835、0.781。

同时,研究还包括性别、生源地、家庭经济状况、是否独生子女、家庭教养方式、是否单亲、学习成绩、是否担任学生干部、是否获得奖学金、人际关系等与研究相关的 10 项人口学变量。

1.3　统计处理

采用 SPSS 13.0 进行描述性统计分析、t 检验、单因素方差分析,以 $P<0.05$ 为差异有统计学意义。

2　结　果

2.1　医学生心理资本整体概况

以各维度的均值作为总问卷的得分。医学生心理资本及其各维度描述性见表 1。由于本问卷采用 Likert 7 点评定量表形式,可见,大学生的心理资本总体上讲是较为正面积极的。尤其在希望和乐观维度上得分较高。

2.2　各人口学变量的医学生心理资本状况的描述性统计

各人口学变量分组后的心理资本状况描述性统计见表 2。由于本问卷采用 Likert 7 点评定量表形式,可见,不同组别医学生的心理资本总体上讲也是较为正面积极的。

表 1　医学生心理资本及各维度的描述性统计

项　目	$\bar{x}\pm s$
自我效能	4.601±0.914
韧　性	4.080±0.624
希　望	5.008±0.714
乐　观	5.096±0.986
心理资本	4.697±0.572

表 2　各人口学变量上医学生心理资本状况的描述性统计($\bar{x}\pm s$)

项　目		n(%)	$\bar{x}\pm s$
性　别	男	188(37.6)	4.759±0.627
	女	312(62.4)	4.659±0.533
生源地	农村	278(55.6)	4.709±0.584
	小城镇	110(22.0)	4.597±0.521
	中小城市	103(20.6)	4.756±0.575
	大城市	9(1.8)	4.820±0.680
家庭经济状况	较贫困	99(19.8)	4.616±0.711
	一　般	395(79.0)	4.709±0.527
	富　裕	6(1.2)	5.182±0.636
是否独生子女	是	115(23.0)	4.765±0.593
	否	385(77.0)	4.676±0.564
家庭教养方式	溺爱型	15(3.0)	4.827±0.556
	民主型	341(68.2)	4.753±0.526
	专制型	47(9.4)	4.572±0.686
	放任型	97(19.4)	4.536±0.634
是否单亲	是	33(6.6)	4.707±0.540
	否	467(93.4)	4.696±0.574
学习成绩	下	18(3.6)	4.274±0.954
	中　下	73(14.6)	4.553±0.568
	中	238(47.6)	4.638±0.535
	中　上	148(29.6)	4.859±0.500
	上	23(4.6)	5.040±0.588
是否获得奖学金	是	236(47.2)	4.737±0.563
	否	264(52.8)	4.660±0.578
是否担任学生干部	是	213(42.6)	4.759±0.557
	否	287(57.4)	4.649±0.759
人际关系	较　差	22(4.4)	4.052±0.724
	一　般	347(69.4)	4.630±0.527
	很　好	131(26.2)	4.980±0.518

在诸多人口学变量中,家庭经济状况富裕、学习

(C)1994-2023 China Academic Journal Electronic Publishing House. All rights reserved.　http://www.cnki.net

成绩上等、人际关系很好的医学生总问卷得分较高,提示家庭经济状况、学习成绩、人际关系较其他变量对心理资本更有积极正面的影响。

2.3　各人口学变量上的医学生心理资本状况

为比较各人口学变量上不同分组的学生样本之间是否存在显著性差异,对数据进行独立样本的 t 检验和单因素方差分析,见表 3。自我效能维度上,男生得分高于女生,独生子女得分高于非独生子女,学习成绩为上等、担任过学生干部、人际关系很好的学生自我效能感最强,且在统计学上具有显著性差异($P<0.001$),生源地、家庭经济状况、家庭教养方式在医学生的自我效能感维度上具有统计学意义。韧性维度上,除性别,其余 9 项人口学变量均不具有统计学意义。希望维度上,学习成绩中上、获得过奖学金、人际关系很好的学生更心怀希望,且在统计学上具有显著性差异($P<0.001$)。乐观维度上,富裕家庭、学习成绩中上、人际关系很好的学生更乐观。总问卷心理资本方面,学习成绩为上等、人际关系很好的学生有较高较积极的心理资本水平。

表 3　各人口学变量上医学生心理资本($\bar{x}\pm s$)

项　目		n	自我效能	韧　性	希　望	乐　观	心理资本
性　别	男	188	4.858±0.942	4.178±0.634	4.958±0.793	5.014±1.092	4.759±0.627
	女	312	4.446±0.862	4.021±0.611	5.021±0.662	5.146±0.914	4.659±0.533
	t		5.003***	2.751**	-0.547	-1.448	1.91
生源地	农　村	278	4.532±0.925	4.108±0.659	5.034±0.702	5.162±0.948	4.709±0.584
	小城镇	110	4.481±0.875	4.000±0.551	4.913±0.723	4.993±1.005	4.597±0.521
	中小城市	103	4.871±0.877	4.115±0.592	5.021±0.710	5.017±1.030	4.756±0.575
	大城市	9	5.079±0.836	3.793±0.655	5.203±0.992	5.203±1.340	4.820±0.680
	F		5.070**	1.539	1.007	1.066	1.671
家庭经济状况	较贫困	99	4.417±1.110	4.111±0.674	4.897±0.841	5.038±1.128	4.616±0.711
	一　般	395	4.636±0.846	4.078±0.608	5.026±0.672	5.095±0.943	4.709±0.527
	富　裕	6	5.285±1.238	3.667±0.764	5.667±0.730	6.111±0.814	5.182±0.636
	F		4.037*	1.441	3.915*	3.378*	3.272*
是否独生	是	115	4.914±0.954	4.096±0.631	5.012±0.714	5.039±1.038	4.765±0.593
	否	385	4.508±0.882	4.075±0.622	5.007±0.714	5.113±0.970	4.676±0.564
	t		4.255***	0.301	0.056	-0.709	1.468
父母教养方式	溺爱型	15	4.657±0.934	4.152±0.557	5.133±0.807	5.367±0.941	4.827±0.556
	民主型	341	4.681±0.840	4.068±0.619	5.061±0.663	5.201±0.929	4.753±0.526
	专制型	47	4.386±1.168	4.218±0.644	4.964±0.785	4.720±1.088	4.572±0.686
	放任型	97	4.413±0.991	4.044±0.638	4.823±0.807	4.866±1.059	4.536±0.634
	F		3.162*	0.989	3.059*	5.893**	4.745**
是否单亲	是	33	4.571±0.842	4.255±0.746	4.994±0.792	5.005±1.077	4.707±0.540
	否	467	4.603±0.920	4.067±0.613	5.009±0.709	5.102±0.980	4.696±0.574
	t		-0.193	1.67	-0.111	-0.55	0.106
学习成绩	下	18	4.127±1.428	4.238±0.699	4.287±1.102	4.444±1.397	4.274±0.954
	中　下	73	4.424±0.911	4.062±0.653	4.776±0.710	4.952±0.943	4.553±0.568
	中	238	4.514±0.827	4.066±0.648	4.923±0.665	5.046±0.986	4.638±0.535
	中　上	148	4.795±0.869	4.083±0.574	5.253±0.606	5.305±0.878	4.859±0.500
	上	23	5.180±1.134	4.142±0.556	5.068±0.528	5.232±1.110	5.040±0.588
	F		6.688***	0.39	17.917***	4.391**	9.955***
是否获得奖学金	是	236	4.622±0.917	4.030±0.635	5.156±0.689	5.141±0.983	4.737±0.563
	否	264	4.582±0.913	4.124±0.611	4.876±0.711	5.056±0.988	4.660±0.578
	t		0.488	-1.676	4.456***	0.963	1.52
是否学生干部	是	213	4.808±0.866	4.069±0.607	5.076±0.707	5.082±1.014	4.759±0.557
	否	287	4.447±0.920	4.088±0.637	4.957±0.715	5.106±0.966	4.649±0.759
	t		4.457***	-0.345	1.848	-0.261	2.124*
人际关系状况	较　差	22	3.616±1.202	4.032±0.576	4.303±0.863	4.257±1.210	4.052±0.724
	一　般	347	4.478±0.817	4.061±0.609	4.960±0.694	5.019±0.951	4.630±0.527
	很　好	131	5.096±0.868	4.137±0.670	5.253±0.633	5.441±0.913	4.980±0.518
	F		40.142***	0.765	20.726***	18.218***	37.166***

注:* $P<0.05$,** $P<0.01$,*** $P<0.001$

(C)1994-2023 China Academic Journal Electronic Publishing House. All rights reserved.　http://www.cnki.net

表 4　单因素方差分析的事后多重比较

项　目				Mean Difference	P
自我效能	生源地	中小城市	农　村	0.3381	0.001
			小城镇	0.3891	0.002
心理资本		中小城市	小城镇	-0.159	0.043
自我效能	家庭经济	较贫困	一　般	-0.219	0.032
			富　裕	-0.868	0.023
希　望		富　裕	较贫困	0.769	0.010
			一　般	0.640	0.029
乐　观		富　裕	较贫困	1.072	0.010
			一　般	1.015	0.012
自我效能	教养方式	民主型	专制型	0.295	0.037
			放任型	0.267	0.011
希　望		民主型	放任型	0.238	0.004
乐　观		民主型	专制型	0.482	0.002
			放任型	0.335	0.003
		溺爱型	专制型	0.288	0.025
心理资本		民主型	专制型	0.181	0.040
			放任型	0.216	0.001
自我效能	学习成绩	中　上	下	-0.668	0.003
			中　下	0.127	0.004
			中	0.093	0.003
		上	下	1.053	0.000
			中　下	0.755	0.000
			中	0.665	0.001
希　望		中　上	下	0.167	0.000
			中　下	0.095	0.000
			中	0.070	0.000
			上	-0.355	0.018
		上	下	1.321	0.000
			中　下	0.832	0.000
			中	0.685	0.000
乐　观		中　上	下	0.860	0.000
			中　下	0.353	0.011
			中	0.258	0.012
		上	下	0.787	0.010
心理资本		中　上	下	0.585	0.000
			中　下	0.305	0.000
			中	0.221	0.000
		上	下	0.766	0.000
			中　下	0.486	0.000
			中	0.403	0.001
自我效能	人际关系	较　差	一　般	-0.861	0.000
			很　好	-1.474	0.000
		一　般	很　好	-0.613	0.000
希　望		较　差	一　般	-0.657	0.000
			很　好	-0.950	0.000
		一　般	很　好	-0.292	0.000
乐　观		较　差	一　般	-0.761	0.000
			很好	-1.183	0.000
		一　般	很　好	-0.422	0.000
心理资本		较　差	一　般	-0.577	0.000
			很　好	-0.928	0.000
		一　般	很　好	-0.350	0.000

方差齐性的前提下，采用 LSD（最小显著差法）进行单因素方差分析的事后多重比较（见表 4）。结果表明，在自我效能维度上，生源地导致差异的原因在于中小城市与农村、小城镇之间的差别；家庭经济状况导致差异的原因在于富裕与较贫困之间的差别；家庭教养方式导致差异的原因在于民主型与专制型、放任型之间的差别；学习成绩导致差异的原因在于上等与下等、中下的差别、上等与中等之间的差别；人际关系导致差异的原因在于人际关系较差与一般、很好之间的差别。

希望维度上，家庭经济状况导致差异的原因在于富裕与较贫困、一般之间的差别；家庭教养方式导致差异的原因在于民主型与放任型之间的差别；学习成绩导致差异的原因在于中上、上等与下等、中下、中等间的差别、中上等与上等之间的差别；人际关系导致差异的原因在于较差与一般、很好之间的差别以及人际关系一般与很好之间的差别。

乐观维度上，家庭经济状况导致差异的原因在于富裕与较贫困、一般之间的差别；家庭教养方式导致差异的原因在于民主型与放任型之间的差别以及溺爱型与专制型之间的差别；学习成绩导致差异的原因在于中上与下等、中下、中等间的差别、上等与下等之间的差别；人际关系导致差异的原因在于较差与一般、很好之间的差别以及人际关系一般与很好之间的差别。

心理资本维度上，生源地导致差异的原因在于中小城市与小城镇之间的差别；家庭教养方式导致差异的原因在于民主型与专制型、放任型之间的差别；学习成绩导致差异的原因在于中上、上等与下等、中下、中等间的差别、上等与中等之间的差别；人际关系导致差异的原因在于较差与一般、很好之间的差别以及人际关系一般与很好之间的差别。

3　讨　论

3.1　医学生心理资本总体情况

本研究医学生心理资本的得分为（4.697±0.572），由于本问卷采用 Likert7 点评定量表形式，可见，医学生的心理资本总体上讲是较为正面积极的。这与理论假设及已有的研究结果基本一致[8-11]。医学生群体虽自身具有较正面积极的心理因素，但不可忽视之处在于仍有一部分医学生的心理资本水平处于中等及较低位，结合心理资本的类状态特点，医学生心理资本的培育及提高仍有很大空间。

在各维度上具体看，医学生韧性因子得分明显低于自我效能、乐观、希望。医学生整体能表现出挑战难度任务的自信、获得成功后的积极归因且具有

(C)1994-2023 China Academic Journal Electronic Publishing House. All rights reserved.　http://www.cnki.net

追求成功受阻后重新调整并力求目标实现的心理动力。韧性因子得分较低，说明医学生在面对困难、生活逆境、创伤或其他生活重大压力时会表现出不同程度的适应不良，面对生活压力和挫折时“反弹能力”不足。作为存在于人类机体中的一种自我保护本能，韧性推动着人们克服生命威胁、追求自我实现[12]。医学生承载着全人类社会健康的维护和促进的艰巨任务，人类社会健康事业任重而道远，动力与压力并存，而面对各方压力，医学生应多渠道多途径提高自身的韧性能力。

3.2　不同人口学变量医学生心理资本的具体表现特点

从第二部分数据分析结果可以看出，不同人口学变量的医学生在心理资本得分及各维度上的一些差异表现具有统计学意义。

研究表明，心理资本得分上，家庭经济情况、父母教养方式、学习成绩、是否学生干部、人际关系状况等人口学变量带来的差异显著。富裕的家庭经济、民主型的父母教养方式给个体提供的资源及自我发展空间较大；担任学生干部的个体在班级管理、社团管理及各项活动中锻炼较多，能力提升较快；医学生的学习成绩在心理资本得分上贡献了较多的差异量，这一特点也是医科院校有别于文科类、理工类院校的。从学校教育角度来看，担任学生干部、提高学习成绩、和谐的人际关系可以明显改善医学生的心理资本状况，因此，学校各级机构含各教学系部、学生处团委等行政管理部门、大学生心理健康中心等机构可以从以上方面入手提升医学生的积极心理资本。

3.3　不同人口学变量医学生心理资本各维度的具体表现特点

对 10 项人口学变量进行归纳，形成性别、家庭环境、学校教育 3 大类。家庭环境包括生源地、家庭经济、是否独生、父母教养方式、是否单亲等 5 个小项。学校教育包括学习成绩、是否担任学生干部、是否获得奖学金及人际关系等 4 个小项。以下仅就结果与理论假设及已有研究结果不一样的变量进行讨论分析。

性别变量上，男生在自我效能、韧性维度得分较女生高，且差异显著。这可能与医学院校、医学专业、医生职业的特点有关。后经个别访谈发现，男生较女生更多地表示未来会从事本专业工作。对于五年制本科医学生来说，男生较女生在学业、职业选择压力面前表现更镇定、更坚定、更具有耐受力、更易调动积极的自我资源。

家庭环境变量在韧性维度的得分上均不显著。这一点与理论假设不一致。已有研究表明家庭环境是影响大学生心理韧性的重要因素[13-15]，但在本研究中，却得到了相反结论。考虑原因主要在于医科院校、所学专业的特点。医学专业知识不论对何种家庭环境的学生来说都是一块难啃的硬骨头，都需要有坚强的意志、不逃避不回避的决心和压力应对策略。在经历了大学 3 年的基础和临床知识的学习后，医学生没有表现出较严重的学习或行为问题，且能很好适应学校生活，更努力地实现自己的职业目标。

（致谢：感谢张阔老师提供大学生积极心理资本问卷供本研究使用。）

参考文献

[1]Luthans F, Avolio B J, Walumbwa F O, et al. The psychological capital of Chinese workers: Exploring the relationship with performance[J]. Management and Orgnization Review, 2005, 1(2): 247-269

[2]Goldsmith A H, Darity W and Veum J R. Race, cognitive skills, psychological capital of Chinese and wages[J]. Review of Black Political Economy. 1998, 26(2): 13-22

[3]Luthans F, Youssef C M, Avolio B J. Psychological capital: Developing the human competitive edge[M]. Oxford, UK: Oxford University Press, 2007: 98-102

[4]Luthans F, Youssef C M. Human social and now positive psychological capital management: Investing in people for competitive advantage[J]. Organizational Dynamics, 2004, 33(2): 143-460

[5]张阔，张赛，董颖红. 积极心理资本：测量及其与心理健康的关系[J]. 心理与行为研究，2010，8(1)：58-64

[6]仲理峰. 心理资本研究述评与展望[J]. 心理科学进展，2007，15(3)：482-487

[7]柯江林，孙健敏，李永瑞. 心理资本：本土量表的开发及中西比较[J]. 心理学报，2009，41(9)：875-888

[8]李林英，肖雯. 大学生心理资本的调查研究[J]. 北京理工大学学报(社会科学版)，2011，13(1)：148-152

[9]秦红霞，张杰覃，冬雪. 应用型本科院校大学生心理资本的调查分析[J]. 黄山学院学报，2013，15(2)：93-96

[10]潘清泉，周宗奎. 贫困大学生心理资本、应对方式与心理健康的关系[J]. 中国健康心理学杂志，2009，17(7)：844-846

[11]张阔，张赛，董颖红. 积极心理资本：测量及其与心理健康的关系[J]. 心理与行为研究，2010，8(1)：58-64

[12]Richardson G E. The metatheory of resilience and resiliency[J]. Journal of Clinical Psychology, 2002, 58(3): 307-321

[13]雷万胜，陈栩，陈锦添. 大学生心理韧性研究[J]. 中国健康心理学杂志，2008，16(2)：155-157

[14]蒋玉涵，李义安. 高中生心理韧性与主观幸福感的关系研究[J]. 中国健康心理学杂志，2011，19(11)：1357-1360

[15]林之锐，胡瑜. 中小学教师健康坚韧性及影响因素的研究[J]. 心理科学，2005，28(4)：969-971

（收稿时间：2017-12-09）

http://www.cjhp.com.cn/

(C)1994-2023 China Academic Journal Electronic Publishing House. All rights reserved.　http://www.cnki.net
