# Supplementary material for: A study on the current status and influencing factors of psychological capital among medical undergraduates
Source: Front Psychol. 2026 Oct 30;17:1830730. doi: 10.3389/fpsyg.2026.1830730 (PMC13759799; doi:10.3389/fpsyg.2026.1830730)
Supplement: Supplementary file 1 [file Data_Sheet_1.ZIP › References PDF/Study on the Current Situation of Career Decision-Making Difficulties Among Medical Interns and Its Relationship with Psychological Capital and Coping Styles.pdf]

●论　著

医学实习生职业决策困难现状及与心理资本和应对方式的相关性研究

马　晶[1]，邓菁菁[2]，杨嘉慧[2]，武晋彤[2]，艾力希尔·艾尼完尔[2]，岳晨媛[2]，汪惠才[1*]

（1. 新疆医科大学第一附属医院；2. 新疆医科大学，新疆 乌鲁木齐 830000）

［摘要］　目的：了解新疆某高校医学实习生职业决策现状，分析其与心理资本和应对方式的相关性。方法：随机选取新疆某高校医学实习生 303 人进行问卷调查，采用一般资料调查表、积极心理资本问卷、职业决策困难问卷、简易应对方式量表对其进行调查。结果：303 名医学实习生职业决策困难总分为（95.68 ± 18.56）分，总均分为 2.73 分，比普通大学生职业决策困难的水平（2.79 分）略低[1]；Pearson 相关性分析，职业决策困难总分与心理资本总分、积极应对方式总分、消极应对方式总分相关系数分别为 －0.44、－0.32、0.23（$P < 0.01$）；多元逐步回归分析显示，心理资本、应对方式、大学期间是否担任学生干部、是否满意自己的专业及专业前景是新疆医学实习生职业决策困难的主要影响因素（$P < 0.05$）。结论：提高心理资本、采用积极应对方式代替消极应对方式、具备良好专业就业前景、提高专业满意度、增加学生担任学生干部的机会对减小职业决策困难具有积极的作用。

［关键词］　医学实习生；心理资本；职业决策；应对方式　DOI：10.3969/j.issn.1002-1701.2024.06.011

［中图分类号］　G465　［文献标识码］　A　［文章编号］　1002-1701（2024）06-0028-04

当前，大学生就业形势严峻，引起了社会的广泛关注。为响应党中央、国务院号召，各地政府和高校高度重视学生就业工作，通过精准的政策扶持和持续的就业服务，助力大学生实现高质量就业[2]。然而，在医学专业领域，毕业生仍面临着一定程度的就业挑战[3]。我国高等医学院校招生规模持续扩大，导致医学毕业生人数增加，同时，医疗行业对医学生个人能力要求不断提高，这也成为大学生心理压力的重要来源，无形中加剧了医学实习生们的职业决策困难[4]。研究表明，医学实习生的职业决策困难关键在于自身的情绪调节与疏通[5-6]，因此，进行医学实习生职业决策困难相关研究，不仅有助于大学生多方位了解自身的价值和意义，也能帮助高校的就业教育服务机构和相关部门更客观地了解大学生的心理特点和就业需求，有针对性地建立系统的职业生涯规划教育体系，提高大学生的社会适应能力，进而发挥推动作用[7]。因此，本研究旨在分析职业决策困难、心理资本、应对方式之间的关系，探究影响职业决策困难的主要因素，为学校管理者更好地帮助实习生降低职业决策难度提供参考。

一、对象与方法

（一）研究对象。

采取随机抽样法，选取新疆某高校附属医院 2019 年 6 月至 2020 年 1 月期间的实习生 303 人为研究对象。其中男生 115 人，女生 188 人。本研究经医院医学伦理委员会审核批准，所有调查对象在知情同意的情况下参与研究。

（二）研究方法。

＊通讯作者　Email：506940196@qq.com

采用问卷星在线电子问卷进行调查，对调查结果统一进行清洗审核，以保证数据质量。（1）一般资料调查表：涵盖基本情况（性别、专业、家庭背景等）、大学期间的学生干部经历、对专业满意程度等方面。（2）积极心理资本问卷[6]：包含 26 个条目，4 个维度。问卷总分越高代表心理资本水平越高。该问卷克朗巴赫 α 系数为 0.90，信度良好。（3）职业决策困难问卷[8]：包含 35 个条目，3 个维度。问卷总分越高表示职业决策困难程度越大。该问卷克朗巴赫 α 系数为 0.89，各分量表与总问卷的相关系数在 0.844 以上，信度良好。（4）简易应对方式量表[9-10]：包含 20 个条目，2 个维度。根据结果偏向哪个维度，可反映出个体采用的应对方式。该问卷克朗巴赫 α 系数为 0.83，信度良好。

（三）统计处理。

数据分析及模型构建使用 SPSS 24.0 统计软件。对于符合正态分布的计量资料采用（$\bar{x} \pm s$）表示；计数资料采用频数和百分比进行描述。在不同组间职业决策困难方面，采用 t 检验和单因素方差分析进行比较。通过 Pearson 相关分析和多元逐步回归分析探究各因素间的相关性。显著性差异将以 $P < 0.05$ 或 $P < 0.01$ 表示。

二、结　果

（一）医学实习生职业决策困难、心理资本和应对方式的总分和各维度得分。

医学实习生职业决策困难总分为（95.68 ± 18.56）分、心理资本总分为（123.36 ± 21.57）分、应对方式的总分为（34.25 ± 7.81）分。各维度得分见表 1。

（二）医学实习生职业决策困难得分的单因素分析。

在性别、是否单亲、家庭子女数方面，医学实习生职业

中国知网　https://www.cnki.net

表 1　医学实习生心理资本、职业决策困难和应对方式各维度得分($n=303$,分,$\bar{x}\pm s$)

项目	维度	条目数	得分
心理资本	自我效能	7	32.06 ± 6.64
	韧性	7	31.22 ± 6.02
	希望	6	30.65 ± 6.64
	乐观	6	29.43 ± 6.38
职业决策困难	缺乏准备	12	32.74 ± 6.78
	信息探索困难	12	33.13 ± 6.83
	冲突矛盾	11	29.81 ± 6.50
应对方式	积极应对	12	23.86 ± 5.72
	消极应对	8	10.38 ± 4.88

决策困难得分的差异均无统计学意义(均 $P>0.05$)。在大学期间是否担任过学生干部、是否接受过职业指导方面的训练或教育、是否满意自己的专业、专业就业前景方面,医学实习生职业决策困难得分的差异均有统计学意义(均 $P<0.05$)。详细情况见表 2。

表 2　实习生职业决策困难得分单因素分析($n=303$,分,$\bar{x}\pm s$)

项目	人数	职业决策困难总分	t/F	P
性别(X_1)			−0.15	0.88
男	115	95.48 ± 17.35		
女	188	95.81 ± 19.31		
是否单亲(X_2)			1.28	0.17
否	261	96.28 ± 18.19		
是	42	92.00 ± 20.57		
家庭子女数(X_3)			0.71	0.54
1 个	66	92.80 ± 15.69		
2 个	96	96.78 ± 19.45		
3 个	94	95.98 ± 18.67		
4 个及以上	47	96.89 ± 20.28		
大学期间是否担任过学生干部(X_4)			3.49	<0.01
否	129	99.92 ± 17.10		
是	174	92.54 ± 19.02		
大学期间是否接受过职业指导方面的训练或教育(X_5)			2.95	<0.01
否	122	99.47 ± 18.10		
是	181	93.13 ± 18.48		
是否满意自己的专业(X_6)			16.39	<0.01
满意	226	92.30 ± 18.15		
不满意	8	107.88 ± 15.15		
说不清	69	105.36 ± 16.28		
专业就业前景(X_7)			13.61	<0.01
不错	135	89.99 ± 18.11		
一般	132	97.58 ± 18.04		
较差	10	113.80 ± 12.04		
不知道	26	108.65 ± 12.04		

(三)医学实习生职业决策困难、心理资本、应对方式的相关性分析。

医学实习生职业决策困难总分与心理资本总分、积极应对方式总分、消极应对方式总分相关系数分别为 −0.44、−0.32、0.23($P<0.01$),见表 3。

表 3　职业决策困难、心理资本、应对方式的相关性分析(r)

项目		积极应对总分	消极应对总分	职业决策困难总分	心理资本总分
积极应对总分	皮尔逊相关性	1	0.079	−0.320**	0.556**
	显著性(双尾)		0.173	0.000	0.000
消极应对总分	皮尔逊相关性	0.079	1	0.233**	−0.086
	显著性(双尾)	0.173		0.000	0.137
职业决策困难总分	皮尔逊相关性	−0.320**	0.233**	1	−0.437**
	显著性(双尾)	0.000	0.000		0.000
心理资本总分	皮尔逊相关性	0.556**	−0.086	−0.437**	1
	显著性(双尾)	0.000	0.137	0.000	

注:** 表示在 0.01 级别(双尾),相关性显著

(四)医学实习生职业决策困难影响因素的多元逐步分析。

设医学实习生职业决策困难总分为因变量 Y,表 2 中的项目 $X_1 \sim X_7$ 为自变量,心理资本总分、积极应对总分、消极应对总分分别为自变量 X_8、X_9、X_{10}。进行逐步线性回归分析后,结果显示:在引入和剔除的标准分别为 0.05 和 0.10 时采用逐步回归法,在全部 10 个自变量中,仅 X_4、X_6、X_7、X_8、X_9、X_{10} 对 Y 有重要的影响,其余自变量对 Y 影响均不显著被剔除,见表 4。结果得出:相关系数 $R=0.587$,决定系数 $R^2=0.344$,说明在逐步拟合的线性回归方程中,因变量 Y 能被自变量解释的部分占 34.4%,其他因素及偶然因素的原因占 65.6%。经过逐步分析得到的最优回归方程为:$Y=-0.190X_8+4.561X_7+0.861X_{10}+3.430X_6-5.095X_4-0.501X_9+117.075$。显著性检验($F$ 检验)得知,$F=25.899$,$P=0.000<0.05$。说明回归方程通过 F 检验,线性回归效果显著,具有统计意义。

表 4　医学实习生职业决策困难影响因素的多元逐步分析

自变量	偏回归系数	标准误差	标准化回归系数	t	P
常量	117.075	7.089		16.515	0.000
X_8	−0.190	0.051	−0.221	−3.698	0.000
X_7	4.561	1.062	0.215	4.297	0.000
X_{10}	0.861	0.182	0.226	4.734	0.000
X_6	3.430	1.105	0.155	3.104	0.002
X_4	−5.095	1.784	−0.136	−2.856	0.005
X_9	−0.501	0.186	−0.154	−2.687	0.008

中国知网　https://www.cnki.net

三、讨　论

(一)医学实习生职业决策困难、心理资本和应对方式的现状。

根据本研究结果显示，医学实习生职业决策困难的总分为(95.68 ± 18.56)分，总均分为 2.73 分，略低于普通大学生职业决策困难水平(2.79 分)，由于医学生的专业性强、就业方向明确且选择面较窄，降低了医学生在职业决策过程中遇到的困难，这一结论与胡霞的研究结果[1]一致。在 3 个维度中，缺乏准备维度的均值得分最高，这表明医学实习生刚开始对医院的工作模式了解较少，准备时间较短；心理资本的总分为(123.36 ± 21.57)分，表现较为优秀，与职业决策困难总分呈负相关，相关系数为 −0.44，这与汤倩等研究结果[11]一致。在 4 个维度中，自我效能的均值得分最高，这说明医学实习生通过 4 年的专业课学习掌握了大量医学基础知识，对即将开始的临床实习充满自信和期待，因此自我效能得分较高，这一结论与周丽等人的研究结果[12]一致；积极应对方式的总分为(23.86 ± 5.72)分，与职业决策困难总分呈负相关，相关系数为 −0.32。而消极应对方式的总分为(10.38 ± 4.88)分，与职业决策困难总分呈正相关，相关系数为 0.23。积极应对方式得分较高，这表明医学实习生心理资本水平较高，遇到问题更倾向于用积极的心态来面对。

(二)医学实习生职业决策困难主要影响因素的分析。

在医学实习生职业决策困难主要影响因素的综合分析中，研究结果显示如下：首先，心理资本与职业决策困难呈负相关。学生若具备奋发进取的精神和积极乐观的态度，即便面临压力和挑战，也能减少心理压力，做出更理性的决策，从而减轻职业决策困难，这与杨丽恒等研究观点[13]一致。其次，积极应对方式与职业决策困难呈负相关，而消极应对方式则呈正相关。研究表明，实习生若能积极沟通、求助他人，而非采取消极态度，能获得情感支持、拓宽信息渠道，从而降低职业决策困难，与石珺等研究结果[14]一致。此外，通过逐步线性回归分析发现，专业就业前景良好、对所学专业满意、担任学生干部等因素也能减少职业决策困难。研究表明，专业前景与社会需求相关，通常专业就业率较高的学生职业决策困难度越低[15]，就业机会越多，竞争压力越小，医学实习生的就业压力也会降低，从而能更从容地做出职业决策。研究显示，对所学专业满意度较高的学生，在校期间培养了良好的学习能力和专业基础，因此在职业选择时通常目标明确，竞争力较强[16-17]。这有助于实习生争取更多机会，减少犹豫不决等职业决策困难因素。研究表明，通过担任学生干部工作，实习生更好地了解自身能力和优势。个体越了解自己，越清晰地了解自己的能力和目标，职业决策困难程度也就越低[18]。通过担任学生干部工作，也可以有效培养学生个人能力，提高竞争力，争取更多机会。

综上所述，提高医学实习生的心理资本水平、采取积极应对方式、提高专业认同、增加学生担任学生干部机会等对降低职业决策困难具有积极作用。建议学校教育管理者从这些影响因素入手，探索综合教育方案。一方面，结合医学专业特色全面认识和分析当前的就业形势，提出切实可行的就业指导对策；另一方面，及时关注医学实习生的心理健康状况，启发学生善于应用积极心理学方法及时干预，调节自身情绪，帮助他们减轻就业压力，降低职业决策困难，促进学生充分发挥才能，助力实习生成长与发展。

[参考文献]

[1] 胡　霞，谢　姒. 医学生职业决策困难的现状及影响因素分析[J]. 蚌埠医学院学报，2015(8)：1080-1083.

[2] 徐雪平. 心理资本理论视角下医学职业院校大学生就业能力培养研究[J]. 决策探索(下)，2021(3)：88-89.

[3] 牛晓璇，许雅鑫，韩　亮. 临床医学专业本科生就业意向与就业现状的调查分析[J]. 中国医疗管理科学，2018，8(4)：66 -69.

[4] 张　倩，蒋冰凝. 高校医学毕业生就业心理影响因素调查[J]. 合作经济与科技，2023(18)：82-83.

[5] 杨嘉慧，邓菁菁，武晋彤，等. 医学实习生主观幸福感对职业决策困难的影响[J]. 职业与健康，2022，38(7)：942-946.

[6] 赵　旋. 大学生心理资本对职业决策的影响[D]. 哈尔滨：哈尔滨师范大学，2019.

[7] 贾殿臻. 大学生情绪智力、职业决策自我效能感与社会适应的关系研究[D]. 郑州：河南大学，2016.

[8] 李　娜. 大学生职业决策困难问卷修编及其特点研究[D]. 重庆：西南大学，2009.

[9] 肖计划. 应付与应付方式[J]. 中国心理卫生杂志，1992，6(4)：181-183.

[10] 于鹏鹏，袁　璐，王东芳，等. 简易应对量表应用于医学生的信效度检验[J]. 山东大学学报(医学版)，2019，57(1)：101 -106.

[11] 汤　倩. 高职院校大学生心理资本、社会技能对职业决策困难的影响研究[J]. 安徽商贸职业技术学院学报(社会科学版)，2019，18(2)：70-73.

[12] 周　丽，赖　文，廖雪萍，等. 医学生心理资本与家庭满意度的关系研究[J]. 重庆医学，2017，46(4)：528-530.

[13] 杨丽恒，母全尚，顾　珍. 大学生心理资本与职业决策困难的关系[J]. 中国健康心理学杂志，2014，22(4)：595-598.

[14] 石　珺. 高中生心理资本现状及干预研究[D]. 兰州：西北师范大学，2015.

[15] 余　婷. 大学生专业认同度与职业决策困难关系研究[D]. 南昌：南昌大学，2017.

[16] 纪君柔，王　艳，施　煜. 大学生专业认同感对学习行为和职业选择行为的影响研究[J]. 教育教学论坛，2015(45)：250-251.

[17] 陈秀梅，马　振，常秀芹，等. 大学生专业认同与职业决策困难关系：心理资本中介作用[J]. 唐山师范学院学报，2022，44(5)：101-104.

(下转第 33 页)

中国知网　https://www.cnki.net

磊等人在各自学科领域进行了课程思政教学的尝试,获得较好教学效果,却均未形成学科统一的教学体系[4-6]。

关键词时间线分析和冲积流结合可见,在国家政策层面的持续催化下,自 2022 年起无论是教学方法还是教学体系建设的研究都展现出星火之势,可以预见,我国临床医学课程思政建设将持续纵深发展。

四、结 论

救死扶伤、仁心仁术的医者精神教育在临床医生的教育体系中至关重要,课程思政是医德医风教育的重要载体。我国临床医学课程思政教育在《纲要》等一系列政策的推动下迅速发展,尽管目前研究主题仍较分散,教学方法和体系建设仍不完善,然而其普及的深度和广度,以及不同学科教学的探索将在教学实践中不断地调整和完善,最终形成润物无声的临床医学课程思政教学体系。

[参考文献]

[1] 韦雨忻,赵士博,李 俊,等.基于 MOOC 翻转课堂的全科医学生临床见习教学"课程思政"的研究与实践[J].科技创新与生产力,2021(7):136-138.

[2] 许明月.高校课程思政三大体系构建[J].高教学刊,2023,9(5):181-184.

[3] 李 沛,李 颖,党 瑞,等.国内医学院校课程思政的研究现状及发展趋势[J].智库时代,2023(18):124-126.

[4] 李春玲,黄长盛,郭曲练,等.临床麻醉学课程思政的设计与实施效果[J].中华医学教育杂志,2023,43(9):659-662.

[5] 王星宇,蒋海泥.课程思政融入心脏外科实习教学的实践探索[J].中国继续医学教育,2023,15(2):30-34.

[6] 黄 磊,王佳贺.课程思政元素融入临床医学本科生全科医学概论教学课程的设计[J].全科医学临床与教育,2023,21(1):1-4.

[作者简介] 程宝莉,女,博士,副教授,硕士生导师,副主任医师,主要研究方向:急危重症患者围术期器官功能保护的临床和基础,麻醉学模拟仿真教学和课程思政。

[基金项目] 浙江大学医学院附属第一医院教学改革项目:麻醉学课程思政建设的探索(zyjg202216);浙江大学学科(专业)思政特色创新项目(第三轮):人民至上,生命至上——高素质药学人才医院联动创新型培养计划。

Research Hotspots and Trends Analysis of Medical Ethics Education in Clinical Medical Courses in Chinese Medical Colleges
——A Bibliometric Study Based on CiteSpace

Cheng Baoli, Zhou Rui, Gao Guohao, Fu Yumeng, Lin Xiaohan, Wu Chaoxin, Ling Qi*

(*The First Affiliated Hospital, Zhejiang University School of Medicine, Hangzhou* 310000, *Zhejiang, China*)

Abstract: Medical ethics education in clinical medical courses is crucial. This article employs bibliometric methods using CiteSpace software to analyze research hotspots, thematic evolution in Chinese medical colleges regarding medical ethics education in clinical medical courses up to September 2023. It also discusses existing issues and development trends, aiming to provide a basis and insights for the construction and implementation of medical ethics education in clinical medical courses.

Keywords: Clinical medicine; Medical ethics education; Research hotspots; Research trends

(上接第 30 页)

[18] 程化琴,庄明科,刘 琉,等.大学新生专业认同感、职业决策困难及生活满意度状况调查——以某大学某专业新生为例[J].教育学术月刊,2014(2):85-89.

[作者简介] 马 晶,女,硕士,讲师,主要研究方向:教育教学管理,健康管理。

[基金项目] 新疆医科大学大学生创新训练计划项目(201910760008);新疆医科大学第十一期教育研究与教学改革项目(YGS2021001);新疆医科大学第二期课程思政教育教学改革项目(YGS2023041)。

Study on the Current Situation of Career Decision-Making Difficulties Among Medical Interns and Its Relationship with Psychological Capital and Coping Styles

Ma Jing[1], Deng Jingjing[2], Yang Jiahui[2], Wu Jintong[2], Ailishier Ainiwener[2], Yue Chenyuan[2], Wang Huicai[1]*

(1. *The First Affiliated Hospital of Xinjiang Medical University*; 2. *Xinjiang Medical University, Urumqi* 830000, *Xinjiang, China*)

Abstract: The objective was to understand the current status of career decision-making difficulties among medical interns at a university in Xinjiang and analyze its correlation with psychological capital and coping styles. A total of 303 medical interns were randomly selected for a questionnaire survey using general information questionnaire, positive psychological capital questionnaire, career decision-making difficulties questionnaire, and a simple coping style scale. Results indicated that the total score of career decision-making difficulties among 303 medical interns was (95.68 ± 18.56) with an average score of 2.73, slightly lower than the general university students' level (2.79). Pearson correlation analysis showed that total career decision-making difficulty score correlated negatively with psychological capital total score and positive coping style total score, but positively with negative coping style total score ($P < 0.01$). Multiple stepwise regression analysis revealed that psychological capital, coping styles, whether they served as student leaders during university, satisfaction with their major, and major prospects were the main influencing factors ($P < 0.05$). Conclusion: Enhancing psychological capital, adopting positive coping styles instead of negative ones, having good career prospects, improving professional satisfaction, and increasing opportunities for students to serve as student leaders can effectively reduce career decision-making difficulties.

Keywords: Medical interns; Psychological capital; Career decision-making; Coping styles

中国知网 https://www.cnki.net
