# Supplementary material for: A study on the current status and influencing factors of psychological capital among medical undergraduates
Source: Front Psychol. 2026 Oct 30;17:1830730. doi: 10.3389/fpsyg.2026.1830730 (PMC13759799; doi:10.3389/fpsyg.2026.1830730)
Supplement: Supplementary file 1 [file Data_Sheet_1.ZIP › References PDF/The association of academic psychological capital and attributional style with psychological help-seeking attitudes of medical students in Ganzhou.pdf]

DOI：10.16506/j.1009-6639.2019.12.003 · 论　著 ·

医学生心理资本与归因风格和心理求助态度的相关性调查研究

黄志靖，陈薇薇，周鑫，许正鹏，许文清，郑亚楠
赣南医学院心理系，江西　赣州　341000

摘要：目的　探讨医学生心理资本、归因风格和心理求助态度的关系，为医学院校开展医学生的心理健康教育及宣传工作提供参考。**方法**　采用心理健康服务求助态度量表、心理资本量表、归因风格量表对赣南医学院 600 名医学生进行匿名施测。**结果**　不同性别医学生在心理健康服务求助态度量表总分中差异有统计学意义（t=2.963，P=0.003）；是否独生子女在心理健康服务求助态度量表总分上差异有统计学意义（t=2.015，P=0.044）；大一与大二、大三学生在心理健康服务求助态度量表总分上差异有统计学意义（t=3.735，P<0.01）；心理资本与心理求助态度呈正相关（r=0.161，P<0.01）；归因风格中能力、努力、运气、任务难度因子都与心理求助态度呈正相关（r=0.241、0.197、0.338、0.339，P<0.01）。线性回归分析显示，心理资本、能力归因、努力归因、运气归因、任务难度归因、性别、年级以及是否独生子女对医学生心理求助态度有一定的预测作用（P<0.05）。**结论**　医学生心理资本和归因风格可以预测心理求助态度。医学生心理求助态度在性别、是否独生子女、年级方面存在差异。

关键词： 医学生；心理求助态度；心理资本；归因风格
中图分类号： R848.3，R395.6　　**文献标识码：** A　　**文章编号：** 1009-6639（2019）12-1140-05

The association of academic psychological capital and attributional style with psychological help-seeking attitudes of medical students in Ganzhou

HUANG Zhi-jing, CHEN Wei-wei, ZHOU Xin, XU Zheng-peng, XU Wen-qing, ZHENG Ya-nan
Department of Psychology, Gannan Medical College, Ganzhou, Jiangxi 341000, China
Corresponding author: ZHENG Ya-nan, E-mail: marine873926@sina.com

Abstract: Objective　To elucidate the association of psychological capital and attributional style with psychological help-seeking attitudes of medical students in Ganzhou, and to provide reference for psychological education.　**Methods**　An anonymous questionnaire survey was conducted among 600 students in Gannan Medical College using inventory of attitudes toward seeking mental health services scale, psychological capital scale and attributional style scale.　**Results**　The differences in the scores of attitudes toward seeking mental health services scale between male and female students (t=2.963, P=0.003), and between students from one-child and multiple children families (t=2.015, P=0.044), as well as among freshmen, sophomore and juniors (t=3.735, P<0.01) were statistically significant. There was a significantly positive correlation between psychological capital and psychological help-seeking attitudes (r= 0.161, P<0.01). The factors in attributional style such as ability (r= 0.241, P<0.01), effort (r=0.197, P<0.01), luck (r=0.338, P<0.01) and task difficulty (r=0.339, P<0.01) were all positively correlated with psychological help-seeking attitudes. Linear-regression analysis showed that psychological capital, ability attribution, effort attribution, luck attribution, task difficulty attribution, gender, grade and number of children in the family had certain predictive values for psychological help-seeking attitudes of medical students (P<0.05).　**Conclusions**　Psychological capital and attributional style of medical students can predict their psychological help-seeking attitudes, which vary significantly between male and female students, and between students from one-child and multiple children families as well as among students in different college grade.

Key words: Medical students; Psychological help-seeking attitudes; Psychological capital; Attributional style

基金项目： 全国教育科学“十三五”规划 2017 年度教育部青年课题项目（EEA170454）
作者简介： 黄志靖，本科，主要从事精神卫生研究
通信作者： 郑亚楠，E-mail：marine873926@sina.com

(C)1994-2022 China Academic Journal Electronic Publishing House. All rights reserved.　http://www.cnki.net

大学时期是人生发展的重要阶段，有研究表明大学生心理问题的发生率高于一般人群[1]，但是大学生寻求专业心理咨询的比率偏低[2]，医学生做为医疗卫生人才，他们心理求助态度可影响未来医生心理健康状况，同时医生心理求助态度会影响患者对心理求助态度，这对改善社会大众人群对心理咨询的态度十分重要。如何提高医学生对心理咨询机构的咨询率、提升其求助态度是未来医学院校心理健康教育的重要内容。

归因风格是个体根据自身内在的认知和价值取向对发生的事件做出习惯性的分析和判定，个体的归因风格具有较鲜明的个性特征，包括能力归因、努力归因、运气归因、任务难度归因，归因的偏差会影响人们看待事情的态度[3]。

心理资本是积极心理学研究的重要领域，是个人成长和发展过程中所体现出来的积极心理状态，包含希望、韧性、乐观和自我效能四个部分，对个体的发展和环境适应有重要促进作用[4]。

临床实践表明大部分寻求心理求助的同学喜欢将不好事情归因于自己，造成自己抑郁、失落，而在平时遇事难过、抑郁的人中，求助于心理咨询中心个体往往能接受心理咨询，他们对生活有着希望，迫切希望去改变当前状态，这提示归因风格、心理资本与心理求助态度可能存在一定的关系。为探讨心理资本、归因风格与心理求助态度是否存在相关关系，本研究于 2019 年 3 月在赣南医学院进行调查，为医学院校开展医学生的心理健康教育及宣传工作提供参考。

1　对象与方法

1.1　对象　2019 年 3 月在赣南医学院以在校大一和大二、大三（临床医学专业、口腔医学专业）为调查对象，以班级为单位，采用整群抽样，将问卷调查总体以专业归并成若干个互不交叉、互不重复的单位，然后从各单位中随机抽取若干个班级，对这些抽取的班级所有个体进行抽样调查。共发放问卷 760 份，回收有效问卷 600 份，回收有效率为 78.95%。其中男生 298 人（49.67%），女生 302 人（50.33%）；大一 300 人（50.00%），大二、大三 300 人（50.00%）；家庭所在地为农村 366 人（61.00%），城镇 234 人（39.00%）；独生子女 154 人（25.67%），非独生子女 446 人（74.33%）；年龄 18～22（19.12±0.65）岁。

1.2　方法

1.2.1　心理健康服务求助态度量表　本研究是以 Mackenzie 和 Corey 修订的心理健康服务求助态度量表（inventory of attitudes toward seeking mental health services，IASMHS）做为研究工具。量表有 3 个分量表，共有 24 个项目，包含心理开放程度因子、心理求助因子以及不在意社会污名因子，总分如果越高，表示被试心理求助态度越积极，反之，则越消极。此量表分为 5 分评分，从“1”到“5”表示“完全不同意”到“完全同意”[5]。在本研究中，量表总分的一致性系数为 0.701。

1.2.2　心理资本量表的修订版　量表由 luthans 编制，用于测量被试心理资本水平的高低，经过胡烨妃修订，共 20 个项目，包含自我效能、希望、韧性和乐观四个维度，问卷采用 1～6 计分法，被试量表总评分越高，表明心理资本水平越高。相关研究表明，修订的量表有较好的信效度[1]，在本研究中，此量表总分的一致性系数为 0.893。为直观显示医学生心理资本整体水平，本研究数据分析使用心理资本总分进行计算。

1.2.3　归因风格量表　本量表根据 Weiner 的归因的理论，按照能力、努力、运气和任务难度四个归因内容编制，问卷共 8 道题目构成，每个归因内容 2 道题目，测查大学生的归因风格，此量表分为 7 级评分，从“1”到“7”表示“根本不符合”到“非常符合”，相关研究表明，本量表有较好的信效度[6]。

1.3　实施程序　测试前，通过接受了统一训练的主试向调查对象介绍测验目的、方式，着重强调调查结果保密，仅用于学术研究，以消除调查对象对于校心理咨询中心态度等敏感调查内容的担心。测试过程采用标准而且统一的指导语，在征得同意后，指导被试应该如何进行答题，要求调查对象自主完成、匿名作答。测试时间控制在 20 min 之内并当场回收好问卷。

1.4　统计学处理　运用 SPSS 23.0 统计软件分析，对正态分布计数资料采用独立样本 t 检验对不同统计学数据组间比较；采用双相关对医学生心理资本、归因风格和心理求助态度相关性进行研究；采用多元线性回归对医学生心理资本、归因风格和心理求助态度进行研究，$P<0.05$ 为差异有统计学意义。

2　结　果

2.1　医学生心理资本、归因风格和心理求助态度的基本特点　心理健康服务求助态度量表的总分为（70.57±9.15）分。不同性别医学生在心理健康服

(C)1994-2022 China Academic Journal Electronic Publishing House. All rights reserved.　http://www.cnki.net

务求助态度量表差异有统计学意义。不同年级医学生在心理健康服务求助态度量表差异有统计学意义。农村子女和城镇子女在心理健康服务求助态度量表差异无统计学意义。独生子女与非独生子女在心理健康服务求助态度量表差异有统计学意义，见表 1。

表 1 不同人口统计学特征医学生心理健康服务求助态度量表总分比较

因素	例数	得分 ($\bar{x}\pm s$，分)	t 值	P 值
性别			2.963	0.003
男性	298	71.68±9.91		
女性	302	69.47±8.21		
年级			3.735	<0.01
新生	300	71.95±9.61		
老生	300	69.19±8.47		
家庭所在地			−0.166	0.868
农村	366	70.52±9.14		
城镇	234	70.64±9.19		
是否独生子女			2.015	0.044
是	154	71.85±9.97		
否	446	70.13±8.82		

2.2 医学生心理资本、归因风格和心理求助的相关性 对医学生心理资本、以及四种归因风格与心理求助态度相关性进行分析，结果显示，医学生心理资本与心理求助态度呈正相关（$r=0.161$，$P<0.01$）；医学生能力归因与心理求助态度呈正相关（$r=0.241$，$P<0.01$）；医学生运气归因与心理求助态度呈正相关（$r=0.388$，$P<0.01$）；医学生努力归因与心理求助态度呈正相关（$r=0.197$，$P<0.01$）；医学生任务难度归因与心理求助态度呈正相关（$r=0.339$，$P<0.01$）。

2.3 医学生心理资本、归因风格和心理求助态度的回归分析 为进一步了解医学生心理资本、四种归因风格对心理求助态度的影响，以医学生心理健康服务求助态度量表总分为因变量，以心理资本、能力归因、运气归因风格、任务难度归因风格、努力归因风格、性别（男性、女性）、年级、是否独生子女（是、否）以及家庭所在地（城镇、农村）为自变量，进行线性回归分析。结果显示，心理资本、能力归因、努力归因、运气归因、任务难度归因对医学生心理求助态度有明显的正性预测作用。性别、年级以及是否独生子女对医学生心理求助态度有一定的预测作用，见表 2。

表 2 医学生心理求助态度评分影响因素线性回归分析（$n=600$）

常数与自变量	回归系数	标准化回归系数	t 值	P 值
常数项	35.160	—	10.605	<0.01
心理资本	0.197	0.271	7.277	<0.01
运气归因	0.867	0.225	4.864	<0.01
任务难度归因	0.763	0.187	4.277	<0.01
能力归因	0.402	0.105	2.498	0.013
努力归因	0.546	0.130	3.394	0.001
性别	−1.728	−0.094	−2.617	0.009
年级	0.891	0.085	2.372	0.018

3 讨 论

本调查结果表明，在独生子女方面，独生子女的心理求助态度更高，这和之前的研究有不同[7]，其原因可能在于独生子女父母对于子女的保护和给予他们的情感温暖大于非独生子女，独生子女父母也容易对独生子女过于呵护和溺爱，导致独生子女依赖性大于非独生子女，他们在离开家庭时会遇到更多的适应问题，心理求助愿望可能会更加迫切。在性别方面，男生心理求助态度高于女生，差异有统计学意义，与之前学者的研究存在差异[8-12]。从传统意义来看女性比男性更易受到心理问题的困扰，但是现在发现男性更愿意接受心理求助。原因在于女性的自尊心较强，不愿意向别人说她的短处，同时有研究结果显示，女性心理污名感受程度低于男性，对于摆脱心理问题的动机没有那么强烈[7]。随着男性社会压力的增大，同时男性的理性思维更为清晰，所以往往男性更愿意去解决心理问题，表现出更为积极的心理求助态度。在年级方面，年级的高低对心理求助态度差异有统计学意义，大一高于大二、大三，这和之前的研究不相同[13-14]，产生这种现象可能是因为大一的医学生作为新生面对着环境的改变，更加倾向于接受外界的帮助，寻求心理求助的愿望更加迫切。

本研究显示，医学生心理资本、归因风格和心理求助态度均呈正相关，说明心理资本得分越高的医学生，即个体有较好胜任任务的信心，具备良好、积极的动机状态，他们有着更多的社会支持资源，能够有效发现和利用这些资源，从而他们的心理求助态度越积极，这与之前的研究相同[1]。相关研究表明心理资本和社会支持呈正相关[15-18]，而社会支持对心理求助行为有预测作用[19]，同时，积极心理资本高的个体往往对自身具有积极的社会评

(C)1994-2022 China Academic Journal Electronic Publishing House. All rights reserved. http://www.cnki.net

价，能够更客观的看待心理咨询、自身心理健康等，故能够提升其心理求助态度。归因风格与心理求助态度呈正相关，与以往研究结论相同[14]。这说明不论医学生是选择了何种归因风格，只要可以减轻个体焦虑、心理负担，减少他们在平时生活中失败信息，改善心理状况，他们都会很积极寻求这种帮助。

回归分析结果表明，除性别、生源地、是否独生子女外，积极心理资本、归因风格对心理求助态度有正性预测作用。美国心理学家海德在 1958 年最早提出了归因问题并且将归因控制点分为内归因（能力和努力）和外归因（运气和任务难度）。将成功归因于能力、努力的个体更倾向于对发生于周围环境中的社会行为归因于自我，为了提高自我，以及减轻个体遇到问题的焦虑，个体的心理求助态度是积极的；同时 Fischer 和 Turner（1970）用行为效能的内在信念来解释这样的结果，内在归因的人对于自己的行为效能是充分的自信的，不会因为向他人寻求帮助而降低对自己行为效能的信任。归因于运气、任务难度的个体，往往对发生于周围环境中的社会行为归因于情景，更倾向于外归因，外归因个体在面对挫折困难时更加乐观，会避免无尽的自责和悔恨，保持良好的心理状态，这对积极的心理求助态度有一定作用。四个不同归因风格都对心理求助态度有一定的预测作用，而个体的不同归因风格和个体的性格有一定的关系，说明性格对心理求助态度影响不大，如果要提高社会人群、医学生的心理求助态度，我们更应该将重心放在提高他们的心理资本上。积极心理资本是大学生在学习和生活中一种积极向上的心理状态，有研究表明，心理资本与心理异常症状呈负相关[20]，积极心理资本高的个体遇到困难挫折时会尽快想办法从挫折中恢复，对生活持有乐观自信的态度[21]；积极心理资本高的个体也更不在意社会污名对他们的影响，他们寻求社会支持、心理求助态度更加积极。社会支持与个体从消极经历中恢复、并且灵活地适应外界多变环境的能力有显著的正相关性[22]，医学生寻求社会支持、心理求助态度越积极，个体的心理健康症状就越少。低心理资本水平的个体在希望、韧性、乐观、自我效能方面相对于高心理资本水平的个体更低，同时他们的心理求助态度又不积极，持久以往他们的心理健康水平会更低。有研究表明，学习成绩偏上、非单亲家庭的大学生普遍比学习成绩偏低、单亲家庭的大学生心理资本水平更高[23]；非贫困生普遍比贫困生心理资本水平更高[24]；遇到问题采用积极应对方式的大学生普遍比消极应对方式的大学生心理资本水平更高[25]，这提示我们在进行提升心理资本改善心理求助态度的策略实施中，应该对成绩偏低、单亲家庭、贫困生、遇事采取消极应对方式的医学生给予更多的关注，增强他们自信、希望、乐观和韧性等心理因素，同时对不同的学生开发他们的心理资本需要有立体化互动，提升学生心理资本的实践还应该加入针对性的指导。

本研究的结果非常有实践意义，既然归因难以改变，我们更应该提高医学生心理资本，培养医学生自信、乐观的性格，同时积极引导医学生找到自己和别人的差异性，让他们做自己擅长的事情；还应鼓励大学生找到自己的榜样，提高自己的心理韧性；医学院校还应掌握帮助医学生提高心理素质的方法以及心理辅助技术引导医学生以积极的情绪投入学习、生活，开发个体心理资本来提高心理求助态度，利于增加心理咨询机构的利用率。学校还应鼓励医学生努力学习，培养他们为实现自己目标努力奋斗的品格，增加他们看待问题的积极性，这有利于自我培养一种积极的心理求助态度。教师应该引导医学生在面对失败、挫折时将更多的原因归于运气和任务难度，使他们乐观的看待自己，提高对自我的信心，同时学校应对不同年级、性别以及是否为独生子女因材施教，对一些更需要心理求助的年级、独生子女有所侧重。相对于向专业性服务求助，大学生更愿意选择的方式为网络、电话咨询[26]，因此我们对大一学生应该多渠道开展心理求助，同时推行心理健康平台，心理咨询中心也应该加强对自身的宣传，提高在校存在感，降低大众对于心理健康服务机构的神秘感，避免学生出现心理困扰但是求助无门的状况。

参考文献

[1] 胡烨妃．大学生心理健康、心理资本与专业性心理求助态度的关系研究[D]. 杭州：浙江理工大学，2011.

[2] 江光荣，王铭．大学生心理求助行为的研究[J]. 中国临床心理学杂志，2003，11（3）：180-184.

[3] 王一名．归因风格与当代大学生心理健康水平之间的关系研究[J]. 中国市场，2015，22（35）：158-159.

[4] 彭咏梅，杨小兵，刘红云，等．医学高职生积极心理资本与心理健康的关系调查[J]. 卫生职业教育，2016，34（19）：123-125.

[5] 庞莉．大学生生命态度与心理求助的现状调查及其教育对策研究[D]. 桂林：广西师范大学，2015.

[6] 游洁．大学生归因风格、价值观和寻求社会支持与帮助的关系研究[D]. 武汉：华中师范大学，2003.

(C)1994-2022 China Academic Journal Electronic Publishing House. All rights reserved. http://www.cnki.net

[7] 郑亚楠，胡雯，赖月月．大学生专业性心理求助态度心理污名和社会支持的关系[J]. 中国学校卫生，2016，37（1）：78-81.

[8] 徐伏莲，钟鸣洁，陈海泉，等．江门市中学生抑郁症状与心理求助现况[J]. 中国学校卫生，2014，35（10）：1500-1502.

[9] 孙思伟，常春，孙玮，等．青少年人群感知抑郁症病耻感与求助意向研究[J]. 中国学校卫生，2013，34（5）：528-530，533.

[10] 李建明．认知及心理健康因素对大学生心理求助的影响研究[D]. 广州：第一军医大学，2006.

[11] Fischer AJ，DeBeer JP，Bokhorst F. Characteristics of students receiving counseling services at the university of Cape Town [J]. South African Br J Guid Couns，2002，30（3）：299-310.

[12] Good GE，Dell DM，Mintz LM，*et al*. Male role and g ender role confl ict：Relations to help seeking in men [J]. J Counsel Psychol，1989（36）：295-300.

[13] 姚莹颖，陈精锋．我国大学生心理求助的研究现状及教育对策[J]. 校园心理，2017，15（2）：109-111.

[14] 杨颖．大学生寻求专业心理帮助态度及其与应对效能和归因方式的关系研究[D]. 福州：福建师范大学，2006.

[15] 姜英达．大学生社会支持、心理资本与学习倦怠的关系研究[D]. 哈尔滨：哈尔滨工程大学，2014.

[16] 何安明，姚予丹．大学生社会支持、心理资本与可就业能力的关系[J]. 信阳师范学院学报（哲学社会科学版），2018，38（4）：27-30+36.

[17] 张晖，李思思，林伟波，等．贫困大学生心理资本、社会支持及心理健康关系研究[J]. 科技创业月刊，2016，29（13）：68-70.

[18] 王良纯．大学生积极心理资本、社会支持与校园生活满意度的关系研究[D]. 昆明：云南师范大学，2014.

[19] 李翠云．大学生心理求助、社会支持与心理健康的关系研究[D]. 呼和浩特：内蒙古师范大学，2007.

[20] 武春霞，秦葆丽．大学生社会支持、心理资本与心理健康的关系[J]. 校园心理，2015，13（6）：376-377.

[21] 安蓉，仉朝晖．生活满意度对大学生心理资本与学业倦怠关系的调节作用[J]. 中国学校卫生，2016，37（2）：229-232.

[22] 李妮娜，李杰，周晓娜，等．医学生心理韧性及其影响因素研究[J]. 中国预防医学杂志，2011，12（10）：878-880.

[23] 陈旭．大学生心理资本提升策略研究[D]. 大连：大连理工大学，2013.

[24] 彭波．贫困大学生心理资本、应对方式与心理健康的关系探讨[J]. 中国市场，2016，23（21）：170-171.

[25] 谢灿杰，肖胜勇，杨玉杰．大学生心理资本、应对方式与幸福感的关系研究[J]. 太原城市职业技术学院学报，2019，21（2）：88-90.

[26] 李艳红．大学生心理求助倾向与求助态度研究[J]. 青少年研究（山东省团校学报），2013，21（6）：39-43.

收稿日期：2019-04-25　**修回日期**：2019-08-10

论文撰写规范

数字使用请按 GB/T 15835-1995《出版物上数字用法的规定》用法。

物理量

物理量量值必须用阿拉伯数字，并正确使用法定计量单位。小学和初中教科书、非专业性科技书刊的计量单位可使用中文符号。

示例：8 736. 80 km（8 736. 80 千米）　600 g（600 克）　100 kg～150 kg（100 千克～150 千克）　12. 5 m^2（12. 5 平方米）　外形尺寸是 400 mm×200 mm×300 mm（400 毫米×200 毫米×300 毫米）　34 ℃～39 ℃（34 摄氏度～39 摄氏度）　0. 59 A（0. 59 安［培］）

非物理量

一般情况下应使用阿拉伯数字。

示例：21. 35 元　45. 6 万元　270 美元　290 亿英镑　48 岁　11 个月　1 480 人　4. 6 万册　600 幅　550 名

整数一至十，如果不是出现在具有统计意义的一组数字中，可以用汉字，但要照顾到上下文，求得局部体例上的一致。

示例 1：一个人　三本书　四种产品　六条意见　读了十遍　五个百分点

示例 2：截至 1984 年 9 月，我国高等学校有新闻系 6 个，新闻专业 7 个，新闻班 1 个，新闻教育专职教员 274 人，在校学生 1 561 人。

(C)1994-2022 China Academic Journal Electronic Publishing House. All rights reserved.　http://www.cnki.net
