# Supplementary material for: A study on the current status and influencing factors of psychological capital among medical undergraduates
Source: Front Psychol. 2026 Oct 30;17:1830730. doi: 10.3389/fpsyg.2026.1830730 (PMC13759799; doi:10.3389/fpsyg.2026.1830730)
Supplement: Supplementary file 1 [file Data_Sheet_1.ZIP › References PDF/The Impact of Positive Psychological Capital on Medical Students’ Innovation and Entrepreneurship Abilities The Multiple Mediating Roles of Entrepreneurial Self-Efficacy and Entrepreneurial Resilience.pdf]

DOI:10.20037/j.issn.1671-1246.2025.20.32

积极心理资本对医学生创新创业能力的影响
——创业自我效能感和创业韧性的多重中介作用

陈凡莹[1],罗 辑[2],谯 攀[3]

(1.贵州医科大学,贵州 贵安新区 561113;2.遵义师范学院,贵州 遵义 563006;3.贵州城市职业学院,贵州 贵阳 550025)

摘 要:目的 探讨积极心理资本、创业自我效能感和创业韧性对医学生创新创业能力的影响机制,并检验创业自我效能感和创业韧性在其中的中介作用,以揭示影响医学生创新创业能力的关键心理因素。方法 采用问卷调查法,收集482名医学生关于积极心理资本、创业自我效能感、创业韧性和创新创业能力的得分,使用SPSS 26.0和Mplus 8.3对收集的数据进行分析并检验。结果 医学生积极心理资本总均分为(4.57±0.67)分,创新创业能力总均分为(3.49±0.48)分,创业自我效能感总均分为(2.63±0.85)分,创业韧性总均分为(3.30±0.56)分。医学生有创业经历者在创新创业能力、积极心理资本、创业自我效能感和创业韧性上的得分均显著高于无创业经历者($P<0.05$)。积极心理资本与创新创业能力呈正相关($P<0.001$),且直接效应显著($\beta=0.649$,$P<0.001$)。创业自我效能感在积极心理资本与创新创业能力间存在部分中介作用,占总效应的9.41%。创业韧性在积极心理资本与创新创业能力间同样存在显著的中介效应,占总效应的8.69%。创业自我效能感和创业韧性在积极心理资本与创新创业能力之间存在链式中介效应,占总效应的2.17%。结论 积极心理资本不仅可直接促进医学生的创新创业能力的提升,还可通过创业自我效能感和创业韧性的中介作用间接提升其创新创业能力,为优化医学生创新创业教育提供了理论依据和实践指导。

关键词:积极心理资本;创新创业能力;创业自我效能感;创业韧性;多重中介效应

中图分类号:G444 文献标识码:B 文章编号:1671-1246(2025)20-0122-06

Impact of Positive Psychological Capital on Medical Students´ Innovation and Entrepreneurship Ability: the Multiple Mediating Role of Entrepreneurial Self-efficacy and Entrepreneurial Resilience

CHEN Fanying[1], *LUO Ji*[2], *QIAO Pan*[3]

(1.Guizhou Medical University, Guian New Area, Guizhou Province, China 561113; 2.Zunyi Normal University, Zunyi City, Guizhou Province, China 563006; 3.Guizhou City Vocational College, Guiyang City, Guizhou Province, China 550025)

Abstract: Objective To explore the impact mechanism of positive psychological capital, entrepreneurial self-efficacy, and entrepreneurial resilience on medical students' innovation and entrepreneurship ability, and to examine the mediating role of entrepreneurial self-efficacy and entrepreneurial resilience in them, so as to reveal medical students' key psychological factors that affect the innovation and entrepreneurship ability. Methods A questronnaire survey was conducted to collect 482 medical students' scores on positive psychological capital, entrepreneurial self-efficacy, entrepreneurial resilience and innovation and entrepreneurship ability, and the collected data were analyzed and tested by SPSS 26.0 and Mplus 8.3. Results The total average score of positive psychological capital for medical students is (4.57± 0.67) score, the total average score of innovation and entrepreneurship ability is (3.49± 0.48) score, the total average score of entrepreneurial self-efficacy is (2.63± 0.85) score, and the total average score of entrepreneurial resilience is (3.30 ± 0.56) score. The scores of medical students with entrepreneurial experience were significantly higher in innovation and entrepreneurship ability, positive psychological capital, entrepreneurial self-efficacy and entrepreneurial resilience than those without entrepreneurial experience ($P<0.05$). Positive psychological capital was positively correlated with innovation and entrepreneurship ability ($P<0.001$), and the direct effect was significant ($\beta=0.649$, $P<0.001$). Entrepreneurship self-efficacy has a partial mediating role between positive psychological capital and innovation and entrepreneurship ability, accounting for 9.41% of the total effect. Entrepreneurial resilience also has a significant mediating effect

基金项目:贵州医科大学创新创业教育实践专项研究课题“后疫情时代地方高校大学生创业者积极心理资本研究及干预”(2022-13)

中国知网 https://www.cnki.net

between positive psychological capital and innovation and entrepreneurship ability, accounting for 8.69% of the total effect. Entrepreneurial self-efficacy and entrepreneurial resilience have a chain mediated effect between positive psychological capital and innovation and entrepreneurship ability, accounting for 2.17% of the total effect. Conclusion Positive psychological capital not only directly promotes the improvement of innovation and entrepreneurship ability of medical students, but also indirectly enhances their innovation and entrepreneurship ability through the mediating role of entrepreneurial self-efficacy and entrepreneurial resilience, providing theoretical basis and practical guidance for optimizing innovation and entrepreneurship education for medical students.
Keywords: Positive psychological capital; Innovation and entrepreneurship ability; Entrepreneurial self-efficacy; Entrepreneurial resilience; Multiple mediating effects

创新创业能力被定义为个体在事业追求和奋斗过程中所表现出的能力总和，它是一个由创造性人格、创新创业素质和关键能力构成的人格-素质-能力系统[1]。创新创业不仅是一种技能，更是一种高度融合的综合能力体系。然而，已有研究表明，当前大学生普遍存在创新创业能力不强、创新意识薄弱、缺乏系统性创新创业观念等问题[2]，这在一定程度上制约了其创新创业实践的有效开展。积极心理资本指个体在成长和发展过程中呈现出的，可塑且可发展的一种积极心理状态[3]。它作为个体发展的重要综合心理资源，可以为个体在创新创业过程中提供持久的心理能量，是大学生创业者是否成功创业的关键，也是影响其创新创业能力水平的重要变量[4]。此外，在创新创业情境中，个体的创新创业行为还会受到自我特征和行为动机的影响，创业自我效能感能让个体在进行创业活动时对自身创业行为能力进行判断和评估，并由此形成对是否能够实现创业行为的自信程度[5]。创业韧性则帮助创业者迅速从创业失败情境中恢复[6]，继续再次创业。这两者都具有特定情境的性质，并且可以作为创业意向和创业目标设定的预测变量[7]。

作为高等教育体系中的特殊组成部分，医科院校的创新创业教育不同于普通高校，应充分结合医学专业的特点与行业需求，培养兼具医学专业素养、创新精神和创业能力的复合型人才[8]。然而，目前针对医学生创新创业教育的系统性研究仍较为有限，现有研究大多关注创新创业能力的培养路径、政策支持与教育模式，而对医学生创新创业能力的影响因素及其心理机制探讨较少。此外，研究多停留在理论分析，缺乏充分的实证支持，对医学生在创新创业中的核心竞争因素、心理动因及认知机制关注较少。因此，本研究拟探讨积极心理资本、创业自我效能感和创业韧性对医学生创新创业能力的影响及其相互关系，构建创新创业能力的心理影响模型，通过实证研究，为医学生创新创业教育的改革与优化提供理论支撑和实践指导。

1 对象与方法

1.1 研究对象

本研究以横断面研究为基础，通过方便取样进行问卷调查，于 2024 年 3—5 月选取贵州省某医科大学就读专业为医学(包括护理和检验)的在校学生作为研究对象。纳入标准：(1) 医学专业(包括护理和检验)的本科生和研究生；(2) 知情同意并自愿参与本研究。排除标准：(1) 非医学专业的本科生和研究生；(2) 不同意参加本研究者。

1.2 研究方法

本研究通过现场调查，在征得被研究者同意后，采取当场发放量表并回收的方式，以确保量表的回收率和完成率。根据样本量计算公式 $n=\frac{z^2 p(1-p)}{e^2}$ 及参考常模样本[9]，计算出本研究所需最低样本量为 385 例，考虑到可能的样本流失，适当增加样本量，最终共发放 600 份问卷，回收 527 份，回收率为 87.8%。在剔除重复作答过多、未全部填写所有问题及随意作答的量表后，最终得到有效问卷 482 份，问卷有效回收率为 80.3%。

1.3 研究工具

1.3.1 一般人口学资料问卷 包括性别、年级、生源地、父母学历以及是否有过创业经历(如在校创业、已注册公司且有实际业务发生、是创业企业股东、曾经或正在参加各类创新创业类项目及比赛)。

1.3.2 积极心理资本量表 该量表[10]共包括 26 个题项，采用 Likert 7 点计分，选项从“1”完全不符合到“7”完全符合，第 8、10、12、14、25 题为反向计分，得分越高代表积极心理资本水平越高。在本研究中 Cronbach′s α 系数为 0.898。

1.3.3 创新创业能力量表 该量表[11]共包括 28 个题项，采用 Likert 5 点计分，从“1”完全不同意到“5”完全同意，得分越高代表创新创业能力越强。在本研究中 Cronbach′s α 系数为 0.922。

1.3.4 创业自我效能感量表 该量表[12]包括 6 个题项，采用 Likert 5 点计分，从“1”完全不同意到“5”完全同意，得分越高代表创业自我效能感越强。在本研究中 Cronbach′s α 系数为 0.927。

1.3.5 创业韧性量表 该量表[13]共包括 10 个题项，采用 Likert 5 点计分，从“1”完全不同意到“5”完全同意，得分越高代表创业韧性越强。在本研究中 Cronbach′s α 系数为 0.855。

1.4 统计学分析

本研究采用 SPSS 26.0 软件进行数据分析，包括共同方法偏差检验、描述性统计分析、独立样本 t 检验以及多重共线性诊断，以确保数据的合理性和稳健性。此外，利用 Mplus 8.3 建模，检验变量之间的中介作用。$P<0.05$ 视为差异有统计学意义。

2 结果

2.1 研究对象的基本情况

本研究 482 名研究对象中，无创业经历者 262 人(54.4%)，有创业经历者 220 人(45.6%)；男生 155 人(32.2%)，女生 327 人(67.8%)；大一 15 人(3.1%)，大二 197 人(40.9%)，大三 146 人(30.3%)，大四 124 人(25.7%)；生源地为城镇及农村的 338 人(70.1%)，生源地为地级市及县级市的 144 人(29.9%)；父母学历为初中及以下的 313 人(64.9%)，为高中的 103 人(21.4%)，为本科及以上的 66 人(13.7%)。

2.2 医学生积极心理资本、创新创业能力、创业自我效能感及创业韧性得分

中国知网 https://www.cnki.net

研究采用的所有量表除积极心理资本量表外，均采用 5 点李克特量表进行测量，得分范围从最低 1 分到最高 5 分，其中位数为 3。创新创业能力和创业韧性的得分均值均高于中位数 3，表明研究对象的创新创业能力和创业韧性处于中等水平，而创业自我效能感得分小于中位数 3，表明研究对象的创业自我效能感处于中等偏下水平。对于积极心理资本量表，由于采用 7 点李克特量表进行测量，得分范围从最低 1 分到最高 7 分，其中位数为 4。积极心理资本均分高于中位数 4，表明研究对象的积极心理资本处于中等水平，见表 1。

表 1　医学生积极心理资本、创新创业能力、创业自我效能感及创业韧性得分情况（$\bar{x}\pm s$，分）

Table 1　Medical students′ scores of positive psychological capital, innovation and entrepreneurship ability, entrepreneurial self-efficacy, and entrepreneurial resilience（$\bar{x}\pm s$, score）

研究对象	积极心理资本	创新创业能力	创业自我效能感	创业韧性
医学生	4.57±0.67	3.49±0.48	2.63±0.85	3.30±0.56

2.3 效度检验

为确保量表的统一性和可比性，本研究对积极心理资本原始的 7 分李克特量表进行了转换，将其调整为 5 分李克特量表。本研究采用 Mplus 8.3 软件进行验证性因子分析（CFA），结果见表 2。四因子模型 v=2.852，CFI=0.911、TLI=0.902、RMSEA=0.062、SRMR=0.047，各项拟合指标均处于合理范围，拟合度较理想，与其他竞争模型相比具有较好的拟合度，表明各变量之间的区分效度达到了统计学要求。

表 2　验证性因子分析结果

Table 2　Results of confirmatory factor analysis

模型	v	χ^2	df	CFI	TLI	RMSEA	SRMR
四因子 X，Y，M1，M2	2.852	906.889	318	0.911	0.902	0.062	0.047
三因子 X，Y，M2+M1	6.574	2 110.233	321	0.729	0.704	0.108	0.105
双因子 X+Y，M1+M2	6.935	2 239.908	323	0.710	0.684	0.111	0.110
单因子 X+Y+M1+M2	7.688	2 490.958	324	0.672	0.644	0.118	0.090

2.4 共同方法偏差检验

采用 Harmans 单因子法与潜变量法评估变量的共同方法偏差[14]。经过检验，在未旋转的主成分分析中提取出 23 个公共因子，解释总变异量的 74.03%。其中第一个因子解释的变异量占比 27.326%，低于 40% 的临界值 引入共同方法偏差潜变量后，验证性因子分析的模型拟合指标变化为 Δv=0.05、ΔCFI=0.004、ΔTIL=0.006，均<0.1，ΔRMSEA=0.002、ΔSRMR=0.006，均小于 0.05。因此，共同方法偏差问题并不严重，可进行进一步的假设分析。

2.5 医学生有创业经历者与无创业经历者在不同变量上的差异

对医学生有创业经历者与无创业经历者在创新创业能力、积极心理资本、创业自我效能感和创业韧性上进行独立样本 t 检验。研究结果显示，医学生有创业经历者在创新创业能力、积极心理资本、创业自我效能感、创业韧性上均显著高于无创业经历者（P<0.05），见表 3。

2.6 多重共线性诊断

为防止各变量间存在严重的多重共线性问题，本研究对各变量进行了方差膨胀因子（VIF）检验，结果见表 4。所有被试各变量的均值与标准差均处于合理范围，变量间相关系数正常且具有相关关系（P<0.001），且所有变量的 VIF（1.554~1.910）均小于 3，说明数据不存在严重共线性问题，适合进行进一步检验。

表 3　医学生有创业经历者与无创业经历者在不同变量上的差异（$\bar{x}\pm s$，分）

Table 3　Differences in different variables between medical students with and without entrepreneurial experience（$\bar{x}\pm s$, score）

变量	有创业经历者（n=220）	无创业经历者（n=262）	t	P
创新创业能力	3.57±0.47	3.42±0.487	3.436	0.001
积极心理资本	3.44±0.44	3.35±0.45	2.372	0.018
创业自我效能感	2.77±0.84	2.51±0.84	3.397	0.000
创业韧性	3.47±0.53	3.16±0.54	6.313	0.001

表 4　多重共线性诊断结果

Table 4　Results of multicollinearity diagnosis

变量	均值	标准差	创新创业能力	积极心理资本	创业韧性	创业自我效能感	VIF
创新创业能力	3.49	0.48	1				
积极心理资本	3.39	0.45	0.657***	1			1.554
创业韧性	3.30	0.56	0.604***	0.582***	1		1.910
创业自我效能感	2.63	0.85	0.514***	0.355***	0.540***	1	1.559

注：***P<0.001。

2.7 中介效应检验

2.7.1 各变量间的相关性分析 为检验各变量的相关关系，本研究使用 Mplus 8.3 软件进行相关路径分析。在控制了性别、年级、父母学历等变量后，首先将创业自我效能感作为因变量、积极心理资本作为自变量，结果显示，积极心理资本与创业自我效能感（β=0.389，SE=0.044，P<0.001）呈正相关。其次将创业韧性作为因变量、积极心理资本和创业自我效能感作为自变量，结果显示，积极心理资本（β=0.494，SE=0.037，P<0.001）、创业自我效能感（β=0.325，SE=0.036，P<0.001）均与创业韧性呈正相关。最后，将创新创业能力作为因变量，积极心理资本、创业自我效能感、创业韧性作为自变量，结果显示，积极心理资本（β=0.649，SE=0.045，P<0.001）、创业自我效能感（β=0.197，SE=0.040，P<0.001）、创业韧性（β=0.143，SE=0.051，P<0.001）均与创新创业能力呈正相关。直接效应获得验证，可以进行下一步分析。变量之间的关系模型见图 1。

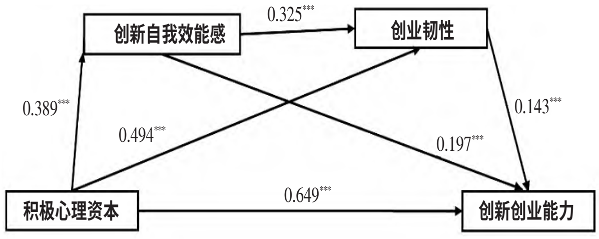

注：***P<0.001。

图 1　变量之间的关系模型

Figure 1　Relationship model among variables

2.7.2 多重中介效应的 Bootstrap 法检验 对于多重中介效应的检验，本研究参考方杰等[15]的研究，采用 Bootstrap 自抽样 1 000次的方法进行分析。研究结果显示，积极心理资本对创新创业能力的总效应显著（效应值=0.829，95% 置信区间=[0.658，0.982]）。直接效应值=0.649，95% 置信区间=[0.550，0.739]，不包括 0，直接效应显著。创业自我效能感在积极心理资本与创新创业能力之间的中介效应值为 0.078，95%置信区间=[0.042，0.127]，不包括 0，中介效应显著，间接效应占比为

中国知网　https://www.cnki.net

9.41%（0.078/0.829）。创业韧性的中介效应值为 0.072，95%置信区间=[0.015，0.133]，不包括 0，说明中介效应成立，间接效应占比为 8.69%（0.072/0.829）。创业自我效能感与创业韧性在积极心理资本与创新创业能力之间的中介效应值为 0.018，95%置信区间=[0.004，0.040]，不包括 0，说明中介效应成立，间接效应占比为 2.17%（0.018/0.829）。总间接效应值=0.168，95%置信区间=[0.100，0.250]，不包括 0，总间接效应占比为 20.27%（0.168/0.829）。此外，研究还发现模型 2 与模型 3 的差值为 0.06，95%的置信区间=[0.011，0.171]，说明模型 2 比模型 3 更能解释积极心理资本对创新创业能力的作用机制。多重中介模型的间接效应和直接效应的结果见表 5。

表 5 多重中介模型的间接效应和直接效应结果

Table 5 Results of indirect and direct effects of the multiple mediation model

中介模型	效应值	效应值占比/%	Bootstrap95%置信区间	
			上限	下限
总效应	0.829		0.658	0.982
直接效应	0.649		0.550	0.739
总间接效应	0.168	20.27	0.100	0.250
积极心理资本→创业自我效能感→创新创业能力（模型 1）	0.078	9.41	0.042	0.127
积极心理资本→创业韧性→创新创业能力（模型 2）	0.072	8.69	0.015	0.133
积极心理资本→创业自我效能感→创业韧性→创新创业能力（模型 3）	0.018	2.17	0.004	0.040

3 讨论

3.1 医学生积极心理资本、创新创业能力和创业韧性处于中等水平，创业自我效能感处于中等偏下水平

研究结果表明，医学生的积极心理资本（4.57±0.67）分与创业韧性（3.30±0.56）分均处于中等水平，与相关作者研究一致[10,13]，这一结果表明，医学教育体系在培养自信心、抗压能力和责任意识方面具有一定成效，但仍有提升空间。积极心理资本和创业韧性被认为是影响个体创业动机和创业成功的重要心理资源，其水平高有助于促使医学生在创业过程中保持积极心态[16]，具备一定的坚持力和抗挫折能力，可以增强创业成功的可能性。值得注意的是，创新创业能力虽处于中等水平（3.49±0.48）分，但低于已有的同类研究结果[9]，而创业自我效能感则处于中等偏下水平（2.63±0.85）分。这说明医学生虽然对创新创业活动具有一定的理解和实践能力，但医学教育对专业技能的侧重与创新创业教育资源的相对匮乏，可能导致医学生在市场开拓、风险管理等实践能力上存在短板。

3.2 医学生有创业经历者的积极心理资本、创新创业能力、创业自我效能感和创业韧性水平均高于无创业经历者

本研究通过实证分析，探讨了医学生有创业经历者与无创业经历者在积极心理资本、创新创业能力、创业自我效能感和创业韧性上的差异。结果发现，医学生有创业经历者的各变量得分均高于无创业经历者（P<0.05），表明创业实践可能在提升医学生积极心理资本、创新能力及创业信心方面发挥了积极作用。创新创业能力是一种系统性、动态性的能力，其发展受到个体内部心理状态的影响[1]。已有研究指出，成就动机、内控倾向和风险承担等心理因素是影响创新创业能力的重要先决条件，而自我效能感、个人目标、愿望和动机则直接塑造创新创业能力[17-18]。同时，面对不确定环境时，高容忍度的创业者比低容忍度的创业者具备更强的创新创业能力[19]，本研究结果支持了这一观点，即有创业经历的医学生可能具备更强的创新创业意识、实践能力和创业学习动机。此外，创业教育与实践经历能够增强医学生对自身成功实施创业行为的信心，提高其心理韧性，使其在面对创业挫折时能够快速调整并持续推进创业进程[20]。创业活动本身具有高风险、高不确定性、高负荷和高压力的特点，因此创业者需要储备足够的心理资源来应对挑战。积极心理资本中的乐观、希望、自信等特质能够帮助创业者在面临挑战时保持积极态度[21-24]，提高创业成功的可能性。

3.3 积极心理资本、创业自我效能感、创业韧性与创新创业能力的相关关系

3.3.1 医学生积极心理资本与创新创业能力得分呈正相关关系

本研究相关分析结果表明，积极心理资本与创新创业能力呈显著正相关（β=0.649，P<0.001），这表明医学生的积极心理资本水平越高，其创新创业能力越强。这一结果支持了积极心理资本在创新创业活动中的关键作用。已有研究指出，积极心理资本是创新创业能力的前提条件，其核心构成——自我效能感、希望感、乐观性和心理韧性，能够为个体提供持续的心理能量，有效增强创业者在不确定环境下的适应能力和决策能力，从而提升创业成功率[4]。

3.3.2 医学生积极心理资本和创业自我效能感得分与创业韧性得分呈正相关关系

研究结果显示，积极心理资本（β=0.494，P<0.001）和创业自我效能感（β=0.325，P<0.001）与创业韧性均呈显著正相关，这表明医学生的积极心理资本和创业自我效能感越高，其创业韧性越强。创业韧性代表个体在面对创业挑战和不确定性时的坚持、适应和应对能力[13]。而积极心理资本中的坚韧、机智、乐观特质能够提高个体对不稳定环境的适应能力，帮助创业者更好地应对商业环境带来的挑战，增加面对创业风险时的信心与抗挫能力[25]。同时，创业自我效能感体现了个体对自身创业能力的感知，以及在创业情境中成功完成任务的信心[26]，这一心理特质能够激发更高水平的自我期望和持久努力，从而进一步强化应对创业挑战时的心理韧性。积极心理资本与创业自我效能感的结合能使个体在创业过程中激发内部动机、认知资源并最终采取合理行动。通过不断积累成功经验与心理资源，帮助创业者展现更强的创新意识和执行意志，还能在遭遇困难与挫折时保持积极主动性和高度的目标承诺，提升医学生的创新创业能力，使其坚定实现创新创业目标的信念，保持长期的创业动力。

3.3.3 医学生创业自我效能感和创业韧性得分均与创新创业能力得分呈正相关关系

研究结果表明，创业自我效能感（β=0.197，P<0.001）和创业韧性（β=0.143，P<0.001）均与创新创业能力显著正相关。这表明医学生的自我效能感越高，越能在创业过程中保持积极心态、增强创业韧性，并最终提升创新创业能力。已有研究指出，创业自我效能感是影响创业动机、行为和成功率的关键心理因素[26]。自我效能感越高，个体的内在行为动机越强，越容易产生创新创业行为[27]。因此，个体对自己创新创业行为的信心能够使个体面对不确定情境时保持更积极乐观的态度并增强创业韧性。

3.4 医学生创业自我效能感和创业韧性在积极心理资本与创

中国知网 https://www.cnki.net

新创业能力之间起部分中介作用

研究结果显示，创业自我效能感和创业韧性不仅直接影响创新创业能力，还在积极心理资本与创新创业能力之间扮演了部分中介角色。首先，积极心理资本对创新创业能力的总效应显著（效应值=0.829），其中直接效应为 0.649，而总间接效应占比 20.27%。这一结果进一步支持了积极心理资本作为个体内在资源的重要性，其不仅直接赋能创新创业能力，还通过激发创业者的创业自我效能感和创业韧性，形成多维度的驱动机制。

其次，从具体中介路径来看，创业自我效能感的中介效应（效应值=0.078，占比 9.41%）与创业韧性的中介效应（效应值=0.072，占比 8.69%）贡献相近，表明两者均是积极心理资本促进创新创业能力提升的关键心理机制。创业自我效能感和创业韧性被视为影响个体创新创业行为选择、努力程度及持久性的重要心理状态[27]。当个体具有较高的创业自我效能感和创业韧性时，可以更快地从创业逆境中恢复稳定，也更有可能采取积极态度，克服创业困难，实现创业目标[20]。

最后，创业自我效能感和创业韧性在积极心理资本与创新创业能力之间的链式中介效应显著（效应值=0.018，中介效应占比 2.17%），这一结果表明，积极心理资本可以先通过提高创业自我效能感，进而增强创业韧性，最终促进创新创业能力的提升。根据 Ajzen[28]提出的计划行为理论，个体是否能够从事创新创业行为主要受行为态度、感知行为控制和行为意图的影响。其中，行为态度指个体对创新创业行为的正面或负面评价，态度越积极，行为意图越强，也就是说，个体对创新创业行为的结果越乐观，越有可能实施该行为。这一发现进一步揭示了医学生积极心理资本对创新创业能力的作用机制，说明医学生的创业自我效能感和创业韧性在其中发挥了相互促进的作用。

4 结语

本研究证实了积极心理资本、创业自我效能感、创业韧性对创新创业能力的正向促进作用，并揭示了创业自我效能感和创业韧性在其中的多重中介机制。这一发现不仅深化了对积极心理资本如何影响创新创业能力的理论理解，也拓展了创业自我效能感与创业韧性在该过程中发挥作用的心理路径，构建了更为系统化的心理影响模型。本研究结果对医学院校培养契合社会需求的高素质创新创业型人才具有重要的实践指导意义。未来研究可进一步从积极心理学和积极组织行为学的视角，系统探讨大学生创新创业能力的培养、积累与开发策略，以优化创业教育体系。此外，基于多重中介模型，应构建“心理赋能—实践强化—评价激励”的三维干预体系，推动医学教育从专业型向复合创新型人才培养模式的转型，提升医学生在复杂创业环境中的适应能力与实践能力。

参考文献：

[1]王洪才.创新创业能力评价：高等教育高质量发展的真正难题与破解思路[J].江苏高教，2022（11）：39-46.

[2]刘夏菡.大学生创新创业能力研究[J].西部素质教育，2022，8（8）：4-7，22.

[3]LUTHANS F，AVOLIO B J，AVEY J B，et al.Positive psychological capital：measurement and relationship with performance and satisfaction[J].Personnel Psychology，2007，60（3）：541-572.

[4]ABBAS M，RAJA U.Impact of psychological capital on innovative performance and job stress [J].Canadian Journal of Administrative Sciences，2015，32（2）：128-138.

[5]CHEN C C，GREENE P G，CRICK A.Does entrepreneurial self-efficacy distinguish entrepreneurs from managers? [J].Journal of Business Venturing，1998，13（4）：295-316.

[6]胡文安，江岩，罗瑾琏.新常态下高校大学生社会网络与创业意向关系：创业心理弹性的解释[J].科技进步与对策，2016，33（19）：125-131.

[7]BOYD N G，VOZIKIS G S.The Influence of self-efficacy on the development of entrepreneurial intentions and actions [J].Entrepreneurship Theory and Practice，1994，18（4）：63-77.

[8]安益强，胡俊峰.医学高校创新创业教育的特征及价值研究[J].卫生职业教育，2021，39（8）：5-6.

[9]王洪才，郑雅倩.大学生创新创业能力测量及发展特征研究[J].华中师范大学学报（人文社会科学版），2022，61（3）：155-165.

[10]张阔，张赛，董颖红.积极心理资本：测量及其与心理健康的关系[J].心理与行为研究，2010，8（1）：58-64.

[11]王辉，张辉华.大学生创业能力的内涵与结构：案例与实证研究[J].国家教育行政学院学报，2012（2）：81-86.

[12]LIÑÁN F，CHEN Y W.Development and cross-cultural application of a specific instrument to measure entrepreneurial intentions [J].Entrepreneurship Theory and Practice，2009，33（3）：593-617.

[13]张秀娥，李梦莹.创业韧性的驱动因素及其对创业成功的影响研究[J].外国经济与管理，2020，42（8）：96-108.

[14]赵富强，胡思源，陈耘，等.创业韧性对再创意愿的影响：双元学习与反事实思维的作用[J].科学学研究，2022，40（3）：505-515.

[15]方杰，温忠麟，张敏强，等.基于结构方程模型的多重中介效应分析[J].心理科学，2014，37（3）：735-741.

[16]邹长华，信中贵，李泳汉，等.大学生情绪调节自我效能感和积极心理资本在心理弹性与心理健康关联中的作用[J].中国学校卫生，2023，44（1）：94-98.

[17]LANS T，HULSINK W，BAERT H，et al.Entrepreneurship education and training in a small business context：insights from the competence-based approach[J].Journal of Enterprising Culture，2008，16（4）：363-383.

[18]MCMULLEN J S，SHEPHERD D A.Entrepreneurial action and the role of uncertainty in the theory of the entrepreneur [J].Academy of Management Review，2006，31（1）：132-152.

[19]AHMAD N H，RAMAYAH T，WILSON C，et al.Is entrepreneurial competency and business success relationship contingent upon business environment? A study of Malaysian SMEs [J].International Journal of Entrepreneurial Behavior & Research，2010，16（3）：182-203.

[20]郝喜玲，涂玉琦，刘依冉.失败情境下创业者韧性对创业学习的影响研究[J].管理学报，2018，15（11）：1671-1678，1712.

[21]李力，廖晓明.积极心理资本：测量及其与工作投入的关系：基于高校积极组织管理的视角[J].江西社会科学，2011，31（12）：204-207.

[22]郭郁，王智庆.大学生心理资本对创业意愿的影响研究：以学业成就为中介[J].山西师范大学学报（自然科学版），2018，32（3）：125-128.

[23]励骅，曹杏田.大学生心理资本与就业能力关系研究[J].中国高教研究，2011（3）：54-56.

[24]吴能全，李芬香.创业者心理资本、人力资本与社会资本对其创业能力的影响研究：基于结构方程模型的分析[J].湖南大学学报（社会科学版），2020，34（4）：39-46.

中国知网 https://www.cnki.net

DOI:10.20037/j.issn.1671-1246.2025.20.33

护理院老年人照护者压力性损伤认知现状及其影响因素分析

詹文娴[1],李翠华[2],钟 琴[1]

(1.上海健康医学院,上海 201318;2.上海市东海老年护理医院,上海 201304)

摘 要:目的 调查老年人照护者压力性损伤认知现状并分析其影响因素。方法 选取2023年12月—2024年2月在上海市某2所护理院工作的220例老年人照护者作为研究对象。采用一般资料调查表、照护者压力性损伤认知问卷和压力性损伤照顾行为问卷进行调查,采用多元线性回归分析了解老年人照护者压力性损伤认知水平的影响因素。结果 220例护理院老年人照护者压力性损伤认知总分为(52.12±4.46)分。多元线性回归分析结果表明,照顾者的性别、有无职业技能培训证书、接受压力性损伤预防相关培训情况、压力性损伤照顾行为的翻身行为是老年人照护者压力性损伤认知的影响因素(P<0.05)。结论 老年人照护者压力性损伤认知处于中等偏上水平,仍需加强相关知识的教育和培训,改善照顾行为,以降低老年人照护者压力性损伤的发生率。

关键词 老年人照护者;压力性损伤;照顾行为

中图分类号:R195 文献标识码:B 文章编号:1671-1246(2025)20-0127-04

The Cognitive Status of Pressure Injuries Among Elderly Caregivers in Nursing Homes and Analysis on Influencing Factors

ZHAN Wenxian[1], *LI Cuihua*[2], *ZHONG Qin*[1]

(1.Shanghai University of Medicine & Health Sciences, Shanghai City, China 201318;
2.Shanghai Donghai Elderly Care Hospital, Shanghai City, China 201304)

Abstract: Objective To investigate the cognitive status of pressure injuries among elderly caregivers and analyze its influencing factors. Methods A total of 220 elderly caregivers working in two nursing homes in Shanghai from December 2023 to February 2024 were selected as the research subjects. A General Information Questionnaire, a Caregiver Pressure Injury Cognition Questionnaire, and a Pressure Injury Care Behavior Questionnaire were used for the survey. Multiple linear regression analysis was employed to identify the influencing factors of pressure injury cognition among elderly caregivers. Results The total score for pressure injury cognition among 220 elderly caregivers in nursing homes was (52.12± 4.46)score.The results of multiple linear regression analysis indicated that the caregivers' gender, being certified with vocational skills training certificates or not, receiving training related to pressure injury prevention, and turning behavior in pressure injury care were significant influencing factors on caregivers' cognition of pressure injuries for the elderly(P<0.05). Conclusion The level of pressure injury cognition among elderly caregivers is moderate to high, and further education and training on related knowledge are still needed to improve care behaviors and reduce the incidence of pressure injuries among the elderly caregivers.

Keywords: Elderly caregivers; Pressure injuries; Caregiving behaviors

[25]FATOKI O.The impact of entrepreneurial resilience on the success of small and medium enterprises in South Africa [J].Sustainability,2018,10(7):2527.

[26]曹之然.创业自我效能感:已有研究成果与未来研究展望[J].科技创业月刊,2023,36(11):190-198.

[27]AMABILE T M.Motivational synergy:toward new conceptualizations of intrinsic and extrinsic motivation in the workplace[J].Human Resource Management Review,1993,3(3):185-201.

[28]AJZEN I.The theory of planned behavior[J].Organizational Behavior and Human Decision Processes,1991,50(2):179-211.

作者简介:陈凡莹(1983—),女,在读博士研究生,讲师。研究方向:大学生心理健康教育。▲

中国知网 https://www.cnki.net
