# Supplementary material for: A study on the current status and influencing factors of psychological capital among medical undergraduates
Source: Front Psychol. 2026 Oct 30;17:1830730. doi: 10.3389/fpsyg.2026.1830730 (PMC13759799; doi:10.3389/fpsyg.2026.1830730)
Supplement: Supplementary file 1 [file Data_Sheet_1.ZIP › References PDF/The Impact of Proficiency in the Five Arts on Medical Students’ Self-Efficacy in Career Decision-Making The Chain Mediation of Psychological Capital and Positive Coping Strategies.pdf]

五术素养对医学生职业决策自我效能感的影响
——心理资本和积极应对方式的链式中介作用

孙建华[1],张博[1],彭青和[2],许胜[2],胡霞[2]

(安徽中医药大学,安徽 合肥 230012 1.医药经济管理学院;2.人文与国际交流学院)

摘要:为探索五术素养对医学生职业决策自我效能感的影响,采用五术素养量表、职业决策自我效能感量表、心理资本量表、积极应对方式量表进行抽样调查。结果显示,职业决策自我效能感在性别($t=2.49$,$P<0.01$)、生源地($t=2.69$,$P<0.01$)等人口学变量差异有统计学意义;五术素养、职业决策自我效能感、心理资本、积极应对方式间存在明显正向关系;心理资本和积极应对方式在医学生五术素养和职业决策自我效能感之间的链式中介效应显著。因此,新时代下高校应该以具备"道术、仁术、学术、技术、艺术"的"五术"医者新内涵强化医学生培养为目标,职业决策自我效能感作为关键心理资源,有机融合多方资源提升医学生的"五术"素养、心理资本和积极应对方式。

关键词:医学生;五术素养;心理资本;积极应对方式;职业决策自我效能感;链式中介

中图分类号:G641　　**文献标志码**:A　　**文章编号**:1009-4784(2025)04-0031-06

职业决策是指大学生在教育阶段根据个人兴趣、能力素养、价值观和社会需求等因素选择职业方向的过程。职业决策在大学生确定职业路径、提高就业竞争力、识别个人强项与短板、减少职业选择风险、增强自信心、规划个人发展等方面具有重要意义,职业决策自我效能是大学生在进行职业决策过程中对胜任工作的信心评估[1-3],近年来,由于医学教育的高压环境、职业路径的复杂性以及就业市场的不确定性等,部分医学生在面对职业选择时表现出犹豫、焦虑,能力与信心的双重缺失,如何建立医学生对职业决策的掌控感,已成为当前医学教育领域值得关注的重要问题。

积极应对方式是指个体在面对挑战和决策时,采取主动的态度和方法来面对问题。大学生的应对方式在职业兴趣与职业决策自我效能感中起调节作用[4],积极应对方式正向影响职业决策自我效能感[5]。心理资本是指个体在成长和发展过程中表现出来的一种积极心理状态,促进个人成长和绩效提升的心理资源。国内外研究表明,大学生职业决策自我效能感和心理资本成正相关[6-7]。

2020 年 9 月国务院办公厅印发《关于加快医学教育创新发展的指导意见》[8],指出要以新内涵强化医学生培养,加强救死扶伤的道术、心中有爱的仁术、知识扎实的学术、本领过硬的技术、方法科学的艺术的教育(以下简称"五术医学教育")。医学五术素养是指医学教育中强调的五个方面的素养,包括道术、仁术、学术、技术和艺术。这旨在培养医德高尚、医术精湛的医学人才,以应对现代医学教育和临床实践的需求。

医学生职业决策过程中,以新内涵提出的"道术、仁术、学术、技术、艺术"五术能力素养[8],是影响卫生健康职业决策自我效能感的重要因素。面对五术融合医学职业就业的新形势、新要求,何碧如等提出医学类院系"五术"融合就业指导课程,探索将"五术"融入医学生"就业指导"课并进行教学实践[9]。而关于医学生的五术素养水平如何影响职业决策自我效能感,目前尚未有研究。

本研究整合职业发展、积极心理学与医学教育

收稿日期:2024-10-28
资助项目:安徽中医药大学"大思政课"研究专项课题(2022AH010038-6);安徽中医药大学校级教学研究(重点)项目(2024xjjy_zd011)
作者简介:孙建华,女,讲师,心理学硕士。研究方向:基础心理学。
通信作者:张博,Email:57263438@qq.com

中国知网 https://www.cnki.net

领域的经典理论框架,旨在从心理学角度探索医学生的五术素养对职业决策自我效能感的影响,通过构建定量五术素养量表模型,探讨医学生五术素养对职业决策自我效能感的影响,建立基于医学生“五术”职业就业目标的定量统计模型,阐述五术素养与职业决策的直接关系,心理资本和积极应对方式与职业决策的间接关系,揭示变量间的理论逻辑,为医学生在职业就业过程中提供可行的实践方式,基于五术新型医学人才培养目标,通过增强医学生心理资本自信心、积极心理状态、专业素养等方式,提供精准化的职业教育与辅导,针对性提升技能,深入了解兴趣与能力,做出明智的职业决策,发挥五术素养牵引作用,助力医学生职业素养与职业决策的融合。

一、研究设计与数据来源

(一)数据来源

本研究数据来源于随机抽样调查,对安徽省合肥市内高校(安徽中医药大学、安徽医科大学、皖南医学院、安徽医学高等专科学校)学生发放调查问卷 694 份,其中有效问卷 670 份,有效率 97%。调研问卷按照性别、生源地、是否担任学生干部、学校类别、是否贫困生、年级等进行研究对象定量框定,其中男生 202 人,城市学生 467 人,本科学生 379 人,256 人担任过学生干部(表 1)。

表 1　人口学统计情况

变量	类别	人数	占比/%
性别	男	202	30
	女	468	70
生源地	农村	203	30
	城市	467	70
担任学生干部	是	256	38
	否	414	62
学校类别	专科	291	43
	本科	379	57
是否贫困生	是	198	30
	否	472	70
年级	大一	328	49
	大二	92	14
	大三	136	20
	大四	99	15
	大五	10	1
	研究生	5	1
家庭教养方式	民主型	527	79
	专制型	62	9
	溺爱型	15	2
	忽视型	66	10

注:$n=670$。

(二)研究设计

本研究设计五术素养、职业决策自我效能感、心理资本、应对方式调研问卷随机抽样调查。

1. **五术素养问卷**。本研究采用沈慧等编制的医学生五术素养评定量表[10],该量表共 19 个条目,分为思想道德维度(1～8 题)与行为实践维度(9～19 题),思想道德维度包含道术、仁术,行为实践维度包含学术、技术以及艺术,其中,采用李克特 Likert 五级评分法(1～5 分),“非常反对”计分为 1 到“完全赞同”计分为 5,分数越高,则代表个体的五术素养水平越高。本次研究中,问卷内部一致性信度 Cronbach's α 系数 0.974,信度较好。

2. **职业决策自我效能感问卷**。本研究采用彭永新等编制的职业决策自我效能感量表[11],该量表采用 Likert 五级评分法(1～5 分),共 25 个条目,得分与职业决策自我效能感呈正比例。本次研究中问卷内部一致性信度 Cronbach's α 系数 0.975,信度较好。

3. **心理资本问卷**。本研究采用张阔等编制的积极心理资本问卷方法(PPQ)[12]。问卷共 26 道题目,分为心理韧性、希望、乐观和自我效能 4 个维度,采用 Likert 七点计分(1～7 分)。本次研究中问卷内部一致性信度 Cronbach's α 系数为 0.928,可信采纳。

4. **应对方式问卷**。采用解亚宁等编制的简易应对方式量表[13],采用李克特 Likert 四级评分法,由积极应对(1～12 题)和消极应对(13～20 题)组成,包括 20 道题目,分值越高该倾向越高,本次研究中,积极应对方式分量表、消极应对方式分量表 Cronbach's α 系数分别为 0.933 和 0.858,信度较好。

(三)数据统计处理

研究采用 SPSS 22.0 软件进行处理分析,剔除存在问题项的问卷调研数据后,进行人口学统计的差异检验、变量之间的 Pearson 相关分析,并用 SPSS 22.0 插件 Process 3.5[13] 进行中介作用检验和 Bootstrap 分析。

二、数据分析与研究结果

(一)数据分析

研究从公共方法偏差的控制与检验、人口学变量差异检验、变量之间的相关分析等方面进行数据分析,分析如下。

1. **共同方法偏差的控制与检验**。研究通过被试匿名测量、部分项目反向等措施从程序上控制共

中国知网 https://www.cnki.net

同方法偏差。本研究共 4 份调查问卷合计 89 道题目，采用 Harman 单因素共同方法偏差检验[14]，对研究变量的全量问卷进行验证性因子分析，结果显示，KMO 的值为 0.97，Bartlett 值为 59 223.59，$df=3\ 916$，$P<0.001$，未旋转的探索性因子分析结果提取出 10 个特征根大于 1 的因子，共可解释全部方差的 70.08%，第一个主因子的特征值为 32.54，解释率为 36.56%，低于 40%的临界值，说明不存在明显的共同方法偏差问题，数据可以进一步统计分析。

2. 人口学变量差异检验。本研究采用独立样本 t 检验和单因素方差分析，对职业决策自我效能感在学校类别、是否贫困生、年级等人口学变量上的差异进行检验（表 2），性别、生源地、是否担任学生干部以及家庭教养方式的得分差异有统计学意义（$P<0.01$），表现为男性的得分高于女性，生源地为农村的得分高于城市，学生干部的得分高于非干部，民主型家庭教养的得分高于专制型，而职业决策自我效能感在学校类别、是否贫困生以及年级的得分差异无统计学意义。

3. 变量间相关分析。采用 Pearson 积差相关对五术素养、心理资本、应对方式、职业决策之间进行变量间相关分析（表 3），五术素养与心理资本、积极应对维度、职业决策自我效能感之间呈正相关关系（$P<0.01$），心理资本与积极应对方式、职业决策之间呈正相关（$P<0.01$），积极应对方式与职业决策自我效能感之间呈正相关（$P<0.01$），为进一步中介效应检验提供了基础。

（二）研究结果

1. 五术素养正向影响心理资本、积极应对方式、职业决策自我效能感。本研究基于数据分析，研究链式中介效应检验结果。研究显示，变量间相互关联，满足中介效应检验的前提条件，采用 PROCESS Model 6（Hayes）回归分析并进行中介模型检验，以职业决策自我效能感作为因变量，五术素养作为自变量，心理资本和积极应对方式作为中间变量（表 4）。

表 2　职业决策自我效能感分数在人口学变量上的差异检验结果

项目	分组	职业决策自我效能感
性别	男	92.42±18.98
	女	88.63±17.69
	t	2.49**
生源地	农村	92.63±18.90
	城市	88.54±17.70
	t	2.69**
担任学生干部	是	92.56±17.06
	否	88.05±18.62
	t	3.14**
学校类别	专科	88.67±19.05
	本科	90.62±17.42
	t	−1.38
是否贫困生	是	89.10±17.87
	否	90.06±18.29
	t	−0.33
年级	大一	18.73±1.03
	大二	16.85±1.76
	大三	16.76±1.44
	大四	18.06±1.82
	大五	23.70±7.50
	研究生	18.75±8.39
	F	2.22
家庭教养方式	民主型	90.79±17.91
	专制型	90.03±19.22
	溺爱型	84.47±13.14
	忽视型	82.65±18.62
	F	4.43**

注：表中自我效能感数据为 $\overline{X}\pm S$；* $P<0.05$，** $P<0.01$，*** $P<0.001$。

表 3　变量间的相关分析结果

变量	五术素养	心理资本	积极应对维度	职业决策自我效能感
1	1			
2	0.41**	1		
3	0.37**	0.64**	1	
4	0.42**	0.71**	0.62**	1

注：$N=670$；* $P<0.05$，** $P<0.01$。

表 4　变量关系的回归分析结果

结果变量	预测变量	β	t	R^2	F
心理资本	五术素养	0.73	11.92***	0.24	22.57
积极应对方式	五术素养	0.08	4.48***	0.43	49.31
	心理资本	0.17	16.56***		
职业决策自我效能感	五术素养	0.18	4.36***	0.57	78.45
	心理资本	0.37	13.60***		
	积极应对方式	0.70	7.78***		

注：* $P<0.05$，** $P<0.01$，*** $P<0.001$。

中国知网　https://www.cnki.net

结果发现,医学生五术素养能够正向影响心理资本($\beta=0.73$,$t=11.92$,$P<0.001$);医学生五术素养($\beta=0.08$,$t=4.48$,$P<0.001$)和心理资本($\beta=0.17$,$t=16.56$,$P<0.001$)能够正向影响积极应对方式;医学生五术素养($\beta=0.18$,$t=4.36$,$P<0.001$)、心理资本($\beta=0.37$,$t=13.60$,$P<0.001$)、积极应对方式($\beta=0.70$,$t=7.78$,$P<0.001$)能够正向影响职业决策自我效能感。

2.五术素养直接影响职业决策自我效能感,五术素养通过心理资本、积极应对方式间接影响职业决策资源效能感。模型检验结果表明(表 5,图 1),五术素养对职业决策自我效能感的直接效应为 0.18,95%的 95%CI 为(0.10,0.25),直接效应显著。心理资本作为单一中介效应的间接效应为 0.27,95%的 95%CI 为(0.20,0.34),间接效应显著。积极应对方式作为单一中介效应的间接效应为 0.05,95%的 95%CI 为(0.02,0.09),间接效应显著。链式中介效应为 0.08,95%的 95%CI 为(0.05,0.12),链式中介效应显著。根据直接效应和各间接效应相加可得五术素养对职业决策自我效能感的总效应为 0.58。直接效应占总效应的 30.07%,链式中介效应占总效应的 14.37%。综上表明,心理资本与积极应对方式在医学生五术素养和职业决策自我效能感之间起部分中介作用。

表 5 中介效应显著性检验的 Bootstrap 分析

路径	效应值	标准误	95%CI		效应占比/%
			下限	上限	
五术素养—职业决策自我效能感(直接效应)	0.18	0.04	0.10	0.25	30.07
五术素养—心理资本—职业决策自我效能感(单一中介效应)	0.27	0.04	0.20	0.34	46.32
五术素养—积极应对方式—职业决策自我效能感(单一中介效应)	0.05	0.02	0.02	0.09	9.24
五术素养—心理资本—积极应对方式—职业决策自我效能感(链式中介效应)	0.08	0.02	0.05	0.12	14.37
五术素养—职业决策自我效能感(总效应)	0.58	0.05	0.49	0.67	

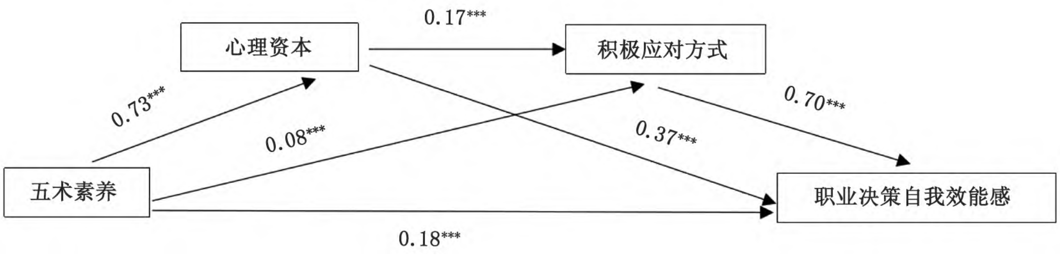

注:*** $P<0.001$。

图 1 五术素养与职业决策自我效能感的链式中介模型

三、研究结论与对策建议

本研究结果表明,医学生五术素养通过心理资本和积极应对方式对职业决策自我效能感产生直接影响,在构建链式中介模型的过程中发现了 3 条中介效应影响路径:心理资本的单独中介作用、积极应对方式的单独中介作用及两者间的链式中介作用。

(一)研究结论

1.人口学变量与职业决策自我效能感呈显著相关。本研究结果显示,职业决策自我效能感在性别上存在明显差异,男性高于女性的结果与中国传统的社会文化以及性别角色期待有关[15]。职业决策自我效能感与学生班级职位存在显著差异,学生干部职业决策自我效能感显著[16]。职业决策自我效能感在生源地上表现为农村高于城市,这与之前的研究结果不一致[16],随着城乡一体化,农村和城镇的生活水平、生活方式差异变小,导致医学生的效能感水平生源地差异缩小。家庭教养方式上,民主型家庭氛围的医学生拥有更加独立的人格以及更高的自尊自信水平,其职业决策自我效能感显著高于专制型、溺爱型和忽视型的家庭[17]。

2.五术素养直接影响职业决策自我效能感。研究发现,医学生五术素养越高,职业决策自我效能感水平越高,提示高校在生涯教育中,要培养医学生的高尚的道德情操和以患者为中心的人文精神,提高医学生的基础知识和临床技能,从而进一步调整和优化学生自身认知和职业认知,增加医学生的求职信心和求职能力,提高职业决策自我效能感。

3.心理资本在五术素养和职业决策自我效能感之间呈中介作用。研究发现,五术素养与心理资

中国知网 https://www.cnki.net

本之间是正相关关系,且存在相互正向影响的因果关系,医学生的心理资本水平越高,五术素养越高,职业决策自我效能感越高,心理资本在医学生五术素养和职业决策自我效能感之间起链式中介作用,职业决策自我效能受心理资本水平的影响,心理资本水平越高,就越相信自己能够应对就业过程中的各种任务[18],高校教育工作者在大学生职业和就业指导工作中,要注意通过开发大学生心理资本的策略和途径,提高大学生完成各种职业选择任务的自我效能,提升就业竞争力。

4.积极应对方式在五术素养和职业决策自我效能感之间呈中介作用。研究发现,积极应对方式在医学生五术素养和职业决策自我效能感之间发挥中介作用,前人研究中,积极应对方式与职业决策自我效能感正相关[19],积极应对方式可以改善医学生面对问题时的认知态度和行为反应,在职业选择遇到未知挑战时会更加积极主动、灵活调动各种资源解决问题,而非被动、逃避的方式来解决问题。医学生五术素养水平越高,当面对与职业选择有关的应激源时,积极应对方式越多,个体会获得更多正向情绪和积极心境,认知水平随之提高,其职业决策自我效能感也会越强。

5.心理资本和积极应对方式在五术素养和职业决策自我效能感之间呈链式中介作用。研究发现,通过心理资本和积极应对方式的链式作用影响职业决策自我效能感,五术素养越高的个体,其心理资本水平越高,拥有良好的心理资本的医学生会更加倾向于采用积极的应对方式解决问题[20],医学生学习时间长且需要经过规培才能进入工作岗位,职业决策时考虑因素更为复杂,而拥有高心理资本和积极的应对方式可以帮助医学生掌握精湛医学理论技术的同时也能培养其高尚医德,人文情怀与仁爱之心,使医学生面对择业压力时会更有信心,更易于采用积极主动的归因模式看待问题,逆境中可以迅速地复原并主动改变应对策略来促进自身成长,进而提高其职业胜任的信心和预期,即提高职业决策自我效能感。

(二)对策建议

本研究基于医学生五术培养内涵和相关实证研究结果,探讨了医学生的五术素养和职业决策自我效能感之间的定量关系,通过构建定量五术素养量表模型,探究了医学生五术素养影响职业决策的关键影响因子及其定量作用,研究结果为该群体的职前指导提供了一定的实证支持,对于深化医学生的五术素养和职业决策自我效能感的关系研究具有积极的理论意义与实践意义。

本研究的创新性在于文化资源与心理机制的融合探索及医学教育领域的应用创新。本研究将五术素养引入医学生职业发展领域,填补了医学教育中综合素养对职业决策能力影响的理论空白,首次将医学五术纳入文化心理学分析框架,提炼为医学人文素养的核心维度,突破传统医学教育研究中偏重技术能力而忽视人文整合的局限,揭示其作为"文化心理资本"的深层作用,通过文化认同与心理资本协同机制影响职业决策的路径,构建"文化素养—心理资源—积极应对"的链式中介模型,将文化心理学与积极心理学深度融合,为五术文化在职业决策中的积极应对提供实证支持,提供"人文赋能心理、心理驱动行为"的新范式。同时本研究属于横断面研究,受研究方法选择的局限,只能得出变量之间的相关关系,需更多追踪研究验证因果关系,未来研究可进一步细化五术素养的测量工具,开展纵向追踪验证干预效果。

新时代下以具备"道术、仁术、学术、技术、艺术"的五术医者新内涵强化医学生培养为目标,职业决策自我效能感作为关键心理资源,高校应该有机融合多方资源提升医学生的五术素养、心理资本和积极应对方式。

一是以专业知识为载体,建立校内外全员育人、全程育人、全方位育人"三全育人"协同育人机制,注重知识传授、能力培养、价值塑造"三位一体";二是丰富教育教学课程体系内容,寻求医学生专业知识、课程思政教育、心理健康教育等完整结合的可行性,助力医学生完整精神与独立人格的成长,培养医学生"大医精诚"人文情怀,实现知识、能力与素养的有机融合;三是针对医学生提供更精细化的心理辅导实践路径以及职业规划和就业指导,建立全面的职业决策机制实践模式,提升医学生的职业决策自我效能感,实现五术医学人才培养目标,是本课题今后的重点研究方向,并开展"基于五术医学人才培养目标的心理健康教育课程思政实施路径研究""积极心理学视域下'家校医社'模式协同大学生心理健康教育效能体系的创新探究与实践"等相关课题探究。

参考文献:

[1]LI S, PAN Q, NIE Y. The impact of career-related parental behaviors on career decision-making self-efficacy and ambivalence: a latent growth model[J]. Journal of Adolescence, 2022, 94(7): 981-995.

(下转第40页)

中国知网 https://www.cnki.net

处,其中,本研究对风险决策倾向的评估没有结合风险程度进一步评估分析,对于风险决策倾向的调查工具需要优化。基于本研究结果,未来可以进一步探索分析,如不同类型的负性情绪的差异化影响,如从神经机制层面(前额叶皮层与边缘系统的互动)以验证三者之间的关系。

参考文献:

[1]ZHOU A B,LI Z K,XIE P,et al. The impact of information presentation on self-other risk decision-making[J]. Frontiers in Psychology,2024,15:1357644.

[2]冯忠良,伍新春,姚梅林,等.教育心理学[M].北京:人民教育出版社,2000.

[3]ATKINSON J W. Motivational determinants of risk-taking behavior[J]. Psychological Review,1957,64(6):359-372.

[4]ELLIOTT C S,ARCHIBALD R B . Subjective framing and attitudes towards risk[J]. Journal of Economic Psychology,1989,10(3):321-328.

[5]叶精华,莫丽蓉.大学生成就动机与职业决策自我效能感的关系:心理控制源的中介作用[J].心理学进展,2023,13(11):5061-5067.

[6]杨昭宁,禹钰,谭旭运.情绪对成就动机与风险决策关系的调节效应[J].应用心理学,2011,17(2):116-122.

[7]刘斌,姜金波,李逢战,等.负性情绪对军医大学学员风险决策及抑制控制影响的研究[J].联勤军事医学,2024,38(2):161-164,179.

[8]WATSON D,CLARK L A,TELLEGEN A . Development and validation of brief measures of positive and negative affect:the PANAS scales[J]. Journal of Personality and Social Psychology,1988,54(6):1063-1070.

[9]叶仁敏,HAGTVET K A.成就动机的测量与分析[J].心理发展与教育,1992(2):14-16.

[10]TVERSKY A,KAHNEMAN D . The framing of decisions and the psychology of choice[J]. Science,1981,211(4481):453-458.

[11]周文婧,宁宁,周一丹,等.突发事件情境下框架效应对哈尔滨市某高校医学生风险决策行为的影响[J].医学与社会,2022,35(4):23-26.

[12]陈浩然,郭伯堂,乔正学,等.成就动机在医学生职业使命感与就业焦虑间的中介作用[J].职业与健康,2022,38(16):2270-2274.

[13]李宵婷.认知闭合需要与成就动机对大学生风险决策的影响[D].兰州:西北师范大学,2020.

(编辑:陈越)

(上接第 35 页)

[2]GATI I,KULCSAR V. Making better career decisions:from challenges to opportunities[J]. Journal of Vocational Behavior,2021,126:103545.

[3]张书皓,杨语煊,吕少博,等.自我意识对职业决策困难的影响:生涯适应力与职业决策自我效能感的链式中介作用[J].中国健康心理学杂志,2022,30(8):1223-1227.

[4]胡洁.毕业生职业兴趣不一致与职业决策自我效能感的关系:应对方式和实习经历的调节作用[D].上海:华东师范大学,2023.

[5]刘玮玮,吴连宁,尹锡杨.民办高职贫困生未来时间洞察力、职业决策自我效能感与应对方式的关系研究[J].心理月刊,2023,18(13):57-59.

[6]王凯丽.大学生心理资本、职业决策自我效能感与职业生涯规划的关系研究[D].聊城:聊城大学,2019.

[7]詹启生,李秒.家庭亲密度对大学生职业决策自我效能感的影响:心理资本的中介作用[J]中国健康心理学杂志,2019,27(10):1585-1589.

[8]中华人民共和国国务院.国务院办公厅关于加快医学教育创新发展的指导意见[J].中华人民共和国国务院公报,2020(28):27-31.

[9]何碧如,郑节霞,章力,等."五术"融合的医学生《就业指导》课程探索与实践[J].温州医科大学学报,2022,52(11):938-942.

[10]沈慧,张宇辰,吴宏宇,等.医学生五术素养评定量表编制及信效度检验[J].长治医学院学报,2023,37(3):223-227.

[11]彭永新,龙立荣.大学生职业决策自我效能测评的研究[J].应用心理学,2001(2):38-43.

[12]张阔,张赛,董颖红.积极心理资本:测量及其与心理健康的关系[J].心理与行为研究,2010,8(1):58-64.

[13]解亚宁.简易应对方式量表信度和效度的初步研究[J].中国临床心理学杂志,1998(2):53-54.

[14]周浩,龙立荣.共同方法偏差的统计检验与控制方法[J].心理科学进展,2004(6):942-950.

[15]张轩辉.大学生心理资本与职业决策效能感、职业倾向的关系研究[D].广州:广州大学,2012.

[16]朱璐,刘晓,丁亚萍,等.医学生职业决策自我效能现状及其影响因素研究[J].中国健康心理学杂志,2012,20(12):1886-1889.

[17]张志学.家庭系统理论的发展与现状[J].心理学探新,1990(1):31-34,20.

[18]周紫婷.大学生心理资本与职业决策自我效能的关系[J].中国健康心理学杂志,2014,22(12):1819-1822.

[19]闫霞.高校毕业生职业决策自我效能感与应对方式、社会支持关系研究[D].长春:吉林大学,2011.

[20]郑亚楠,胡雯.某医学院校大一新生积极心理资本现况研究[J].中国预防医学杂志,2019,20(4):276.

(编辑:李鑫梅)

中国知网 https://www.cnki.net
