# Supplementary material for: A study on the current status and influencing factors of psychological capital among medical undergraduates
Source: Front Psychol. 2026 Oct 30;17:1830730. doi: 10.3389/fpsyg.2026.1830730 (PMC13759799; doi:10.3389/fpsyg.2026.1830730)
Supplement: Supplementary file 1 [file Data_Sheet_1.ZIP › References PDF/The Impact of Psychological Capital on the Subjective Well-being of Students at Higher Vocational Medical Colleges.pdf]

DOI:10.3969/j.issn.1008-4118.2022.03.029

心理资本对医学高职院校学生主观幸福感的影响

张慧珺，刘 旬，袁 珍
（菏泽医学专科学校，山东 菏泽 274000）

关键词：心理资本；主观幸福感；高职院校；医学生
中图分类号： B84 **文献标识码：**A **文章编号：**1008-4118（2022）03-0091-03

医学高职院校是为祖国输送医疗卫生人才的重要基地，而研究医学高职院校学生的心理状态不仅关乎学生自身的成长与发展，也关乎到整个医疗行业乃至整个国家和民族的命运，具有重要的社会意义。

主观幸福感作为重要的心理状态指标是指个体根据自己的判断对本人生活质量的整体性评估[1]。国内外关于影响主观幸福感的因素很多，其中心理资本是影响主观幸福感的重要指标之一。Luthans 等人认为心理资本是个体在成长和发展过程中表现出来的一种积极心理发展状态，包括效能感、希望、乐观和坚韧性 4 个维度[2]。梳理文章发现，心理资本对大学生、硕士研究生、少数民族预科生的主观幸福感均具有正向预测作用[3-5]；虽然这些研究都表明心理资本对主观幸福感有积极影响，但仍然存在以下问题有待探讨：对于医学院校学生，尤其是医学高职院校学生这一特殊群体来说，心理资本是如何影响主观幸福感的。查阅文献表明，医学高职院校学生心理资本与主观幸福感之间的关系研究较少，为丰富已有研究成果，弥补数量的不足，特开展此研究。

1 资料与方法

1.1 一般资料 2021 年 9 月—2022 年 1 月从山东省某医学专科学校随机抽取被试者进行问卷调查，得到有效问卷 604 份。本次被试者年龄 18~21 岁；大一年级 263 人（43.54%），大二年级 206 人（34.11%），大三年级 135 人（22.35%）；男生 151 人（25.00%），女生 453 人（75.00%）；独生子女 107 人（17.72%），非独生子女 497 人（82.28%）；家庭所在地：城镇 235 人（38.91%），农村 369 人（61.09%）。

1.2 观察指标 （1）积极心理资本量表（PPQ）：采用张阔等[6]（2008）编制的积极心理资本问卷，问卷共 26 个项目，包括 4 个维度，分别为自我效能、韧性、希望、乐观。每一个题按照七点量表进行评分，1 分表示完全不符合，7 分表示完全符合，问卷采用正向计分，得分越高积极心理资本越强。量表的克隆巴赫 α 系数均大于 0.7，整体的内部一致性系数为 0.85，信度、效度良好。（2）总体幸福感量表（GWB）[7]：该量表是由美国国立卫生统计中心制定的，1996 年段艳华对该量表进行了修订，修订后的量表共有 18 个题目，共包括 6 个维度：对健康的担心、精力、对生活的满足和兴趣、忧郁或愉快的心境、对情感和行为的控制以及松弛与紧张（焦虑）。量表总分越高表示主观幸福感水平越强烈。量表各项目与总分相关在 0.48~0.78 之间，内部一致性系数为 0.91，信度、效度良好。

1.3 统计学处理 应用 SPSS22.0 进行数据分析，从问卷星导出数据后进行整理，分别做了描述性统计分析，独立样本 *t* 检验，皮尔逊相关分析，回归分析。

2 结果

2.1 医学高职院校学生心理资本和主观幸福感的情况分析 心理资本平均总分（4.87 ± 0.65）分，自我效能、韧性、希望、乐观四个维度平均得分分别为（4.92 ± 1.01）分、（4.08 ± 0.61）分、（5.14 ± 0.81）分、（5.48 ± 1.03）分。主观幸福感平均总分（4.71 ± 0.73）分，对健康的担心、精力、对生活的满足和兴趣、忧郁或愉快的心境、对情感和行为的控制、松弛与紧张六个维度平均得分分别为（4.19 ± 1.27）分、（4.93 ± 1.08）分、（5.03 ± 1.39）分、（5.69 ± 1.06）分、（4.48 ± 0.82）分、（4.69 ± 0.83）分、

(C)1994-2022 China Academic Journal Electronic Publishing House. All rights reserved. http://www.cnki.net

（4.71±0.73）分。心理资本总分和各维度得分以及主观幸福感总分和各维度得分均高于平均分数。经过统计处理发现，性别、独生子女和家庭所在地人口学变量对于主观幸福感总分以及各维度的差异无统计学意义。性别和独生子女对于心理资本总分差异无统计学意义，生活所在地虽然对于心理资本总分差异无统计学意义，但是对其韧性维度差异有统计学意义。见表1。

2.2 心理资本低分组和心理资本高分组的主观幸福感分析 按照27%的占比进行高低分组，即将心理资本总分从低到高进行排序，取得分在前27%（n=163）作为低分组，得分在后27%（n=163）作为高分组。对低分组和高分组的主观幸福感总分和各维度分进行独立样本t检验，结果发现在对情感和行为的控制这一维度中，低分组显著低于高分组（$P<0.01$）。

2.3 医学高职院校学生心理资本和主观幸福感的相关分析 将心理资本总分及各维度和主观幸福感总分及各维度进行相关性分析，结果发现，对健康的担心和心理资本总分及各维度呈负相关，其中除韧性外，其余均达到显著水平。从心理资本的韧性维度看，韧性和主观幸福感总分及各维度呈负相关；除对健康的担心这一维度外，其余均达到显著水平。其余维度及总分都呈显著正相关。见表2。

2.4 医学高职院校学生心理资本和主观幸福感的回归分析 为研究心理资本对主观幸福感的预测作用，采用回归分析，以主观幸福感总分为因变量，以心理资本总分以及各维度为自变量，结果发现，自我效能没有进入统计方程，这表明自我效能对主观幸福感总分无预测作用。韧性、希望、乐观以及心理资本总分可以作为自变量正向预测主观幸福感，且达到统计学显著水平（$P<0.01$）。结果见表3。由R2可知，预测变量对主观幸福感总分的解释量为53%。

表1 心理资本维度（韧性）的人口统计学差异（M±SD）

项目		韧性	t	P
家庭所在地	农村（n=369）	4.03±0.59	2.696	<0.01
	城镇（n=235）	4.17±0.67		

表2 心理资本总分及各维度与主观幸福感总分及各维度的相关性（r）

	自我效能	韧性	希望	乐观	心理资本总分
对健康的担心	–0.173	–0.750	–0.191	–0.168	–0.207
精力	0.660	–0.222	0.562	0.655	0.620
对生活的满足和兴趣	0.578	–0.200	0.485	0.606	0.552
忧郁或愉快的心境	0.659	–0.215	0.554	0.686	0.630
对情感和行为的控制	0.532	–0.164	0.479	0.522	0.509
松弛与紧张	0.456	–0.263	0.349	0.460	0.393
主观幸福感总分	0.650	–0.269	0.537	0.659	0.599

表3 心理资本各维度对主观幸福感的逐步回归

因变量	自变量	β	R2	F	P
主观幸福感总分	常量	3.243	0.535	172.413	0.000
	韧性	–0.538			0.000
	希望	–0.354			0.000
	乐观	0.064			0.247
	心理资本总分	1.054			0.000

3 讨论

3.1 主观幸福感的现状分析 研究发现，主观幸福感总分较为理想，整体上处于中等偏上水平，这和前人的研究一致[3]。其中，对健康的担心维度在各个维度中得分

(C)1994-2022 China Academic Journal Electronic Publishing House. All rights reserved. http://www.cnki.net

最低，说明大学生对健康的担心重视程度有待加强。此外，主观幸福感总分及各维度在性别的差异不显著，这可能是因为，一方面从社会认同的角度来说，重男轻女这一观念不断淡化，社会对男女生的要求趋于一致，所以男女生的主观幸福感水平无差异；另一方面，从性别认同的角度来说，当理想的性别类型和生理性别类型相一致的时候，人类对环境具有良好的适应性，且具有较好的心理健康水平，主观幸福感也会较好，这也符合一致性模型 [8]。

按照27%的占比进行高低分组，结果发现在对情感和行为的控制这一维度，心理资本低分组显著低于高分组，符合合理认知，由此可见，医学高职院校学生心理资本越强，其主观幸福感或维度的趋势越强，心理资本是影响其主观幸福感的因素。

3.2 *心理资本与主观幸福感的相关性* 关于心理资本和主观幸福感的关系，从相关分析中可以看出，对健康的担心和心理资本总分及各维度呈负相关，其中除韧性分子外，其余均达到显著水平。这说明对健康的担心越低，心理资本以及各维度都会越高，这符合常人的合理认知，如果对健康的担心达到不合理水平将会导致疑病症的发生，从而影响心理资本。

从心理资本的韧性维度看，韧性和主观幸福感总分及各维度呈负相关，这说明，心理韧性改变时主观幸福感及各维度会向相反的方向发生变化，这和以往的研究不同 [9]，有研究认为，压力会调节心理韧性对主观幸福感的影响 [10]，这说明压力较大的情况下，可能会影响研究结果，在本研究问卷收集期间，恰逢考试周，学生普遍压力较大，因此在今后的研究中，将尽可能的排除其他因素所带来的的影响，或者将压力作为调节变量进一步研究。此外，心理资本总分和主观幸福感总分呈正相关，这和前人研究一致 [3-5]。

3.3 *心理资本对主观幸福感的预测作用* 逐步回归分析发现，对高职医学院校学生来说，自我效能不能起预测作用。我们选取具代表性的回归模型，即希望、韧性、乐观、心理资本总分作为预测变量共同对主观幸福感起正向预测作用，这和前人研究相同 [3-5]，符合主效应模型（main effect model），主效应模型认为，个体的心理资本会对个体的行为产生直接影响，会提高主观幸福感、工作满意度，降低旷工行为、旷课行为等 [2]。

综上所述，提高医学高职院校学生的心理资本有助于提高学生主观幸福感，而具备较强心理韧性、乐观积极向上品格等是心理资本提升的重要因素。对此我们建议：（1）培养学生应对挫折的能力，既能增强学生心理韧性也能帮助他们在逆境中成长。（2）提高学生情绪调节的能力，使学生做情绪的舵手，培养其乐观积极向上的品格。（3）增强学生自信水平，提高自信心有助于增强学生的自我效能感，使其悦纳自我、完善自我。（4）唤醒学生学习潜力，加强学生学习管理，丰富学生的创造性思维，使其对未来充满希望。（5）加强学生思想政治教育，总体上提高其心理健康水平。

参考文献：

[1] Rikke A.A. Jensen,Dorthe Kirkegaard Thomsen,Maja O'Connor,et al.Age differences in life stories and neuroticism mediate age differences in subjective well - being[J].Appl Cogn Psychol, 2020, 34(1):3-15.

[2] Luthans Fred,Broad Julie Dyrdek.Positive psychological capital to help combat the mental health fallout from the pandemic and vuca environment[J].Organ Dyn, 2020(prepublish):100817.

[3] 陈莹，雷祺，高明杰．社交媒体使用对大学生主观幸福感的影响—本土心理资本的视角 [J]. 现代教育科学，2021(5):120-127.

[4] 张鲜华，周佳佳，冯雪．导师支持对硕士研究生主观幸福感的影响：心理资本中介及家人支持调节 [J]. 成都师范学院学报，2022, 38(2):34-43.

[5] 叶宝娟，方小婷，廖雅琼，等．心理资本对少数民族预科生主观幸福感的影响：有调节的中介效应 [J]. 中国临床心理学杂志，2017, 25(5):967-969, 966.

[6] 张阔，张赛，董颖红．积极心理资本：测量及其与心理健康的关系 [J]. 心理与行为研究，2010, 8(1):58-64.

[7] 段建华．总体幸福感量表在我国大学生中的试用结果与分析 [J]. 中国临床心理学杂志，1996(1):56-57.

[8] 陈少青，王芳，王美芳．青少年性别角色与体质健康的关系：体育行为的中介作用及性别差异 [J]. 体育学刊，2021, 28(5):94-99.

[9] 花军，张东．高职生心理资本在学业压力与主观幸福感之间的中介效应 [J]. 扬州教育学院学报，2021, 39(3):73-76.

[10] 王田，刘启蒙，罗海风，等．高中生学习动机、学习压力与主观幸福感的阈值研究——以我国东部S省的测评结果为例 [J]. 华东师范大学学报（教育科学版），2021, 39(3):59-70.

收稿日期：2022-02-09

执行编委：邵文锦
责任编辑：邵文锦 付甜甜
英文校对：付甜甜
责任校对：邵文锦 付甜甜

(C)1994-2022 China Academic Journal Electronic Publishing House. All rights reserved. http://www.cnki.net
