# Supplementary material for: A study on the current status and influencing factors of psychological capital among medical undergraduates
Source: Front Psychol. 2026 Oct 30;17:1830730. doi: 10.3389/fpsyg.2026.1830730 (PMC13759799; doi:10.3389/fpsyg.2026.1830730)
Supplement: Supplementary file 1 [file Data_Sheet_1.ZIP › References PDF/The Mediating Role of Psychological Capital and Self-Efficacy in Career Decision-Making on the Professional Identity of Clinical Medical Students at a Certain Medical School.pdf]

某医学院临床医学生职业认同在心理资本与职业决策自我效能感中的中介作用

王　洁[1]　曾劲超[2]　李淼晶[3]　李保国[1]

1.成都医学院马克思主义学院，四川成都 610500；2.绵阳开放大学职教处，四川绵阳 621000；3.成都医学院大健康与智能工程学院，四川成都 610500

[摘要] 目的 探究医学生职业认同在临床医学专业学生心理资本和职业决策自我效能感关系中的中介作用。方法 以成都医学院 2023—2024 学年在校临床医学专业学生为研究对象，采用分层抽样的方法进行抽样，使用医学生职业认同量表、医学生心理资本问卷和大学生职业决策自我效能感问卷进行调查，对数据进行描述性分析、差异性分析和中介效应分析。结果 共发放问卷 1 500 份，有效问卷共 1 361 份，问卷有效回收率为 90.7%。医学生职业认同量表得分为（3.59±0.57）分，医学生的心理资本量表得分为（3.55±0.54）分，医学生职业决策自我效能感量表得分为（3.56±0.69）分；职业决策自我效能感与心理资本和医学生职业认同呈正相关（r=0.82、0.73，P＜0.05）；医学生职业认同在临床医学专业学生心理资本和职业决策自我效能感之间存在部分中介作用，心理资本对职业决策自我效能感的直接效应和间接效应分别为 0.810 和 0.241。结论 心理资本水平越高，职业决策自我效能感越高，良好的心理资本水平可以促进医学生职业认同，从而对职业决策自我效能感产生正向影响。

[关键词] 医学生；职业认同；心理资本；职业决策自我效能感

[中图分类号] R395.6　[文献标识码] A　[文章编号] 1673-7210(2025)07(c)-0076-06

DOI：10.20047/j.issn1673-7210.2025.21.13

The mediating role of professional identity in psychological capital and professional decision-making self-efficacy of clinical medical students in a medical college

WANG Jie[1]　ZENG Jinchao[2]　LI Miaojing[3]　LI Baoguo[1]

1.School of Marxism, Chengdu Medical College, Sichuan Province, Chengdu 610500, China; 2.Department of Vocational Education, Mianyang Open University, Sichuan Province, Mianyang 621000, China; 3.School of Great Health and Intelligent Engineering, Chengdu Medical College, Sichuan Province, Chengdu 610500, China

[Abstract] **Objective** To explore the mediating role of medical student professional identity in the relationship between psychological capital and psychological decision-making self-efficacy among clinical medical students. **Methods** The clinical medical students enrolled in Chengdu Medical College during the 2023 to 2024 academic year were used as the research subjects, and the stratified sampling method was used for sampling, the medical student professional identity scale, the questionnaire psychological capital scale of medical students, and the questionnaire on college students' self-efficacy in psychological decision-making was used for the survey, and the data were subjected to descriptive analysis, analysis of variance, and analysis of mediating effects. **Results** A total of 1 500 questionnaires were distributed, with a total of 1 361 valid questionnaires, and the effective recovery rate of the questionnaires was 90.7%. The score of medical student professional identity scale was (3.59±0.57) points, the questionnaire psychological capital scale was (3.55±0.54) points, and the questionnaire on college students' decision-making self-efficacy in career was (3.56±0.69) points; psychological decision-making self-efficacy had a significant positive correlation with psychological capital and medical students' career identity (r=0.82, 0.73, P<0.05); medical students' career identity was partially mediated between psychological capital and psychological decision-making self-efficacy of clinical medical students; the direct and indirect effects of psychological capital on career psychological decision-making self-efficacy were 0.810 and 0.241, respectively. **Conclusion** The higher the level of psychological capital, the higher the sense of self-efficacy in psychological decision-making, and a good level of psychological capital can promote medical student' career identity, thus positively affecting the sense of self-efficacy in psychological decision-making.

[Key words] Medical students; Professional identity; Psychological capital; Psychological decision-making self-efficacy

[基金项目] 成都医学院教育教学改革重点项目（JG202107）；成都医学院教育教学改革重点（研究生）项目（YJG202201）。

[作者简介] 王洁（2000.9-），女，成都医学院马克思主义学院 2024 级思想政治教育专业在读硕士研究生；研究方向：思想政治教育、医学人文。

[通讯作者] 李保国（1975.10-），男，博士，教授；研究方向：马克思主义理论。

中国知网 https://www.cnki.net

临床医学类专业学生是未来医学界的主力军，提高其心理素质和职业认同感尤为重要。职业认同是人们努力达成组织目标的心理基础，它涉及个人对职业方面的看法和认识，以及对他人或群体的认可和赞同[1]。心理资本是指个体在成长和发展过程中表现出的积极心理状态，包含自我效能、乐观、韧性和希望 4 个核心部分[2]。职业决策自我效能是指在职业决策过程中，个人对自己完成各项任务的能力进行自我评估和自信心的表现[3]。

研究发现，心理资本水平越高，个体的职业决策自我效能感越强[4]；心理资本也能有效增强职业认同感[5]。张敏等[6]和徐慧等[7]研究还发现，职业认同与职业决策自我效能感呈显著正相关。上述研究表明，职业认同、心理资本与职业决策自我效能感之间有紧密的关系。但是，目前关于职业认同、心理资本与职业决策自我效能感三者关系的研究较少，且主要研究对象为护理学专业学生及护士，未见以临床医学生为对象开展的研究。此外，医学生职业认同在心理资本和职业决策自我效能感之间可能存在中介效应，有待进一步开展研究。基于此，本研究旨在研究医学生职业认同现状，探讨医学生职业认同、心理资本和职业决策自我效能感三者之间关系，为医学院校临床医学专业提供职业教育的科学依据。

1 对象与方法

1.1 研究对象

本研究以成都医学院 2023—2024 学年在校临床医学专业学生为研究对象。纳入标准：①2023—2024学年就读于成都医学院临床医学专业的在校本科生；②能够理解问卷内容并自愿参加本研究；③调查过程中回答完整、无明显缺失值。排除标准：①非临床医学专业学生或已离校人员；②在填写问卷过程中规律明显或异常者（如全选同一选项）；③未能完成全部问卷项目，存在大量缺失值；④调查过程中表示不愿继续参与。本研究经成都医学院伦理委员会批准（CMCIR2025NO.052）。

1.2 研究方法

1.2.1 调查工具

1.2.1.1 一般资料调查表　该调查表由研究者自行设计，包括性别、年级、是否担任学生干部 3 个条目。

1.2.1.2 医学生职业认同量表　医学生职业认同量表采用张丽莉[8]编制的《医学生职业认同量表》，由职业认知、职业情感、职业承诺、职业行为、职业期望、职业价值观所构成。共 38 个条目，采用 Likert 5 级计分法，总分为 38～190 分。得分与医学生职业认同呈正相关，即个体得分越高，医学生职业认同度越高。该量表的 Cronbach's α 系数为 0.83。

1.2.1.3 医学生心理资本问卷　心理资本量表采用张阔等[9]（2010）在 PCQ-24 的基础上编制的《心理资本问卷（PPQ）》，共 26 个条目，其中，自我效能和韧性因子各包括 7 个项目，乐观和希望因子各包括 6 个项目。采用 Likert 5 级计分法，总分为 26～130 分。得分与医学生心理状态呈正相关，即个体得分越高，医学生心理状态越高。该问卷的 Cronbach's α 系数为 0.86。

1.2.1.4 大学生职业决策自我效能感问卷　职业决策自我效能感量表采用《大学生职业决策自我效能感问卷》，共 39 个项目，包括自我评价、收集信息、选择目标、制定规划、问题解决 5 个方面[10]。采用 Likert 5 级计分法，总分为 39～195 分。得分与医学生职业决策自我效能呈正相关，即个体得分越高，医学生自我决策能力越高。该问卷的 Cronbach's α 系数为 0.95。

各量表总分以总体均分计入，总体均分 = 总分 / 条目数。

1.2.2 调查方法

本研究采用分层抽样的方式，从成都医学院 2023—2024 学年临床专业的 5 个年级中分别抽取样本。因部分年级学生课业任务繁重或临床实习分布不均，导致样本数量存在一定差异。共发放问卷 1 500份，有效问卷共计 1 361 份，问卷有效回收率为 90.7%。

1.2.3 质量控制措施

本研究采用已有成熟量表进行测量。在正式调查前进行了小范围预测试，及时发现并修正了问卷中存在的逻辑问题与设置错误。为控制问卷的回收效率与质量，设置了每个 IP 和设备仅可提交一次的限制，并剔除填写时间少于 1 min 的问卷。在数据录入阶段，研究人员对异常值及量表得分范围进行了核查，剔除逻辑不一致或数据异常的问卷，确保数据的真实性与可靠性。

1.3 统计学方法

采用 SPSS 21.0 统计学软件进行数据分析，符合正态分布的计量资料采用均数±标准差（$\bar{x}\pm s$）表示，比较采用 t 检验和单因素方差分析进行组间比较，计数资料以例数和百分率进行表示，比较采用 χ^2 检验。相关性采用 Pearson 相关性分析，用相关系数 r 表示关联强度，运用 Bootstrap 抽样法重复抽样 5 000 次，构建间接效应置信区间，若置信区间不包含 0，认为中介效应显著进行中介效应检验。以 P<0.05 差异有统计学意义。

2 结果

2.1 调查对象人口学特征

本研究以成都医学院 2023—2024 学年在校临床医学专业学生为研究对象，共纳入 1 361 名调查对象，其中男 485 名（35.64%），女 876 名（64.36%）。见表 1。

中国知网 https://www.cnki.net

表 1　1 361 名学生一般情况

项目	人数	百分比（%）
性别		
男	485	35.64
女	876	64.36
年级		
大一	283	20.79
大二	284	20.87
大三	282	20.72
大四	248	18.22
大五	264	19.40
担任学生干部		
是	468	34.39
否	893	65.61
合计	1 361	100.00

2.2 医学生职业认同、心理资本与职业决策自我效能感状况

医学生职业认同得分为（3.59±0.57）分，心理资本得分为（3.55±0.54）分，职业决策自我效能感得分为（3.56±0.69）分。在性别比较中，男性医学生职业认同、心理资本与职业决策自我效能感得分均高于女性，差异有统计学意义（$P<0.05$）。在年级比较中，心理资本得分比较，差异无统计学意义（$P>0.05$）；医学生职业认同和职业决策自我效能感得分比较，差异有统计学意义（$P<0.01$），均为大一得分最高。担任学生干部的医学生职业认同、心理资本与职业决策自我效能感得分均高于未担任学生干部的学生，差异有统计学意义（$P<0.01$）。见表 2。

表 2　医学院医学生职业认同、心理资本与决策自我效能感评分比较（分，$\bar{x}\pm s$）

项目	人数	医学生职业认同	心理资本	职业决策自我效能感
性别				
男	485	3.64±0.64	3.60±0.61	3.65±0.69
女	876	3.56±0.53	3.53±0.50	3.51±0.69
t 值		2.41	2.04	3.35
P 值		0.020	0.040	<0.001
年级				
大一	283	3.71±0.68	3.62±0.69	3.72±0.79
大二	284	3.53±0.59	3.51±0.57	3.47±0.68
大三	282	3.65±0.52	3.59±0.51	3.56±0.70
大四	248	3.52±0.47	3.52±0.44	3.49±0.60
大五	264	3.51±0.54	3.53±0.43	3.55±0.65
F 值		6.82	1.79	5.54
P 值		<0.001	0.130	<0.001
担任学生干部				
是	468	3.67±0.57	3.63±0.55	3.67±0.67
否	893	3.54±0.57	3.51±0.53	3.50±0.70
t 值		3.83	3.67	4.30
P 值		<0.001	<0.001	<0.001

2.3 医学生职业认同、心理资本与职业决策自我效能感的相关性分析

医学生职业认同各维度与心理资本各维度呈正相关性（r=0.47～0.75，$P<0.01$），与职业决策自我效能感各维度呈正相关（r=0.50～0.73，$P<0.01$）。见表 3～4。心理资本各维度与职业决策自我效能感各维度呈正相关（r=0.60～0.82，$P<0.01$）。见表 5。

表 3　医学生职业认同与心理资本的相关性（r 值）

项目	心理资本	自我效能	韧性	希望	乐观
医学生职业认同	0.75[a]	0.69[a]	0.61[a]	0.72[a]	0.68[a]
职业认知	0.59[a]	0.54[a]	0.53[a]	0.54[a]	0.50[a]
职业情感	0.61[a]	0.56[a]	0.47[a]	0.59[a]	0.56[a]
职业承诺	0.61[a]	0.55[a]	0.52[a]	0.58[a]	0.55[a]
职业行为	0.71[a]	0.67[a]	0.55[a]	0.70[a]	0.64[a]
职业期望	0.69[a]	0.65[a]	0.51[a]	0.66[a]	0.64[a]
职业价值	0.67[a]	0.59[a]	0.55[a]	0.65[a]	0.61[a]

注　[a]$P<0.01$。

表 4　医学生职业认同与职业决策自我效能感的相关性（r 值）

项目	职业决策自我效能感	自我评价	收集信息	选择目标	制定规划	问题解决
医学生职业认同	0.73[a]	0.71[a]	0.71[a]	0.71[a]	0.72[a]	0.69[a]
职业认知	0.53[a]	0.53[a]	0.52[a]	0.52[a]	0.52[a]	0.50[a]
职业情感	0.59[a]	0.58[a]	0.57[a]	0.57[a]	0.59[a]	0.56[a]
职业承诺	0.57[a]	0.56[a]	0.55[a]	0.57[a]	0.56[a]	0.54[a]
职业行为	0.70[a]	0.68[a]	0.69[a]	0.68[a]	0.70[a]	0.67[a]
职业期望	0.70[a]	0.69[a]	0.68[a]	0.69[a]	0.69[a]	0.68[a]
职业价值	0.63[a]	0.62[a]	0.61[a]	0.62[a]	0.62[a]	0.61[a]

注　[a]$P<0.01$。

表 5　心理资本与职业决策自我效能感的相关性（r 值）

项目	心理资本	自我效能	韧性	希望	乐观
职业决策自我效能感	0.82[a]	0.76[a]	0.62[a]	0.80[a]	0.76[a]
自我评价	0.81[a]	0.74[a]	0.61[a]	0.79[a]	0.75[a]
收集信息	0.80[a]	0.74[a]	0.60[a]	0.78[a]	0.73[a]
选择目标	0.80[a]	0.74[a]	0.61[a]	0.78[a]	0.75[a]
制定规划	0.80[a]	0.74[a]	0.60[a]	0.79[a]	0.74[a]
问题解决	0.79[a]	0.74[a]	0.61[a]	0.76[a]	0.72[a]

注　[a]$P<0.01$。

中国知网 https://www.cnki.net

2.4 医学生职业认同在心理资本、职业决策自我效能感的中介效应

自变量和因变量显著相关是检验中介效应的前提，从相关分析结果显示，本研究满足进行中介效应检验的条件。本研究以心理资本为自变量 X，职业决策自我效能感为因变量 Y，医学生职业认同为中介变量 M，运用 Bootstrap 检验法，重复抽样 5 000 次进行中介效应检验，获得 3 条回归路径模型，如图 1。对于模型 1，心理资本对职业决策自我效能感的总效应显著（R^2=0.672，F=2 785.258，P<0.01）。模型 2，心理资本对医学生职业认同具有显著正向预测作用（R^2=0.569，F=1 793.011，P<0.01）。模型 3 引入中介变量职业认同后，心理资本对职业决策自我效能感的直接效应仍然显著，整体模型拟合略有提升（R^2=0.699，F=1 576.243，P<0.01）。见表 6。

由表 7、图 1 可知，心理资本通过医学生职业认同，对职业决策自我效能感产生显著的间接影响，效应值为 0.241，占总效应的 22.93%，其 95%CI（0.186，0.300）不包含 0；同时，直接效应值为 0.810，占总效应的 77.07%，95%CI（0.753，0.867）亦不包含 0，显示直接路径同样显著。

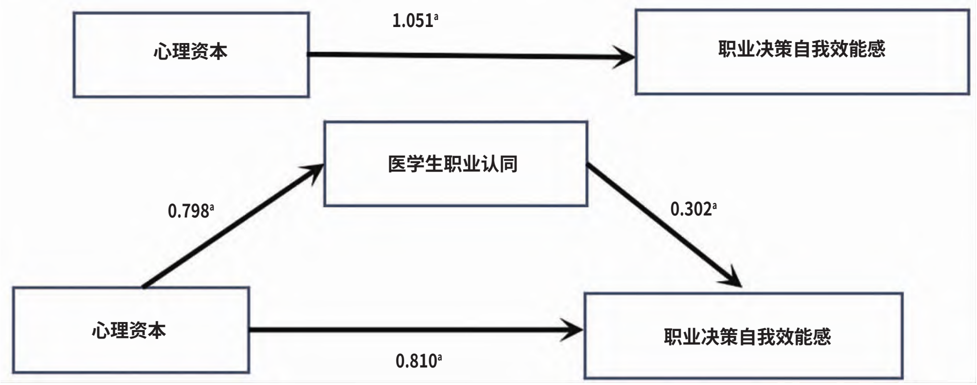

aP<0.01。

图 1 医学生职业认同在心理资本和职业决策自我效能感间的中介效应模型

表 6 中介效应模型检验

项目	职业决策自我效能感（模型 1）	医学生职业认同（模型 2）	职业决策自我效能感（模型 3）
心理资本	1.051a	0.798a	0.810a
医学生职业认同			0.302a
R 值	0.820	0.754	0.836
R^2 值	0.672	0.569	0.699
F 值	2 785.258a	1 793.011a	1 576.243a

注 aP<0.01。

3 讨论

3.1 医学生职业认同感处于较高水平，但高年级职业认同感仍需注意

本研究中，医学生职业认同得分处于中等偏上水平，与学者刘智新等[11]研究结果一致，显示本研究中医学生职业认同状况较为良好，但在不同年级间表现出显著差异。大一医学生职业认同感最高，大五阶段最低，呈现出“先降后升再降”的趋势，与学者张丽莉[8]的研究结果相似。这一趋势可能与医学生在不同阶段的职业期待、学业压力及临床实践体验相关。学生职业认同的建构始于专业学习阶段，并持续贯穿其职业生涯全程，是一个不断变化发展的过程[12]。大一新生初学医学专业，通常对医学领域充满理想化认知，认为医学职业崇高且富有社会价值。因此，这一阶段表现出较高的职业认同感。然而，随着课程难度增加，特别是在基础医学阶段，学生可能因高强度的学习任务、考试压力和临床技能欠缺而对自身能力产生怀疑，现实与理想之间的落差可能导致职业认同感下降[13]。进入临床学习阶段后，医学生有更多的机会接触真实的医疗环境，并通过参与临床实践增强职业自信，因此，医学生在大四阶段的职业认同感有一定回升。然而，到大五阶段，医学生面临着角色转变的挑战，从理论学习和临床观察过渡到真正的医患互动，承担着更大的社会与工作责任，同时由于经验不足和技术不成

表 7 1 361 名医学生职业认同在心理资本与职业决策自我效能感间的中介效应模型路径系数

模型效应	效应值	Bootstrap 95%CI		Boot S.E.	效应占比（%）
		下限	上限		
总效应	1.051	1.012	1.090	0.020	
直接效应（心理资本→职业决策自我效能感）	0.810	0.753	0.867	0.029	77.07
中介效应（心理资本→医学生职业认同→职业决策自我效能感）	0.241	0.186	0.300	0.029	22.93

中国知网 https://www.cnki.net

熟,面临着患者排斥等现实压力[14]。此外,医学职业相较于其他专业,面临着职业发展路径更长、收入起点相对较低等现实因素,使得部分医学生会重新审视自己的职业,对未来发展产生不确定性,进而使职业认同感在这一阶段降至最低。这提示,医学院校可以从课程设置、临床实践、职业规划指导等多个方面入手[1]。在基础医学阶段,可通过医学人文课程、职业道德教育、红医精神教育等,强化医学生对医学职业的认同感,促使其建立长期职业目标[15]。针对大五阶段的职业迷茫期,可以开展规培政策解读、专科方向选择、就业市场分析等,帮助医学生明确职业发展方向,设立积极的职业发展目标,增强医学生对未来的可控感。

3.2 良好的心理资本与较强的职业认同，可以提高医学生的职业决策自我效能感

本研究结果显示,医学生心理资本与职业决策自我效能感呈正相关($P<0.01$),与学者曹虹等[16]的研究结果一致。具体来看,心理资本的 4 个核心维度均与职业决策自我效能感密切相关,尤其是希望、自我效能、乐观对职业决策自我效能感的影响最显著。医学生对未来的乐观预期及对自身能力的信心,在其职业决策过程中发挥着关键作用。计划行为理论认为,个体的职业决策受态度、主观规范和感知行为控制的影响[17]。希望感能够塑造医学生对未来职业发展的积极态度,促使其设定明确的职业目标,并增强坚持实现目标的意愿,从而减少职业决策过程中的不确定性与焦虑感,提升其职业决策自我效能感。此外,希望感还能够提升医学生的感知行为控制,使其在面对复杂职业选择时更加自信。这提示在医学教育过程中,提供及时的鼓励和积极反馈，营造支持性的学习范围,减少过度竞争带来的焦虑感。同时,建立实习生心理支持系统,设立心理咨询热线、心理辅导小组等,增强医学生的心理韧性与希望感,以此提升医学生的职业决策自我效能感。

此外,医学生职业认同对职业决策自我效能感也呈正相关($P<0.01$),与学者张敏等[6]和徐慧等[7]的研究结果一致。在职业认同中,职业期望维度和职业行为维度的相关系数较高。医学生对未来职业的期望和实际行为,对提升其职业决策自我效能感的提升具有显著作用。医学生的职业认同感是影响职业决策自我效能感的重要因素[18]。社会认同理论认为,个体的职业选择和决策是基于对自己社会角色的认同和接纳程度[19]。当医学生对医学职业有强烈的认同感和归属感时,他们更倾向于将自己归属于医学群体,从而增强职业归属感和职业承诺,从而提高其职业决策的稳定性和自信心。相反,缺乏职业认同感和归属感的医学生可能会感到迷茫、焦虑和无力,从而降低职业决策的自我效能感。职业行为维度反映个体在实际行动中对职业角色的践行程度,例如参与实践、科研和临床实践的主动性。这些行为不仅能够强化医学生的职业认同,还能够通过实践增强职业决策自我效能感[20]。因此,当医学生对医学职业形成高度认同感和归属感时，他们更容易在职业决策过程中保持信念与自信。这提示,医学院校可以采取优化临床实践体验、邀请资深医师和优秀校友分享职业发展经验等,强化医学生的职业认同,间接增强其职业决策自我效能感。

3.3 医学生职业认同在心理资本与职业决策自我效能感之间存在部分中介作用

中介效应分析结果显示,医学生职业认同在心理资本和职业决策自我效能感之间存在部分中介作用,直接效应值为 0.810,间接效应值为 0.241,占总效应的 22.93%，提示心理资本可以通过职业认同的中介作用,间接影响医学生职业决策自我效能感。一方面,自我决定理论认为,人类天生具有内在的自主性和自我决定性动机。当个体感到自己的行为是自主的,而不是受到外界压力或强制的，会感到满足和快乐[21]。作为心理资本核心维度之一的自我效能感,即指个体完成特定任务的能力和自信心,能够使医学生在面临挑战时更倾向于采取积极的应对策略[22]。当医学生的职业身份与自己价值观和人生目标相符,如能够胜任医学学习任务、临床技能训练及未来职业要求时,他们更容易将医学职业视为个人的理想路径,并形成较强的职业认同感。此外,心理资本中希望感和韧性同样发挥重要作用。这些心理资本要素的共同作用,使得医学生能够在面对现实挑战时,依然保持较高的职业认同感,并在职业决策中表现出更强的信心和自主性。另一方面,结合本研究,心理资本得分为(3.55±0.54),职业认同得分为(3.59±0.57)分,职业决策自我效能感得分为(3.56±0.69)分,整体水平良好,与学者郑亚楠等[23]和刘洋等[24]的结果一致。

医学高校的教育环境和心理支持措施在一定程度上促进了医学生的积极心理状态,增强了其对未来职业选择的信心。研究进一步发现,担任学生干部的医学生在职业认同、心理资本与职业决策自我效能感方面,得分均显著高于未担任学生干部的学生。这可能是由于学生干部在参与管理、组织及社交互动的过程中,提升了人际交往、组织协调和问题解决的能力,积累了更高水平的心理资本,从而更容易形成对医学职业的高度认同感,并在职业决策中表现出更强的信心与主动性[25]。心理资本与职业决策自我效能感在不同年级间呈现出一致的变化趋势。这显示随着学业和

中国知网 https://www.cnki.net

职业现实压力的逐渐加深，心理资本的降低可能导致职业认同感下降，从而又进一步削弱职业决策自我效能感。因此，医学院校应采取针对性的干预措施，加强心理资本建设与职业认同教育，以确保医学生在未来职业发展过程中保持较高的职业决策自信心和稳定性[26]。

4 小结

本研究以临床医学专业学生为研究对象，探究了医学生职业认同在心理资本和职业决策自我效能感关系中的中介作用。结果显示，良好的心理资本水平可以促进医学生职业认同，从而对职业决策自我效能感产生正向影响。这显示医学院校若要加强医学生职业决策自我效能感，需更注重培养积极且健康的心理资本，注重培育与提升医学生职业认同感。本研究不足之处是存在专业局限。今后研究可以将中医学、儿科和全科等专业学生纳入研究范围，研究对象可以扩大至医学研究生，进一步对医学生职业决策效能感的变化趋势与影响因素进行讨论，以得到更为全面的研究结果，制订更有效的医学生职业决策效能感提升方案。

利益冲突声明：本文所有作者均声明不存在利益冲突。

参考文献

[1] 郑致成，陶磊，王铭泽. 后疫情时代医学生职业认同与职业规划现状及影响因素研究[J].中国卫生事业管理，2022，39（12）：941-945.
[2] 石雪娟，吴师伟，董莉. 心理资本对大学生就业焦虑的影响：自我管理的中介作用[J]. 中国健康心理学杂志，2023，31（10）：1558-1564.
[3] 王秋红，李三阳，罗新红，等. 生命意义感在医学院校大学生心理健康状况、职业决策自我效能感间的中介作用[J]. 中国健康心理学杂志，2023，31（12）：1893-1898.
[4] 沈贺，马虎，吕少博，等. 社会支持对青少年足球运动员职业决策的影响：核心自我评价和心理资本的链式中介效应[J]. 中国健康心理学杂志，2023，31（8）：1234-1238.
[5] 彭玉娇，毛孝容，曹亚雨，等. 心理资本在护生临床实习环境与职业认同间的中介作用[J]. 职业与健康，2022，38（20）：2819-2822，2827.
[6] 张敏，陈庆红. 麻醉科护士职业认同现状及其影响因素分析[J]. 护理学杂志，2023，38（10）：50-53.
[7] 徐慧，巢健茜. 南京市临床护士职业认同的潜在剖面分析[J]. 医学与社会，2023，36（6）：53-58.
[8] 张丽莉. 医学生职业认同现状及影响因素研究[D]. 上海：华东师范大学，2010.
[9] 张阔，张赛，董颖红. 积极心理资本：测量及其与心理健康的关系[J]. 心理与行为研究，2010，8（1）：58-64.
[10] 彭永新，龙立荣. 大学生职业决策自我效能测评的研究[J]. 应用心理学，2001（2）：38-43.
[11] 刘智新，崔宇，赵子华，等. 组态视角下全科医学规培生职业认同的多重并发因果关系与多元提升路径分析[J]. 中国卫生事业管理，2022，39（8）：617-620，640.
[12] 孙景贤，王爱红，刘月仙，等. 护理专业学生专业认同建构体验质性研究的系统评价[J]. 中华护理教育，2022，19（4）：331-338.
[13] 侯朝铭，柏丁兮，高静，等. 认知重评对医学生职业认同影响：学业情绪和学习动机的多重链式中介效应[J]. 中国卫生事业管理，2023，40（2）：142-147.
[14] 郭瑞泽，赵娟，王晨，等. 执业环境对医学生防御性医疗行为倾向的影响分析[J]. 中国医院管理，2023，43（2）：29-32.
[15] 杨晓庆，王计生. 红医精神融入医学生实践育人体系：困境、价值与路径——基于成都医学院的实践思考[J]. 中国卫生事业管理，2023，40（8）：622-625.
[16] 曹虹，汪家龙，李惠萍，等. 心理资本在高职护生社会支持与职业决策自我效能间的中介效应[J]. 蚌埠医学院学报，2020，45（5）：653-657.
[17] 严妮，袁文艺，任立. 高校学生新业态认知对就业意愿的影响研究——基于计划行为理论的实证分析[J]. 社会保障研究，2023（3）：80-91.
[18] 朱礼敬，吕利明，刘培培，等. 护生职业决策自我效能的特点及相关研究进展[J]. 中国医药导报，2020，17（2）：41-43，47.
[19] 王晔安，郑广怀，朱苗. 职业支持：社会认同理论与职业认同的新维度[J]. 社会发展研究，2021，8（1）：52-75，242.
[20] 陈兆军，郭建鹏，裴水妹. 实习实训体验对大学生职业决策自我效能感的影响——基于民办本科院校的案例研究[J]. 教育学术月刊，2022（2）：71-80.
[21] 李斌，朱钦，张凯迪，等. 删繁就简：自愿简约及其前因、后果和理论解释[J]. 心理科学，2024，47（4）：918-925.
[22] 武晓伟，万芝敏. 女大学生心理资本、人际交往与主观幸福感的关系研究[J]. 山东女子学院学报，2023（2）：79-87.
[23] 郑亚楠，廖慧云，胡雯，等. 公正世界信念在赣州市高年级医学生积极心理资本与焦虑水平之间的中介作用[J]. 医学与社会，2021，34（10）：100-103.
[24] 刘洋，李杰，高晓萌，等. 临床医学专业学生工匠心理对职业成熟度的影响：职业决策自我效能的中介作用[J]. 中国健康心理学杂志，2022，30（2）：261-266.
[25] 丁坤，杨晓静，张康娣，等. 大学生心理应激及成就动机与心理资本的关系[J]. 中国学校卫生，2021，42（11）：1645-1649.
[26] 于晨，吴红斌，王维民，等. 中国临床医学生职业认同现状分析[J]. 中国公共卫生，2023，39（4）：437-441.

（收稿日期：2025-03-24）
（修回日期：2025-06-12）

中国知网 https://www.cnki.net
