# Supplementary material for: A study on the current status and influencing factors of psychological capital among medical undergraduates
Source: Front Psychol. 2026 Oct 30;17:1830730. doi: 10.3389/fpsyg.2026.1830730 (PMC13759799; doi:10.3389/fpsyg.2026.1830730)
Supplement: Supplementary file 1 [file Data_Sheet_1.ZIP › References PDF/The Relationship between Academic Stress and Academic Burnout among Medical Students The Mediating Role of Psychological Capital.pdf]

●教学管理

医学生学业压力与学业倦怠的关系：心理资本的中介作用

于增艳，李　平，曹运华，张英华，王　娜，赵慧童*

（齐齐哈尔医学院精神卫生学院，黑龙江 齐齐哈尔 161006）

［摘要］　目的：探究医学生心理资本在学业压力与学业倦怠之间的中介作用。方法：在黑龙江某医学院校选取 364 名医学生进行问卷调查。结果：学业压力可以显著正向预测学业倦怠，心理资本在二者之间起到部分中介作用，中介效应值是 0.36，中介效应占 51.34%。结论：提升学生的心理资本有助于其更好地应对学业压力，从而减轻学业压力对学业倦怠的负面影响。

［关键词］　医学生；学业压力；学业倦怠；心理资本　DOI：10.3969/j.issn.1002-1701.2026.02.020

［中图分类号］　G444　［文献标识码］　A　［文章编号］　1002-1701(2026)02-0056-03

学业倦怠是指学生在面对学习任务时，由于内在动机的缺失或外部压力的累积，经历的一种心理状态[1]。这种状态特征表现在对学习活动的持续兴趣减退、情感上的疲劳感、成就感的下降以及对学业成功的期望降低[2]。此外，学业倦怠还表现为一系列逃避学习的行为模式，如拖延、减少学习投入、消极应对学业要求等。长期来看，它们会对学生的学习成效、心理健康以及未来的职业发展产生不利影响。已有研究表明，学业压力是学业倦怠的重要影响因素，它可以正向预测学业倦怠[3]。而心理资本作为一种积极的心理状态，包括自我效能、希望、乐观和韧性等维度[4]，研究表明心理资本水平较高的学生更不容易产生学业倦怠[5]。然而探究心理资本在学业压力与学业倦怠之间作用的研究相对匮乏，尤其是针对医学生。为此，以黑龙江省某医学院校为研究对象，探究心理资本在学业压力与学业倦怠关系之间的作用，为提升医学生心理健康水平，降低学业压力对学业倦怠的消极影响提供一些参考依据。

一、对象与方法

（一）研究对象。

采取整群抽样的方式对黑龙江省某医学高校大学生进行问卷调查，从大一到大五累计共发放问卷 400 份，其中有效问卷 364 份，有效率为 91.0%。

（二）研究工具。

1. 学习压力量表。采用梁宝勇编制，高登峰修订的大学生学习压力量表[6]，对医学生的学业压力进行评定。该量表共包含 16 个事件，采用两点计分，“没有经历”计 0 分，“经历过”计 1 分。得分越高，学习压力越大。本研究中该量表的 α 系数为 0.887。

2. 学习倦怠量表。采用连榕等编制的大学生学习倦怠量表来评定大学生学习倦怠状况[7]。该量表共包含 20 道题目，采用 5 点计分，总分越高则表明学习倦怠程度越高。本研究中该量表的 α 系数为 0.951。

3. 心理资本问卷。采用张阔等人编制的心理资本问卷来测量大学生的心理资本水平[8]。该问卷有 26 道题目，采用 7 点计分，包含自我效能、韧性、希望、乐观 4 个维度。分数越高，代表大学生的心理资本越丰富。研究中该量表的 α 系数 0.865，4 个维度 α 系数分别为 0.895，0.888，0.892 和 0.872。

（三）统计学方法。

使用 SPSS 26.0 进行独立样本 t 检验、方差分析、相关分析，并通过 SPSS 插件 PROCESS 4.0 进行中介分析和检验。

二、结　果

（一）医学生的学业压力、学业倦怠以及心理资本在不同人口学特征上的差异比较。

对学业压力、学业倦怠和心理资本在人口学变量上进行差异检验，三者在性别、生源地上均存在显著差异。从性别来看，相对于男生，女生的学业压力、学业倦怠水平比较高，但心理资本较低；从生源地来看，相对于农村和乡镇，城市的学生学业压力、学业倦怠分值最高，但是心理资本却最低，见表 1。

表 1　学业压力、学业倦怠及心理资本在性别和生源地的比较（$\bar{x} \pm s$）

项目	组别	学业压力	学业倦怠	心理资本
性别	男	0.26 ± 0.24	2.74 ± 0.78	2.92 ± 0.55
	女	0.37 ± 0.29	2.91 ± 0.75	2.75 ± 0.51
t		−3.80***	−2.05*	3.06***
生源地	农村	0.27 ± 0.24	2.70 ± 0.77	3.01 ± 0.56
	乡镇	0.30 ± 0.23	2.82 ± 0.73	2.80 ± 0.47
	城市	0.39 ± 0.33	2.97 ± 0.78	2.69 ± 0.53
F		6.77**	3.92*	12.30***

注：* 表示 $P<0.05$，** 表示 $P<0.01$，*** 表示 $P<0.001$，下同。

* 通讯作者　Email：2447003922@qq.com

中国知网　https://www.cnki.net

（二）学业压力、学业倦怠与心理资本之间的关系分析。

首先，通过相关分析探讨学业压力、学业倦怠与心理资本之间关系。结果表明，学业压力与学习倦怠存在正相关，与心理资本存在负相关，学习倦怠与心理资本也表现出负相关（$P<0.01$），见表 2。

表 2　医学生学业压力、学业倦怠与心理资本的相关关系

变量	学习压力	学习倦怠	心理资本
学习压力	1		
学业倦怠	0.254^{**}	1	
心理资本	-0.394^{**}	-0.379^{**}	1

进一步将学业压力作为预测变量，学业倦怠作为结果变量，心理资本作为中介变量进行路径分析。结果显示，学业压力能够直接预测学业倦怠，直接效应（0.34）占总效应（0.71）的 48.65%，学业压力还能够通过心理资本的中介作用预测学业倦怠，中介效应（0.36）占总效应的 51.34%。学业压力对学业倦怠的直接效应以及心理资本的中介效应的 bootstrap95% 的置信区间上下限内均不包含 0，见表 3 和图 1。

表 3　心理资本在医学生学业压力与学业倦怠间的中介效应检验

路径	效应类型	效应值	95% *CI*
学习压力→心理资本→学习倦怠	直接效应	0.34	0.06～0.63
	中介效应	0.36	0.08～0.19
	总效应	0.71	0.43～0.98

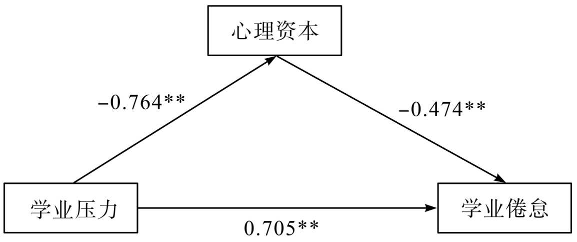

图 1　心理资本在医学生学习压力与学习倦怠之间的中介作用

三、讨　论

（一）学业压力、学业倦怠与心理资本在性别和生源地存在的差异。

本研究发现在人口学变量上存在显著差异。首先，女生的学业压力与学业倦怠水平显著高于男生，但心理资本水平相对较低。这一结果与既往研究结论一致[8-10]。这可能源于社会文化对性别角色的塑造，即女性通常被赋予更高的情绪敏感性和完美主义倾向，使其更易内化学业压力并体验到更深的情感耗竭；而传统社会文化常常鼓励男性展现更高的自信和抗压能力，而女性表达情绪和脆弱则更被接纳。这种文化差异可能使得女大学生在主观报告中展现其心理资本处于劣势，未来可以通过实验研究进一步验证。其次，本研究发现城市生源学生的学业压力与学业倦怠水平最高，但其心理资本得分最低。这与"农村学生处境更艰难"的普遍假设看似矛盾，实则揭示了压力感知的相对性。城市学生由于面临更多的社会比较和教育压力，可能会导致其更高的学业压力和倦怠程度。另外，根据逆境成长理论，面对挑战和困难可以促进个体的心理成长和韧性。相对于城市学生，农村学生可能在成长过程中面临更多早期逆境，这些经历在某种程度上可能有助于他们积累更多的心理资本[11]。

（二）学业压力、学业倦怠与心理资本存在密切关系。

本研究首先表明学业压力与学业倦怠之间存在显著正相关关系，且学业压力对学业倦怠具有正向预测作用。该结果符合应激理论，即学业压力作为外部应激源，当超出了学生的应对能力，就可能会导致学业倦怠的产生。学业倦怠是学生因不能有效应对学业压力而产生的应激反应[12]。其次，本研究结果表明心理资本在学业压力与学业倦怠间起到部分中介作用。心理资本的积累，作为一种保护性因素，它可以在学业压力与学业倦怠之间筑起堡垒[13]。这一点可以从自我决定理论、积极心理学等多个角度进行解释。具体而言，心理资本通过增强学生的自我效能、希望、乐观和韧性等积极心理特质，培养了学生对学业的积极态度和应对策略，有效缓解了学业压力，降低了学业倦怠水平。总之，心理资本的积累能够使医学生更好地应对学业压力，从而维持良好的学业表现和心理健康状态。

（三）教育启示。

随着社会经济发展和教育竞争日益激烈，高校应积极整合心理健康教育与积极心理学理念，着力提升学生的心理资本，从而增强其应对学业压力的能力，降低学业倦怠风险。教师应针对不同学生群体，尤其是女生和城市生源学生，设计和实施个性化的心理支持计划，重点培养其压力管理技巧与自我调节能力。同时，通过鼓励自主学习与加强职业规划指导，能够有效增强学生的自我决定感，激发其内在动机，提升学习满意度。综上所述，高校应采取综合策略，通过强化心理资本、提供个性化支持以及激发内在动机，系统性地提升医学生应对学业压力的能力，减少学习倦怠现象。

［参考文献］

［1］叶钰侨，陆爱桃，张心怡，等. 大学生社会支持对学习倦怠的影响：经济状况和生活满意度的有调节中介模型［J］. 心理研究，2017，10（4）：79-85.

［2］孙晓维. 硕士研究生学业倦怠现状与成因调查研究［D］. 浙江：浙江大学，2019.

［3］刘怡婷，叶宝娟，杨　强. 压力性生活事件对大学生学习倦怠的影响：链式中介效应分析［J］. 中国临床心理学杂志，2019，27（4）：782-784，789.

［4］Luthans F，Avolio BJ，Avey JB，et al. Positive psychological capital：measurement and relationship with performance and

中国知网　https://www.cnki.net

satisfaction[J]. Personnel psychology,2007,60(3): 541-572.
[5] 陈值娥.内地新疆高中班学生生活事件与学习倦怠的关系:积极心理资本的调节作用[D].山东:山东师范大学,2017.
[6] 高登峰.大学生学习压力、心理弹性、心理健康的关系研究[D].湖北:华中科技大学,2008.
[7] 连　榕,杨丽娴,吴兰花.大学生的专业承诺、学习倦怠的关系与量表编制[J].心理学报,2005(5):632-636.
[8] 张　阔,张　赛,董颖红.积极心理资本:测量及其与心理健康的关系[J].心理与行为研究,2010,8(1):58-64.
[9] 张洪峰,刘旭璐.大学生学习压力和学业倦怠与成绩关系的实证研究[J].北京教育(德育),2014(4):4-7.
[10] 余圣陶,许书萍,秦丹萍.工科院校学生心理压力现状及应对策略的研究[J].中国健康心理学杂志,2006(5):568-570.
[11] 熊　猛,叶一舵.心理资本:理论、测量、影响因素及作用[J].华东师范大学学报(教育科学版),2014,32(3):84-92.
[12] 王财玉,李　文,秦寒雪.学业压力的积极应对:自然联结与青少年学业倦怠[J].中国临床心理学杂志,2023,31(3):709-713,629.
[13] 时　宏.医学研究生学业倦怠影响因素、作用路径及其对健康的影响研究[D].吉林:吉林大学,2024.

[作者简介]　于增艳,女,博士,副教授,研究方向:发展心理病理学。

[基金项目]　黑龙江省高等教育教学改革重点委托项目:新文科视域下应用心理学专业核心课课程思政建设路径探索与实践(编号:SJGZ20220149)。

Relationship Between Academic Stress and Academic Burnout Among Medical Students: The Mediating Role of Psychological Capital

Yu Zengyan, Li Ping, Cao Yunhua, Zhang Yinghua, Wang Na, Zhao Huitong*
(*School of Mental Health*, *Qiqihar Medical College*, *Qiqihar* 161006, *Heilongjiang*, *China*)

Abstract: Objective: To explore the mediating role of psychological capital in the relationship between academic stress and academic burnout among medical students. Methods: A questionnaire survey was conducted among 364 medical students from a medical university in Heilongjiang Province. Results: Academic stress significantly and positively predicted academic burnout, and psychological capital played a partial mediating role between them, with a mediation effect value of 0.36, accounting for 51.34% of the total effect. Conclusion: Enhancing students' psychological capital helps them cope with academic stress more effectively, thereby reduce the adverse impact of academic stress on academic burnout.

Keywords: medical students; academic stress; academic burnout; psychological capital

(上接第55页)

[作者简介]　徐子涵,女,本科生在读,研究方向:预防医学。

[基金项目]　山东省教育科学"十四五"规划2023年度课题:生成式人工智能在高等医学教育中的价值、风险及防控策略研究(2023ZC421);2024年度全省卫生健康人力资源管理研究课题:山东省公共卫生人才培育体系现状、问题及对策研究(2024RLZY008);2025年度卫生健康人力资源管理研究课题:山东省精神心理专业人才培养体系建设现状、问题及对策研究(2025RLZY034)。

A Multidimensional Research on the Differences in Medical Students' Learning Adaptability: A Comparison of Gender, Grade, Geographic Origin, and Behavioral Types

Xu Zihan[1], Zhang Qiumei[1], Gao Li[2*]
(1. *School of Public Health*, *Jining Medical University*, *Jining* 272002, *Shandong*, *China*;
2. *Institute of Behavioral Medicine Education*, *Jining Medical University*, *Jining* 272002, *Shandong*, *China*)

Abstract: Objective :To explore the differences in learning adaptability among medical students with different behavioral types. Methods: Type A Behavior Pattern Scale (TABP) and the Learning Adjustment Scale (LAS) were used to conduct a comparative study on the behavioral types and learning adaptability of medical students from a medical school in Shandong Province. Results: Among 2,449 medical students, the detection rates of Type A, intermediate Type A, Type M, intermediate Type B, and Type B behavioral patterns were 4.94%, 13.31%, 5.60%, 32.71%, and 43.44%, respectively. The average factor scores of learning motivation, teaching model, learning ability and learning attitude between male and female students have statistically significant differences ($P<0.05$). All the 5 average score of LAS of medical students from different grades exhibited slightly different, and the differences in teaching model and learning ability has statistically significant ($P<0.05$). All the 5 average score of LAS of medical students from urban and rural areas exhibited slightly different, but all the differences has no statistically significant ($P>0.05$). All the 5 average score of LAS of medical students with different behavioral types exhibited slightly significant differences ($P<0.05$). Conclusion :Medical students from different geographical origins demonstrate similar learning adaptability, there are certain differences among students from different grades, more pronounced differences between genders. Additionally, significant variations exist in learning adaptability among students with different behavioral types.

Keywords: medical students; learning adaptability; comparative study

中国知网　https://www.cnki.net
