# Supplementary material for: A study on the current status and influencing factors of psychological capital among medical undergraduates
Source: Front Psychol. 2026 Oct 30;17:1830730. doi: 10.3389/fpsyg.2026.1830730 (PMC13759799; doi:10.3389/fpsyg.2026.1830730)
Supplement: Supplementary file 1 [file Data_Sheet_1.ZIP › References PDF/The Relationship Between Psychological Capital and Family Satisfaction Among Medical Students.pdf]

察。7例脏器侵犯的患者，术后予以长春新碱＋氨甲蝶呤化疗，对患者随访未发现复发。化疗对腹壁硬纤维瘤治疗效果，仍需要大样本、长时间的观察给予验证。

参考文献

[1] 尹森琴，刘勇强，张国正.腹壁硬纤维瘤的临床特点与CT表现[J].浙江实用医学，2014，19(5)：359-361.

[2] Meyerson SL，D′Amico TA. Intrathoracic desmoid tumor：brief report and review of literature[J]. J Thorac Oncol，2008，3(6)：656-661.

[3] Catania G，Ruggeri L，Iuppa G，et al. Abdominal wall recnsruction with intraperitoneal prostthesis in desmoid tumors surgery[J]. Updata Surg，2012，64(1)：43-48.

[4] Shinagare AB，Ramaiya NH，Jagannathan JP，et al. A to Z of desmoid tumors[J]. AJR AM J Roentgenol，2011，197(6)：1008-1014.

[5] Wong SL. Diagnosis and management of desmoid tumors and fibrosarcoma[J]. J Surg Oncol，2008，97(6)：554-558.

[6] 陈坤，张增芳，王克，等.腹壁韧带样瘤的临床与超声分析[J].中华肿瘤防治杂志，2011，18(12)：965-967.

[7] 焦丹，田稳，隋国庆.腹壁韧带样瘤的超声诊断价值[J].中国实验诊断，2015，19(8)：1412-1413.

[8] 严骏燕，刘金有.CT对腹壁韧带样瘤的诊断价值[J/CD].中华疝和腹壁外科杂志（电子版），2014，8(2)：116-118.

[9] Galdwell EH. Desmoid tumor：muscloaponeurotic fibnosis of the abdominal wall[J]. Surgery，1976，79(1)：104-106.

[10] 孙志国，王猛，朱卫红.腹壁韧带样瘤16例临床分析[J/CD].中华疝和腹壁外科杂志（电子版），2012，6(1)：578-581.

[11] 顾伟青，王强，杨徐雄.腹壁韧带样瘤诊治特点[J].齐齐哈尔医学院学报，2012，33(21)：2902-2903.

[12] 杨小梅，乔宠，周颖，等.妊娠期妇女腹壁韧带样瘤1例报告并文献复习[J].中国实用妇科与产科杂志，2014，30(4)：308-311.

[13] 黄恺.韧带样瘤诊治进展及热点问题[J].中国癌症杂志，2010，20(3)：227-230.

（收稿日期：2016-09-21　修回日期：2016-10-19）

·经验交流·　doi：10.3969/j.issn.1671-8348.2017.04.034

医学生心理资本与家庭满意度的关系研究

周　丽，赖　文，廖雪萍，石素芝，林　凤，李绪莉
（攀枝花学院医学院，四川攀枝花 617000）

［摘要］ 目的　了解医学生心理资本及家庭满意度的现状及两者的相关性。方法　采用积极心理资本问卷，家庭满意度问卷对150名医学生进行调查。结果　(1)医学生的心理资本的自我效能、希望、韧性及乐观得分分别为(31.35±5.06)、(29.5±3.63)、(29.2±4.65)、(28.9±5.45)、(28.9±5.45)分，医学生的家庭满意度的家庭教养方式、家庭成员的沟通了解、家庭环境得分分别为(25.5±4.70)、(27.8±6.87)、(42.3±8.80)分，均在中点以上。(2)医学生的心理资本的自我效能、希望与家庭满意度各因子，韧性与家庭教养方式因子，乐观与家庭教养方式和家庭成员的沟通了解因子呈正相关关系($P<0.05$)。结论　医学生的心理资本状况相对较好。家庭环境、家庭成员的沟通了解和家庭教养方式是影响其心理资本的重要因素，提高医学生对家庭的满意度有助于提高和改善其心理资本。

［关键词］ 学生，医科；心理资本；家庭满意度
［中图分类号］ B849　　［文献标识码］ B　　［文章编号］ 1671-8348(2017)04-0528-03

随着社会的进步、社会竞争越来越激烈，在社会与学校间跨度的大学生也承受着越来越多的压力。家庭的期待、情感的成熟、学业的压力、生活与就业的压力等众多的问题困扰着大学生，焦虑、抑郁、敌对等心理问题随之出现得也越来越多，因此大学生的心理健康受到广泛的社会关注。心理资本是Luthans等[1]2002年在积极心理学和积极组织行为的框架下提出的，是“个体在成长和发展过程中呈现出来的一种积极心理状态”，既往研究发现大学生心理健康与心理资本密切相关，大学生心理资本的得分越高，越具备积极的心理能力，其心理健康的水平越高[2-4]。因此提高大学生的心理资本对提高大学生心理健康水平起到重要作用。医学生承受着比别的专业更重的学业与责任，良好的心理资本对其显得尤为重要。作为个体所拥有的积极心理资源，可通过开发和管理提升心理资本[4]。而家庭是每个孩子走向社会的港口，家庭是影响个体心理品质极其重要的因素。那么家庭与心理资本存在着怎样的关系？家庭是否影响学生心理资本形成和提升？本文对此进行研究，希望找到影响医学生心理资本积累的因素及途径，从而挖掘学生潜能，促进医学生的心理健康。

1　资料与方法

1.1　一般资料

依据既往医学生心理相关研究[5-6]进行统计学样本估计后，从四川省某医学院校，按整群随机抽样法抽出4个班级的学生进行问卷调查，收回问卷150份。除去一份无效问卷，有效问卷149份。其中男生67名、女生82名；年龄18～22岁，平均(19.79±2.42)岁。

1.2　方法

1.2.1　积极心理资本问卷[7]

问卷包括4个维度：分别为自我效能、韧性、希望、乐观。题目的评价按Liken式七点量表进行。该量表的内部一致性α系数全量表为0.79，自我效能、韧性、希望、乐观均在0.70以上，全量表及各分量表具有良好的信、效度。

作者简介：(1974－)，副教授，硕士，主要从事心理学研究。

(C)1994-2023 China Academic Journal Electronic Publishing House. All rights reserved.　http://www.cnki.net

1.2.2　家庭满意度问卷[8]　量表由23个条目组成，包括家庭教养方式，家庭成员的沟通了解和家庭环境3个因子。对问卷进行因子分析，各因子的再测信度在0.671～0.823之间，构成各因子条目的因素负荷取值在0.458～0.753之间。填表前征得知情同意。问卷工作在一个独立的安静房间内进行，答卷学生之间不得相互交流，用统一的指导语，各问卷经核查无遗漏后收回试卷。

1.3　统计学处理　采用SPSS17.0软件对数据进行分析，计量资料采用$\overline{x}\pm s$表示，相关性分析采用Person积差相关分析。以$P<0.05$为差异有统计学意义。

2　结　果

2.1　医学生心理资本及家庭满意度现状　医学生心理资本及家庭满意度各因子得分均高于中值。其中希望、家庭教养方式、家庭环境尤为显著，见表1。

表1　医学生心理资本及家庭满意度现状($n=149$)

项目	得分($\overline{x}\pm s$)	中值
自我效能	31.35±5.06	28
韧性	29.2±4.65	28
希望	29.5±3.63	24
乐观	28.9±5.45	24
家庭教养方式	25.5±4.70	18
家庭成员的沟通了解	27.8±6.87	21
家庭环境	42.3±8.80	30

2.2　医学生心理资本与家庭满意度相关分析　除了韧性因子与家庭成员的沟通了解、家庭环境因子，乐观因子与家庭环境因子不相关外，心理资本其余各因子与家庭满意度因子均呈正相关，差异有统计学意义($P<0.05$，$P<0.01$)，见表2。

表2　医学生心理资本与家庭满意度相关分析(r)

因子	自我效能	韧性	希望	乐观
家庭教养方式	0.326^b	0.185^a	0.379^b	0.272^b
家庭成员的沟通了解	0.228^b	0.113	0.210^b	0.265^b
家庭环境	0.184^a	0.143	0.147^a	0.152

^{a}：$P<0.05$，^{b}：$P<0.01$。

3　讨　论

心理资本的自我效能是指个体能面对各种挑战并争取成功，有胜任工作的自信；乐观是指个体对现在和未来持积极态度，具有积极的归因方式；韧性是指个体能从失败和逆境中迅速恢复，甚至积极改变和成长；希望则是通过各种途径努力实现既定目标的积极动机状态[4]。本研究发现医学生的心理资本得分均高于中值，现状较好。青少年家庭满意度是青少年在与家庭充分互动的基础上，在个体评价方式、态度取向影响下产生的对于家庭教育、教育方式等方面相对稳定的、具有个体特异性的综合态度[9]。本研究中医学生对家庭满意度的现状是基本满意的。

本研究结果显示家庭教养方式与自我效能、韧性、希望和乐观都成正相关，提示家庭教养方式与心理资本之间的相关性显著。这可能因为家庭被称为创造人类性格的工厂，家庭教养方式会直接影响到孩子性格的形成，栾雅松等[10]的研究中就指出：父母教养方式对孩子的生活满意度有一定的影响，严厉惩罚、过度保护型教养方式更容易使孩子对家庭不满意，而产生自卑、胆怯、退缩，体验不到生活的乐趣，而民主型的教养方式中父母能平等对待教育子女，这类家庭中成员之间平等、随和、谅解、互相爱护、关心，父母教养时多对子女鼓励和诱导，而对子女的缺点、错误能适当地批评指正，逐渐培养了子女对别人坦诚友好、自尊、自立、大方、热情，能接受批评，承受压力，关心他人，有独立处事的能力。因此良好的家庭其教养方式可能使孩子更加自信、乐观，坚韧和充满希望，心理资本也就越好。

在本研究中家庭成员的沟通了解与自我效能、希望和乐观这些因子的相关性显著。这可能是由于家庭成员之间的沟通是一种成员间进行的互动过程，自小生活在家庭中，孩子会在很大程度上通过对家庭成员交往方式的观察、学习，形成自我的交往方式[9]。在沟通良好的家庭中父母对子女的想法、行为方式能够较好地理解，而子女也能从与父母的沟通中学习到分析、处理事情的能力和方法，家庭成员的有效沟通能使孩子理解沟通的重要性，学习更多的沟通技巧，从而善于沟通。当遇见问题的时候能够通过向别人倾述来缓解自己的压力，同时能够获得有效的解决方法。因此说明家庭成员的沟通了解是影响大学生心理资本的重要条件之一，会影响学生的自信、乐观和希望。

本研究中医学生家庭环境与自我效能和希望呈正相关。这可能与家庭是一个人生活和成长的首要场所，家庭环境如何，直接决定和影响其是否能够健康成长相关。个体价值观念的形成、生活目标的确立、行为方式的养成、生活技能的掌握、社会规范的接受和社会角色的培养等，最初都是在家庭中完成的[11]。良好的家庭环境促使学生从小保持自信的心态，看待问题的时候总是充满希望能找到事物发展好的一面，也更积极通过各种途径实现生活目标。因此拥有良好家庭环境的人，其心理资本往往较强。家庭环境是大学生对家庭满意与否的重要组成部分，更是大学生心理资本培养的基础。

综上所述：医学生的心理资本及对家庭的满意度基本较好，家庭满意度越好，其心理资本的得分越高。因此，家庭方面是减少医学生心理问题发生的重要影响因素之一。通过引导家长给孩子创造一个良好的家庭环境，与孩子多做有效沟通，采用正确的教养方式来提高孩子的心理资本，提高其抗挫能力，可在心理问题出现前进行有效的早期干预。

参考文献

[1] Luthans F，Avey JB，Avolio BJ. Psychological capital-evelopment：toward a micro-intervention[J]. J Organiz Behav，2006，27(3)：387-393.
[2] 廖军和，李志勇，欧阳儒阳，等.贫困大学生压力知觉与心理健康的关系[J].中国特殊教育，2015，22(5)：91-96.
[3] 孟林，杨慧.心理资本对大学生学习压力的调节作用[J].河南大学学报(社会科学版)，2012，52(3)：142-150.
[4] 黄领仪，陈婷，王树青.大学生就业困难原因分析与指导政策[J].职业，2010，7(21)：28-29.
[5] 王旭美，谢晖.医学本科生健康促进生活方式与学校适应相关性分析[J].中国学校卫生，2013，34(2)：160-162，165.
[6] 徐灿，李巍.重庆医科大学临床本科生与英语本科生的心理健康与社会支持现状及问题[J].重庆医学，2014，43(9)：1113-1114，1116.
[7] 张阔，张赛，董颖红.积极心理资本：测量及其与心理健康的关系[J].心理与行为研究，2010，8(1)：58-64.

(C)1994-2023 China Academic Journal Electronic Publishing House. All rights reserved.　http://www.cnki.net

[8] 郑玉萍，邱海棠，蒙华庆，等.青少年家庭满意度量表结构探索与验证性因素分析[J].中华行为医学与脑科学杂志，2010，19(4)：374-376.
[9] 邱海棠，罗庆华，蒙华庆，等.青少年家庭满意度的理论构建及量表的初步编制[J].中国心理卫生杂志，2007，21(9)：606-609.
[10] 栾雅松，曹建琴，周郁秋，等.大庆市大学生社交焦虑障碍患病率及成长环境因素调查[J].中国行为医学与脑科学杂志，2014，23(9)：793-796.
[11] 武瑞娜，韦丽琴，侯瑞丽，等.家庭养育环境对学龄前儿童心理行为发育的影响[J].包头医学院学报，2014，30(2)：140-142.

（收稿日期：2016-09-22　修回日期：2016-10-20）

·经验交流·　doi：10.3969/j.issn.1671-8348.2017.04.035

茵栀黄口服液联合清蛋白治疗新生儿病理性黄疸疗效及安全性研究

徐睿霞[1]，王甫琴[2]，吴亚臻[1]
（1.青海大学附属医院儿科，西宁 810000；2.新疆五家渠市 103 团医院儿科，新疆五家渠 831304）

［摘要］ 目的　探讨茵栀黄口服液联合清蛋白治疗新生儿病理性黄疸疗效、安全性及可能机制。方法　收集 150 例新生儿病理性黄疸患儿，分为对照组 75 例和观察组 75 例。常规治疗基础上，对照组给予清蛋白，而观察组则联合给予茵栀黄口服液治疗。比较对照组和观察组患儿临床疗效、血清胆红素水平、胆红素下降速度、血清超敏 C 反应蛋白(hs-CRP)、甲胎蛋白(AFP)、转铁蛋白(TRF)水平及不良反应发生情况。结果　疗效评价显示，观察组患儿治疗总有效率明显高于对照组(96.0% *vs*. 86.7%，$P<0.05$)。治疗后，与对照组相比，观察组患儿血清胆红素水平显著降低(110.2±17.1)μmol/L *vs*. (148.8±19.1)μmol/L，胆红素日均下降程度更明显[(45.1±4.5)μmol·L^{-1}·d^{-1} *vs*. (28.3±5.2)μmol·L^{-1}·d^{-1}]，退黄时间明显缩短[(5.1±1.8)d *vs*. (7.6±1.6)d]，血清 hs-CRP、AFP 水平明显降低[(7.3±1.6)mg/L *vs*. (13.7±2.2)mg/L，(13.2±2.1)mg/L *vs*. (18.1±2.5)mg/L]，TRF 水平明显升高(1.97±0.28)g/L *vs*. (1.64±0.24)g/L，组间比较差异均有统计学意义(均 $P<0.05$)。治疗期间，两组患儿均未出现严重不良反应病例，观察组不良反应发生率明显低于对照组(8.0% *vs*. 20.0%，$P<0.05$)。结论　茵栀黄口服液联合清蛋白治疗新生儿病理性黄疸疗效可靠，能够显著提高临床疗效，临床上值得进一步研究。
［关键词］ 茵栀黄口服液；清蛋白；黄疸，新生儿；C 反应蛋白；甲胎蛋白类；转铁蛋白

［中图分类号］ R722.17　　［文献标识码］ B　　［文章编号］ 1671-8348(2017)04-0530-03

病理性黄疸是临床上常见的新生儿疾病，发病率高，以皮肤、巩膜、黏膜黄染为临床表现。新生儿的血脑屏障尚未发育成熟，当患儿体内胆红素不能迅速恢复至正常水平，疾病进展时，胆红素可以通过血脑屏障，损伤神经系统，轻者遗留神经系统后遗症，严重者甚至导致患儿死亡[1-2]。多项研究表明，蓝光照射与药物联合作用疗效优于单独蓝光治疗，然而联用药物的选择与方案却尚无统一共识。清蛋白可以迅速结合水溶性胆红素，增加血清胆红素清除率[3]，茵栀黄口服液可以增加胆管收缩功能，从而使胆汁排出增加，起到利胆退黄作用[4]。在蓝光照射基础上联用清蛋白和茵栀黄口服液可能以不同机制协同提高治疗新生儿病理性黄疸疗效，然而目前尚无相关报道。本研究旨在探讨茵栀黄口服液联合清蛋白治疗新生儿病理性黄疸疗效、安全性及可能的机制，以期为该疾病的治疗提供一定参考依据。

1　资料与方法

1.1　一般资料　收集 2015 年 1－10 月于青海大学附属医院接受治疗的 150 例符合纳入标准的新生儿病理性黄疸患儿作为研究对象，分为对照组 75 例和观察组 75 例。所有患儿均符合新生儿病理性黄疸的相关诊断标准[5]，且不需要换血治疗，并排除核黄疸、阻塞性黄疸、心肺肝肾功能不全、感染性疾病等患儿。所有入选患儿家属对具体治疗方案知情同意，自愿参与本研究，并签订知情同意书。其中对照组患儿男 40 例，女 35 例，日龄(12.6±4.1)d；胎龄(38.7±4.2)周；出生体质量(3.4±0.8)kg。观察组患儿男 42 例，女 33 例，日龄(11.8±3.7)d；胎龄(38.2±4.4)周；出生体质量(3.2±0.6)kg。对照组和观察组患儿一般资料比较差异无统计学意义(均 $P>0.05$)，具有可比性。

1.2　方法　患儿入选后，均给予保暖，改善缺氧，抗感染，纠正水电解质、酸碱平衡紊乱，营养支持等一般治疗及蓝光照射治疗。在此基础上，对照组患儿给予清蛋白(上海新兴医药股份有限公司，国药准字 S20033048)治疗，剂量：1 g/kg 静脉滴注，1 次/d。而观察组患儿在对照组基础上联合给予茵栀黄口服液(北京华润高科技天然药物有限公司，国药准字 Z11020607)治疗，给药剂量：5 mL/次，口服，2 次/d。疗程均为 1 周。

1.3　疗效评价及观察指标　1 个疗程结束后，评价新生儿病理性黄疸疗效，标准如下，痊愈：经过治疗，患儿皮肤、巩膜及黏膜黄染完全消退，复查血清胆红素水平恢复正常水平；有效：经过治疗，患儿黄染症状部分消退，复查血清胆红素较治疗前有所下降，但仍未恢复至正常水平；无效：经过治疗，患儿黄染消退不明显甚至加重，血清胆红素下降不明显甚至升高。痊愈及有效病例均为治疗有效。治疗前后，抽取患儿静脉血，检测组间血清超敏 C 反应蛋白(hs-CRP)、甲胎蛋白(AFP)、转铁蛋白(TRF)水平，其中 hs-CRP、AFP 采用酶联免疫吸附试验(ELISA)法检测，TRF 采用全自动生化分析仪检测。

1.4　统计学处理　采用 SPSS16.0 软件对数据进行分析，计量资料以 $\bar{x}\pm s$ 表示，采用 t 检验，计数资料采用 χ^2 检验，以 $P<0.05$ 为差异有统计学意义。

2　结　　果

2.1　临床疗效比较　疗效评价显示，对照组患儿和观察组患儿治疗总有效率分别为 86.7%和 96.0%。统计学显示，观察

作者简介：徐睿霞(1976－)，本科，主治医师，主要从事新生儿疾病方面的治疗工作。

(C)1994-2023 China Academic Journal Electronic Publishing House. All rights reserved.　http://www.cnki.net
