# Supplementary material for: A study on the current status and influencing factors of psychological capital among medical undergraduates
Source: Front Psychol. 2026 Oct 30;17:1830730. doi: 10.3389/fpsyg.2026.1830730 (PMC13759799; doi:10.3389/fpsyg.2026.1830730)
Supplement: Supplementary file 1 [file Data_Sheet_1.ZIP › References PDF/Zhu, Y. L., and Y. Tao (2022). The Relationship Between Effort-reward Imbalance and Positive Psychological Capital of College Students The Mediating Role of Social Support.pdf]

华南师范大学学报(自然科学版) 2022,54(2):120-128
Journal of South China Normal University(Natural Science Edition) doi:10.6054/j.jscnun.2022035

大学生付出-回报失衡与积极心理资本的关系:社会支持的中介作用

朱阳莉[1,2], 陶　云[1*]
(1. 云南师范大学教育学部, 昆明 650500; 2. 云南师范大学学生处, 昆明 650500)

摘要:基于付出-回报失衡模型,以 1 318 名大学生为被试,采用大学生付出-回报失衡问卷、领悟社会支持量表和积极心理资本问卷,探讨社会支持在付出-回报失衡与积极心理资本之间的中介作用及此作用在不同生源地类型群体中的差异性。结果发现:(1)付出-回报失衡感与过度投入显著正相关,与社会支持、积极心理资本显著负相关;过度投入与社会支持显著负相关,与积极心理资本显著正相关;社会支持与积极心理资本显著正相关。(2)社会支持在付出-回报失衡感与积极心理资本之间起部分中介作用。(3)过度投入与积极心理资本之间存在社会支持的遮掩效应。(4)不同生源地类型学生能够调节付出-回报失衡感与社会支持之间的关系,在城市学生中,付出-回报失衡感对感知社会支持程度的影响相较于农村学生而言更加明显。

关键词:付出-回报失衡感; 过度投入; 社会支持; 积极心理资本; 中介作用; 遮掩效应
中图分类号:G444　　**文献标志码**:A　　**文章编号**:1000-5463(2022)02-0120-09

The Relationship Between Effort-reward Imbalance and Positive Psychological Capital of College Students: The Mediating Role of Social Support

ZHU Yangli[12], TAO Yun[1]
(1. Faculty of Education, Yunnan Normal University, Kunming 650500, China;
2. Department of Student Affairs, Yunnan Normal University, Kunming 650500, China)

Abstract: Based on the effort-reward imbalance model, the mediating effects of social support between effort-reward imbalance and positive psychological capital of college students were examined and the question whether the effects were different on urban and rural students was explored. The participants were 1 318 college students, who completed questionnaires, including the Effort-Reward Imbalance for University Students (ERIUS), the Perceived Social Support Scale (PSSS) and the Positive Psycap Questionnaire (PPQ). The results are as follows. First, the effort-reward ratio (ERR) was positively correlated with over-commitment and negatively correlated with social support and positive psychological capital; over-commitment was negatively correlated with social support and positively correlated with positive psychological capital; and social support was positively correlated with positive psychological capital. Second, social support partially mediated the relationship between ERR and positive psychological capital. Third, the relationship between over-commitment and positive psychological capital was suppressed by social support. Fourth, the relationship between ERR and social support was significantly moderated by whether college students were from urban or rural areas. Compared with the rural students, the influence of ERR on social support is more obvious among urban students.

Keywords: effort-reward ratio; over-commitment; social support; positive psychological capital; mediating role; suppressing effect

---

收稿日期: 2021-07-04　　《华南师范大学学报(自然科学版)》网址:http://journal-n.scnu.edu.cn
基金项目: 云南省 2020 年度哲学社会科学规划教育学项目(AFSZ20010)
* 通信作者:陶云,Email:taoyun2011@126.com

(C)1994-2022 China Academic Journal Electronic Publishing House. All rights reserved. http://www.cnki.net

高校大学生作为新时代的奋斗者和生力军,是国家人才的储备库,承担着重大的使命,大学生的优秀心理品质可以提升其核心竞争优势。积极心理资本是指个体在成长和发展过程中展现出来的一种积极心理状态,包括了自我效能、乐观、韧性和希望 4 种积极心理能力[1]。心理资源理论认为积极心理资本作为积极心理学中一种关键的心理资源,是高校大学生在个人发展、学业评价和职业规划等过程中表现出来的积极核心心理要素。已有研究证实,积极心理资本与个体的各项心理健康指标(自尊、焦虑、抑郁和情感平衡等)显著相关[2],不仅可以显著正向预测大学生的学业成绩、就业能力、成就目标定向和生活满意度,还可以调节大学生学习压力与心理抑郁的关系[3-7]。以往研究主要集中于探讨积极心理资本对个体的保护作用,很少有研究探讨积极心理资本的影响因素。积极心理资本作为一种可开发、可干预的积极心理特质,探究其影响因素,进而有针对性培育,对提升大学生积极心理资本存储量具有重要的现实意义。

付出-回报失衡模型(Effort-reward Imbalance Model,ERI Model)[8]认为当个体自我感知付出努力程度高于得到回报程度时,个体会产生失衡感、会过度否定自己,破坏个体的"内稳态",并将这种失衡感转化为对未来的无望感,对未来失去信心,从而引发强烈的负性情绪和持续压力反应。作为一种典型的研究心理问题的工作压力模型,该模型被广泛应用于国内外职业工作领域,近年来,被逐渐拓展应用到大学生心理社会压力的研究中,并验证了其在高等教育环境中的适用性[9]。既有实证研究发现,当个体处于高付出-低回报状态和高过度投入状态时,大学生可能更容易产生心理困扰,增加患抑郁症的风险;学业付出-回报失衡感与大学生不良心理健康、焦虑症、学习倦怠和退学意向显著相关[10-13]。在中国职业工作研究领域,付出-回报失衡的 2 个测量指标(付出-回报失衡感和过度投入)与积极心理资本呈显著负相关[14-15]。一般学习模型认为,处于付出-回报失衡状态的个体会不自觉地通过内隐学习,形成负性自动思维图式,从而影响积极心理资本的存量[16]。基于此,本研究认为付出-回报失衡能够显著预测大学生的积极心理资本。

社会支持是个体直接感受到的来自社会个人(舍友、同学、老师等)或组织(家庭、班级、社团等)在物质或精神上的帮扶和支持。社会支持作为一种外在的支持性资源可以缓冲应激状态对个体的刺激,改变个体对负性生活事件的认知[17],从内部心理动机的维度对个体起到保护的作用,是影响个体积极心理资本水平的外在保护核心变量之一。关于社会支持和心理资本的相关研究表明,个体感知社会支持水平越高,其积极心理资本的储存量也会越多[18]。此外,还有研究发现,负性情绪与社会支持呈负相关,社会支持可以对负性情绪起缓冲作用[19],还可以作为中介变量,在老年人付出回报失衡感与主观幸福感之间、职业心理资本与工作绩效之间起中介作用[20-21]。因此,本研究假设社会支持可以在一定程度缓解付出-回报失衡所引发的负性情绪对积极心理资本的消极影响,即社会支持在大学生付出-回报失衡与积极心理资本间起中介作用。

综上,本研究将付出-回报失衡模型应用于学校环境中,检验该模型在高等教育环境中的适用性,并进一步揭示付出-回报失衡对大学生积极心理资本作用的内在机制,拟为大学生积极心理资本的保持和提升提供有益指导。

1　对象与方法

1.1　研究对象

采取随机取样法,选取云南省某高校的 1 344 名大学生为被试进行问卷调查。选取时间为 2020 年 11 月,因为该月为学校奖助学金发放签字确认月,而学业成绩的高低与奖助学金的获得息息相关,学生在该时期更容易体验到付出-回报失衡感。经统计整理得到有效问卷 1 318 份,有效率为 98%。其中,男生 404 人(30.7%),女生 914 人(69.3%);师范类学生 949 人(72%),非师范类学生 369 人(28%);城市学生 447 人(33.9%),农村学生 871 人(66.1%);独生子女学生 398 人(30.2%),非独生子女学生 920 人(69.8%);家庭经济困难学生 675 人(51.2%),非家庭经济困难学生 643 人(48.8%)。

1.2　研究工具

1.2.1　大学生付出-回报失衡问卷(Effort-reward Imbalance for University Students,ERIUS)　采用楚克群等[22]修订的"大学生付出-回报失衡问卷"评估当前大学生付出-回报失衡程度。该表共 14 个条目,分为付出、回报和过度投入 3 个分量表;用 5

(C)1994-2022 China Academic Journal Electronic Publishing House. All rights reserved.　http://www.cnki.net

点计分,从“完全不符合”到“完全符合”分别计1~5分,其中第6、7、11条目反向计分;付出分量表的得分范围为3~15分,回报分量表的得分范围为6~30分,过度投入分量表的得分范围为5~25分;各分量表得分越高说明个体在该维度的水平越高。本研究中3个分量表的内部一致性信度在0.56~0.73之间。

付出-回报失衡感采用SIEGRIST等[23]提出的付出与回报的加权比率计算,计算公式如下:

$$\mathrm{ERR}=\frac{e}{r*c},$$

其中,ERR(Effort-reward Ratio)代表付出-回报失衡感;e代表付出分量表的总分;r代表回报分量表的总分;c是用于调整付出分量表和回报分量表不同条目数的修正系数,本研究定义如下:

$$c=\frac{\text{付出分量表条目数}}{\text{回报分量表条目数}}=\frac{3}{6}=0.5。$$

SIEGRIST等[23]指出:当付出-回报失衡感的值越接近0时,代表个体处于低付出-高回报的有利状态;当付出-回报失衡感的值大于1时,表示个体处于高付出-低回报的不利状态。基于此,本研究把付出-回报失衡感>1界定为个体付出-回报处于失衡状态,数值越大表明该个体体验到的付出-回报失衡感越强;将付出-回报失衡感≤1界定为个体付出-回报处于未失衡状态,即平衡状态。本研究中付出-回报失衡感的取值范围在0.20~4.29之间。另外,参考陈思璐等[24]的分类方式,本研究将过分投入总分排名前1/3的个体定义为处于高过度投入状态。因过度投入总分在5~25之间,因此本研究中将过度投入>16分的个体界定为处于高过度投入状态,将过度投入≤16分的个体界定为处于低过度投入状态。

1.2.2 积极心理资本问卷(Positive Psycap Questionnaire,PPQ) 采用张阔等[2]编制的“积极心理资本问卷”评估大学生的积极心理资本水平。该问卷共26个条目,分为自我效能、乐观、韧性和希望4个维度;用7点计分,从“完全不符合”到“完全符合”分别计1~7分,其中第8、10、12、14、25条目反向计分,总分范围为26~182分,分数越高代表个体积极心理资本存储量越高。本研究中该量表的内部一致性信度为0.83。

1.2.3 领悟社会支持量表(Perceived Social Support Scale,PSSS) 采用姜乾金[25]编制的“领悟社会支持量表”评估大学生感知到社会支持的程度。该量表共12个条目,分为家庭支持、朋友支持和其他支持3个维度;用7点计分,从“完全不符合”到“完全符合”分别计1~7分,总分范围为12~84分,分数越高代表个体感知到社会支持的程度越高。由于本研究的研究对象为在校大学生,为了更好地反映大学生的社会支持,本研究将量表中的“领导、亲戚、同事”替换为“老师、亲戚、同学”。本研究中该量表的内部一致性信度为0.93。

1.3 统计方法

使用SPSS 23.0,采用平均值($\bar{x}$)、标准差(s)等描述性方法来对研究样本和研究变量的特征进行描述统计;采用Harman单因素检验法对共同方法偏差进行检验;采用独立样本t检验和单因素方差分析进行差异性检验;采用Pearson积差相关考察付出-回报失衡感、过度投入、社会支持和积极心理资本两两之间的相关性。使用Amos 24.0软件进行模型建构,采用偏差校正非参数百分位Bootstrap法重复抽样5 000次(95%置信区间),对社会支持在付出-回报失衡与积极心理资本间的中介作用进行检验,采用多群组路径分析方法对不同生源地类型在模型中的调节作用进行检验。

2 研究结果

2.1 共同方法偏差检验

如对所有条目均采用自我报告法,则易出现共同方法变异,因此,本研究采用Harman单因素检验(Harman's One-factor Test)对所有项目进行未旋转的主成分因素分析[26]。结果表明:共有10个因子的特征根值大于1,揭示了56%的变异,且第一个因子解释的变异量为20.04%,远远小于临界标准(40%)。因此,本研究不存在显著的共同方法偏差。

2.2 各变量描述统计和相关分析

研究结果(表1)表明:专业类型、师范生与否、生源地类型、独生子女与否、困难学生与否在主要研究变量的得分差异均无统计学意义($P>0.05$);男生和女生在过度投入程度和感知社会支持得分差异显著($t_{过度投入}=5.20$,$P_{过度投入}<0.001$;$t_{社会支持}=-2.13$,$P_{社会支持}<0.05$),男生在过度投入程度上的得分高于女生,但是女生在感知社会支持上的得分高于男生。

(C)1994-2022 China Academic Journal Electronic Publishing House. All rights reserved. http://www.cnki.net

表 1　不同人口学特征在研究变量上的差异

Table 1　The difference of demographic factors on the main variables

人口学统计学指标		统计值	付出-回报失衡感	过度投入	社会支持	积极心理资本
性别	男	$\bar{x}\pm s$	0.94±0.37	13.70±3.07	58.53±12.34	116.15±14.82
	女	$\bar{x}\pm s$	0.92±0.28	12.78±2.91	60.09±12.23	115.82±15.07
		t 值	−0.72	5.20***	−2.13*	0.37
师范生与否	师范生	$\bar{x}\pm s$	0.92±0.30	13.02±2.95	59.26±12.34	115.82±15.18
	非师范生	$\bar{x}\pm s$	0.93±0.33	13.15±3.08	60.50±12.10	116.20±14.50
		t 值	−0.55	−0.71	−1.65	−0.41
生源地类型	城市	$\bar{x}\pm s$	0.93±0.29	13.15±3.05	59.81±12.93	116.47±15.90
	农村	$\bar{x}\pm s$	0.93±0.32	13.01±2.95	59.51±11.94	115.65±14.50
		t 值	0.19	0.84	0.42	0.91
独生子女与否	独生子	$\bar{x}\pm s$	0.92±0.29	13.08±3.17	59.88±12.25	116.27±15.76
	非独生子	$\bar{x}\pm s$	0.93±0.31	13.05±2.90	59.49±12.30	115.77±14.65
		t 值	−0.37	0.20	0.53	0.56
困难学生与否	困难生	$\bar{x}\pm s$	0.93±0.33	13.12±2.92	59.35±11.86	115.91±14.83
	非困难生	$\bar{x}\pm s$	0.92±0.28	13.00±3.05	59.88±12.71	115.93±15.17
		t 值	0.66	0.73	−0.79	−0.03

注:N=1 318;检验数值通过四舍五入保留 2 位小数,*** 表示 P<0.001,** 表示 P<0.01,* 表示 P<0.05,下同。

为考察付出-回报失衡感程度和过度投入程度在研究变量(社会支持和积极心理资本)间是否存在显著差异,以二分类后的“付出-回报失衡感”和“过度投入”为分组变量进行独立样本 t 检验。结果(表 2)显示:约 33.08%的大学生处于付出-回报失衡状态;约 10.70%的大学生处于高过度投入状态。经过 t 检验发现:处于付出-回报失衡状态的大学生在社会支持和积极心理资本中的得分均显著低于处于付出-回报平衡状态的大学生,即个体体验到的付出-回报失衡感越强,其感知到社会支持的程度就会越低,积极心理资本的存储量也越少。处于高过度投入状态的大学生在积极心理资本中的得分显著高于处于低过度投入状态的大学生,在感知社会支持得分中差异不显著,说明个体过度投入程度越高,其积极心理资本的储存量也越高。

表 2　不同付出-回报失衡水平下大学生积极心理资本和社会支持的评分比较

Table 2　The comparison of scores of college students' positive psychological capital and social support under different levels of effort-reward imbalance

衡量标准	人数	统计值	社会支持	积极心理资本
付出-回报失衡感≤1	882	$\bar{x}\pm s$	60.61±11.96	117.45±14.67
付出-回报失衡感>1	436	$\bar{x}\pm s$	57.58±12.68	112.82±15.17
		t 值	−4.24	−5.34
		P 值	<0.001	<0.001
过度投入≤16	1 177	$\bar{x}\pm s$	59.80±12.01	115.49±14.61
过度投入>16	141	$\bar{x}\pm s$	58.06±14.29	119.51±17.51
		t 值	−1.39	2.62
		P 值	0.17	<0.05

注:付出-回报失衡感和过度投入均为二分类变量。

采用 Pearson 积差相关分析付出-回报失衡感、过度投入、社会支持和积极心理资本之间的关系。

(C)1994-2022 China Academic Journal Electronic Publishing House. All rights reserved.　http://www.cnki.net

结果(表3)显示:付出-回报失衡感与过度投入显著正相关,与社会支持和积极心理资本显著负相关;过度投入与社会支持显著负相关,与积极心理资本显著正相关;社会支持与积极心理资本显著正相关。

表3 大学生付出-回报失衡感、过度投入与社会支持和积极心理资本的相关分析

Table 3 The descriptive statistical correlation of the main variables

研究变量	付出-回报失衡感	过度投入	积极心理资本	社会支持
付出-回报失衡感	—			
过度投入	0.11**	—		
积极心理资本	-0.13**	0.11**	—	
社会支持	-0.16**	-0.12**	0.35**	—
M	0.93	13.06	115.92	59.61
SD	0.31	2.99	14.99	12.28

注:付出-回报失衡感为连续变量。

2.3 大学生付出-回报失衡对积极心理资本的作用:社会支持的中介作用检验

本研究以付出-回报失衡的2个测量指标(付出-回报失衡感和过度投入)为预测变量、积极心理资本为结果变量、社会支持为中介变量,通过Amos 24.0构建结构方程模型。模型拟合结果显示:χ^2/df=4.86,CFI=0.98,GFI=0.98,IFI=0.98,RMSEA=0.05,SRMR=0.03。模型各拟合指数均符合测量学标准,表明模型拟合良好。各路径系数(图1)显示:付出-回报失衡感显著负向预测社会支持(β=-0.16,SE=0.04,P<0.001),过度投入显著负向预测社会支持(β=-0.11,SE=0.03,P<0.001),社会支持显著正向预测积极心理资本(β=0.46,SE=0.03,P<0.001),付出-回报失衡感显著负向预测积极心理资本(β=-0.13,SE=0.03,P<0.001),过度投入显著正向预测积极心理资本(β=0.12,SE=0.03,P<0.001)。

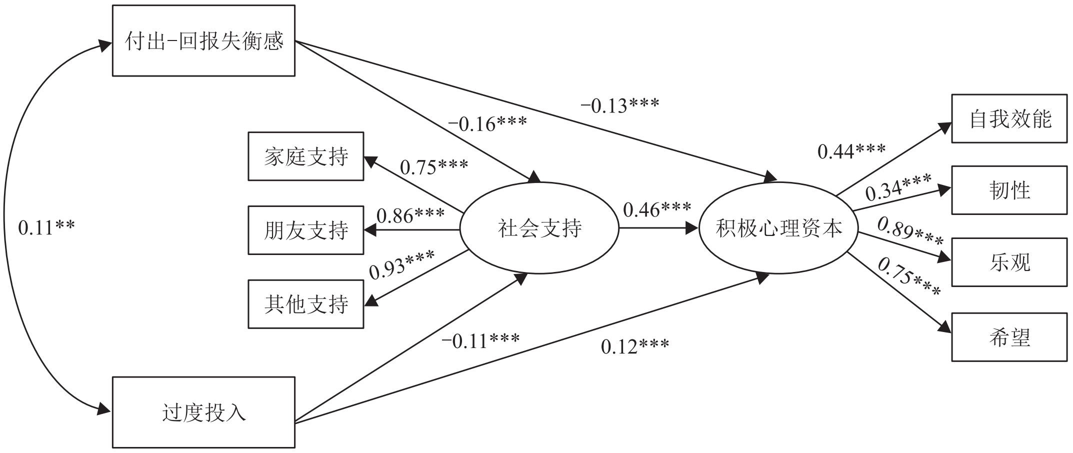

图1 社会支持在大学生付出-回报失衡与积极心理资本间的中介模型

Figure 1 The model of social support mediation between college students' effort-reward imbalance and positive psychological capital

注:模型中所有变量均为连续变量,图中数值均为标准化回归系数。

另外,采用偏差校正的非参数百分位Bootstrap方法重复抽样5 000次,对社会支持在大学生付出-回报失衡(付出-回报失衡感和过度投入)和积极心理资本之间的中介效应进行检验。中介效应的显著性检验结果(表4)表明付出-回报失衡的2个测量指标作用于社会支持和积极心理资本的机制是存在差异的:(1)付出-回报失衡感对积极心理资本的直接效应显著(95%置信区间为[-0.19,-0.07]),社会支持在付出-回报失衡感与积极心理资本的关系中的中介效应显著(95%置信区间为[-0.11,-0.03]),直接效应和中介效应的置信区间均不包含0,说明直接效应和中介效应均有统计学意义,社会支持在付出-回报失衡感与积极心理资本的关系中起到部分中介作用。(2)社会支持在付出-回报失衡感与积极心理资本间的中介效应量为-0.16×0.46=-0.07,直接效应量为-0.13,中介效应量占总效应量的35%。(3)过度投入对积极心理资本的直接效应显著(95%置信区间为[0.06,0.18]),社会支持在过度投入与积极心理资本的关系中的中介效应显著(95%置信区间为[-0.08,-0.02]),直接效应和中介效应的置信区间均不包含0,说明直接效应和中介效应均有统计学意义,社会支持在过度投入

(C)1994-2022 China Academic Journal Electronic Publishing House. All rights reserved. http://www.cnki.net

与积极心理资本的关系中起到部分中介作用。(4)社会支持在过度投入与积极心理资本间的中介效应量为 $-0.11\times0.46=-0.05$，直接效应量为 0.12，两者符号相反，说明过度投入通过社会支持影响积极心理资本的中介效应属于“遮掩效应”[27]，即过度投入会通过社会支持的“遮掩效应”对大学生积极心理资本产生负向削弱作用，遮掩效应量占直接效应量的比例为 41.67%。

表 4　中介效应模型检验各路径分析表

Table 4　The mediating effect of different paths

效应	路径	效应量	标准误	95%置信区间
直接效应	付出-回报失衡感→积极心理资本	-0.13	0.03	[-0.19,-0.07]
中介效应	付出-回报失衡感→社会支持→积极心理资本	-0.07	0.02	[-0.11,-0.03]
直接效应	过度投入→积极心理资本	0.12	0.04	[0.06,0.18]
中介效应	过度投入→社会支持→积极心理资本	-0.05	0.01	[-0.08,-0.02]

2.4　社会支持在大学生付出-回报失衡与积极心理资本间的中介效应跨组比较

为探讨生源地类型在中介模型中是否具有调节效应，本研究采用结构方程模型中的多群组分析方法，来验证生源地类型在中介模型中的差异。由结果(表 5)可知：当限定所有路径系数在不同生源地类型组相等后，模型拟合度变差($\Delta\chi^2=23.31$，$\Delta df=10$，$P<0.05$)，表明模型在城市学生和农村学生群体间差异显著。进一步分析发现不同生源地类型学生仅在“付出-回报失衡感→社会支持”这条路径上存在差异，城市学生、农村学生在该路径中的标准化路径系数分别为 -0.236($P<0.001$)、-0.119($P<0.001$)，参数间差异的临界比率值为 2.254，其绝对值大于1.96[28]，说明不同生源地类型学生在付出-回报失衡感到社会支持这条路径上存在调节作用，在城市学生中，付出-回报失衡感对感知社会支持程度的影响大于农村学生的。

表 5　中介模型在城市学生与农村学生群体间的路径系数差异比较

Table 5　The comparison of path coefficient differences between urban and rural student groups under the mediation model

路径	城市学生		农村学生		临界比率值
	β	P	β	P	
付出-回报失衡感→社会支持	-0.236	0.000	-0.119	0.000	2.254
社会支持→积极心理资本	0.509	0.000	0.430	0.000	-1.148
付出-回报失衡感→积极心理资本	-0.044	0.368	-0.169	0.000	-1.804
过度投入→社会支持	-0.169	0.000	-0.080	0.023	1.537
过度投入→积极心理资本	0.114	0.028	0.130	0.000	0.158

3　讨论

以往关于大学生积极心理资本的实证研究主要是把积极心理资本作为预测变量，主要体现积极心理资本对就业绩效、就业压力、择业效能感、创业意愿、学习倦怠、心理压力、成就动机和社会适应等方面的影响。本研究聚焦于付出-回报失衡和社会支持对积极心理资本的影响，把积极心理资本作为结果变量，探究影响积极心理资本的内部因素和外在因素，为提升大学生的积极心理发展水平提供实证支撑。

本研究结果表明，大学生付出-回报失衡感与积极心理资本显著负相关，这说明大学生感知到付出-回报失衡感的程度越高，其积极心理资本的水平也就越低，这与已有研究结果[14-15]是一致的。究其缘由，稀缺理论认为当个体长期处于付出-回报失衡状态时，个体的意识就会出现“稀缺”，未被满足的需求会俘获个体的大脑，占据个体的认知资源[29]，进而影响个体对自身能力和价值的基本评价，而核心自我评价又与积极心理资本呈显著的正相关关系[30]，因此大学生付出-回报失衡感能显著负向预测积极心理资本。本研究还发现，过度投入与积极心理资本显著正相关，即过度投入程度越高，

(C)1994-2022 China Academic Journal Electronic Publishing House. All rights reserved.　http://www.cnki.net

积极心理资本的存储量也越高,这与已有研究结果[14-15]不一致。可能的原因是,学生群体和职业群体所面临的心理社会压力环境存在差异。在学校教育环境中,过度投入是指学生为了完成或达到预期目标,投入时间、精力和情感的一种应对模式。归因理论认为,当个体认为通过自己努力就可以实现预期目标时,个体会倾向于内归因,为之投入更多的心理资源,即会更加努力,而积极心理资本也是一种积极心理资源。因此,当个体过度投入程度越高,个体对达到预期目标做出积极评价的可能性越大,自信心也越强,进而大学生积极心理资本的存储量也会得到提升。

本研究运用结构方程模型考察社会支持在付出-回报失衡与积极心理资本之间的中介作用。结果表明付出-回报失衡的2个测量指标(付出-回报失衡感和过度投入)通过社会支持作用于积极心理资本的中介机制并不相同。研究发现,付出-回报失衡感这一内部因素是通过社会支持这一外部因素间接影响积极心理资本的,社会支持在付出-回报失衡感与积极心理资本之间起部分中介作用,说明内部因素和外在因素共同影响个体的积极心理资本,这与已有研究结果[20]吻合。社会支持作为一副"缓冲剂",在本研究中发挥的积极作用机制在于可以减少付出-回报失衡感对积极心理资本的消极影响。依据社会支持主效应模型的观点,社会支持可以缓解个体付出-回报失衡感带来的负性体验,即个体获得来自于朋友、教师、家人等的外部支持程度越高时,对生活满意度的主观评价就会越高,更倾向于采取积极、客观的应对方式应对挫折,进而对积极心理资本起到保护性作用。

本研究还发现社会支持在过度投入与积极心理资本的关系中存在遮掩效应,这说明过度投入对社会支持的影响表现为一种"削弱效应",即过度投入程度越高,感知到的外部支持就会越少,越不利于大学生的积极心理资本存储。导致这一结果可能的原因是个体过度投入程度越高,消耗的心理资源越多。根据自我控制的能量模型,个体的自我控制资源是有限的,长期心理资源的消耗会导致个体产生持续性疲劳,进而影响其对来自外界支持的感知。

多群组路径分析发现,不同生源地类型仅在"付出-回报失衡感→社会支持"路径上存在调节作用,在城市学生中,付出-回报失衡感对感知社会支持程度的影响相较于农村学生而言更加明显。究其根源,可能与个体从小所生活的环境相关。生态系统理论认为发展的个体位于生态系统的中心,同时受到微观系统、中间系统、外系统和宏观系统的共同影响[31]。生活在城市的大学生,由于从小所生活环境较好,生活舒适度较高,因此在面对压力、挫折时展现出较低的心理韧性水平,更容易受到负性情绪的影响。既有研究证实,农村群体的心理韧性水平显著高于城市群体[32],因此,付出-回报失衡感对大学生感知社会支持程度的影响在城市大学生这一群体中更加明显。

基于本研究的相关结果,社会支持在付出-回报失衡感负向影响积极心理资本时发挥了缓冲和保护作用,这一结论可启示高校学生教育管理工作者:付出-回报失衡作为个体自我感知的一种内部心理状态,是一种客观存在的现象,直接降低大学生付出-回报失衡程度的难度较大,可以从社会支持这一外在、可控的外在保护因素出发,通过社会支持状况的改善来缓解付出-回报失衡感对积极心理资本带来的消极影响。研究结果有利于高校学生教育管理工作者从社会支持理论视角理解大学生付出-回报失衡感与积极心理资本的关系,加强对大学生社会支持的感知干预,将社会支持作为应对压力事件的能量补充,从而为提升高校大学生积极心理资本存储量提供参考依据。

本研究还存在一些不足之处:一是研究样本来自单一高校,不能代表我国大学生群体总体。今后还需在不同省份不同高校大学生群体中进行重复研究,检验研究结论的外部效度,使得该研究结论具备更强的解释力。二是研究设计采用横断面研究方法,研究结论还不能做出大学生付出-回报失衡与积极心理资本是否存在因果关系的结论,未来可以考虑采用追踪研究来进一步检验本研究成果。

参考文献:

[1] 仲理峰. 心理资本研究评述与展望[J]. 心理科学进展,2007(3):482-487.
ZHONG L F. Review of psychological capital research [J]. Advances in Psychological Science,2007(3):482-487.

[2] 张阔,张赛,董颖红. 积极心理资本:测量及其与心理健康的关系[J]. 心理与行为研究,2010,8(1):58-64.

(C)1994-2022 China Academic Journal Electronic Publishing House. All rights reserved. http://www.cnki.net

ZHANG K,ZHANG S,DONG Y H. Positive psychological capital:measurement and relationship with mental health [J]. Studies of Psychology and Behavior,2010,8(1):58-64.

[3] 张阔,付立菲,王敬欣. 心理资本、学习策略与大学生学业成绩的关系[J]. 心理学探新,2011,31(1):47-53.
ZHANG K,FU L F,WANG J X. The relation of psychological capital learning strategies and academic achievement of undergraduates[J]. Psychological Exploration,2011,31(1):47-53.

[4] 励骅,曹杏田. 大学生心理资本与就业能力关系研究[J]. 中国高教研究,2011(3):54-56.
LI H,CAO X T. A study on the relationship between psychological capital and employability of college students [J]. China Higher Education Research,2011(3):54-56.

[5] 王雁飞,李云健,黄悦新. 大学生心理资本、成就目标定向与学业成就关系研究[J]. 高教探索,2011(6):128-136;148.
WANG Y F,LI Y J,HUANG Y X. A study on the relationship between college students' psychological capital, achievement goal orientation and academic achievement [J]. Higher Education Exploration,2011(6):128-136;148.

[6] 王建坤,陈剑,郝秀娟,等. 大学生学习倦怠对生活满意度的影响——领悟社会支持与心理资本的中介作用[J]. 中国心理卫生杂志,2018,32(6):526-530.
WANG J K,CHENG J,HAO X J,et al. Effect of learning burnout on life satisfaction:mediating effect of psychological capital and perceived social support in college students [J]. Chinese Mental Health Journal,2018,32(6):526-530.

[7] 孟林,杨慧. 心理资本对大学生学习压力的调节作用——学习压力对大学生心理焦虑、心理抑郁和主观幸福感的影响[J]. 河南大学学报(社会科学版),2012,52(3):142-150.
MENG L,YANG H. Effects of undergraduates' academic stress on psychological depression,anxiety and subjective well-being:the moderation role of psychological capital [J]. Journal of Henan University(Social Science),2012,52(3):142-150.

[8] SIEGRIST J. Adverse health effects of high-effort/low-reward conditions[J]. Journal of Occupational Health Psychology,1996,1(1):27-41.

[9] WEGE N,LI J,MUTH T,et al. Student ERI:psychometric properties of a new brief measure of effort-reward imbalance among university students[J]. Journal of Psychosomatic Research,2017,94:64-67.

[10] FABIO P,ROBROEK SUZAN J W,UTE B,et al. Mental health among university students:the associations of effort-reward imbalance and overcommitment with psychological distress[J]. Journal of Affective Disorders,2021,282:953-961.

[11] HENRY H,PETER K,TIMO K,et al. Test anxiety in medical school is unrelated to academic performance but correlates with an effort/reward imbalance[J]. PloS One,2017,12(2):e0171220/1-14.

[12] HILGER-KOLB J,DIEHL K,HERR R,et al. Effort-reward imbalance among students at German universities:associations with self-rated health and mental health[J]. International Archives of Occupational and Environmental Health,2018,91(8):1011-1020.

[13] JOHN W C,SUZANNE D,BRODY H. More pain than gain:effort-reward imbalance,burnout,and withdrawal intentions within a university student population[J]. Journal of Educational Psychology,2018,110(3):378-394.

[14] LIU L,CHANG Y,FU J Y,et al. The mediating role of psychological capital on the association between occupational stress and depressive symptoms among Chinese physicians:a cross-sectional study[J]. BMC Public Health,2012,12(1):219-226.

[15] LIU C,WANG L,ZHAO Q. Factors related to health-related quality of life among Chinese psychiatrists:occupational stress and psychological capital[J]. BMC Health Services Research,2015,15(1):20/1-10.

[16] 高素华. 大学生生活事件与主观幸福感间的作用机制研究——以心理资本和社会支持为中介[J]. 教育学术月刊,2017(3):95-99.

[17] 李俊一,靳宇倡. 暴力视频游戏与亲社会游戏对认知、情感和行为的影响:基于一般学习模型的视角[J]. 中国临床心理学杂志,2014,22(6):985-988;969.
LI J Y,JIN Y C. The effects of violent video games and prosocial video games on cognition,emotion and behavior [J]. Chinese Journal of Clinical Psychology,2014,22(6):985-988;969.

[18] YARCHESKI A,MAHON N E. Meta-analyses of predictors of hope in adolescents[J]. Western Journal of Nursing Research,2016,38(3):345-368.

[19] 冯鹤,刘明芳. 朝鲜族中小学生健康相关生命质量及与社会支持和负性情绪的关联[J]. 中国学校卫生,2021,42(1):73-76;82.
FENG H,LIU M F. Analysis of the association between

(C)1994-2022 China Academic Journal Electronic Publishing House. All rights reserved.　http://www.cnki.net

health-related quality of life with social support and negative emotions among ethnic Korean children and adolescents[J]. Chinese Journal of School Health,2021,42(1):73-76;82.

[20] 李西营,姚雪梅,邵景进. 老年人家庭付出回报不平衡感对主观幸福感的影响[J]. 西南大学学报(社会科学版),2019,45(5):101-108.
LI X Y,YAO X M,SHAO J J. The effect of family effort-reward imbalance on subjective well-being in elderly[J]. Journal of Southwest University (Social Sciences Edition),2019,45(5):101-108.

[21] 李力,郑治国,廖晓明. 高校教师职业心理资本与工作绩效:社会支持的中介效应[J]. 心理与行为研究,2016,14(6):802-810.
LI L,ZHEGN Z G,LIAO X M. University teachers' professional psychological capital and job performance:mediating effect of social support[J]. Studies of Psychology and Behavior,2016,14(6):802-810.

[22] 楚克群,蓝雅惠,谭有模. 付出-回报失衡量表在中国大学生样本中的信度和效度[J]. 中国健康教育,2019,35(6):521-524.
CHU K Q,LAN Y H,TAN Y M. Reliability and validity of the effort-reward scale in Chinese college students[J]. Chinese Journal of Health Education,2019,35(6):521-524.

[23] SIEGRIST J,STARKE D,CHANDOLA T,et al. The measurement of effort-reward imbalance at work:European comparisons[J]. Social Science & Medicine,2004,58(8):1483-1499.

[24] 陈思璐,成诚,茅金凤,等. 江苏省医务人员付出-回报失衡、组织支持与抑郁症状的关系研究[J]. 中国卫生统计,2019,36(1):44-48.
CHEN S L,CHENG C,MAO J F,et al. Effort-reward imbalance,perceived organizational support and depression symptoms among medical staff in Jiangsu Province[J]. Chinese Journal of Health Statistics,2019,36(1):44-48.

[25] 姜乾金. 领悟社会支持量表[M]∥汪向东,王希林,马弘. 心理卫生评定量表手册(增订版). 北京:中国心理卫生杂志社,1999:131-133.

[26] 周浩,龙立荣. 共同方法偏差的统计检验与控制方法[J]. 心理科学进展,2004(6):942-950.
ZHOU H,LONG L R. Statistical remedies for common method biases[J]. Advances in Psychological Science,2004(6):942-950.

[27] 温忠麟,叶宝娟. 中介效应分析:方法和模型发展[J]. 心理科学进展,2014,22(5):731-745.
WENG Z L,YE B J. Analyses of mediating effects:the development of methods and models[J]. Advances in Psychological Science,2014,22(5):731-745.

[28] 荣泰生. AMOS与研究方法[M]. 重庆:重庆大学出版社,2007.

[29] 德希尔·穆来纳森,埃德尔·沙菲尔. 稀缺:我们是如何陷入贫困与忙碌的[M]. 魏薇,龙志勇,译. 杭州:浙江人民出版社,2014.

[30] 臧爽,刘富强,李妍,等. 少数民族医学生心理资本与情绪弹性和核心自我评价的中介关系研究[J]. 中国全科医学,2015,18(12):1436-1440.
ZANG S,LIU F Q,LI Y,et al. Mediate effects of psychological capital appreciation between emotional elasticity and core self-evaluation among the ethnic minority medical students[J]. Chinese General Practice,2015,18(12):1436-1440.

[31] 戴维·谢弗,凯瑟琳·基普. 发展心理学[M]. 9版. 邹泓,谨识,译. 北京:中国轻工业出版社,2016.

[32] 李芳,李长风,刘素,等. 农民工、城市居民、农村居民心理韧性及对心理健康的影响[J]. 现代预防医学,2019,46(22):4150-4153.
LI F,LI C F,LIU S,et al. The status of essential resilience and its impact on mental health among rural-to-urban migrants,rural residents and urban residents[J]. Modern Preventive Medicine,2019,46(22):4150-4153.

【责任编辑:庄晓琼 责任校对:庄晓琼 英文审校:程杰】

(C)1994-2022 China Academic Journal Electronic Publishing House. All rights reserved. http://www.cnki.net
