# Supplementary material for: A study on the current status and influencing factors of psychological capital among medical undergraduates
Source: Front Psychol. 2026 Oct 30;17:1830730. doi: 10.3389/fpsyg.2026.1830730 (PMC13759799; doi:10.3389/fpsyg.2026.1830730)
Supplement: Supplementary file 2 [file Table_1.DOCX]

**Supplementary Table S1.** Bonferroni-adjusted pairwise comparisons of psychological capital and its dimensions by academic year

| **Outcome** | **Academic year (I)** | **Academic year (J)** | **Mean difference (I − J)** | **SE** | **Bonferroni-adjusted 95% CI** | | **Adjusted *p*** |
| --- | --- | --- | --- | --- | --- | --- | --- |
|  |  |  |  |  | **Lower limit** | **Upper limit** |  |
| Self-efficacy | 2021 | 2022 | 0.177 | 0.125 | −0.155 | 0.509 | 0.951 |
|  | 2021 | 2023 | 0.082 | 0.136 | −0.277 | 0.442 | 1.000 |
|  | 2021 | 2024 | −0.126 | 0.117 | −0.436 | 0.183 | 1.000 |
|  | 2022 | 2023 | −0.095 | 0.108 | −0.380 | 0.191 | 1.000 |
|  | 2022 | 2024 | −0.303 | 0.083 | −0.523 | −0.084 | 0.002 |
|  | 2023 | 2024 | −0.209 | 0.098 | −0.468 | 0.051 | 0.201 |
| Resilience | 2021 | 2022 | 0.096 | 0.124 | −0.231 | 0.423 | 1.000 |
|  | 2021 | 2023 | 0.071 | 0.134 | −0.283 | 0.425 | 1.000 |
|  | 2021 | 2024 | −0.127 | 0.115 | −0.432 | 0.178 | 1.000 |
|  | 2022 | 2023 | −0.025 | 0.106 | −0.307 | 0.256 | 1.000 |
|  | 2022 | 2024 | −0.223 | 0.082 | −0.440 | −0.007 | 0.039 |
|  | 2023 | 2024 | −0.198 | 0.097 | −0.454 | 0.057 | 0.244 |
| Hope | 2021 | 2022 | 0.328 | 0.134 | −0.025 | 0.682 | 0.086 |
|  | 2021 | 2023 | 0.077 | 0.145 | −0.306 | 0.459 | 1.000 |
|  | 2021 | 2024 | −0.108 | 0.125 | −0.438 | 0.222 | 1.000 |
|  | 2022 | 2023 | −0.252 | 0.115 | −0.556 | 0.053 | 0.174 |
|  | 2022 | 2024 | −0.437 | 0.089 | −0.671 | −0.203 | <0.001 |
|  | 2023 | 2024 | −0.185 | 0.104 | −0.461 | 0.091 | 0.462 |
| Optimism | 2021 | 2022 | 0.234 | 0.143 | −0.145 | 0.612 | 0.620 |
|  | 2021 | 2023 | −0.087 | 0.155 | −0.497 | 0.323 | 1.000 |
|  | 2021 | 2024 | −0.250 | 0.134 | −0.604 | 0.103 | 0.369 |
|  | 2022 | 2023 | −0.321 | 0.123 | −0.647 | 0.005 | 0.056 |
|  | 2022 | 2024 | −0.484 | 0.095 | −0.735 | −0.233 | <0.001 |
|  | 2023 | 2024 | −0.163 | 0.112 | −0.459 | 0.133 | 0.872 |
| Psychological capital | 2021 | 2022 | 0.203 | 0.113 | −0.097 | 0.503 | 0.440 |
|  | 2021 | 2023 | 0.039 | 0.123 | −0.286 | 0.364 | 1.000 |
|  | 2021 | 2024 | −0.151 | 0.106 | −0.431 | 0.129 | 0.924 |
|  | 2022 | 2023 | −0.164 | 0.098 | −0.422 | 0.094 | 0.554 |
|  | 2022 | 2024 | −0.354 | 0.075 | −0.553 | −0.156 | <0.001 |
|  | 2023 | 2024 | −0.190 | 0.089 | −0.424 | 0.044 | 0.195 |
